# Design and synthesis of new thiazolidinone/uracil derivatives as antiproliferative agents targeting EGFR and/or BRAF^V600E^

**DOI:** 10.3389/fchem.2022.1076383

**Published:** 2022-12-12

**Authors:** Mohammed B. Alshammari, Ashraf A. Aly, Bahaa G. M. Youssif, Stefan Bräse, Akil Ahmad, Alan B. Brown, Mahmoud A. A. Ibrahim, Asmaa H. Mohamed

**Affiliations:** ^1^ Chemistry Department, College of Sciences and Humanities, Prince Sattam Bin Abdulaziz University, Al-Kharij, Saudi Arabia; ^2^ Chemistry Department, Faculty of Science, Minia University, El-Minia, Egypt; ^3^ Pharmaceutical Organic Chemistry Department, Faculty of Pharmacy, Assiut University, Asyut, Egypt; ^4^ Institute of Organic Chemistry, Karlsruher Institut fur Technologie, Karlsruhe, Germany; ^5^ Institute of Biological and Chemical Systems (IBCS-FMS), Karlsruhe Institute of Technology, Karlsruhe, Germany; ^6^ Chemistry Department, Florida Institute of Technology, Melbourne, FL, United States; ^7^ Computational Chemistry Laboratory, Chemistry Department, Faculty of Science, Minia University, Minia, Egypt

**Keywords:** 5-AU, thiourea, thiazolidinone, EGFR, B-RAF, viability, molecular modeling

## Abstract

Thiourea derivatives of uracil were efficiently synthesized *via* the reaction of 5-aminouracil with isothiocyanates. Then, we prepared uracil-containing thiazoles *via* condensation of thioureas with diethyl/dimethyl acetylenedicarboxylates. The structures of the products were confirmed by a combination of spectral techniques including infra-red (IR), nuclear magnetic resonance (NMR), mass spectrometry (MS) and elemental analyses. A rationale for the formation of the products is presented. The newly synthesized compounds were evaluated for their *in vitro* antiproliferative activity against four cancer cell lines. The compounds tested showed promising antiproliferative activity, with GI_50_ values ranging from 1.10 µM to 10.00 µM. Compounds **3c, 5b, 5c, 5h, 5i,** and **5j** were the most potent derivatives, with GI_50_ values ranging from 1.10 µM to 1.80 µM. Compound **5b** showed potent inhibitory activity against EGFR and BRAF^V600E^ with IC_50_ of 91 ± 07 and 93 ± 08 nM, respectively, indicating that this compound could serve as a dual inhibitor of EGFR and BRAF^V600E^ with promising antiproliferative properties. Docking computations revealed the great potency of compounds **5b** and **5j** towards EGFR and BRAF^V600E^ with docking scores of −8.3 and −9.7 kcal/mol and −8.2 and −9.3 kcal/mol, respectively.

## Graphical Abstract

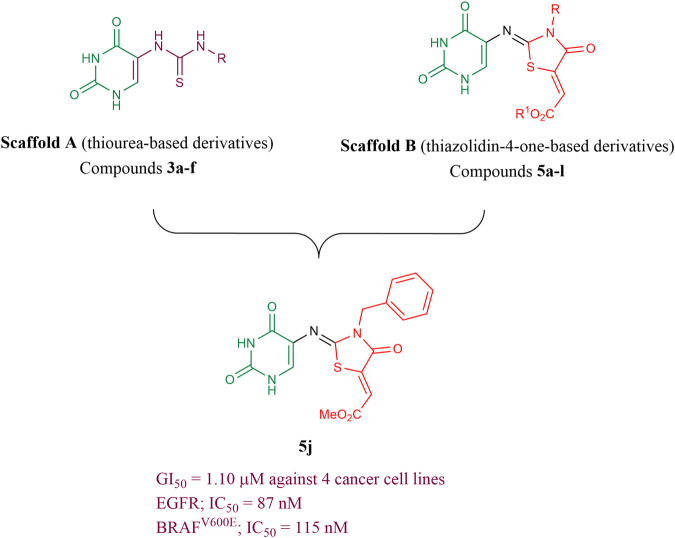

## 1 Introduction

**FIGURE 1:**
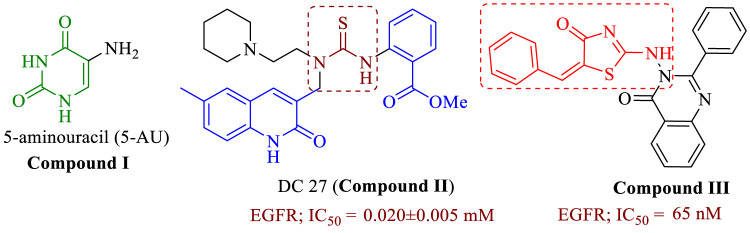
Structure of compounds **I-III**.

Uracil compounds are promising structures in the field of drug discovery (Bouhadir et al., 2016; Putz and Dudas, 2013). Uracils substituted in position five stand out in bioactivity (Gimadieva et al., 2015), with various biological activities including antiviral properties (Palasz and Ciez, 2015), anticancer, cytotoxic (Tanase et al., 2015; Krutikov and Erkin, 2009), antimycobacterial (Isobe et al., 2003; Baraldi et al., 2002), and antitumor (Seferoglu and Ertan, 2008), to antibacterial (Lee et al., 1997). According to Rana and Ganesh (2000), 5-aminouracil (5-AU) (**compound I**, Figure 1) binds to receptors with high affinity by forming hydrogen-bonded triplexes *via* amino and carbonyl groups and a ring nitrogen. Interestingly, 5-aminouracil has antitumor, antibacterial, and antiviral properties (Zielenkiewicz et al., 2000). Furthermore, 5-AU is widely used as a cell cycle inhibitor (Oliev, 1994), as it inhibits the mitotic cycle and the incorporation of guanosine into nucleic acids (Roth and Cheng, 1982). Many thiourea derivatives demonstrate antibacterial, antifungal (Abbas et al., 2013a; El-Sharief et al., 2013), and antiviral (Abbas et al., 2013b) activities. Their ability to inhibit enzymes such as protein tyrosine kinases (PTKs) (Li et al., 2010), topoisomerase II (Huang et al., 2010), human sirtuin type proteins (Napper et al., 2005), and DNA repair synthesis (Ziegler-Skylakakis et al., 1985) may explain their anticancer activity. Some diaryl-thiourea derivatives have been reported to be EGFR inhibitors (Xiong et al., 2008). The thiourea derivative DC27 (**compound II**, Figure 1) was tested for antitumor activity in a panel of human lung carcinoma cell lines (Xiong et al., 2008). The outcomes demonstrated dose-dependent inhibition of cell proliferation, with an IC_50_ of 2.5–12.9 µM, comparable to gefitinib (1.1–15.6 µM). In contrast to gefitinib (IC_50_ = 0.018 ± 007 µM), **DC27** showed potent inhibition of EGFR with a value of 0.020 ± 005 µM. Additionally, a flow cytometry study of **DC27** (Xiong et al., 2008) induced apoptosis and G0/G1 cell cycle arrest.

The thiazolidin-4-one moiety has been proposed as a scaffold for constructing new molecules in medicinal chemistry. Positions 2, 3, and 5 of the thiazolidin-4-one ring, are amenable to modification. When modified with other substituents, thiazolidin-4-one exhibits a wide range of biological activities, including anticancer (Asati et al., 2014; Aly et al., 2020; Sharma et al., 2020). Thiazolidin-4-one hybrids were developed (Aziz et al., 2021), and their anticancer properties were tested on breast cancer (MCF-7) and lung cancer (A549) cell lines. The most effective derivative against the lung cancer (A549) cell line was compound **III** (Figure 1), with an IC_50_ value of 0.72 µM and promising EGFR inhibitory activity at a concentration of 65 nM.

**FIGURE 2:**
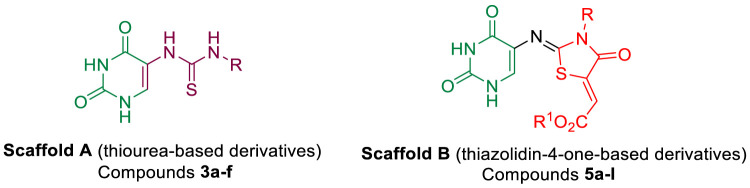
Structures of new hybrids **3a-f** and **5a-l**.

In response to the previous, continuing our efforts to discover novel hybrids as inhibitors of cancer cell growth with dual targeting inhibitory action (Al-Wahaibi et al., 2020; Gomaa et al., 2022), we have now prepared two new series of hybrids (Figure 2): the dihydropyrimidine-2,4-dione/thioureas **3a-f** (**Scaffold A**) and the dihydropyrimidine-2,4-dione/thiazolidin-4-ones **5a-l** (**Scaffold B**). Our goal was to obtain a new antiproliferative agent that can target EGFR and/or BRAF^V600E^. Using an MTT assay, the compounds were tested on a panel of four different cancer cell lines. The EGFR and BRAF enzymatic assays were used to investigate the hybrids’ potential antiproliferative mechanism. A molecular docking study was conducted on the most active compounds within the target active sites of the enzymes.

## 2 Results and discussion

### 2.1 Chemistry

**SCHEME 1:**
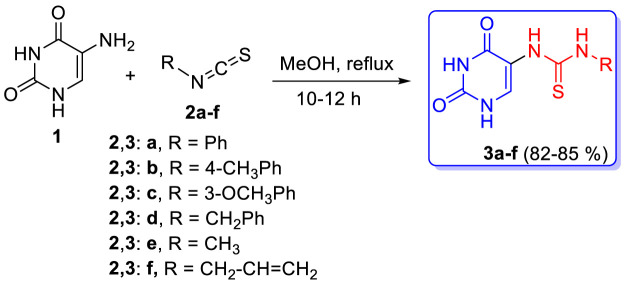
Synthesis of thiourea-based hybrids **3a-f**.

**SCHEME 2:**
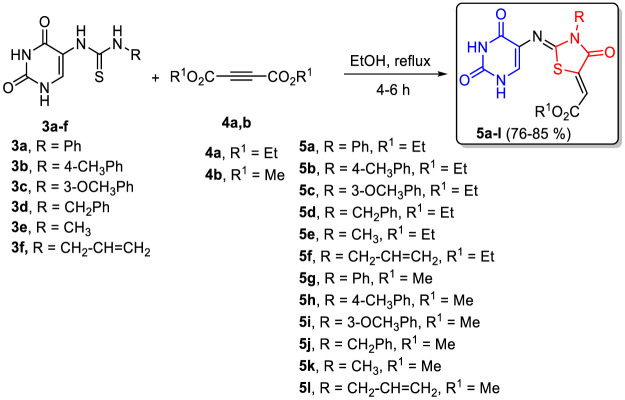
Synthesis of thiazolidin-4-ones **5a-l**.

**FIGURE 3:**
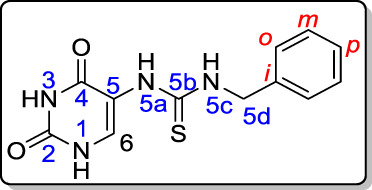
Distinctive carbons and hydrogens for compound **3d**.

The syntheses of the target compounds are depicted in Schemes 1 and 2. The new thioureas **3a-f** (Scaffold A) were synthesized by the reaction of 5-aminouracil (**1**) with isothiocyanate derivatives **2a-f** in boiling methanol for 10–12 h. The structures of the target compounds **3a-f** were confirmed by elemental analyses, IR, NMR (^1^H, ^13^C, 2D NMR, ^15^N), and mass spectroscopy. The ^1^H NMR spectrum of **3d** showed six singlet signals at *δ_H_* 11.33, 10.79, 8.69, 8.45, 7.97 and 4.69 ppm, assigned as NH-3, NH-1, NH-5a, NH-5c, CH-6 and CH_2_-benzyl protons, respectively. The aromatic protons were appeared between *δ_H_* 7.25–7.30 ppm. The ^13^C NMR spectrum showed two carbonyl carbon (C=O) signals at *δ_C_* 161.6 and 150.4 ppm, assigned as C-4 and C-2; there were also signals at *δ_C_* 181.8, 134.8, and 112.7 ppm, assigned as C=S, C-6, and C-5 respectively. A non-protonated carbon at *δ_C_* 138.9 ppm gave HMBC correlation with H-*o,* H-*m,* and H-5d; this carbon is assigned as C-*i.* The four nitrogens all gave HSQC correlation with their attached protons (Table 1; Figure 3).

**TABLE 1:** NMR spectroscopy of compound **3d**.

^1^H NMR (DMSO-*d* *_6_*)	^1^H–^1^H COSY	Assignment
11.33 (bs; 1H)		NH-3
10.79 (bs; 1H)		NH-1
8.69 (bs; 1H)		NH-5a
8.45 (b; 1H)	4.69	NH-5c
7.97 (b; 1H)		H-6
7.30 (m; 4H)		H-*o*, *m*
7.25 (m; 1H)		H-*p*
4.69 (s; 2H)	8.45	H-5d

^13^C NMR (DMSO-*d* *_6_*)	HSQC	HMBC	Assignment
181.8		4.69	C-5b
161.6			C-4
150.4			C-2
138.9		7.30, 4.69	C-*i*
134.8			C-6
128.1	7.30	7.30	C-*m*
127.2	7.30	7.30, 4.69	C-*o*
126.7	7.25	7.30	C-*p*
112.7		11.33	C-5
47.2	4.69	7.30, 4.69	C-5d

^15^N NMR (DMSO-*d* *_6_*)	HSQC	HMBC	Assignment
157.1	11.33		N-3
127.7	10.79		N-1
118.0	8.45		N-5c
107.8	8.69		N-5a

A new series of pyrimidine-bearing thiazolidinones (Scaffold B, **5a-l)** was synthesized by refluxing thioureas **3a-f** with acetylenedicarboxylate derivatives **4a,b** in methanol for 4–6 h in 76–85% yields. The spectral and elemental data revealed that all **5a-l** derivatives underwent the reaction smoothly to give the respective 2,4-dioxo-1,2,3,4-tetrahydro-pyrimidin-5-yl)imino)-4-oxo-3-substituted thiazolidin-5-ylidene)-acetates.

**FIGURE 4:**
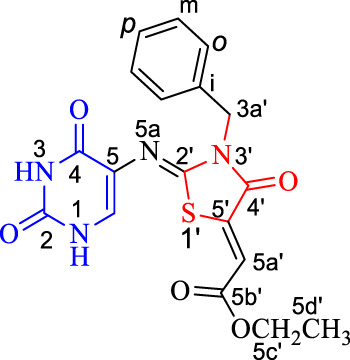
Distinctive carbons and hydrogens for compound **5d**.

The ^1^H NMR spectra showed the disappearance of NH-5a signal of the thiourea in molecule **3**. For example, **5d** formed by the reaction of compound **3d** with diethyl acetylenedicarboxylate (**4a**). The signals of the ethoxy group were distinctive at *δ*_*H*_ 4.23 (H-5c′) and 1.25 (H-5d′), *δ*_*C*_ 61.5 (C-5c′) and 13.9 ppm (C-5d′): H-5c′ gave HMBC correlation with a carbon at *δ*_*C*_ 165.3 ppm, assigned as C-5b’. C-5b′ also gave HMBC correlation with a proton at *δ*_*H*_ 6.77 ppm, assigned as H-5a’; its attached carbon appeared at *δ*_*C*_ 115.2 ppm. H-5a′ also gave HMBC correlation with carbons at *δ*_*C*_ 140.5 and 164.1 ppm; these carbons are assigned as C-5′ and C-4′, respectively on chemical-shift grounds, confirmed by HMBC correlation between C-4′ and the singlet of CH_2_-benzyl at *δ*_*H*_ 5.02, assigned as H-3a’. H-3a′ gave HSQC correlation with its attached carbon at *δ*_*C*_ 45.5; H-3a′ also gave HMBC correlation with a nitrogen at *δ*_*N*_ 160.4, assigned as *N*-3′, and with a carbon at *δ*_*C*_ 153.0, assigned as C-2’. H-6 gave HMBC correlation with an *sp* ^2^ nitrogen at *δ*_*N*_ 244.8 ppm, assigned as *N*-5a, and with carbon at *δ*_*C*_ 159.6. The IR spectrum of **5d** showed strong absorption bands between *ν* = 3150 (NH), 2975 (Ar-CH), 1687 (CO), 1647 (C=N) and 1510 cm^−1^ (C=C). The mass spectrum and elemental analyses of **5d** agreed with the assigned structure (Table 2; Figure 4).

**TABLE 2:** NMR spectroscopy of compound **5d**.

^1^H NMR (DMSO-*d* *_6_*)	^1^H–^1^H COSY	Assignment
11.41 (bs; 1H)		NH-3
10.96 (bs; 1H)	7.30	NH-1
7.40 (d, *J* = 6.9; 2H)	7.33, 5.02	H-*o*
7.33 (dd, *J* = 7.5, 6.8; 2H)	7.40, 7.30	H-*m*
7.30 (m; 2H)	10.96, 7.33	H-*p*
6.77 (s; 1H)		H-5a′
5.02 (s; 2H)	7.40	H-3a′
4.23 (q, *J* = 7.1; 2H)	1.25	H-5c′
1.25 (t, *J* = 7.1; 3H)	4.23	H-5d′

^13^C NMR (DMSO-*d* *_6_*)	HSQC	HMBC	Assignment
165.3		6.77, 4.23	C-5b′
164.1		6.77, 5.02	C-4′
159.6		7.30	C-4
153.0		7.30, 5.02	C-2′
150.4		7.30	C-2
140.5		6.77	C-5′
135.5		7.33, 5.02	C-*i*
131.3	7.30	7.33, 7.30	C-*o*
128.4	7.33	7.40, 7.33, 5.02	C-*m*
127.8	7.40	7.40, 7.33	C-*p*
127.6	7.30		C-6
121.1		11.41	C-5
115.2	6.77	6.77	C-5a′
61.5	4.23	1.25	C-5c′
45.5	5.02	7.40, 5.02	C-3a′
13.9	1.25	4.23, 1.25	C-5d′

^15^N MR (DMSO-*d*_*6*_)	HSQC	HMBC	Assignment
244.8		7.30	N-5a
160.4		5.02	N-3′
157.3	11.41	11.41	N-3
127.6	10.96	7.30	N-1

On reacting compounds **3a-f** with dimethyl acetylene dicarboxylate (**4b**), methyl (*Z*)-2-((*Z*)-2-((2,4-dioxo-1,2,3,4-tetrahydropyrimidin-5-yl)imino)-3-yl-4-oxothiazolidin-5-ylidene) acetates **5g-l** were formed. The structure assignments of **5g**–**I** were delineated from their spectroscopic properties and elemental analyses. The molecular structure of **5j**, for example, was supported as follows: the molecular formula of **5j** (C_17_H_14_N_4_O_5_S) corresponded to one molecule of **3d** and one molecule of dimethyl acetylenedicarboxylate (**4b**) less one molecule of methanol, giving rise to the ion *m*/*z* = 386. The NMR spectra of **5j** closely resembled those of **5d,** with a methyl ester replacing the ethyl ester (Table 3).

**TABLE 3:** NMR spectroscopy of compound **5j**.

^1^H NMR (DMSO-*d* *_6_*)	^1^H–^1^H COSY	Assignment
11.41 (bs; 1H)		NH-3
10.95 (b; 1H)	7.30	NH-1
7.41 (d, *J* = 7.0; 2H)	7.34, 5.03	H-*o*
7.34 (dd, *J* = 7.4, 6.8; 2H)	7.41, 7.29	H-*m*
7.30 (s; 1H)	10.95	H-6
7.29 (t, *J* = 6.5; 1H)	7.34	H-*p*
6.81 (s; 1H)		H-5a′
5.03 (s; 2H)		H-3a′
3.77 (s; 3H)		H-5c′

^13^C NMR (DMSO-*d* *_6_*)	HSQC	HMBC	Assignment
165.7		6.81, 3.77	C-5b′
164.2		6.81, 5.03	C-2′
159.5		11.51, 7.30	C-4
153.0		5.03	C-4′
150.4		7.30	C-2
140.7		6.81	C-5′
135.6		7.34, 5.03	C-*i*
131.4	7.30		C-6
128.5	7.34	7.34	C-*m*
127.9	7.41	7.41, 7.29	C-*o*
127.6	7.29	7.41, 7.29, 5.03	C-*p*
120.7		11.41	C-5
115.4	6.81	6.81	C-5a′
52.6	3.77	3.77	C-5c′
45.6	5.03	7.41, 5.03	C-3a′

^15^N NMR (DMSO-*d* *_6_*)	HSQC	HMBC	Assignment
160.9	11.41	5.03	N-3′
158.0	11.41	11.41	N-3
127.2	10.95	11.41	N-1

**FIGURE 5:**
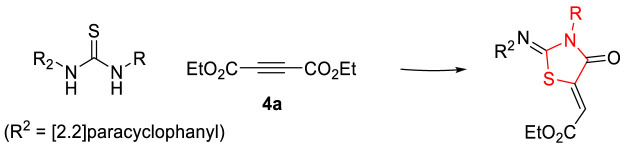
Stereochemistry of **5a-l**.

**FIGURE 6:**
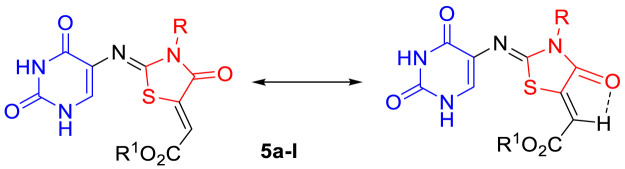
Resonance stabilized compounds **5a-l**.

We recently reacted thioureas bearing a [2.2]paracyclophane moiety with diethyl acetylene-dicarboxylate (**4a**) to form thiazolidinones (Alshammari et al., 2022). An X-ray crystal structure showed the C=N and C=C double bonds to both have (*Z*) stereochemistry (Figure 5); in the current series **5a-l,** we assign the C=N and C=C bonds as both (*Z*) by analogy with our earlier work. Further evidence was based upon the suggestion that there is a resonance stabilized by the hydrogen bond formed *via* the oxygen of the carbonyl group and the exo-cyclic hydrogen as shown in Figure 6.

**SCHEME 3:**
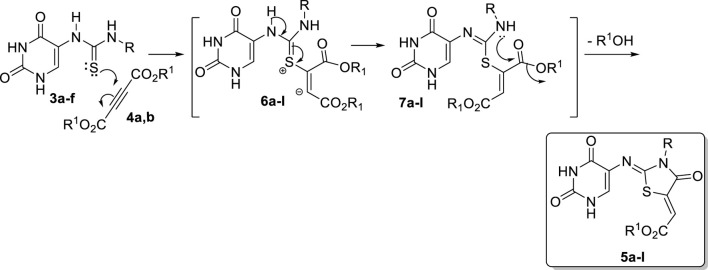
Suggested mechanism for the formation of compounds **5a-l**.

The rationale for forming **5a-l** begins with conjugate attack by the sulfur lone-pair of the thione group in **3a-f** on the triple bond of **4a,b** by the nitrogen lone pairs to generate the zwitterions **6a-l**. Subsequently, proton migration would give intermediates **7a-l** (Scheme 3). Finally, the lone pair of the nitrogen atom in the intermediate **7a-l** would attack the carbonyl group of the same compound, which is accompanied by the elimination of an alcohol molecule to give the final product **5a-l** (Scheme 3).

### 2.2 Biology

#### 2.2.1 Cell viability assay

To test the viability of new compounds, the human mammary gland epithelial (MCF-10A) cell line was used (Youssif et al., 2019; Mahmoud et al., 2022). MCF-10A cells were incubated with compounds **3a-f** and **5a-l** for 4 days before being tested for viability using the MTT assay. Table 4 shows that none of the compounds tested exhibited cytotoxic effects, and cell viability was greater than 86% for the compounds tested at 50 µM.

**TABLE 4:** Antiproliferative activity of compounds **3a-f**, **5a-l**, and Doxorubicin.

	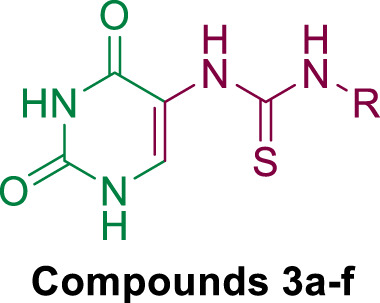	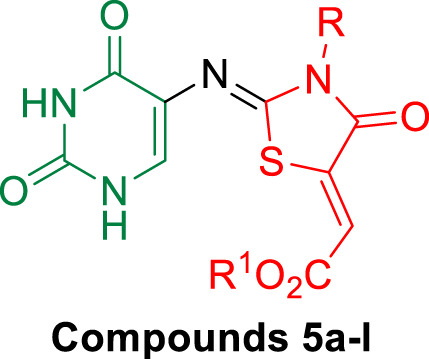				
Compd.	Cell viability %	Antiproliferative activity IC_50_ ± SEM (nM)				
		A-549	MCF-7	Panc-1	HT-29	Average
**3a**	**86**	8.90 ± 0.80	8.50 ± 0.80	8.80 ± 0.80	9.10 ± 0.80	8.80
**3b**	**87**	3.70 ± 0.30	3.60 ± 0.30	4.10 ± 0.30	3.90 ± 0.30	3.80
**3c**	**89**	1.80 ± 0.20	1.40 ± 0.10	2.10 ± 0.20	2.10 ± 0.20	1.85
**3d**	**91**	4.10 ± 0.40	3.90 ± 0.40	4.30 ± 0.40	4.30 ± 0.40	4.15
**3e**	**89**	9.70 ± 0.80	9.60 ± 0.80	9.80 ± 0.80	10.80 ± 0.90	10.00
**3f**	**89**	3.80 ± 0.30	3.70 ± 0.30	3.90 ± 0.30	4.10 ± 0.30	3.90
**5a**	**91**	3.50 ± 0.30	3.10 ± 0.30	3.30 ± 0.30	3.90 ± 0.30	3.45
**5b**	**92**	1.20 ± 0.10	1.10 ± 0.10	1.40 ± 0.10	1.40 ± 0.10	1.30
**5c**	**96**	1.50 ± 0.10	1.60 ± 0.10	1.90 ± 0.20	1.80 ± 0.10	1.70
**5d**	**86**	4.90 ± 0.50	4.70 ± 0.40	5.50 ± 0.50	5.50 ± 0.50	5.15
**5e**	**86**	7.20 ± 0.60	6.70 ± 0.70	7.30 ± 0.70	7.20 ± 0.70	7.10
**5f**	**89**	8.20 ± 0.70	7.90 ± 0.70	8.80 ± 0.70	8.90 ± 0.70	8.50
**5g**	**87**	2.70 ± 0.20	2.20 ± 0.20	2.90 ± 0.20	2.20 ± 0.20	2.50
**5h**	**92**	1.40 ± 0.10	1.70 ± 0.10	1.80 ± 0.10	1.70 ± 0.10	1.65
**5i**	**89**	1.30 ± 0.10	1.00 ± 0.08	1.50 ± 0.10	1.60 ± 0.10	1.35
**5j**	**89**	1.10 ± 0.10	0.90 ± 0.10	1.20 ± 0.10	1.20 ± 0.10	1.10
**5k**	**89**	5.70 ± 0.60	5.10 ± 0.50	5.90 ± 0.50	6.20 ± 0.60	5.70
**5l**	**86**	6.00 ± 0.60	6.50 ± 0.60	6.40 ± 0.60	6.60 ± 0.60	6.40
**Doxorubicin**	-	1.20 ± 0.10	0.90 ± 0.10	1.40 ± 0.10	1.00 ± 0.10	1.10

#### 2.2.2 Antiproliferative assay

Using the MTT assay (Abdelrahman et al., 2017; El-Sherief et al., 2018) and doxorubicin as the reference drug, **3a-f** and **5a-l** were tested for antiproliferative activity against four human cancer cell lines: Panc-1 (pancreas cancer cell line), MCF-7 (breast cancer cell line), HT-29 (colon cancer cell line), and A-549 (epithelial cancer cell line). The median inhibitory concentrations (IC_50_) are shown in Table 4.

The 18 newly synthesized compounds have two main backbones: thiourea-based derivatives, Scaffold A (**3a-f**), and thiazolidin-4-ones, Scaffold B (**5a-l**). The compounds tested showed promising antiproliferative activity, with GI_50_ values ranging from 1.10 µM to 10.00 µM. Compounds **3c**, **5b**, **5c, 5h, 5i,** and **5j** were the most potent derivatives of both backbones, with GI_50_ values ranging from 1.10 µM to 1.80 µM. Compound **5j** (R = benzyl, R^1^ = Me; thiazolidin-4-one backbone) demonstrated the most potent activity, with a GI_50_ value of 1.10 µM, comparable to the reference doxorubicin (GI_50_ = 1.10 µM) and even more potent than doxorubicin against A-549 and Panc-1 cancer cell lines, as shown in Table 4. Compound **5d** (R = benzyl, R^1^ = Et; thiazolidin-4-one backbone) showed a GI_50_ of 5.15 µM, which is approximately 5-fold less potent than compound **5j**, indicating the importance of methyl ester for antiproliferative action, which is more tolerated than ethyl ester. Furthermore, compound **3d** (R = benzyl; thiourea-based backbone) showed moderate antiproliferative activity with a GI_50_ value of 4.15 µM, four times less active than compound **5j** of thiazolidin-4-one backbone, indicating that thiazolidin-4-one backbone is more tolerated for antiproliferative action than thiourea one.

Compound **5b** (R = *p*-CH_3_-Ph, R^1^ = Et; thiazolidin-4-one backbone) ranks second in activity, with a GI_50_ of 1.30 µM, which is 1.2-fold less potent than compounds **5j** and doxorubicin. Compound **5b** had the same potency as doxorubicin against both A-549 and Panc-1 cancer cell lines, with IC_50_ values of 1.20 µM and 1.40 µM, respectively. **5b** was found to be more potent than its methyl ester derivative, compound **5h** (R = *p*-CH_3_-Ph, R^1^ = Me; thiazolidin-4-one backbone), which had a GI_50_ value of 1.65 µM (Table 1).

Once again, the methyl ester derivative compound **5i** (R = *m*-OCH_3_-Ph, R^1^ = Me; thiazolidin-4-one backbone) outperformed the ethyl ester derivative **5c** (R = *m*-OCH_3_-Ph, R^1^ = Et; thiazolidin-4-one backbone) with GI_50_ values of 1.35 µM and 1.70 µM against the four cancer cell lines, respectively. Moreover, the antiproliferative activity of compounds **5i** and **5c** (thiazolidin-4-one-based derivatives; R = *m*-OMe-Ph) was comparable to that of compound **5b** (thiazolidin-4-one-based derivatives; R = *p*-Me-Ph), indicating that both *m*-OMe-Ph and *p*-Me-Ph groups are well tolerated. Compound **3c** (R = *m*-OCH_3_-Ph; thiourea-based backbone) was the only thiourea-based derivative with a GI_50_ less than 2 µM (GI_50_ = 1.80 µM), confirming that thiazolidin-4-one based derivatives have higher inhibitory activity against the tested cancer cell line than thiourea-based derivatives.

#### 2.2.3 EGFR inhibitory assay

**FIGURE 7:**
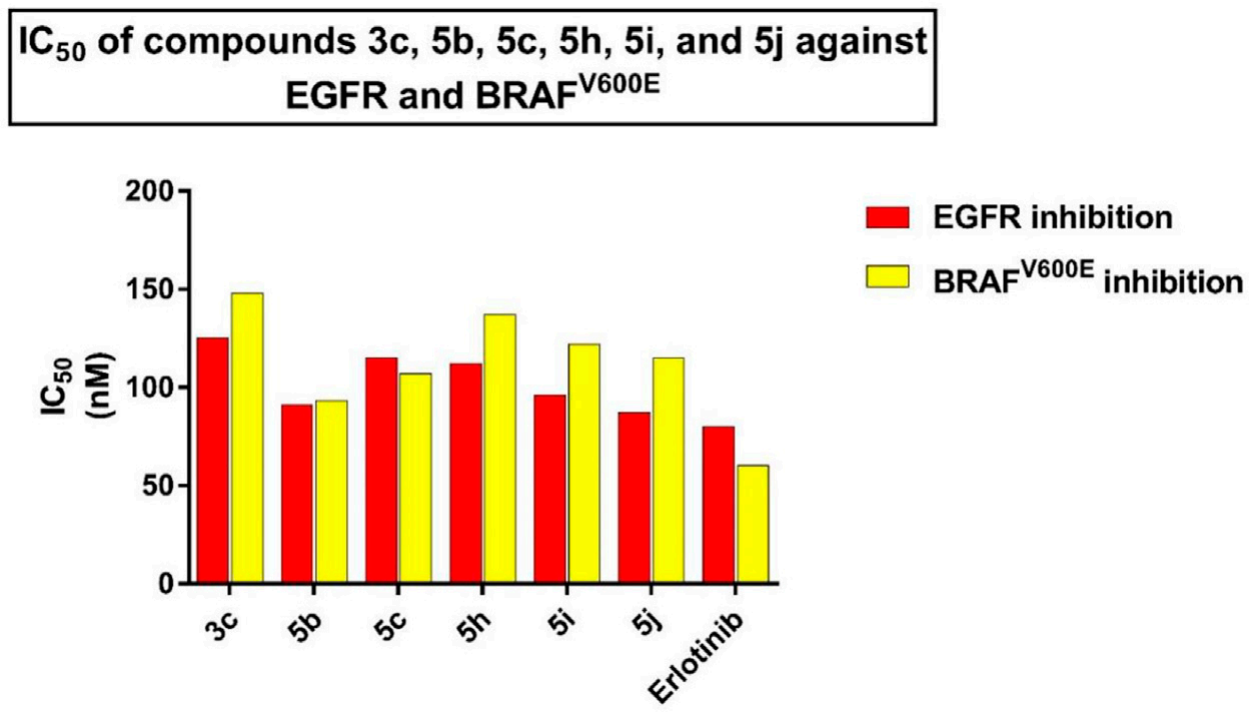
IC_50_ of compounds **3c**, **5b**, **5c**, **5h**, **5i**, and **5j** against EGFR and BRAF^V600E^.

The six most potent antiproliferative derivatives (**3c, 5b, 5c, 5h, 5i,** and **5j**) were further tested for their inhibitory action against EGFR (Mohamed et al., 2021) as a potential target for their antiproliferative activity. Table 5; Figure 7 shows the results as IC_50_ values.

**TABLE 5:** IC_50_ of compounds **3c**, **5b**, **5c**, **5h**, **5i**, and **5j** against EGFR and BRAF^V600E^.

Compd.	EGFR inhibition IC_50_ ± SEM (nM)	BRAF^V600E^ inhibition IC_50_ ± SEM (nM)
**3c**	125 ± 11	148 ± 12
**5b**	91 ± 07	93 ± 08
**5c**	115 ± 10	107 ± 10
**5h**	112 ± 10	137 ± 12
**5i**	96 ± 07	122 ± 12
**5j**	87 ± 05	115 ± 12
**Erlotinib**	80 ± 05	60 ± 05

The findings of this test are consistent with the findings of the antiproliferative assay, in which compound **5j** (R = benzyl, R^1^ = Me; thiazolidin-4-one backbone), the most potent antiproliferative, demonstrated the highest inhibitory activity against EGFR with an IC_50_ value of 87 ± 05 nM, which is very close to that of the reference erlotinib (IC_50_ = 80 ± 05 nM). Compounds **5b** (R = *p*-CH_3_-Ph, R^1^ = Et; thiazolidin-4-one backbone) and **5i** (R = *m*-OCH_3_-Ph, R^1^ = Me; thiazolidin-4-one backbone) rank second and third in activity with IC_50_ values of 91 ± 07 nM and 97 ± 07 nM, respectively. Compound **3c** (R = *m*-OCH_3_-Ph; thiourea-based backbone) was the least potent of the six compounds tested, with an IC_50_ value of 125 ± 11 nM, making it 1.6-fold less potent than erlotinib. Based on the results of this assay, compounds **5b**, **5i**, and **5j** showed promising antiproliferative activity and have the potential to act as EGFR inhibitors.

#### 2.2.4 BRAF^V600E^ inhibitory assay

Compounds **3c, 5b, 5c, 5h, 5i,** and **5j** were further tested for their inhibitory action against mutant BRAF (Mohassab, A. M., et al., 2021), and results were cited in Table 5 and Figure 7 as IC_50_ values. The tested compounds showed moderate inhibitory activity against the tested mutant BRAF, with IC_50_ values ranging from 93 nM to 148 nM and were all less potent than the reference erlotinib (IC_50_ = 60 ± 05 nM). Compound **5b** (R = *p*-CH_3_-Ph, R^1^ = Et; thiazolidin-4-one backbone) was the most potent derivative as BRAF^V600E^ inhibitor with IC_50_ of 93 ± 08 nM indicating that this compound could serve as a dual inhibitor of EGFR and BRAF^V600E^ with promising antiproliferative properties.

### 2.3 Molecular docking

AutoDock4.2.6 software was utilized to carry out all docking computations (Morris et al., 2009). The crystal structures of EGFR and BRAF^V600E^ with PDB accession codes: 1M17 (Stamos et al., 2002) and 3OG7 (Bollag et al., 2010), respectively, were obtained and utilized as templates for all docking computations. The pdbqt file of both EGFR and BRAF^V600E^ was prepared as described by Ibrahim et al. (2022a) and Ibrahim et al. (2022b). The Lamarckian genetic algorithm (LGA) opted for inhibitor conformational searching and docking parameters involving 25,000,000 energy evaluations and 250 genetic algorithm runs. The rest parameters were kept as default. A grid box with 50 Å × 50 Å × 50 Å in the *x*, *y*, and *z* directions were utilized to include the binding pocket of EGFR and BRAF^V600E^. The grid maps with a spacing of 0.375 Å were generated utilizing the AutoGrid program (Goodford, 1985). The grid was positioned at the center of the active sites of EGFR and BRAF^V600E^. The molecular interactions were depicted using the BIOVIA Discovery Studio Visualizer 2020 (Dassault Systèmes, 2019).

**FIGURE 8:**
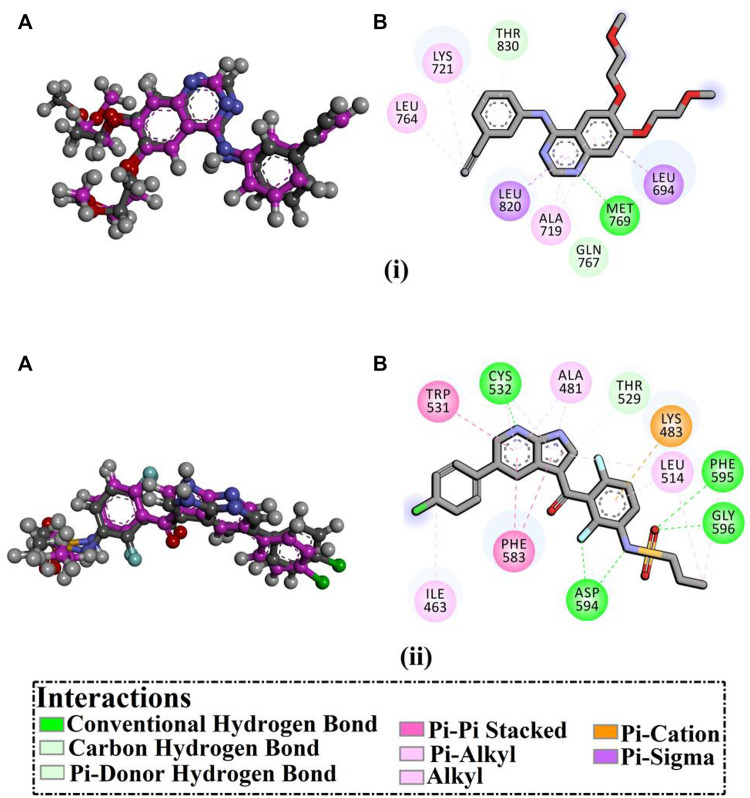
**(A)** 3D molecular interactions of the predicted binding modes (in purple) and the native structures (in gray) and **(B)** 2D representations of the portended binding modes of (i) 4-anilinoquinazoline with EGFR and (ii) PLX4032 with BRAF^V600E^.

To reveal the binding modes of the synthesized compounds with the active site of the EGFR and BRAF^V600E^, docking computations were performed. Validation of the AutoDock4.2.6 software with the employed parameters was initially executed according to the accessible experimental data. The co-crystallized inhibitors–namely 4-anilinoquinazoline and PLX4032—with the EGFR and BRAF^V600E^ were redocked and compared to the native structures (PDB ID; 1M17 and 3OG7, respectively) (Figure 8). As shown in Figure 8, the predicted docking poses were approximately identical to the co-crystallized structures, having 0.20 and 0.18 Å RMSD compared to the co-crystallized conformations of 4-anilinoquinazoline and PLX4032, respectively (Figure 8). In essence, the utilized docking protocol could be applied to foretell the correct binding mode of ligands with the targeted receptors.

**FIGURE 9:**
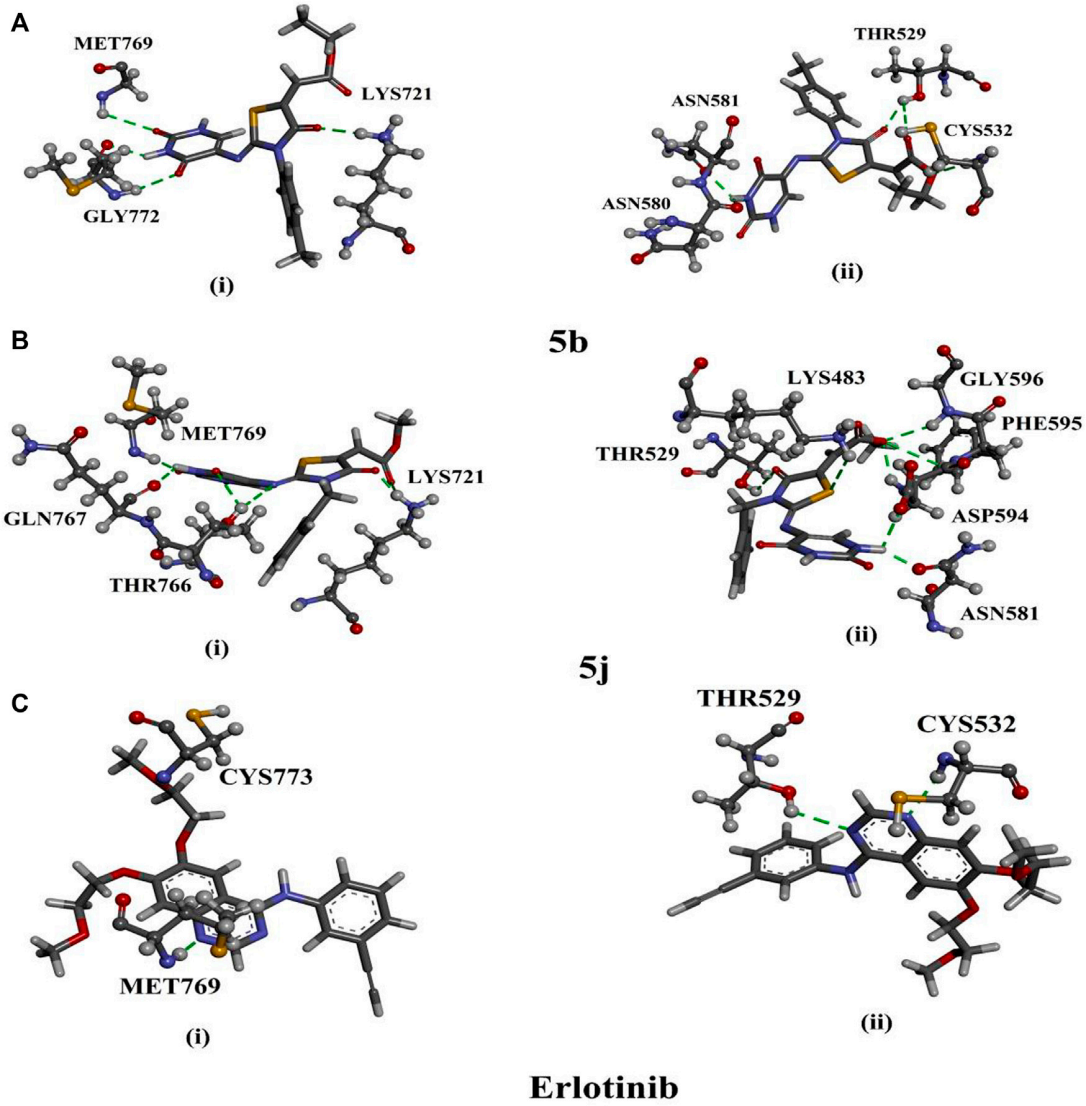
3D representations of interactions of compounds **(A) 5b**, **(B) 5j**, and **(C)** erlotinib with key residues of (i) EGFR and (ii) BRAF^V600E^.

Based on the validated performance of AutoDock4.2.6 software, it was utilized to predict the docking scores and binding features of the synthesized compounds against EGFR and BRAF^V600E^. The anticipated binding features and docking scores are compiled in Supplementary Table S1. Based on the data enrolled in Supplementary Table S1, all investigated molecules demonstrated superior docking scores against EGFR and BRAF^V600E^, with values ranging from −8.0 to −8.3 and from −9.1 to −9.7 kcal/mol, respectively. The special binding affinities against EGFR and BRAF^V600E^ may be attributed to their capability of exhibiting a diversity of H-bonds, π-based, hydrophobic, and vdW interactions with the most important amino acids inside the binding pockets of EGFR and BRAF^V600E^. Comparing the docking results demonstrated that compounds **5b** and **5j** unveiled promising docking scores against EGFR and BRAF^V600E^ with values of −8.3 and −9.7 kcal/mol and −8.2 and −9.3 kcal/mol, respectively. More exactly, compound **5b** exhibited four and five hydrogen bonds with LYS721 (2.14 Å), MET769 (2.09, 2.43 Å), and GLY772 (2.83 Å) and THR529 (2.24, 3.03 Å), CYS532 (2.13 Å), ASN580 (2.65 Å), and ASN581 (2.12 Å) within the binding pockets of EGFR and BRAF^V600E^, respectively (Supplementary Table S1; Figure 9).

Compound **5j** formed six and seven hydrogen bonds with LYS721 (1.63, 2.96 Å), THR766 (2.44, 2.84 Å), GLN767 (2.29 Å), and MET769 (1.82 Å) and LYS483 (1.86 Å), THR529 (2.52 Å), ASN581 (1.97 Å), ASP594 (2.08, 2.20 Å), PHE595 (2.95 Å), and GLY596 (2.39 Å) inside the binding sites of EGFR and BRAF^V600E^, respectively (Supplementary Table S1; Figure 9).

Erlotinib, a positive control, manifested good docking scores towards EGFR and BRAF^V600E^ with values of −8.6 and −8.4 kcal/mol, respectively (Supplementary Table S1). As illuminated in Figure 9, erlotinib demonstrated two hydrogen bonds with MET769 (1.62 Å) and CYS773 (1.91 Å) within the active site of EGFR and two hydrogen bonds with THR529 (2.07 Å) and CYS532 (2.02 Å) within the active site of BRAF^V600E^ (Supplementary Table S1). A docking comparison of erlotinib with compounds **5b** and **5j** exposed competing docking scores proposing the *in-silico* perspective of the three molecules as EGFR and BRAF^V600E^ inhibitors.

## 3 Experimental

Instrumentation: See Supplementary Appendix SA.

### 3.1 General procedure for the synthesis of compounds 3a-f

Compounds **3a-f** were synthesized by refluxing of 5-aminouracil (**1**, 10 mmol) and different isothiocyanates **2a-f** (1.2 mmol) in methanol (50 ml) and the presence of a few drops of triethylamine (0.5 ml) as a catalyst for 10–12 h. The resulting solid was filtered and recrystallized from DMF.

#### 3.1.1 1-(2,4-Dioxo-1,2,3,4-tetrahydropyrimidin-5-yl)-3-phenylthiourea (3a)

Yield: 84%; mp 340–342°C, IR (KBr): υ_max_/cm^−1^ = 3165 (NH), 2991 (Ar-CH), 1735 (CO), 1669 (CO), 1574 (C=C), 1330, 1208 (C=S). ^1^H NMR (DMSO-*d_6_*): *δ_H_* = 7.15 (t, 1H, *J* = 7.4 Hz, Ar-H), 7.34 (dd, 2H, *J* = 8.1, 7.6 Hz, Ar-H), 7.48 (d, 2H, *J* = 7.8 Hz, Ar-H), 11.41 (bs, 1H, ^3^NH), 8.16 (bs, 1H, CH-6), 8.87 (s, 1H, ^5a^NH), 9.98 (bs, 1H, ^5c^NH), 10.82 ppm (bs, 1H, ^1^NH), ^13^C NMR (DMSO-*d_6_*): *δ_C_* = 113.2 (C-5), 123.9, 124.7, 128.4 (CH-Ar), 134.9 (C-6), 139.2 (C-Ar), 150.1 (^2^C = O), 161.5 (^4^C = O), 179.7 ppm (C=S). ^15^N NMR (DMSO-*d_6_*): *δ_N_* = 126.9 (N-1), 131.8 (N-5c), 157.0 ppm (N-3). MS: *m/z* = 262 (M^+^, 40), 228 (100), 185 (12), 169 (15), 157 (15), 103 (50), 77 (15). Anal. Calcd for C_11_H_10_N_4_O_2_S (262.29):C, 50.37; H, 3.84; N, 21.36; S, 12.23. Found: C, 50.46; H, 3.87; N, 21.48; S, 12.31.

#### 3.1.2 1-(2,4-Dioxo-1,2,3,4-tetrahydropyrimidin-5-yl)-3-(*p*-tolyl)thiourea (3b)

Yield: 82%; mp 338–340°C, IR (KBr): υ_max_/cm^−1^ = 3165 (NH), 2994 (Ar-CH), 1743 (CO), 1668 (CO), 1575 (C=C), 1330, 1210 (C=S). ^1^H NMR (DMSO-*d_6_*): *δ_H_* = 2.29 (s, 3H, CH_3_), 7.14 (d, 2H, *J* = 8.2 Hz, Ar-H), 8.15 (s, 1H, CH-6), 8.78 (s, 1H, ^5a^NH), 9.88 (bs, 1H, ^5c^NH), 10.80 (bs, 1H, ^1^NH), 11.39 ppm (bs,1H, ^3^NH). ^13^C NMR (DMSO-*d_6_*): *δ_C_* = 20.5 (CH_3_), 113.3 (C-5), 124.1, 128.8 (CH-Ar), 134.0 (C-6), 136.5 (C-Ar), 150.1 (^2^C = O), 161.5 (^4^C = O), 179.7 ppm (C=S). ^15^N NMR (DMSO-*d_6_*): *δ_N_* = 111.9 (N-5a, 5c), 126.8 (N-1), 156.9 ppm (N-3). MS: *m/z* = 276 (M^+^, 10), 242 (100), 169 (16), 149 (25), 127 (27), 117 (52), 91 (28). Anal. Calcd for C_12_H_12_N_4_O_2_S (276.31): C, 52.16; H, 4.38; N, 20.28; S, 11.60. Found: C, 52.27; H, 4.41; N, 20.36; S, 11.71.

#### 3.1.3 1-(2,4-Dioxo-1,2,3,4-tetrahydropyrimidin-5-yl)-3-(3-methoxyphenyl)thiourea (3c)

Yield: 84%; mp = 350–352°C, IR (KBr) υ_max_/cm^−1^ = 3164 (NH), 2996 (Ar-CH), 1741 (CO), 1668 (CO), 1574 (C=C), 1330, 1208 (C=S). ^1^H NMR (DMSO-*d_6_*): *δ_H_* = 3.75 (s, 3H, OCH_3_), 6.73 (dd, 1H, *J* = 8.1, 2.0 Hz, Ar-H), 7.02 (bd, 1H, *J* = 7.9 Hz, Ar-H), 7.22 (bs, 1H, Ar-H), 7.24 (t, 1H, *J* = 8.2 Hz, Ar-H), 8.16 (b, 1H, H-6), 8.87 (bs, 1H,^5c^NH), 10.00 (b, 1H, ^5a^NH), 10.81 (bs, 1H, ^1^NH), 11.40 ppm (bs, 1H, ^3^NH). ^13^C NMR (DMSO-*d_6_*): *δ_C_* = 55.1 (OCH_3_), 109.3, 110.2 (CH-Ar), 113.3 (C-5), 115.7, 129.2 (CH-Ar), 140.3 (C-Ar), 134.9 (C-6), 150.1 (^2^C = O), 161.5 (^4^C = O), 179.4 ppm (C=S). MS: *m/z* = 292 (M^+^, 28), 264 (100), 233 (30), 157 (28), 143 (14), 84 (44), 77 (35). ^15^N NMR (DMSO-*d_6_*): *δ_N_* = 113.5 (N-5c), 126.7 (N-1), 156.9 ppm (N-3). Anal. Calcd for C_12_H_12_N_4_O_3_S (292.31): C, 49.31; H, 4.14; N, 19.17; S, 10.97. Found: C, 49.45; H, 4.17; N, 19.32; S, 10.86.

#### 3.1.4 1-Benzyl-3-(2,4-dioxo-1,2,3,4-tetrahydropyrimidin-5-yl)thiourea (3d)

Yield: 83%; mp = 350–352 (decomp) °C, IR (KBr) υ_max_/cm^−1^ = 3162 (NH), 2998 (Ar-CH), 1743 (CO), 1668 (CO), 1575 (C=C), 1330, 1210 (C=S). NMR (DMSO-*d_6_*): See Table 1; Figure 3. MS: *m/z* = 276 (M^+^, 24), 244 (100), 171 (20), 157 (35), 143 (20), 98 (41), 91 (48). Anal. Calcd for C_12_H_12_N_4_O_2_S (276.31): C, 52.16; H, 4.38; N, 20.28; S, 11.60. Found: C, 52.27; H, 4.41; N, 20.40; S, 11.72.

#### 3.1.5 1-(2,4-Dioxo-1,2,3,4-tetrahydropyrimidin-5-yl)-3-methylthiourea (3e)

Yield: 85%; mp = 332–334°C, IR (KBr) υ_max_/cm^−1^ = 3127 (NH), 3086 (Ar-CH), 1689 (CO), 1654 (CO), 1553 (C=C), 1335, 1207 (C=S). ^1^H NMR (DMSO-*d_6_*): *δ_H_* = 2.85 (d, 3H, *J* = 4.2 Hz, CH_3_), 7.89 (bs, 2H,^5a^NH, CH-6), 8.53 (bs, 1H, ^5c^NH), 10.77 ppm (bs, 1H, ^1^NH), 11.29 ppm (bs, 1H, ^3^NH). ^13^C NMR (DMSO-*d_6_*): *δ_C_* = 31.1 (CH_3_), 112.7 (C-5), 136.3 (C-6), 150.4 (^2^C = O), 161.7 (^4^C = O), 182.1 ppm (C=S). ^15^N NMR (DMSO-*d_6_*): *δ_N_* = 104.6 (N-5a), 106.1 (N-5c), 127.3 (N-1), 157.0 ppm (N-3). MS: *m/z* = 200 (M^+^, 24), 127 (100), 56 (52). Anal. Calcd for C_6_H_8_N_4_O_2_S (200.22): C, 35.99; H, 4.03; N, 27.98; S, 16.02. Found: C, 35.89; H, 4.07; N, 28.05; S, 16.12.

#### 3.1.6 1-Allyl-3-(2,4-dioxo-1,2,3,4-tetrahydropyrimidin-5-yl)thiourea (3f)

Yield: 83%; mp = 348–350 °C (decomp), IR (KBr) υ_max_/cm^−1^ = 3209 (NH), 3011 (Ar-CH), 1738 (CO), 1667 (CO), 1574 (C=C), 1328, 1205 (C=S). ^1^H NMR (DMSO-*d_6_*): *δ_H_* = 4.08 (bs, 2H, N-CH_2_), 5.07 (d, 1H, *J* = 10.2, H-5f), 5.17 (dd, 1H, *J* = 17.2, 1.1 Hz, H-5f), 5.83 (ddt, 1H, *J*_*d*_ = 17.2, 10.4 Hz, *J*_*t*_ = 5.2 Hz, H-5e), 7.98 (b, 1H, H-6), 8.14 (b, 1H; NH-5a), 8.62 (bs, 1H, NH-5c), 10.76 (b, 1H, NH-1), 11.31 ppm (bs, 1H, NH-3). ^13^C NMR (DMSO-*d_6_*): *δ_C_* = 46.2 (N-CH_2_), 112.9 (C-5), 115.5 (C-5f), 134.7 (C-5e, C-6), 150.3 (^2^C = O), 161.6 (^4^C = O), 182.4 ppm (C=S). MS: *m/z* = 226 (M^+^, 100), 127 (100), 98 (55), 84 (15), 56 (44). ^15^N NMR (DMSO-*d_6_*): *δ_N_* = 107.0 (N-5a), 114.9 (N-5c), 157.2 ppm (N-3), Anal. Calcd for C_8_H_10_N_4_O_2_S (226.26): C, 42.47; H, 4.45; N, 24.76; S, 14.17. Found: C, 42.56; H, 4.48; N, 24.88; S, 14.25.

### 3.2 General procedure for the synthesis of compounds 5a-l

A solution of **3a-f** (1 mmol) in methanol (20 ml) was added to a 100 ml round bottom flask containing **4a** or **4b** (1.2 mmol) in methanol (10 ml), with refluxing for 4–6 h. After cooling, the yellow precipitate was filtered off, washed with methanol, and recrystallized from a suitable solvent to give pure crystals of **5a-l**.

#### 3.2.1 (*Z*)-Ethyl-2-((*Z*)-2-((2,4-dioxo-1,2,3,4-tetrahydropyrimidin-5-yl)imino)-4-oxo-3-phenyl-thiazolidin-5-ylidene)acetate (5a)

Yield: 76%; mp 320–322°C, IR (KBr) υ_max_/cm^−1^ = 3147 (NH), 2979 (Ar-CH), 1686 (CO), 1643 (C=N), 1510 (C=C). ^1^H NMR (DMSO-*d_6_*): *δ_H_* = 1.24 (t, 3H, *J* = 6.9 Hz, CH_3_), 4.20 (q, 2H, *J* = 7.0 Hz, CH_2_), 6.86 (s, 1H, CH-5a′), 6.92–6.94 (d, 2H, *J* = 7.8 Hz, Ar-H),7.20–7.23 (t, 2H, *J* = 7.8 Hz, Ar-H), 7.45–7.47 (d, 2H, *J* = 7.8 Hz, Ar-H), 8.00 (s, 1H, CH-6), 11.45 (bs, 1H, ^1^NH), 11.68 ppm (bs, 1H, ^3^NH). ^13^C NMR (DMSO-*d_6_*): *δ_C_* = 13.9 (CH_3_), 61.6 (OCH_2_), 116.5 (C-5′a), 120.7 (C-5), 125.2, 128.2, 129.0, 129.5 (CH-Ar), 134.2 (C-Ar), 140.1 (C-6), 143.7 (C-5′), 147.0 (2C = O), 150.6 (C-2′), 159.8 (C-4), 163.5 (C-4′), 165.2 ppm (C-5′b). MS: *m/z* = 388 (M+2, 24), 387 (M+1, 100), 386 (M^+^, 32), 373 (10), 289 (7), 229 (15), 172 (80), 136 (65). Anal. Calcd for C_18_H_20_N_8_O_7_S_2_ (524.53): C, 41.22; H, 3.84; N, 21.36; S, 12.23. Found: C, 41.37; H, 3.87; N, 21.43; S, 12.30.

#### 3.2.2 (*Z*)-Ethyl-2-((*Z*)-2-((2,4-dioxo-1,2,3,4-tetrahydropyrimidin-5-yl)imino)-4-oxo-3-(*p*-tolyl)thiazolidin-5-ylidene)acetate (5b)

Yield: 78%; mp 348–350°C, IR (KBr) υ_max_/cm^−1^ = 3148 (NH), 2979 (Ar-CH), 1686 (CO), 1644 (C=N), 1505 (C=C). ^1^H NMR (DMSO-*d_6_*): *δ_H_* = 1.24 (t, 3H, *J* = 7.1 Hz, CH_3_), 2.32 (s, 3H, CH_3_), 3.28 (s, 3H, NCH_3_), 4.22 (q, 2H, *J* = 7.1 Hz, CH_2_), 6.58 (d, 1H, *J* = 5.2, 0.6 Hz, H-6), 6.83 (d, 2H, *J* = 8.3 Hz, Ar-H),6.85 (s, 1H, H-5′a), 7.22 (d, 2H, *J* = 8.00 Hz, Ar-H), 11.05 (bs, 1H, ^1^NH), 11.42 ppm (bs, 1H, ^3^NH). ^13^C NMR (DMSO-*d_6_*): *δ_C_* = 13.9 (C-5d′), 20.5 (CH_3_), 61.6 (C-5c′), 108.0 (C-5), 116.4 (C-5a′), 116.4 (C-6), 120.6 (C-*o*), 129.9 (C-*m*), 134.4 (C-*p*), 143.8 (C-*i*), 144.5 (C-2′), 150.6 (C-2), 163.5 (C-4), 161.6 (C-4′), 165.2 ppm (C-5b′). ^15^N NMR (DMSO-*d_6_*): *δ_N_* = 119.2 (N-1), 155.8 ppm (N-3). MS: *m/z* = 402 (M+2, 20), 401 (M+1, 85), 400 (M^+^, 30), 373 (18), 301 (10), 243 (10), 154 (25), 149 (48), 136 (22), 107 (14), 91 (14). Anal. Calcd for C_18_H_16_N_4_O_5_S (400.41): C, 53.99; H, 4.03; N, 13.99; S, 8.01. Found: C, 53.90; H, 4.06; N, 14.07; S, 8.11.

#### 3.2.3 (*Z*)-Ethyl-2-((*Z*)-2-((2,4-dioxo-1,2,3,4-tetrahydropyrimidin-5-yl)imino)-3-(3-methoxyphenyl)-4-oxo-thiazolidin-5-ylidene)acetate (5c)

Yield: 75%; mp 330–332°C, IR (KBr) υ_max_/cm^−1^ = 3142 (NH), 2950 (Ar-CH), 1691 (CO), 1648 (C=N), 1598 (C=C). ^1^H NMR (DMSO-*d_6_*): *δ_H_* = 1.24 (t, 3H, *J* = 7.1 Hz, H-5d′), 3.76 (s, 3H, OCH_3_), 4.22 (q, 2H, *J* = 7.1 Hz, H-5c′), 6.46 (d, 1H, *J* = 1.9 Hz, H-2″), 6.51 (d, 1H, *J* = 8.6 Hz, H-6″), 6.79 (dd, 1H, *J* = 8.2 Hz, 1.7 Hz, H-4″), 6.86 (s, 1H, H-5a′), 7.32 (dd, 1H, *J* = 8.1, 8.0 Hz, H-5″), 7.99 (s, 1H, H-5), 11.45 (b, 1H, NH-1), 11.67 ppm (bs, 1H, NH-3). ^13^C NMR (DMSO-*d_6_*): *δ_C_* = 13.9 (C-5d′), 55.2 (C-3a''), 61.6 (C-5c′), 106.3 (C-2″), 108.0 (C-5), 111.0 (C-4″), 112.7 (C-6″), 116.5 (C-5a''), 130.3 (C-5″), 140.2 (C-5′), 143.7 (C-6), 148.3 (C-1″), 150.6 (C-2′), 150.9 (C-1), 159.8 (C-3″), 160.1 (3″), 163.5 (C-4′), 165.2 ppm (C-5b′). ^15^N NMR (DMSO-*d_6_*): *δ_N_* = 133.1 (N-1), 158.3 ppm (N-3). MS: *m/z* = 418 (M+2, 20), 417 (M+1, 84), 416 (M^+^, 32), 372 (10), 289 (15), 259 (14), 195 (10), 154 (100), 137 (66), 107 (22). Anal. Calcd for C_18_H_16_N_4_O_6_S (416.41): C, 51.92; H, 3.87; N, 13.45; S, 7.70. Found: C, 51.98; H, 3.85; N, 13.56; S, 7.78.

#### 3.2.4 (*Z*)-Ethyl-2-((*Z*)-3-benzyl-2-((2,4-dioxo-1,2,3,4-tetrahydropyrimidin-5-yl)imino)-4-oxo-thiazolidin-5-ylidene)acetate (5d)

Yield: 85%; mp 314–316°C, IR (KBr) υ_max_/cm^−1^ = 3150 (NH), 2975 (Ar-CH), 1687 (CO), 1647 (C=N), 1510 (C=C). NMR (DMSO-*d_6_*): See Table 2; Figure 4. MS: *m/z* = 402 (M+2, 25), 401 (M+1, 100), 400 (M^+^, 25), 341 (8), 313 (9), 289 (10), 91 (30). Anal. Calcd for C_18_H_16_N_4_O_5_S (400.41): C, 53.99; H, 4.03; N, 13.99; S, 8.01. Found: C, 53.90; H, 4.06; N, 14.07; S, 8.11.

#### 3.2.5 (*Z*)-Ethyl-2-((*Z*)-2-((2,4-dioxo-1,2,3,4-tetrahydropyrimidin-5-yl)imino)-3-methyl-4-oxo-thiazolidin-5-ylidene)acetate (5e)

Yield: 84%; mp 302–304°C, IR (KBr) υ_max_/cm^−1^ = 3149 (NH), 3062 (Ar-CH), 1716 (CO), 1674 (CO), 1647 (C=N), 1520 (C=C). ^1^H NMR (DMSO-*d_6_*): *δ_H_* = 1.25 (t, 3H, *J* = 6.9 Hz, CH_3_), 3.28 (s, 3H, NCH_3_), 4.23 (q, 2H, *J* = 7.0 Hz, CH_2_), 6.77 (s, 1H, CH-5′a), 7.25 (s, 1H, CH-6), 10.92 (bs, 1H, ^1^NH), 11.41 ppm (bs, 1H, ^3^NH). ^13^C NMR (DMSO-*d_6_*): *δ_C_* = 13.9 (CH_3_), 29.2 (NCH_3_), 61.5 (OCH_2_), 115.2 (C-5′a), 121.1 (C-5), 131.0 (C-6), 141.0 (C-5′), 150.4 (2C = O), 153.9 (C-2′), 159.6 (C-4), 164.4 (C-4′), 165.3 ppm (C-5b′). ^15^N NMR (DMSO-*d_6_*): *δ_N_* = 127.0 (N-1), 149.0 (N-3′), 158.2 (N-3), 244.8 ppm (N-5a). MS: *m/z* = 326 (M+2, 5), 325 (M+1, 30), 324 (M^+^, 20), 289 (10), 279 (5), 242 (5), 195 (10). Anal. Calcd for C_12_H_12_N_4_O_5_S (324.31): C, 44.44; H, 3.73; N, 17.28; S, 9.89. Found: C, 44.56; H, 3.80; N, 17.40; S, 9.98.

#### 3.2.6 (*Z*)-Ethyl-2-((*Z*)-3-allyl-2-((2,4-dioxo-1,2,3,4-tetrahydropyrimidin-5-yl)imino)-4-oxo-thiazolidin-5-ylidene)acetate (5f)

Yield: 76%; mp 310–312°C, IR (KBr) υ_max_/cm^−1^ = 3148 (NH), 2989 (Ar-CH), 1714 (CO), 1675 (CO), 1643 (C=N), 1510 (C=C). ^1^H NMR (DMSO-*d_6_*): *δ_H_* = 1.25 (t, 3H, *J* = 7.1 Hz, CH_3_), 4.23 (q, 2H, *J* = 7.1 Hz, OCH_2_), 4.44 (d, 2H, *J* = 5.2 Hz, NCH_2_), 5.19 (dd, 1H, *J* = 10.3 Hz, 1.0 Hz, 3c′CH), 5.21 (dd, 1H, *J* = 17.2 Hz, 1.1 Hz, CH-3c′), 5.89 (ddt, 1H, *J*_*d*_ = 17.2, 10.4 Hz, *Jt* = 5.2 Hz, CH-3b′), 6.78 (s, 1H, CH-5a′), 7.27 (d, 1H, *J* = 6.6 Hz, CH-6), 10.94 (bd, 1H, *J* = 4.7 Hz, ^1^NH), 11.40 ppm (bs, 1H, ^3^NH). ^13^C NMR (DMSO-*d_6_*): *δ_C_* = 13.9 (CH_3_), 44.4 (NCH_2_), 61.5 (OCH_2_), 115.5 (C-5a′), 117.7 (C-3c′), 120.8 (C-5), 130.9 (C-3b′), 131.3 (C-6), 140.7 (C-5′), 150.4 (C-2), 152.8 (C-2′), 159.5 (C-4), 163.9 (C-4′), 165.2 ppm (C-5b′). ^15^N NMR (DMSO-*d_6_*): *δ_N_* = 127.3 (N-1), 157.2 (N-3′), 158.1 (N-3), 245.0 ppm (N-4a). MS: *m/z* = 352 (M+2, 20), 351 (M+1, 100), 350 (M^+^, 30), 335 (5), 289 (10), 273 (5), 107 (20). Anal. Calcd for C_14_H_14_N_4_O_5_S (350.35): C, 47.99; H, 4.03; N, 15.99; S, 9.15. Found: C, 47.87; H, 4.07; N, 15.89; S, 9.21.

#### 3.2.7 (*Z*)-Methyl-2-((*Z*)-2-((2,4-dioxo-1,2,3,4-tetrahydropyrimidin-5-yl)imino)-4-oxo-3-phenyl-thiazolidin-5-ylidene)acetate (5g)

Yield: 83%; mp 302–304°C, IR (KBr) υ_max_/cm^−1^ = 3307, 2975, 1740, 1647. ^1^H NMR (DMSO-*d_6_*): *δ_H_* = 3.76 (s, 3H, OCH_3_), 6.89 (s, 1H, H-5a′), 6.93 (d, 2H, *J* = 7.4 Hz, Ar-H), 7.22 (t, 1H, *J* = 7.4 Hz, Ar-H), 7.42 (dd, 2H, *J* = 8.0, 7.7 Hz, Ar-H), 8.00 (s, 1H, H-6), 11.45 (b, 1H, ^1^N**H**), 11.67 ppm (b, 1H, ^3^N**H**). ^13^C NMR (DMSO-*d_6_*): *δ_C_* = 52.6 (CH_3_), 108.0 (C-5), 116.3 (C-5a′), 120.7 (C-*o*), 125.2 (C-*p*), 129.5 (C-*m*), 140.2 (C-5′), 143.8 (C-6), 147.0 (C-*i*), 150.6, 150.7 (C-2, 2′), 159.8 (C-4), 163.5 (C-4′), 165.6 ppm (C-5b′). ^15^N NMR (DMSO-*d_6_*): *δ_N_* = 133.5 (N-1), 151.4 ppm (N-3). Anal. Calcd for C_16_H_12_N_4_O_5_S (372.36): C, 51.61; H, 3.25; N, 15.05; S, 8.61. Found: C, 51.72; H, 3.28; N, 15.16; S, 8.73.

#### 3.2.8 (*Z*)-Methyl-2-((*Z*)-2-((2,4-dioxo-1,2,3,4-tetrahydropyrimidin-5-yl)imino)-4-oxo-3-(*p*-tolyl)-thiazolidin-5-ylidene)acetate (5h)

Yield: 78%; mp 308–310°C, IR (KBr) υ_max_/cm^−1^ = 3215 (NH), 3092 (Ar-CH), 1710 (CO), 1679 (CO), 1641 (C=N), 1512 (C=C). ^1^H NMR (DMSO-*d_6_*): *δ_H_* = 2.32 (s, 3H, CH_3_), 3.67 (s, 3H, OCH_3_), 6.87 (s, 1H, H-5a′), 7.13–7.15 (m, 4H, Ar-H), 8.00 (s, 1H; H-6), 11.44 (s, 1H, ^1^NH), 11.66 ppm (b, 1H, ^3^NH). ^13^C NMR (DMSO-*d_6_*): *δ_C_* = 20.45 (CH_3_), 52.7 (OCH_3_), 107.9 (C-5), 116.2 (C-5a′), 120.6, 125.1, 129.9 (CH-Ar), 139.7 (C-5′), 143.8 (C-6), 144.2, 147.0 (C-Ar), 150.5 (C-2), 151.1 (C-2′), 160.0 (C-4), 163.5 (C-4′), 165.6 ppm (C-5b′). MS: *m/z* = 388 (M+2, 35), 387 (M+1, 100), 386 (M^+^, 25), 341 (10), 289 (15), 273 (10), 242 (10), 217 (20), 195 (35), 107 (20). Anal. Calcd for C_17_H_14_N_4_O_5_S (386.38): C, 52.84; H, 3.65; N, 14.50; S, 8.30. Found: C, 52.93; H, 3.69; N, 14.61; S, 8.43.

#### 3.2.9 (*Z*)-Methyl-2-((*Z*)-2-((2,4-dioxo-1,2,3,4-tetrahydropyrimidin-5-yl)imino)-3-(3-methoxyphenyl)-4-oxothiazolidin-5-ylidene)acetate (5i)

Yield: 80%; mp 322–324°C, IR (KBr) υ_max_/cm^−1^ = 3198 (NH), 2998 (Ar-CH), 1711 (CO), 1674 (CO), 1641 (C=N), 1510 (C=C). ^1^H NMR (DMSO-*d_6_*): *δ_H_* = 3.77 (s, 6H, 2OCH_3_), 6.46 (bs, 1H, Ar-H), 6.51 (d, *J* = 7.8 Hz, 1H, Ar-H), 6.79 (d, 1H, *J* = 7.4 Hz, Ar-H), 6.89 (s, 1H, H-5a′), 7.32 (dd, 1H, *J* = 8.0, 8.0 Hz, Ar-H), 8.00 (bd, 1H, H-6), 11.45 (b, 1H, ^1^N**H**), 11.67 ppm (bs, 1H, ^3^N**H**). ^13^C NMR (DMSO-*d_6_*): *δ_C_* = 52.6 (OCH_3_), 55.2 (OCH_3_), 106.3 (CH-Ar), 108.0 (C-5), 111.0 (CH-Ar), 112.7 (CH-Ar), 116.3 (C-5a′), 130.4 (CH-Ar), 140.2 (C-5′), 143.7 (C-6), 148.3 (Ar-C), 150.8 (C-1), 150.6 (C-2′), 160.1 (C-4), 159.8 (Ar-C), 163.5 (C-4′), 165.6 ppm (C-5b′). ^15^N NMR (DMSO-*d_6_*): *δ_N_* = 133.1 (N-1), 158.1 (N-3), 145.5 ppm (N-3′). MS: *m/z* = 404 (M+2, 25), 403 (M+1, 100), 402 (M^+^, 38), 391 (5), 273 (10), 274 (15), 258 (30), 167 (32). Anal. Calcd for C_17_H_14_N_4_O_6_S (402.38): C, 50.74; H, 3.51; N, 13.92; S, 7.97. Found: C, 50.86; H, 3.55; N, 13.85; S, 7.85.

#### 3.2.10 (*Z*)-Methyl-2-((*Z*)-3-benzyl-2-((2,4-dioxo-1,2,3,4-tetrahydropyrimidin-5-yl)imino)-4-oxothiazolidin-5-ylidene)acetate (5j)

Yield: 85%; mp 306–308°C, IR (KBr) υ_max_/cm^−1^ = 3307, 2975, 1740, 1647. NMR (DMSO-*d_6_*): *See* Table 3. MS: *m/z* = 404 (M+2, 25), 403 (M+1, 100), 402 (M^+^, 38), 391 (5), 273 (10), 274 (15), 258 (30), 167 (32). Anal. Calcd for C_17_H_14_N_4_O_5_S (386.38): C, 52.84; H, 3.65; N, 14.50; S, 8.30. Found: C, 52.94; H, 3.68; N, 14.62; S, 8.44.

#### 3.2.11 (*Z*)-Methyl-2-((*Z*)-2-((2,4-dioxo-1,2,3,4-tetrahydropyrimidin-5-yl)imino)-3-methyl-4-oxothiazolidin-5-ylidene)acetate (5k)

Yield: 85%; mp 300–302°C, IR (KBr) υ_max_/cm^−1^ = 3307, 2975, 1740, 1647. ^1^H NMR (DMSO-*d_6_*): *δ_H_* = 3.27 (s, 3H, NCH_3_), 3.77 (s, 3H, OCH_3_), 6.80 (s, 1H, H-5a′), 7.24 (d, 1H, *J* = 5.1 Hz, H-6), 10.92 (bs, 1H, ^1^N**H**), 11.40 ppm (bs, 1H, ^3^N**H**). ^13^C NMR (DMSO-*d_6_*): *δ_C_* = 29.3 (NCH_3_), 52.5 (OCH_3_), 115.0 (C-5a′), 121.0 (C-5), 131.0 (C-6), 141.0 (C-5’), 150.4 (C-2), 153.8 (C-2′), 159.6 (C-4), 164.4 (C-4′), 165.7 ppm (C-5b′). ^15^N NMR (DMSO-*d_6_*): *δ_N_* = 127.2 (N-1), 158.0 (N-3), 160.9 ppm (N-3′). Anal. Calcd for C_11_H_10_N_4_O_5_S (310.29): C, 42.58; H, 3.25; N, 18.06; S, 10.33. Found: C, 42.70; H, 3.28; N, 18.15; S, 10.44.

#### 3.2.12 (*Z*)-Methyl-2-((*Z*)-3-allyl-2-((2,4-dioxo-1,2,3,4-tetrahydropyrimidin-5-yl)imino)-4-oxothiazolidin-5-ylidene)acetate (5l)

Yield: 78%; mp 314–316°C, IR (KBr) υ_max_/cm^−1^ = 3147 (NH), 2999 (Ar-CH), 1710 (CO), 1675 (CO), 1644 (C=N), 1511 (C=C). ^1^H NMR (DMSO-*d_6_*): *δ_H_* = 3.77 (s, 3H, OCH_3_), 4.21 (d, 2H, *J* = 5.2 Hz, NCH_2_), 5.19 (dd, 1H, *J* = 10.3 Hz, 1.0 Hz, 3c′CH), 5.22 (dd, 1H, *J* = 17.2 Hz, 1.1 Hz, CH-3c′), 5.80 (ddt, 1H, *Jd* = 17.2, 10.4 Hz, *Jt* = 5.2 Hz, CH-3b′), 6.75 (s, 1H, CH-5a′), 7.24 (d, 1H, *J* = 6.6 Hz, CH-6), 10.89 (bs, 1H, ^1^N**H**), 11.42 ppm (bs, 1H, ^3^N**H**). ^13^C NMR (DMSO-*d_6_*): *δ_C_* = 42.6 (NCH_2_), 53.6 (OCH_3_), 116.1 (C-5a′), 116.5 (C-3c′), 120.6 (C-5), 130.0 (C-3b′), 134.4 (C-6), 140.3 (C-5′), 150.3 (C-2), 150.6 (C-2′), 159.8 (C-4), 163.5 (C-4′), 165.6 ppm (C-5b′). MS: *m/z* = 336 (M^+^, 35), 321 (20), 305 (10), 273 (5), 277 (14), 250 (25), 125 (100). Anal. Calcd for C_13_H_12_N_4_O_5_S (336.32): C, 46.43; H, 3.60; N, 16.66; S, 9.53. Found: C, 46.56; H, 3.64; N, 16.75; S, 9.66.

## 4 Biology

Supplementary Appendix SA contains information on all biological assay tests.

## 5 Conclusion

Due to the importance of thiazolidinone-pyrimidine derivatives, we direct for the synthesis of 2,4-dioxo-1,2,3,4-tetrahydropyrimidin-5-yl)imino)-4-oxo-3-yl-thiazolidin-5-ylidene)acetates **5a-l** through the reaction of thioureas **3a-f** with diethyl/dimethyl acetylenedicarboxylates (**4a,b**). The structure of compounds was examined by ^1^H, ^13^C-NMR, 2D-NMR, and ^15^N-NMR spectroscopy and elemental analyses. Compounds **5b** and **5j** were the most potent EGFR and BRAF^V600E^ inhibitors and could be used as dual EGFR and BRAF^V600E^ inhibitors with promising antiproliferative properties. Moreover, the synthesized molecules were *in-silico* inspected towards EGFR and BRAF^V600E^ as anticarcinoma drug candidates using AutoDock4.2.6 software. Based on docking scores, compounds **5b** and **5j** disclosed auspicious docking scores towards EGFR and BRAF^V600E^. These findings shed new light on the importance of compounds **5b** and **5j** as appropriate therapeutic treatments for cancer disease.

## Data Availability

The original contributions presented in the study are included in the article/Supplementary Material, further inquiries can be directed to the corresponding authors.
