# Supplementary material for: Design and synthesis of new thiazolidinone/uracil derivatives as antiproliferative agents targeting EGFR and/or BRAFV600E
Source: Front Chem. 2022 Dec 12;10:1076383. doi: 10.3389/fchem.2022.1076383 (PMC9792171; doi:10.3389/fchem.2022.1076383)
Supplement: Supplementary file 1 [file DataSheet1.docx]

**Design and synthesis of new thiazolidinone/uracil derivatives as antiproliferative agents targeting EGFR and/or BRAF^V600E^**

**Mohammed B. Alshammari^a^, Ashraf A. Aly ^b^****^*^, Bahaa G. M. Youssif^c*^ Stefan Bräse^d,e^, Akil Ahmad^a^ , Alan B. Brown, ^f^ Mahmoud A. A. Ibrahim, ^g^ Asmaa H. Mohamed ^b^**

^a^ Chemistry Department, College of Sciences and Humanities, Prince Sattam Bin Abdulaziz University, Al-Kharij, Saudi Arabia; ^b^ Chemistry Department, Faculty of Science, Minia University, 61519, El-Minia, Egypt; ^c^ Pharmaceutical Organic Chemistry Department, Faculty of Pharmacy, Assiut University, 71526, Assiut, Egypt; ^d^ Institute of Organic Chemistry, Karlsruher Institut fur Technologie, 76131 Karlsruhe, Germany; ^e^ Institute of Biological and Chemical Systems (IBCS-FMS), Karlsruhe Institute of Technology, 76344 Eggenstein Leopoldshafen, Germany; ^f^ Chemistry Department, Florida Institute of Technology, Melbourne, FL 32901, USA; ^g^ Computational Chemistry Laboratory, Chemistry Department, Faculty of Science, Minia University, Minia 61519, Egypt.


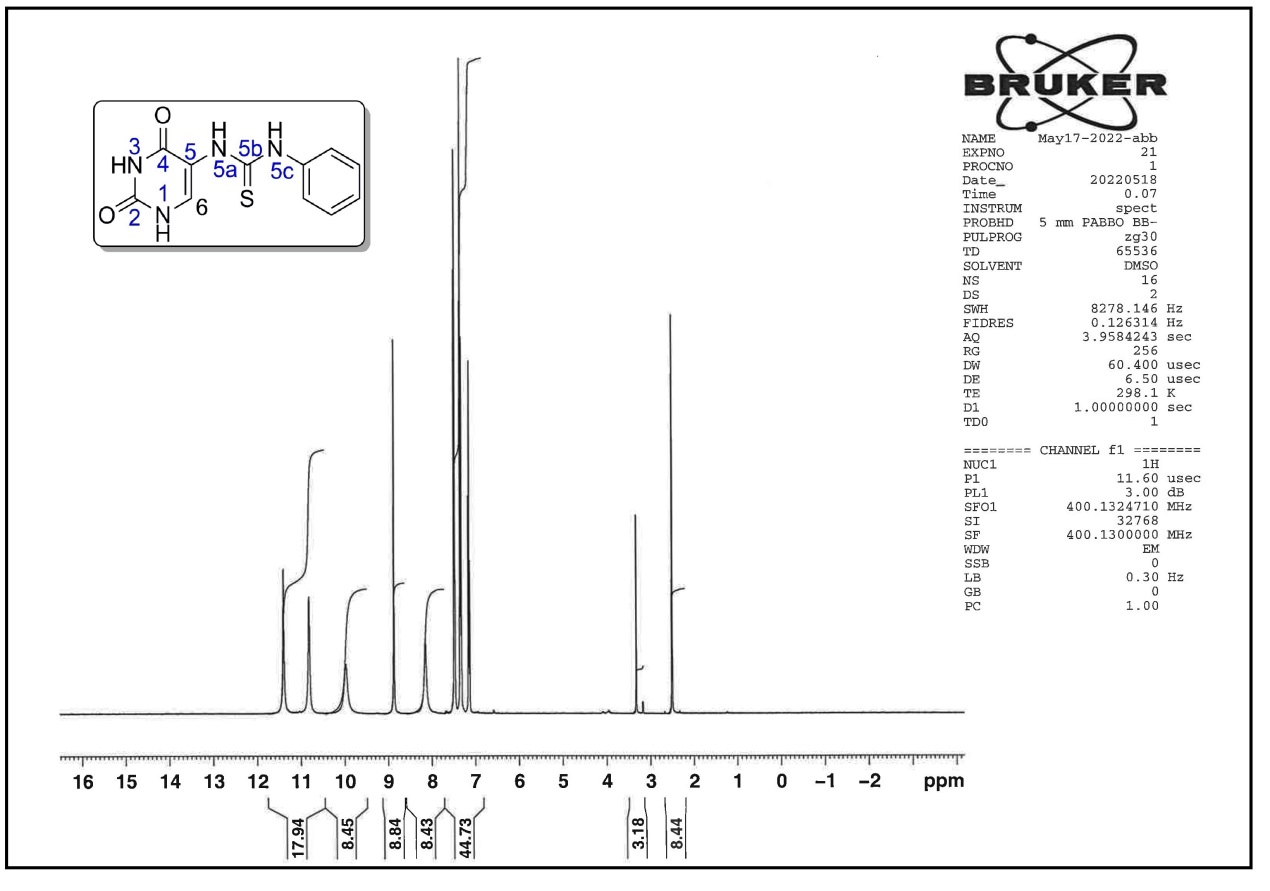


SI figure 1: ^1^H-NMR spectrum of **3a**


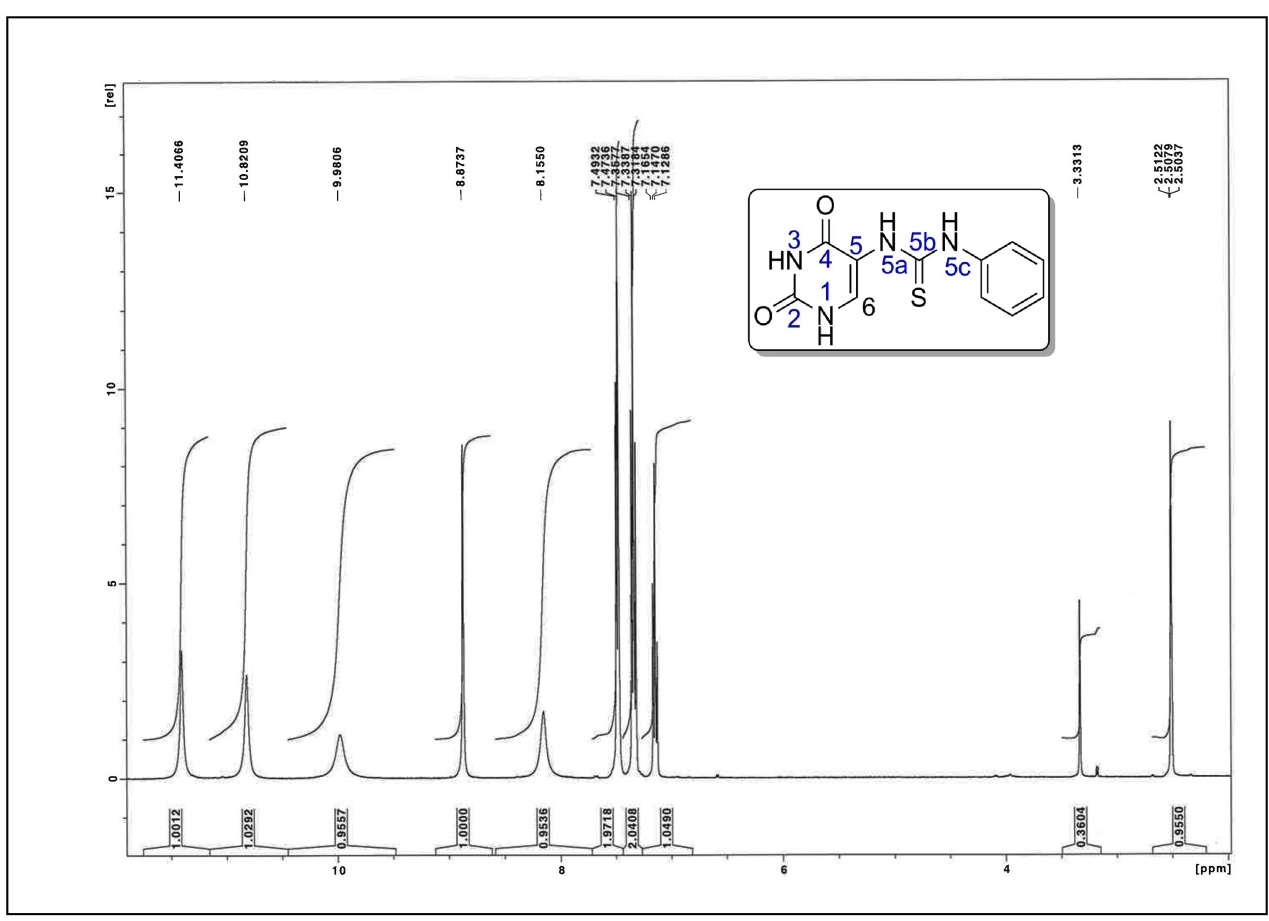


SI figure 2: ^1^H-NMR of spectrum **3a**

**
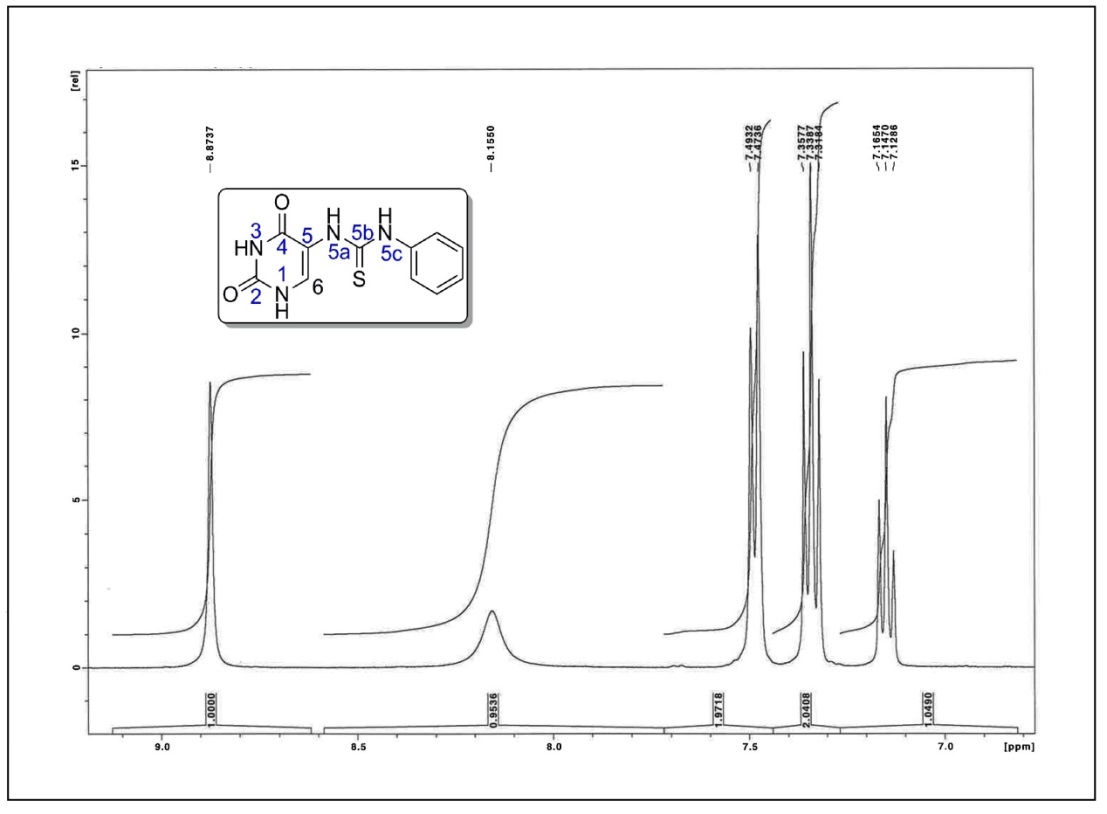
**

SI figure 3: ^1^H-NMR spectrum of **3a**

**
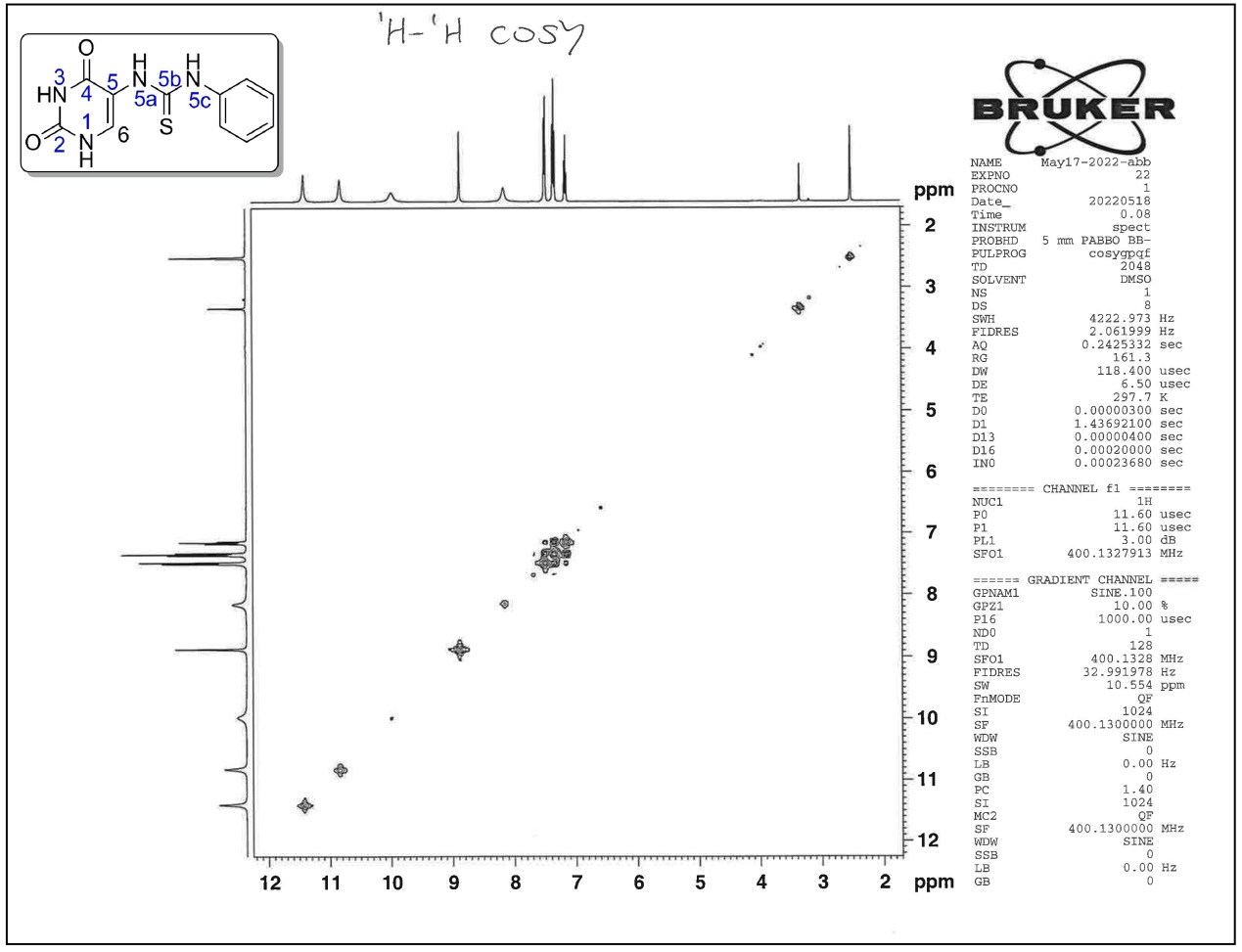
**

SI figure 4: ^1^H-^1^H COSY spectrum of **3a**

**
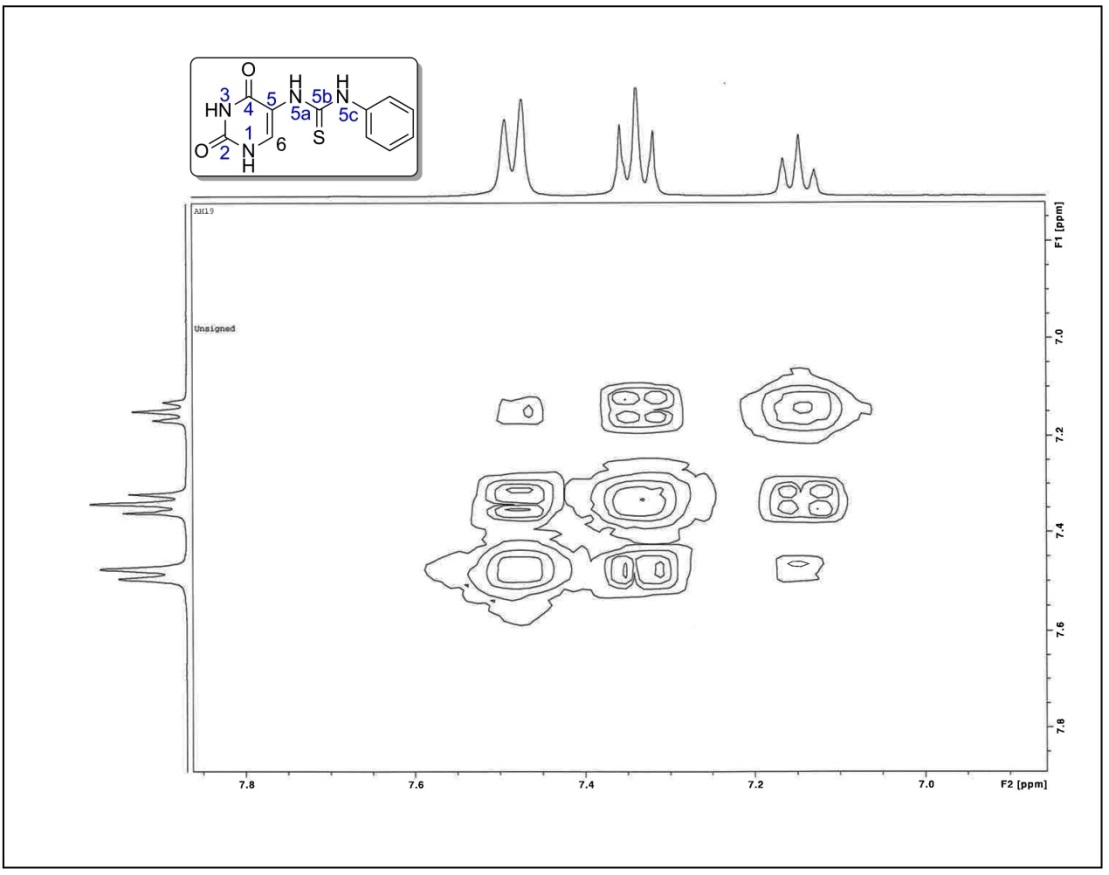
**

SI figure 5: ^1^H COSY spectrum of **3a**

**
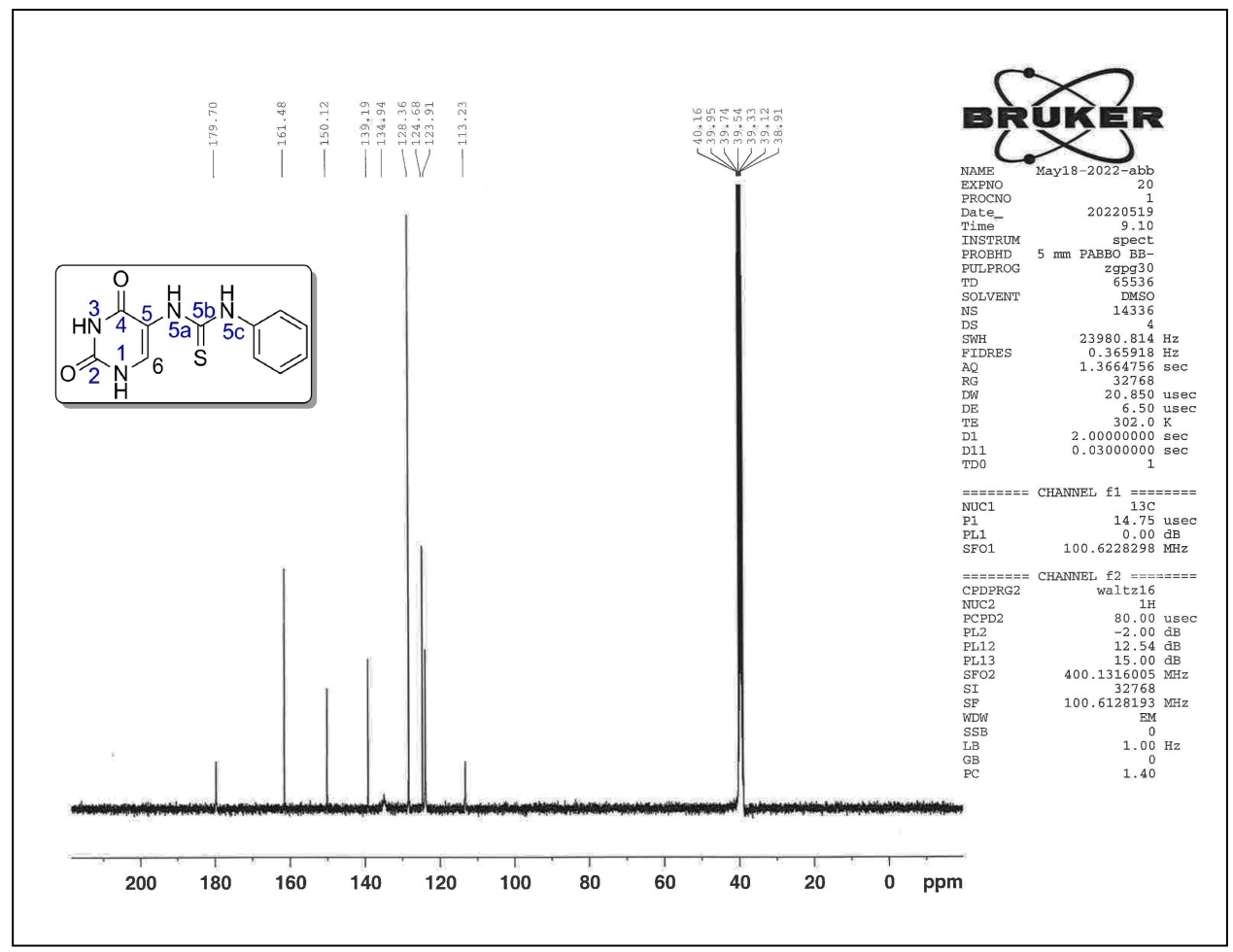
**

SI figure 6: ^13^C-NMR spectrum of **3a**

**
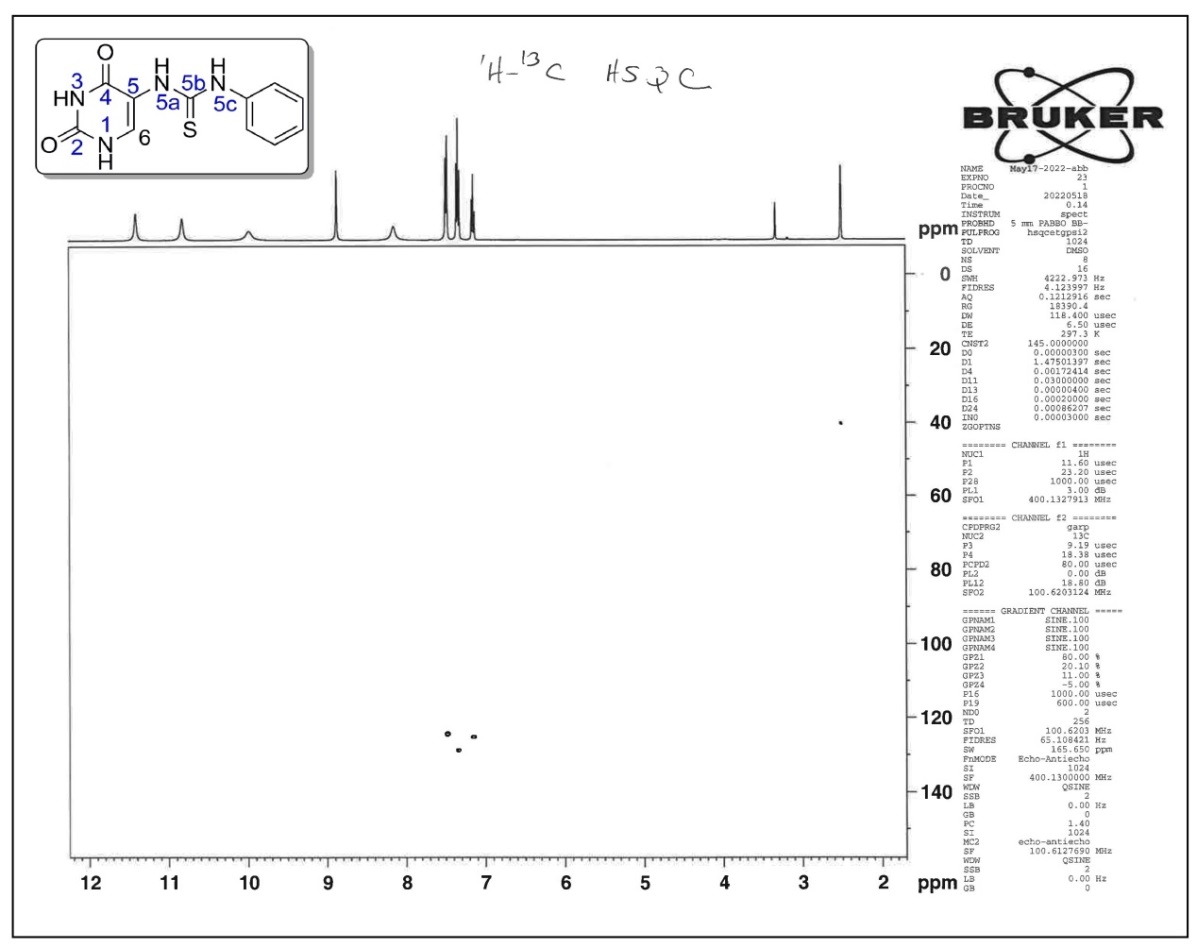
**

SI figure 7: ^1^H-^13^C-HSQC of spectrum **3a**

**
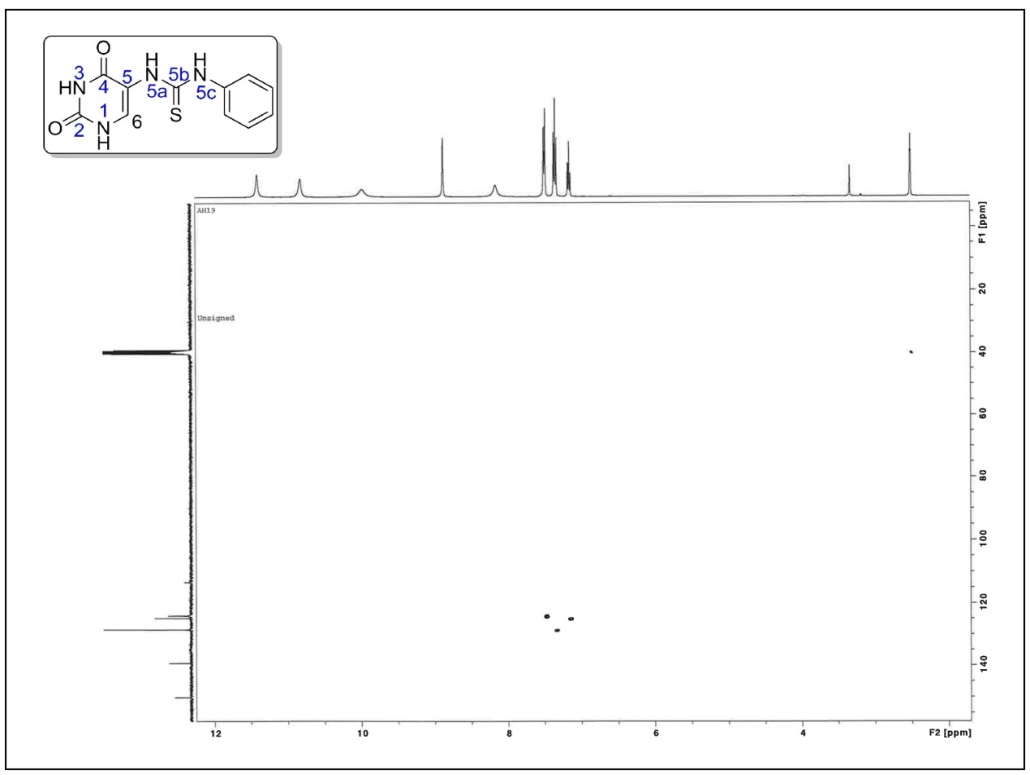
**

SI figure 8: ^1^H-^13^C-HSQC spectrum of **3a**

**
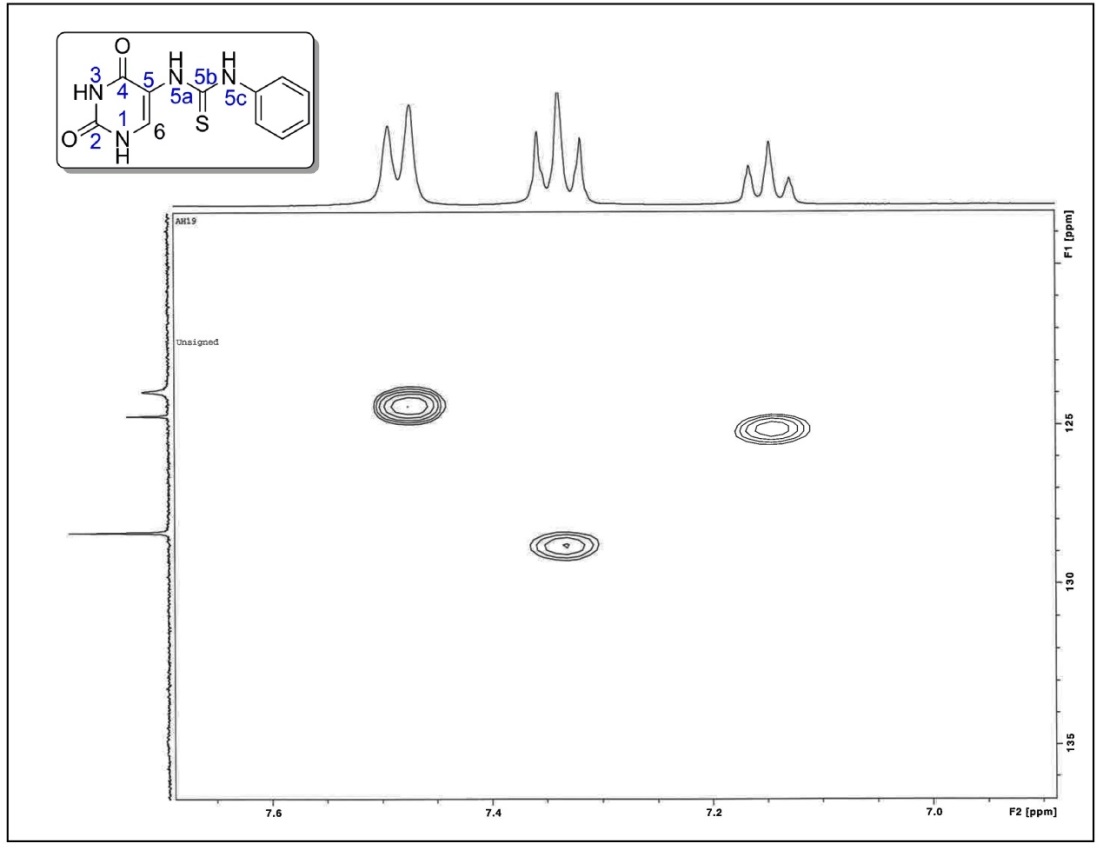
**

SI figure 9: ^1^H-^13^C-HSQC spectrum of **3a**

**
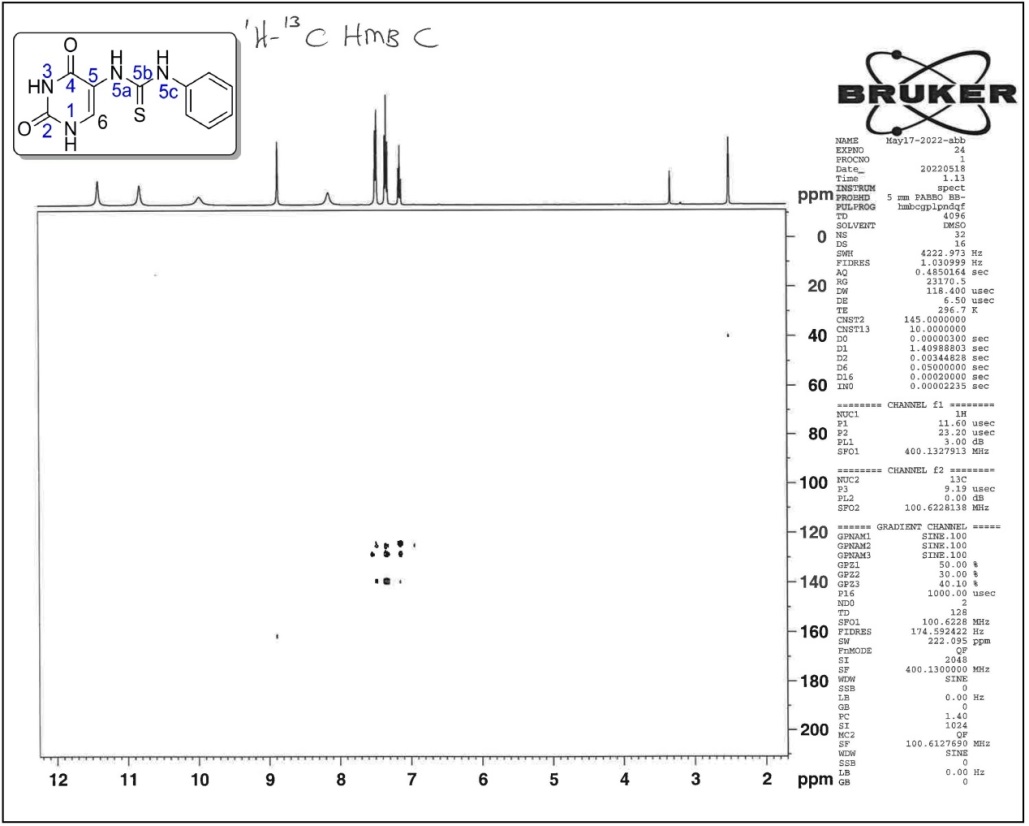
**

SI figure 10: ^1^H-^13^C-HMBC spectrum of **3a**

**
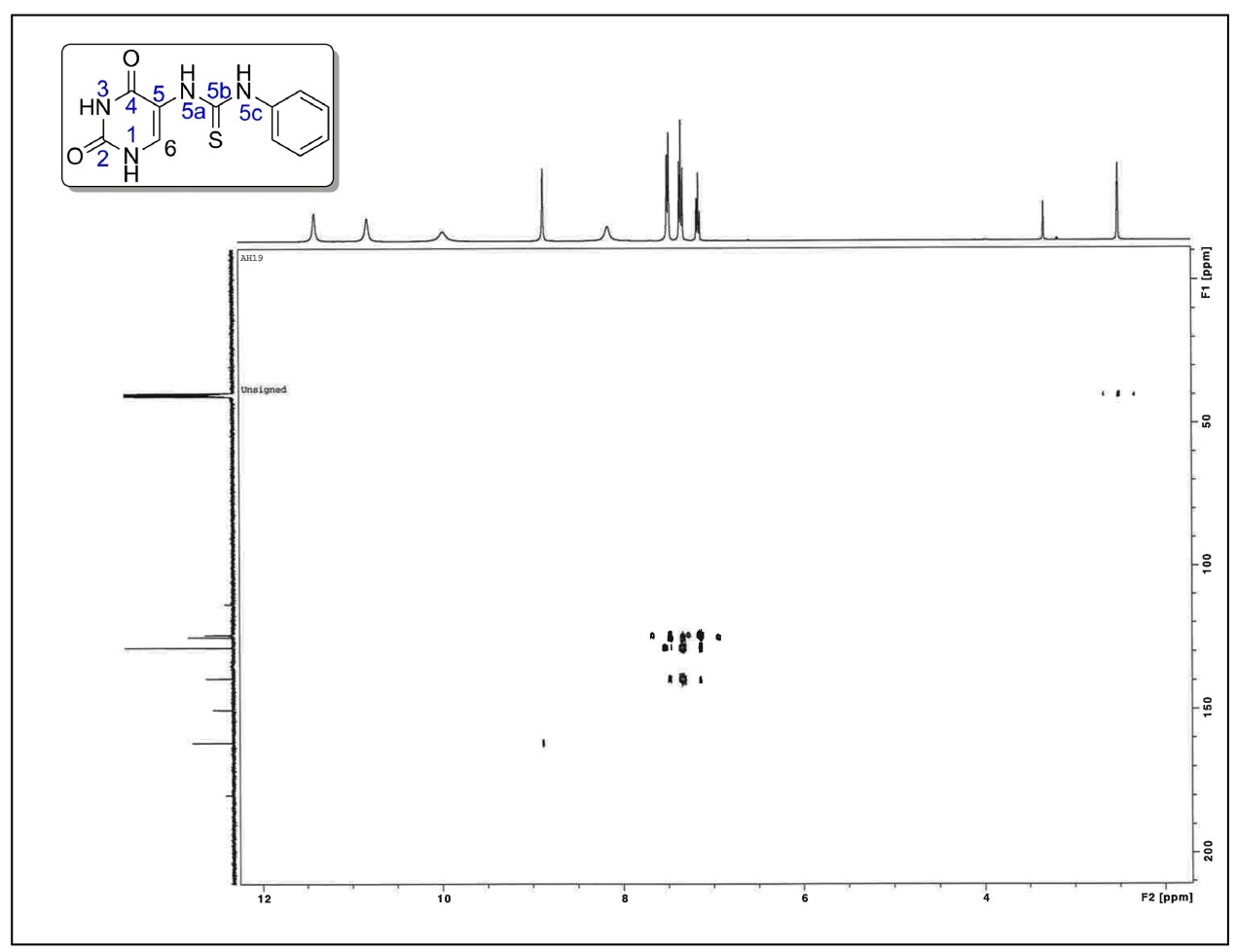
**

SI figure 11: ^1^H-^13^C-HMBC of spectrum **3a**

**
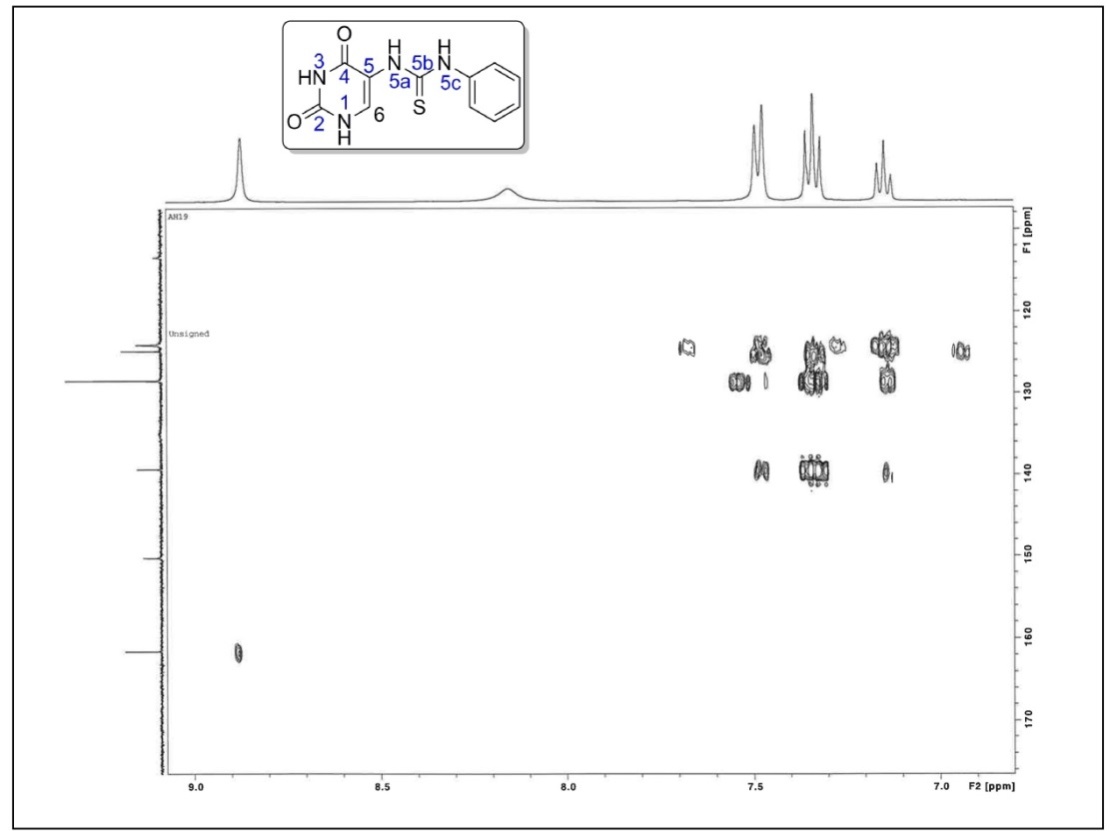
**

SI figure 12: ^1^H-^13^C-HMBC spectrum of **3a**

**
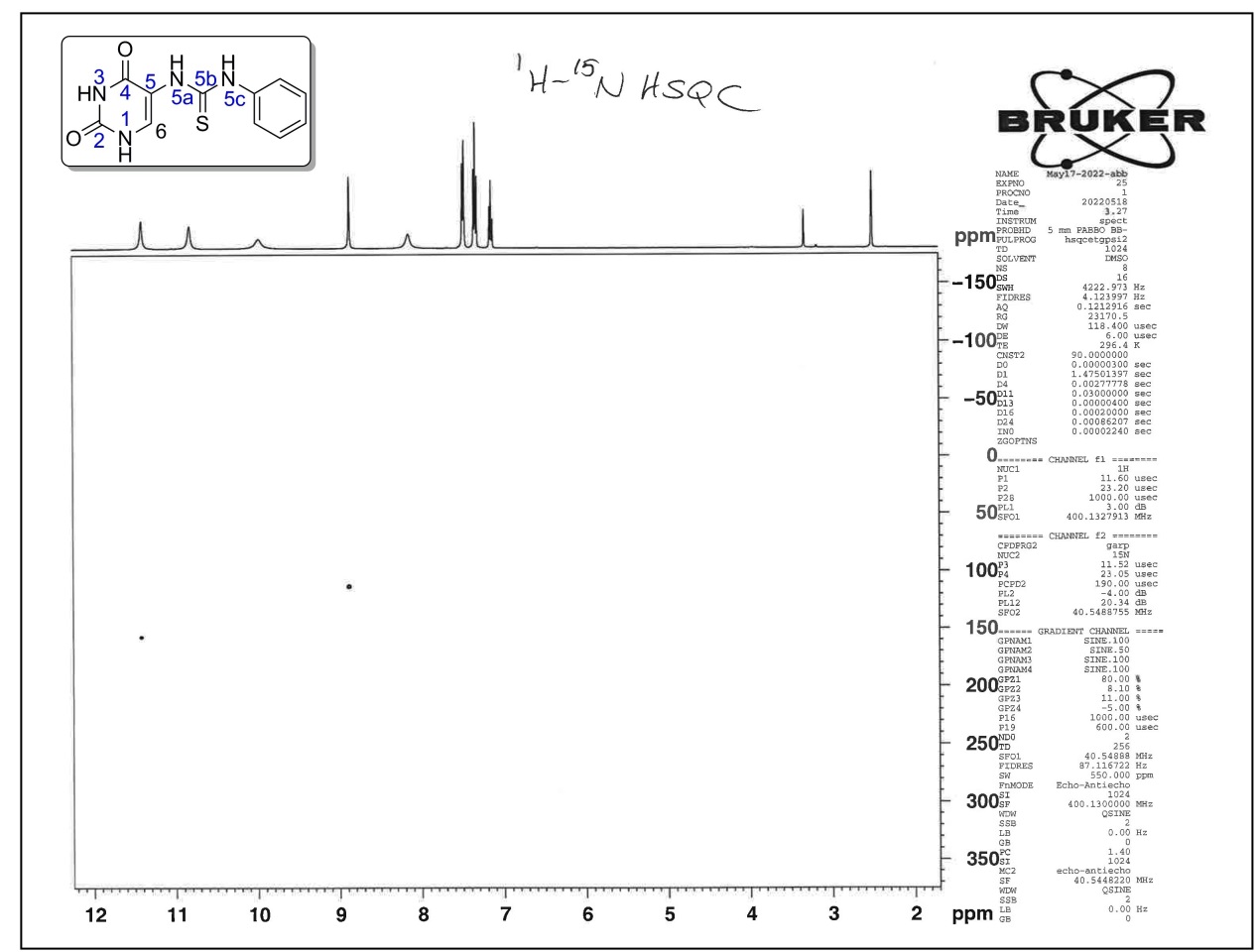
**

SI figure 13: ^1^H-^15^N-HSQC spectrum of **3a**

**
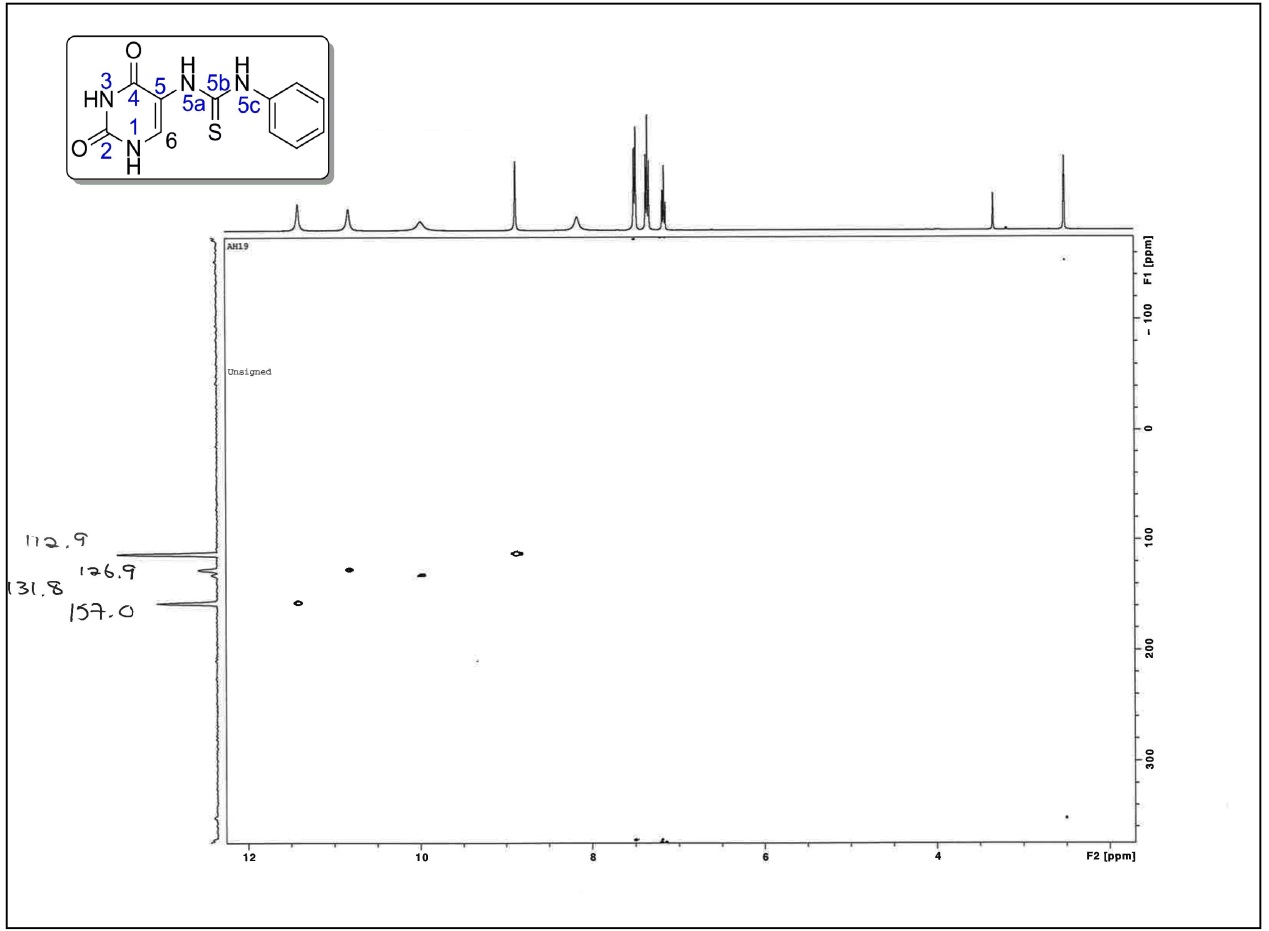
**

SI figure 14: ^1^H-^15^N-HSQC spectrum of **3a**

**
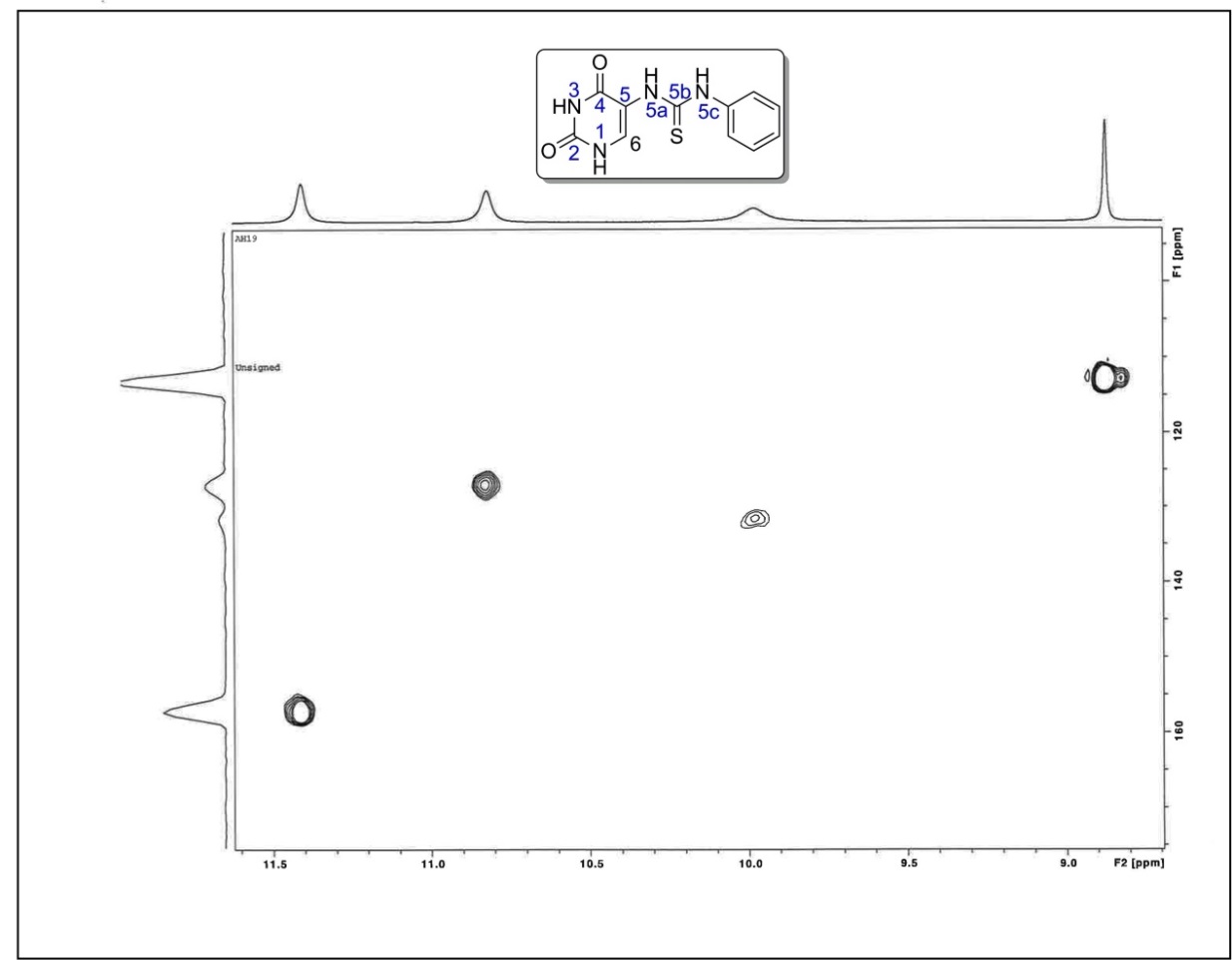
**

SI figure 15: ^1^H-^15^N-HSQC spectrum of **3a**

**
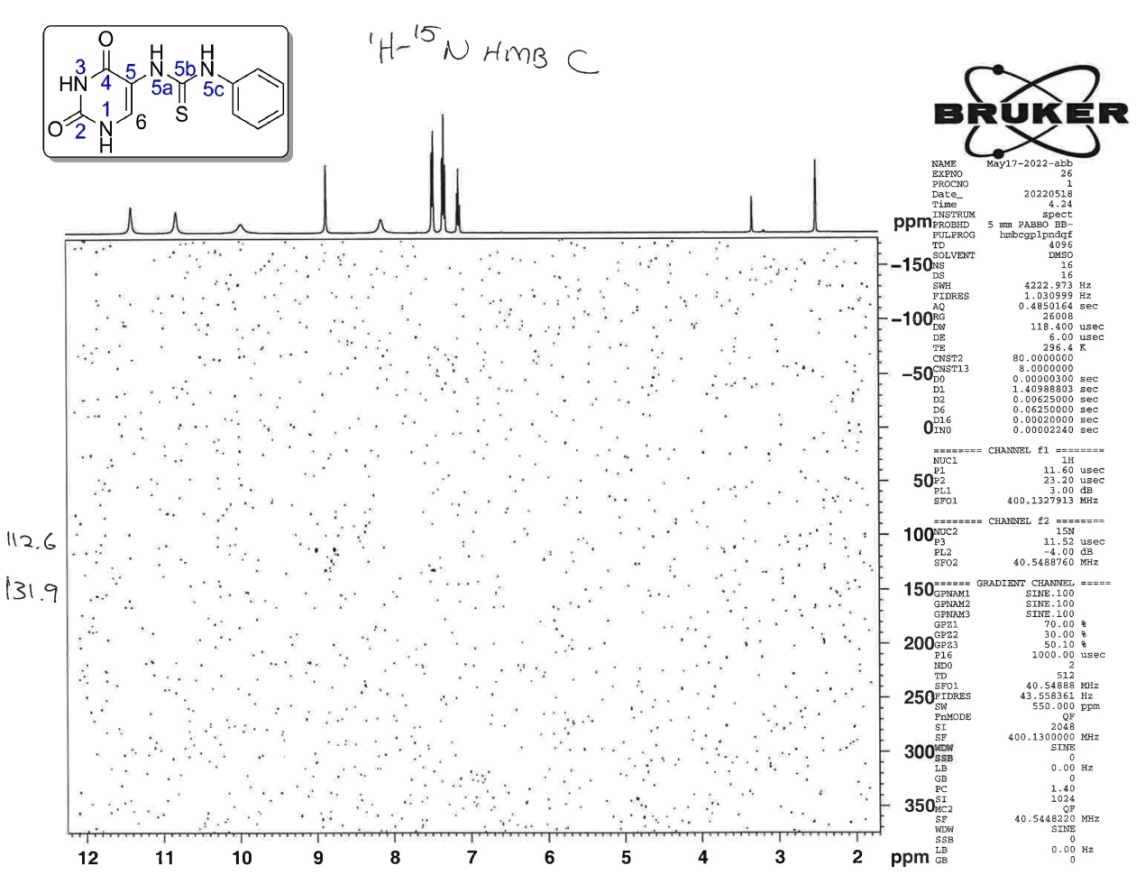
**

SI figure 16: ^1^H-^15^N-HMBC spectrum of **3a**

**
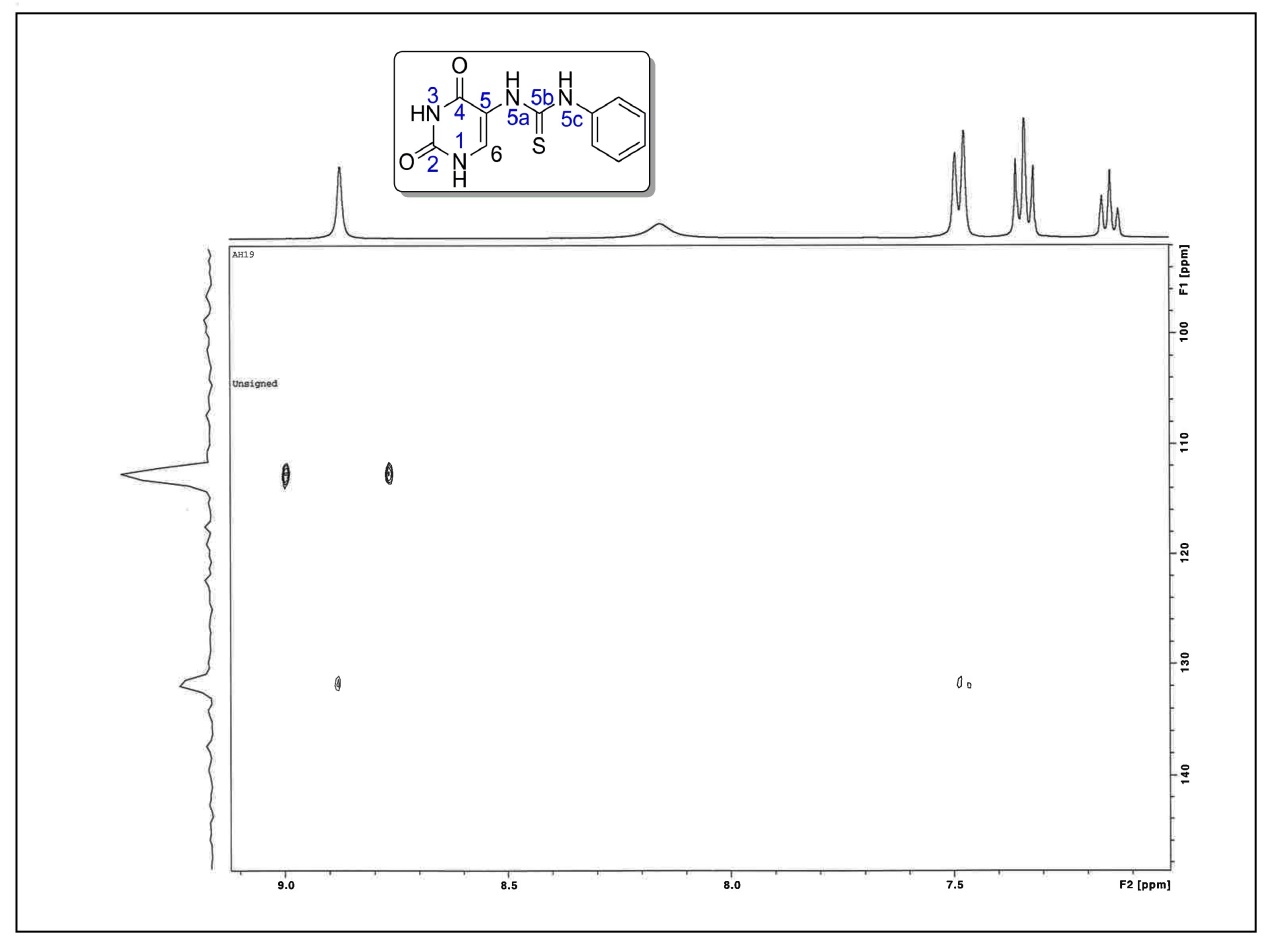
**

SI figure 17: ^1^H-^15^N-HMBC spectrum of **3a**

**
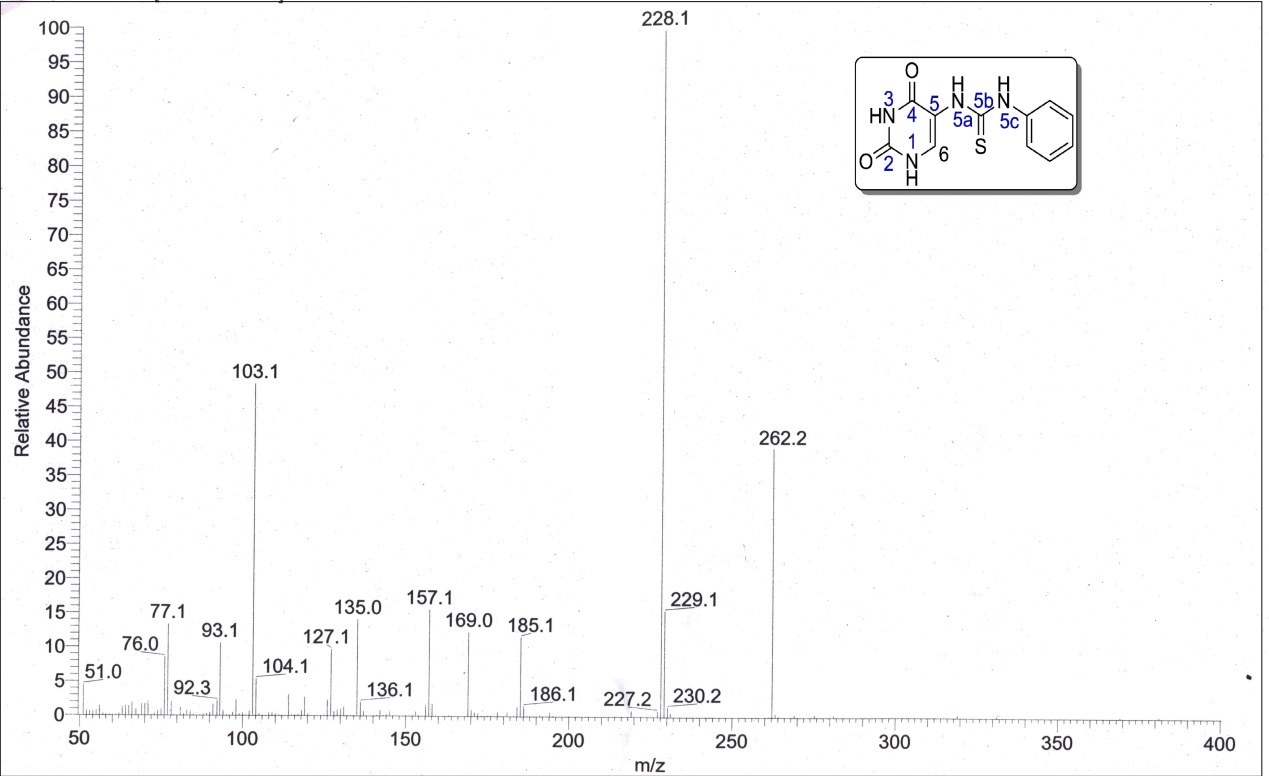
**

SI figure 18: Mass Spectroscopy of **3a**


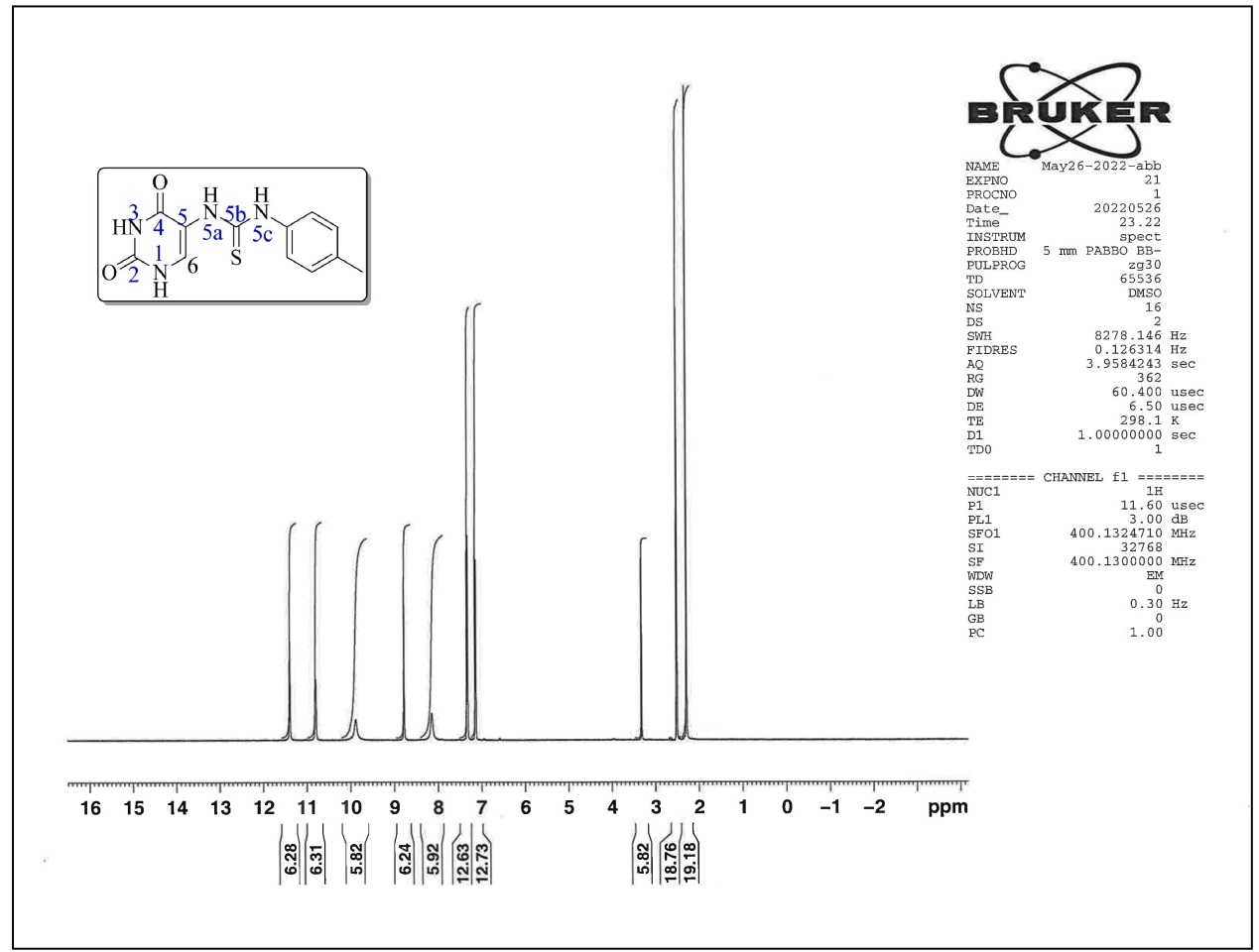


SI figure 19: ^1^H-NMR spectrum of **3b**


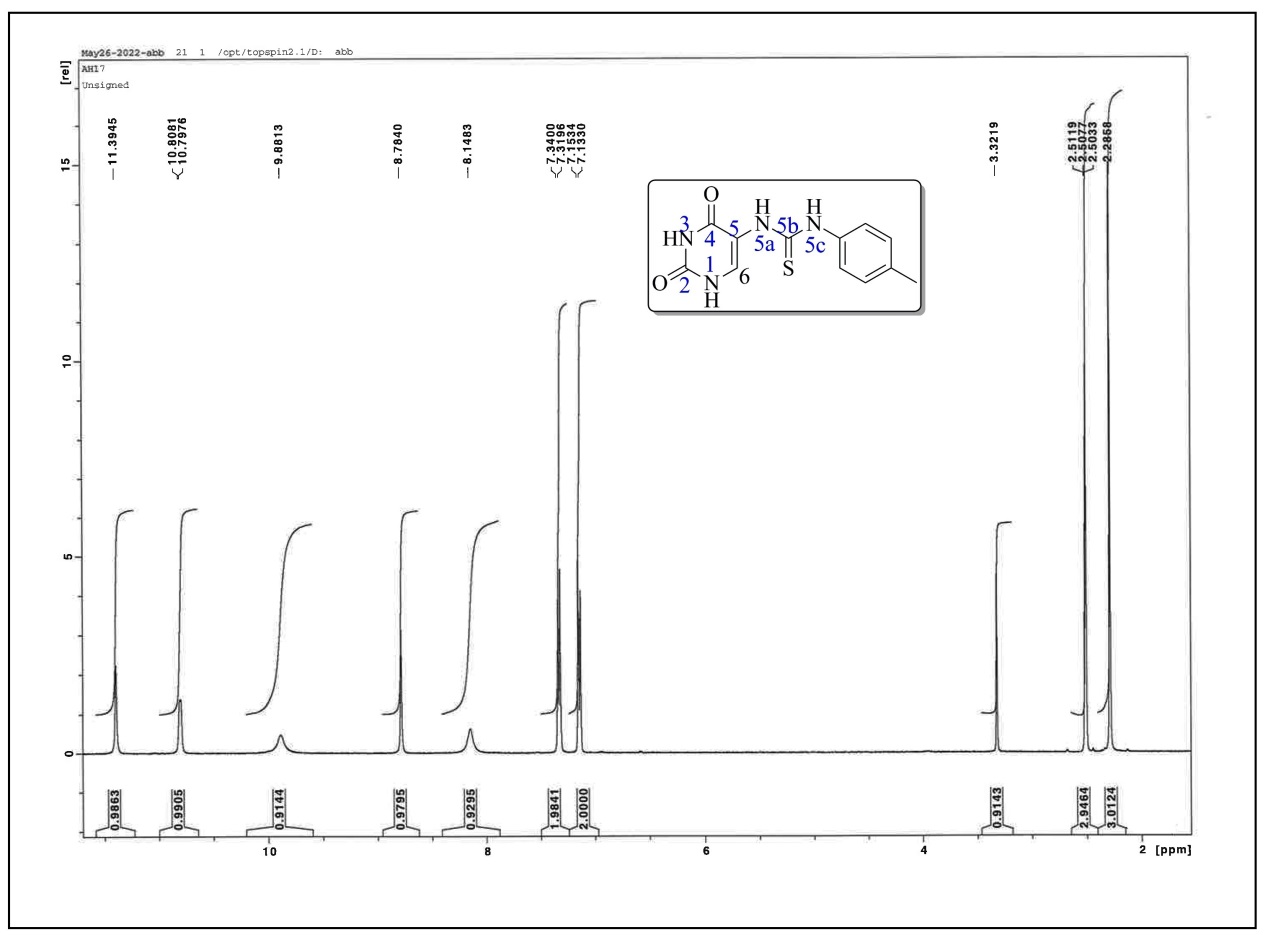


SI figure 20: ^1^H-NMRspectrum of **3b**


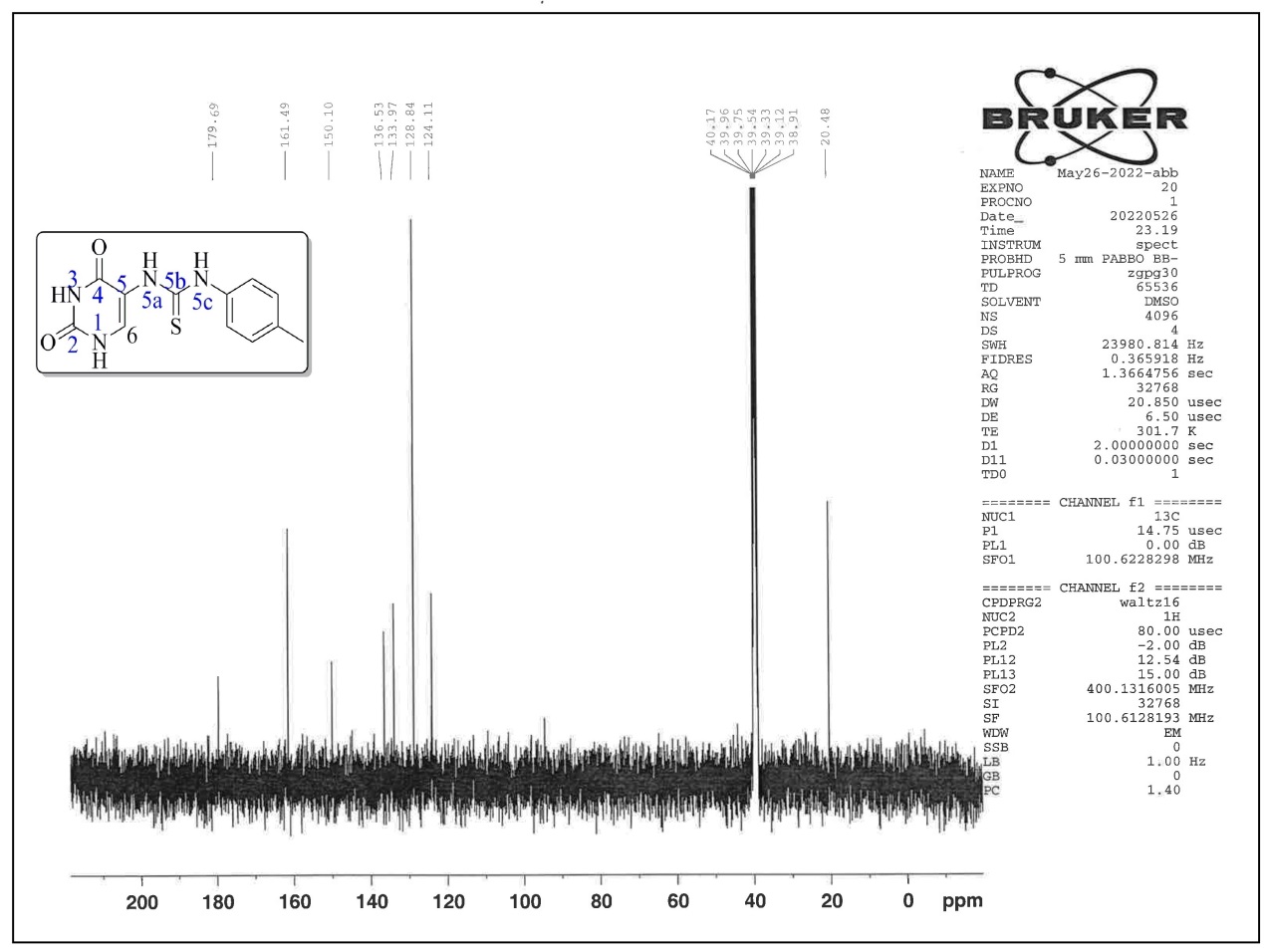


SI figure 21: ^13^C-NMR spectrum of **3b**


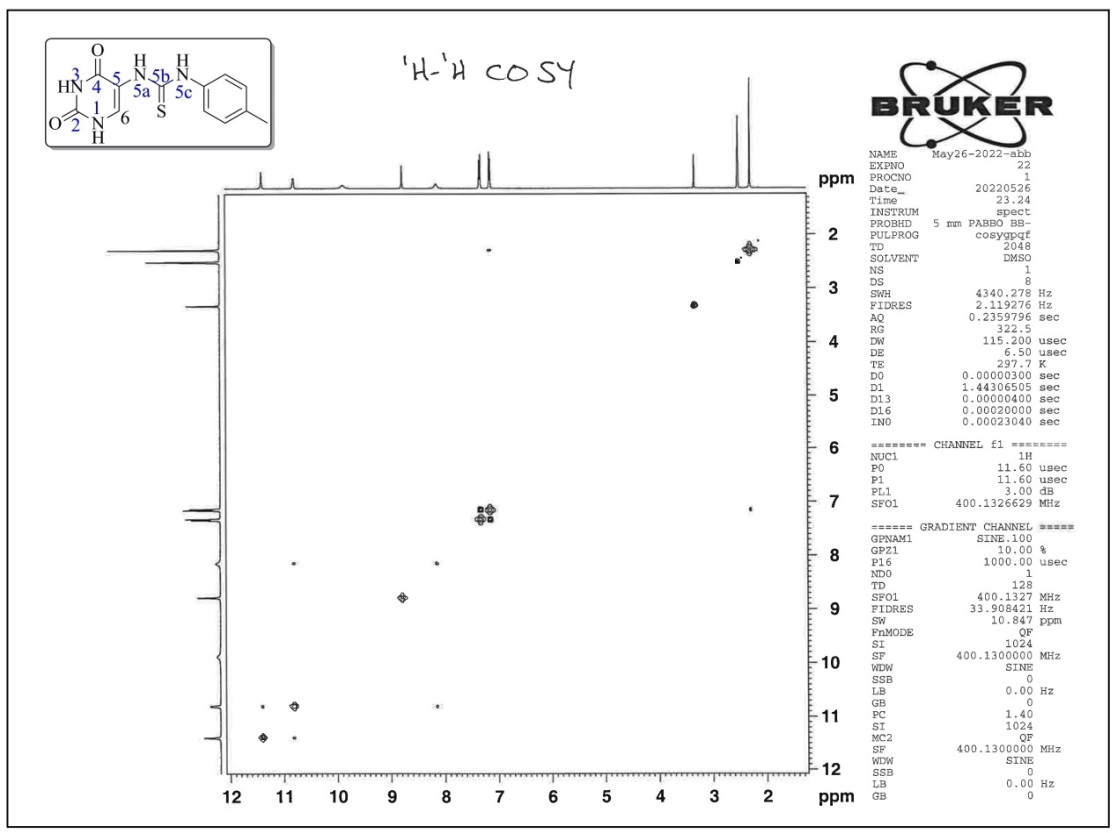


SI figure 22: ^1^H-^1^H COSY spectrum of **3b**


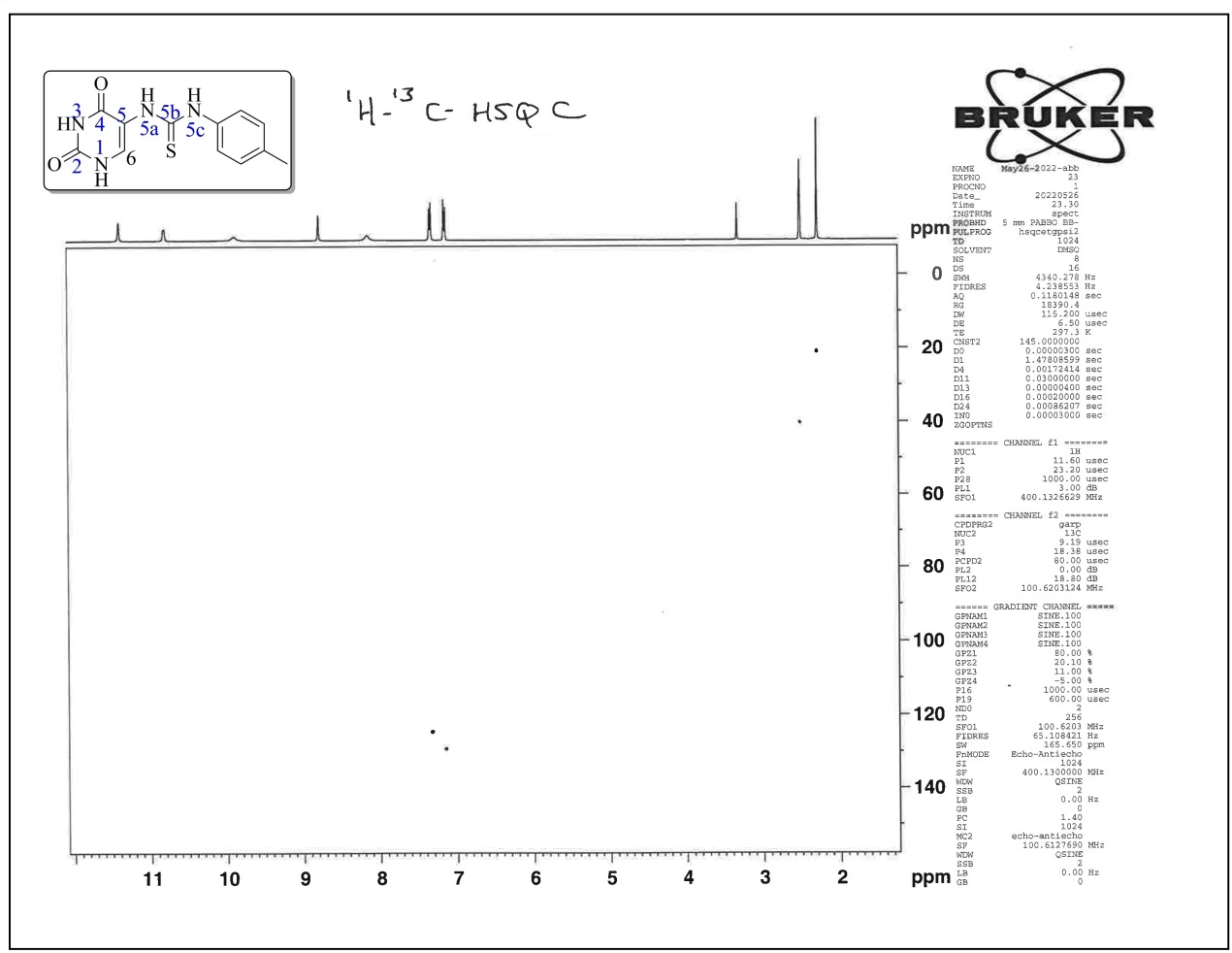


SI figure 23: ^1^H-^13^CHSQC spectrum of **3b**


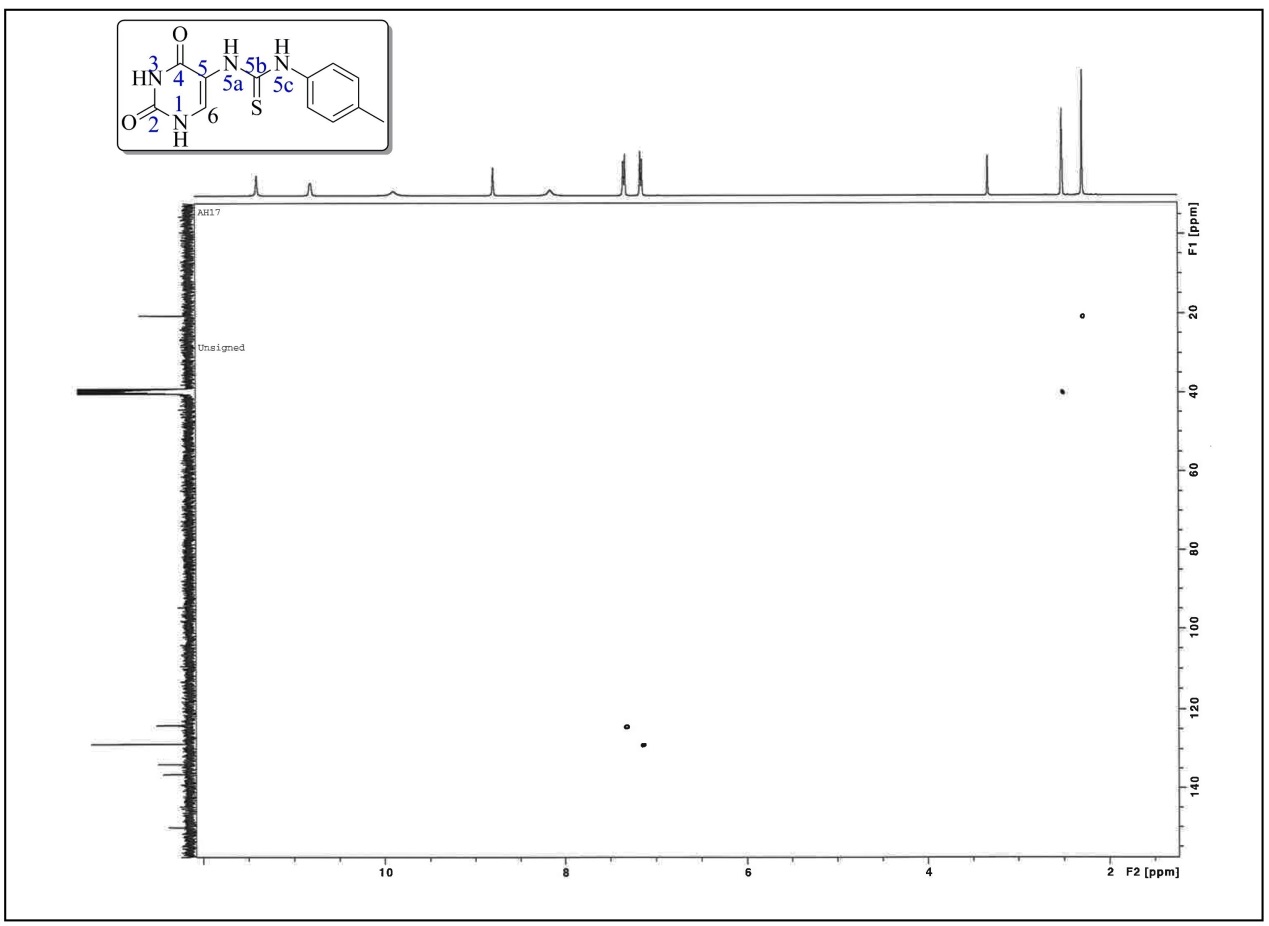


SI figure 24: ^1^H-^13^CHSQC spectrum of **3b**


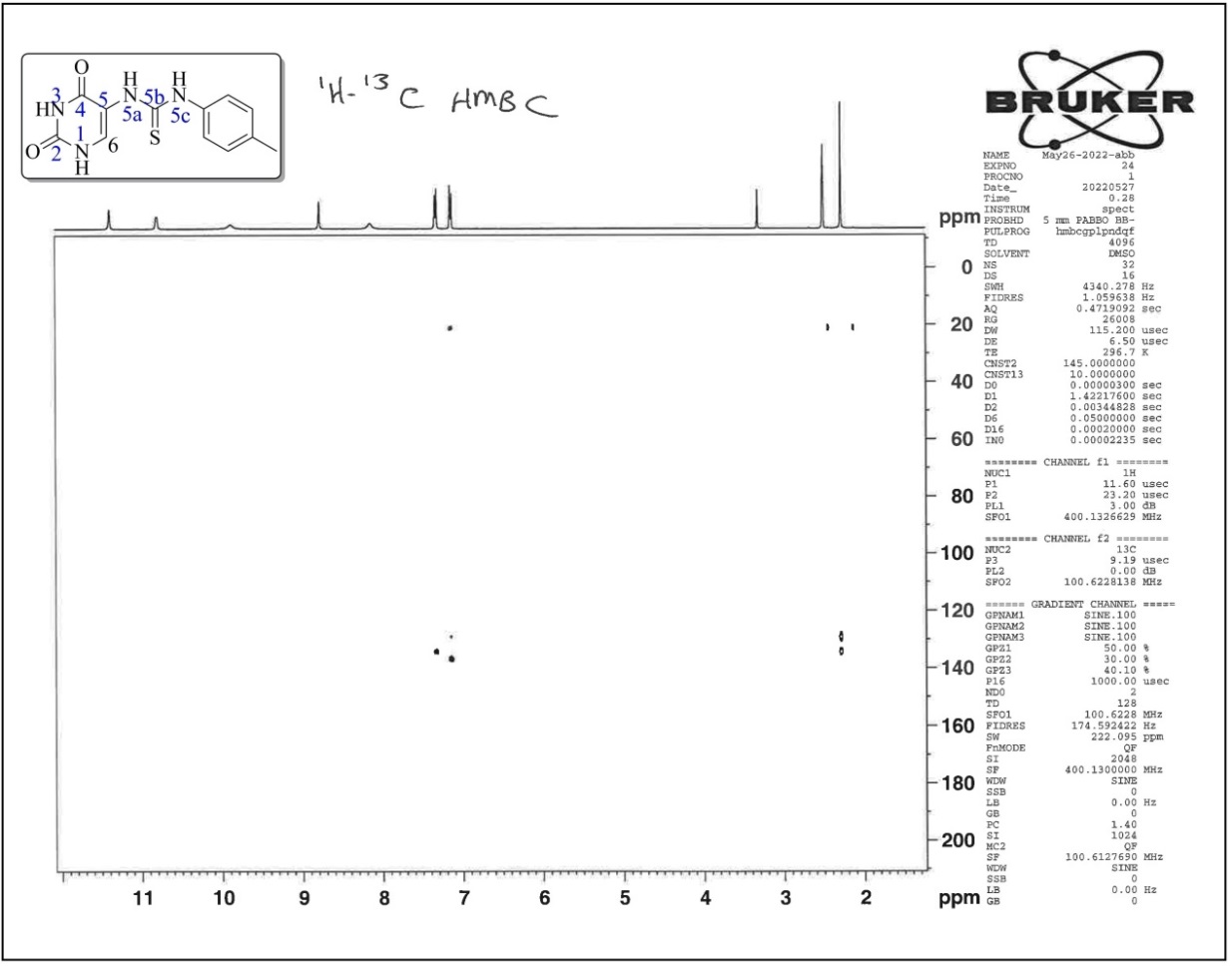


SI figure 25: ^1^H-^13^CHMBC spectrum of **3b**


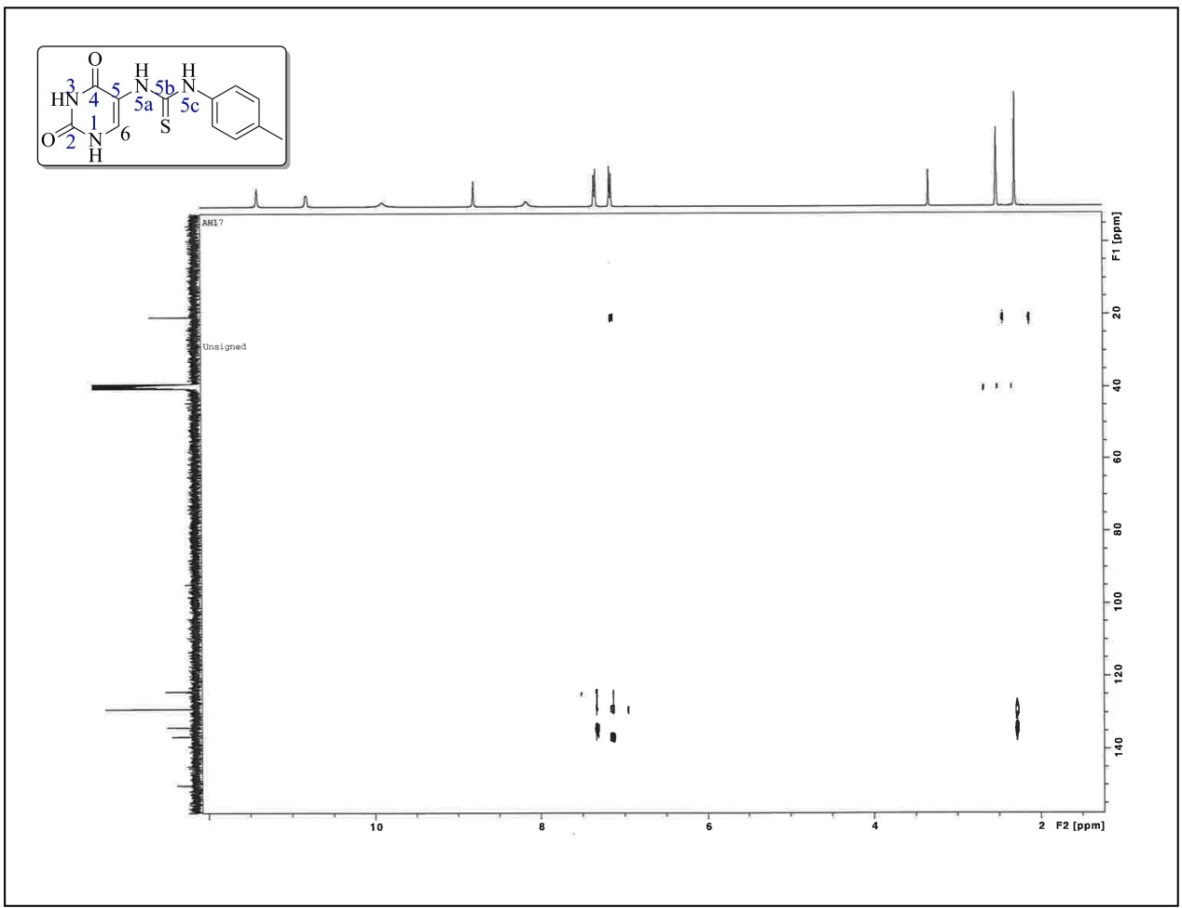


SI figure 26: ^1^H-^13^CHMBC spectrum of **3b**


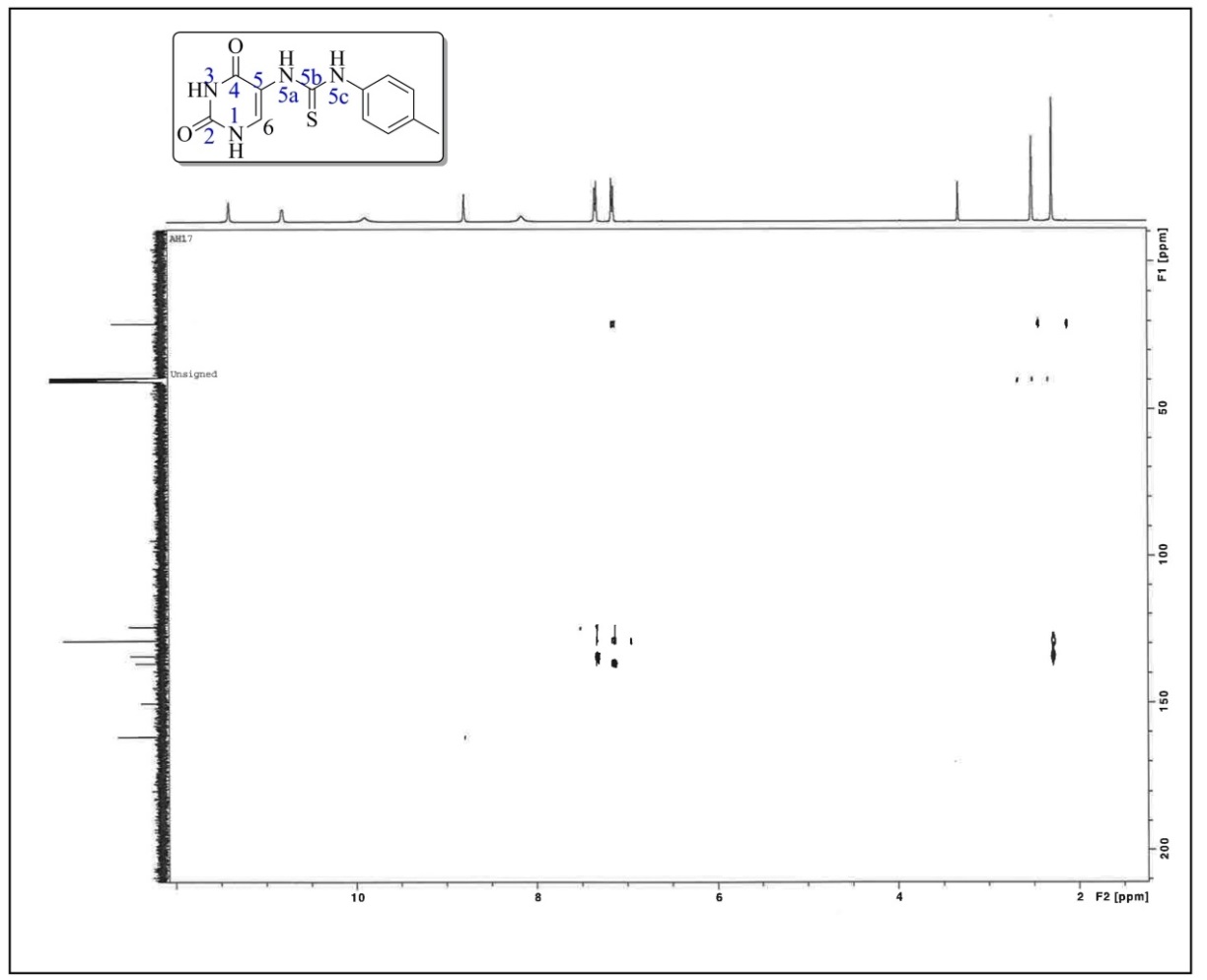


SI figure 27: ^1^H-^13^CHMBC spectrum of **3b**


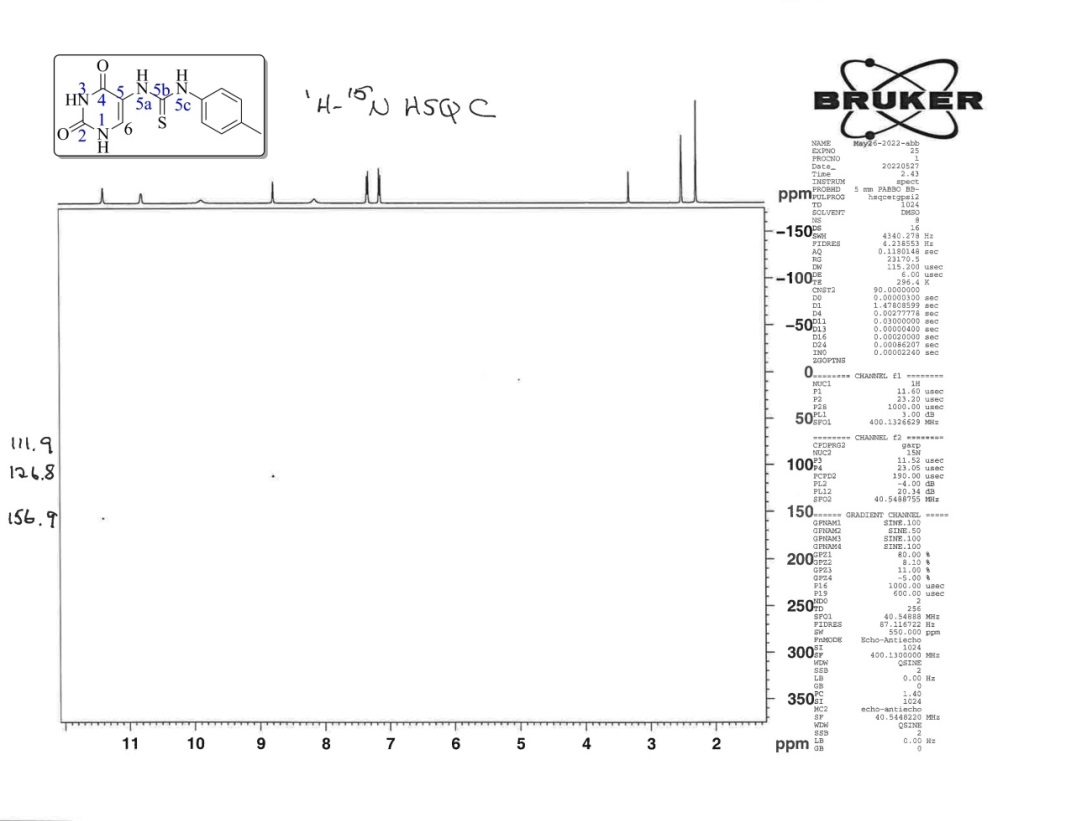


SI figure 28: ^1^H-^15^NHSQC spectrum of **3b**


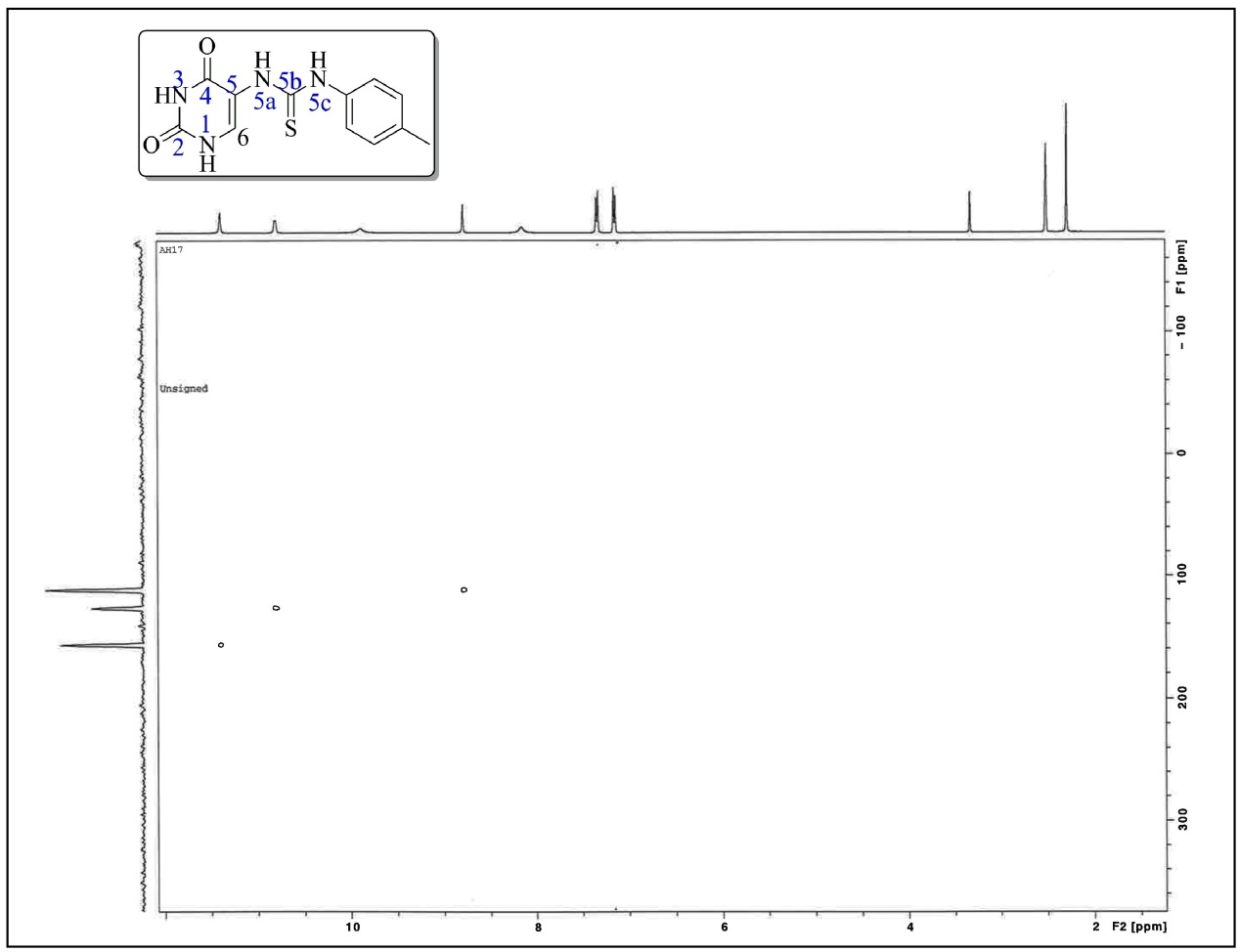


SI figure 29: ^1^H-^15^NHSQC spectrum of **3b**


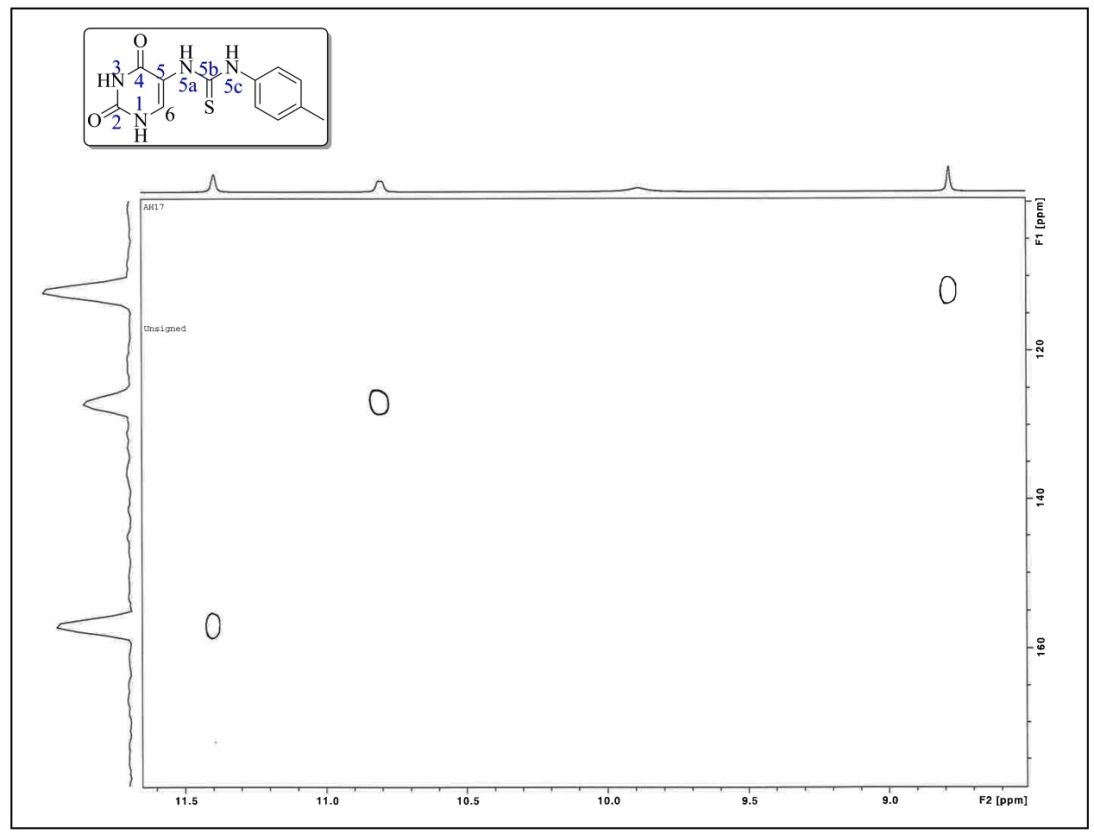


SI figure 30: ^1^H-^15^NHSQC spectrum of **3b**


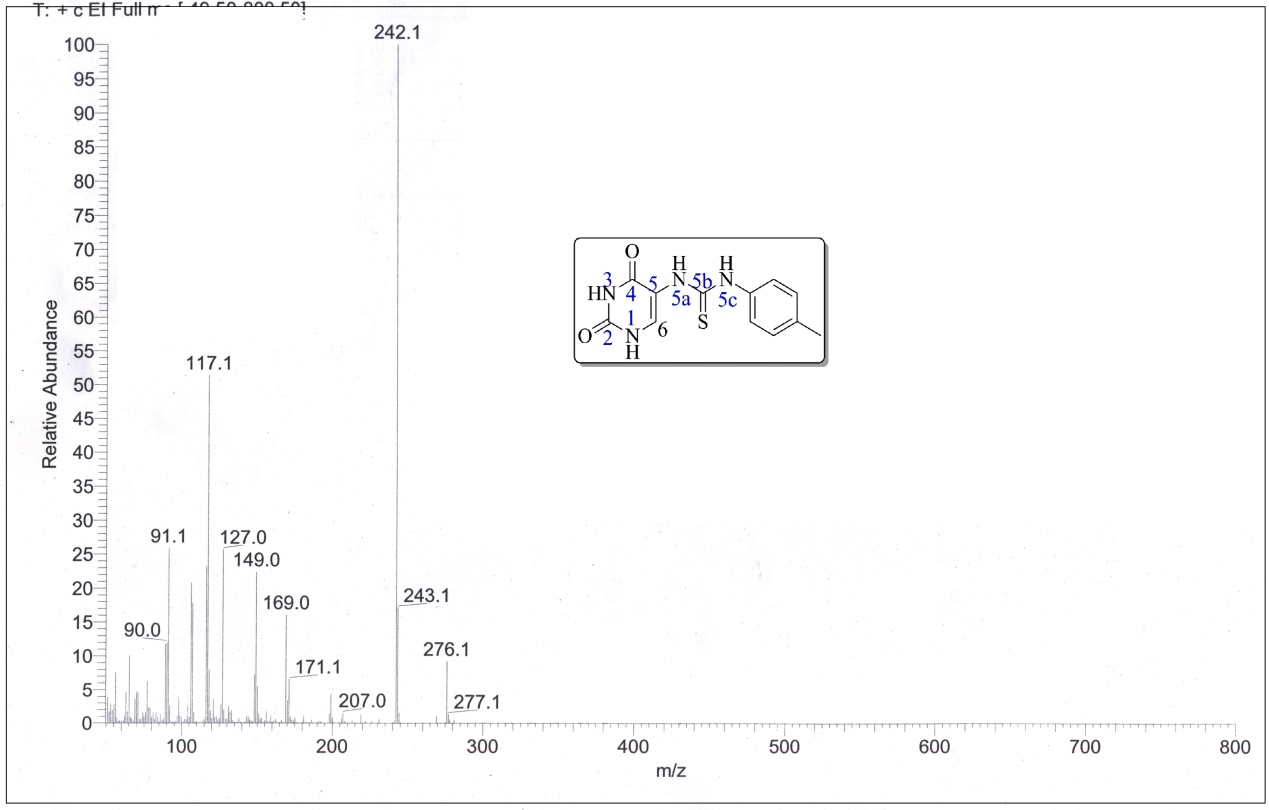


SI figure 30: Mass Spectroscopy of **3b**


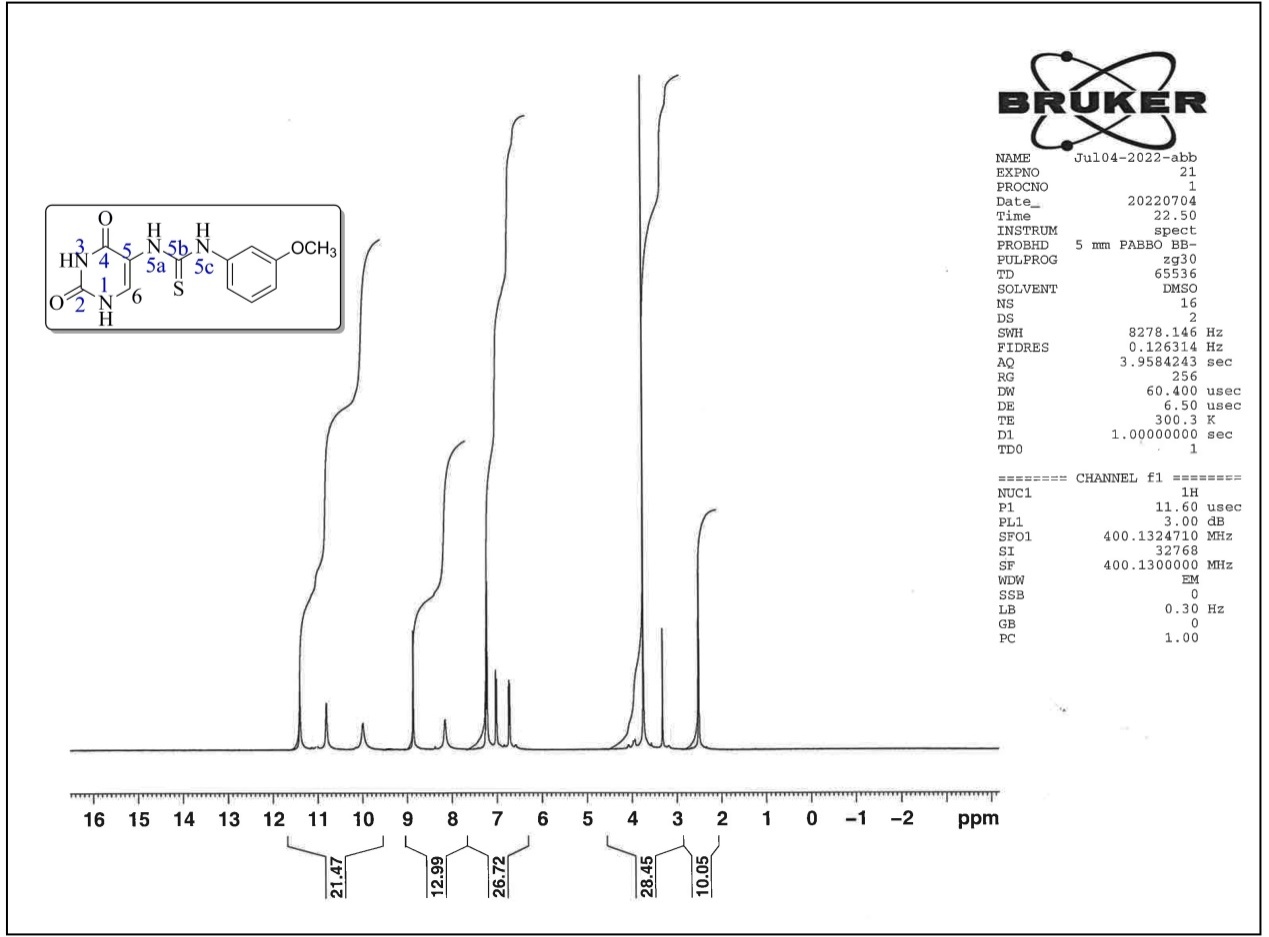


SI figure 31: ^1^H-NMR spectrum of **3c**


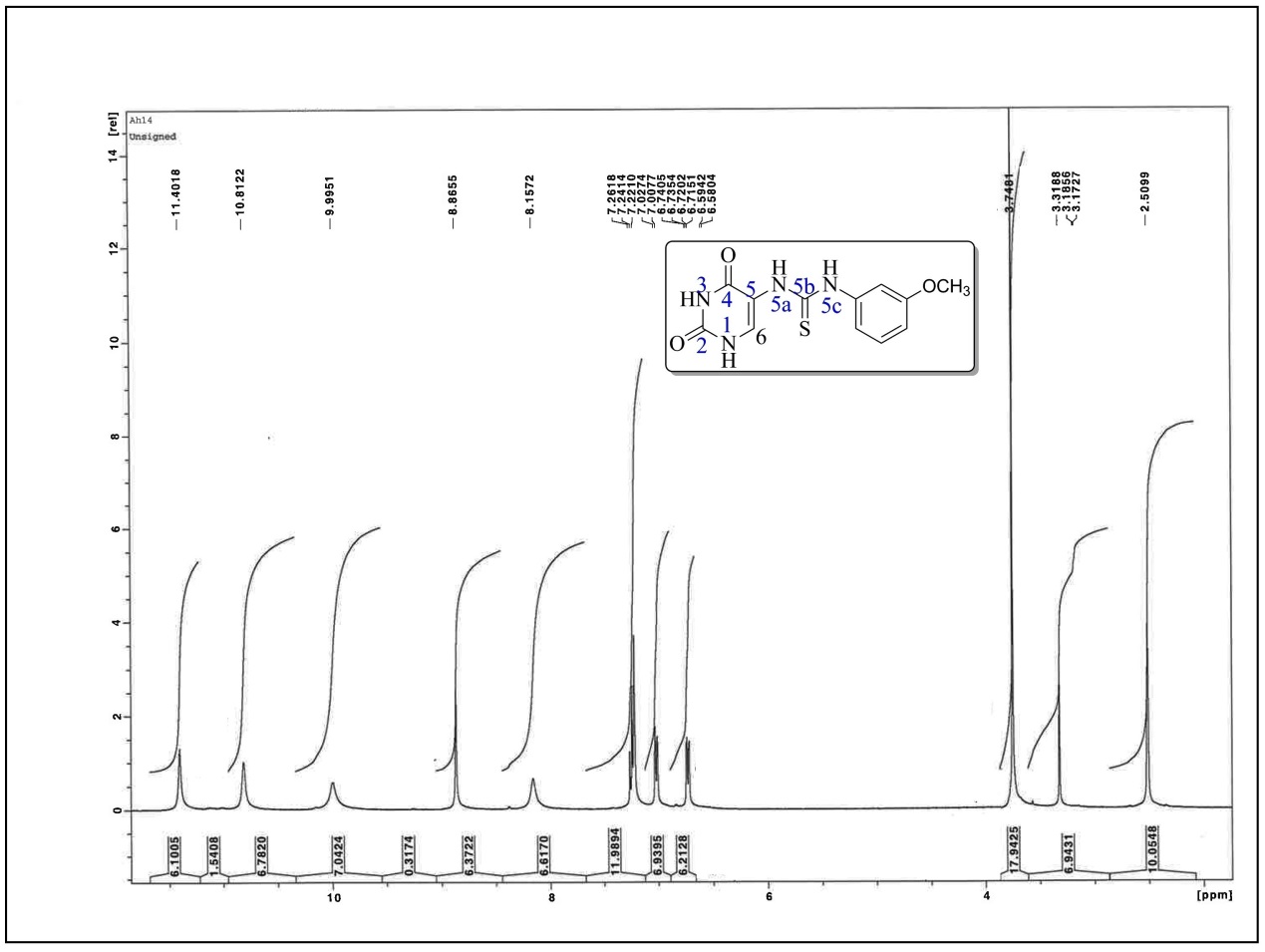


SI figure 32: ^1^H-NMR spectrum of **3c**


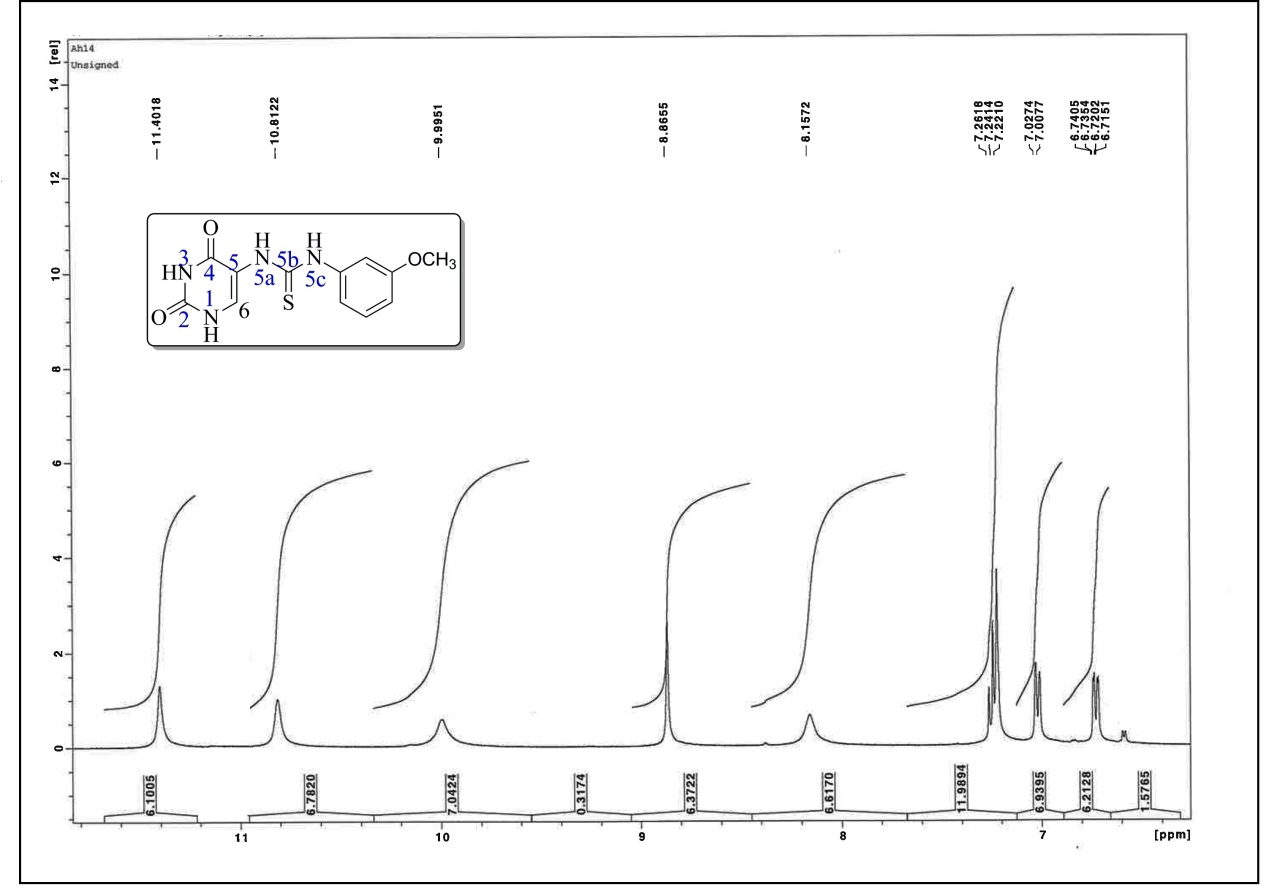


SI figure 33: ^1^H-NMR spectrum of **3c**


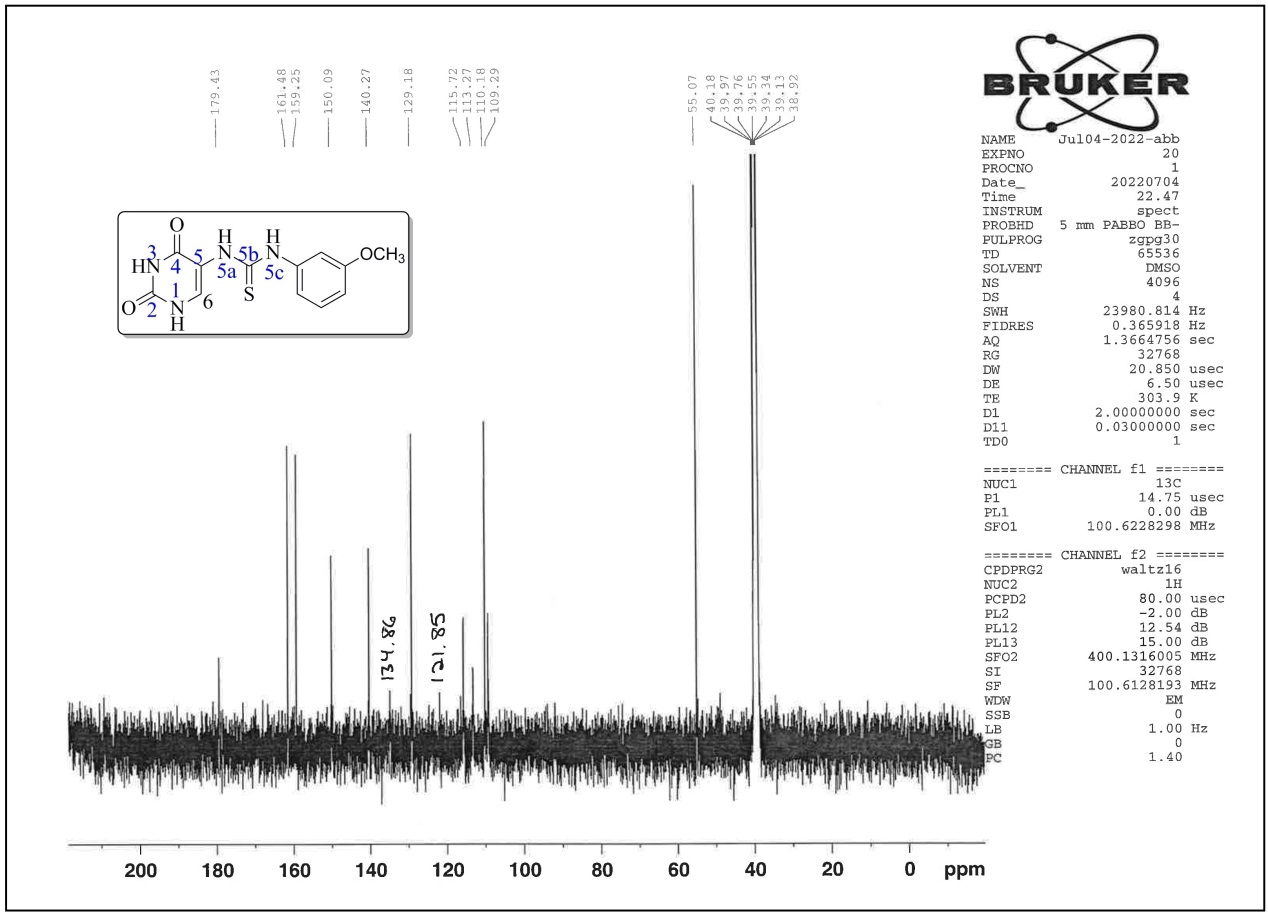


SI figure 34: ^13^C-NMR spectrum of **3c**


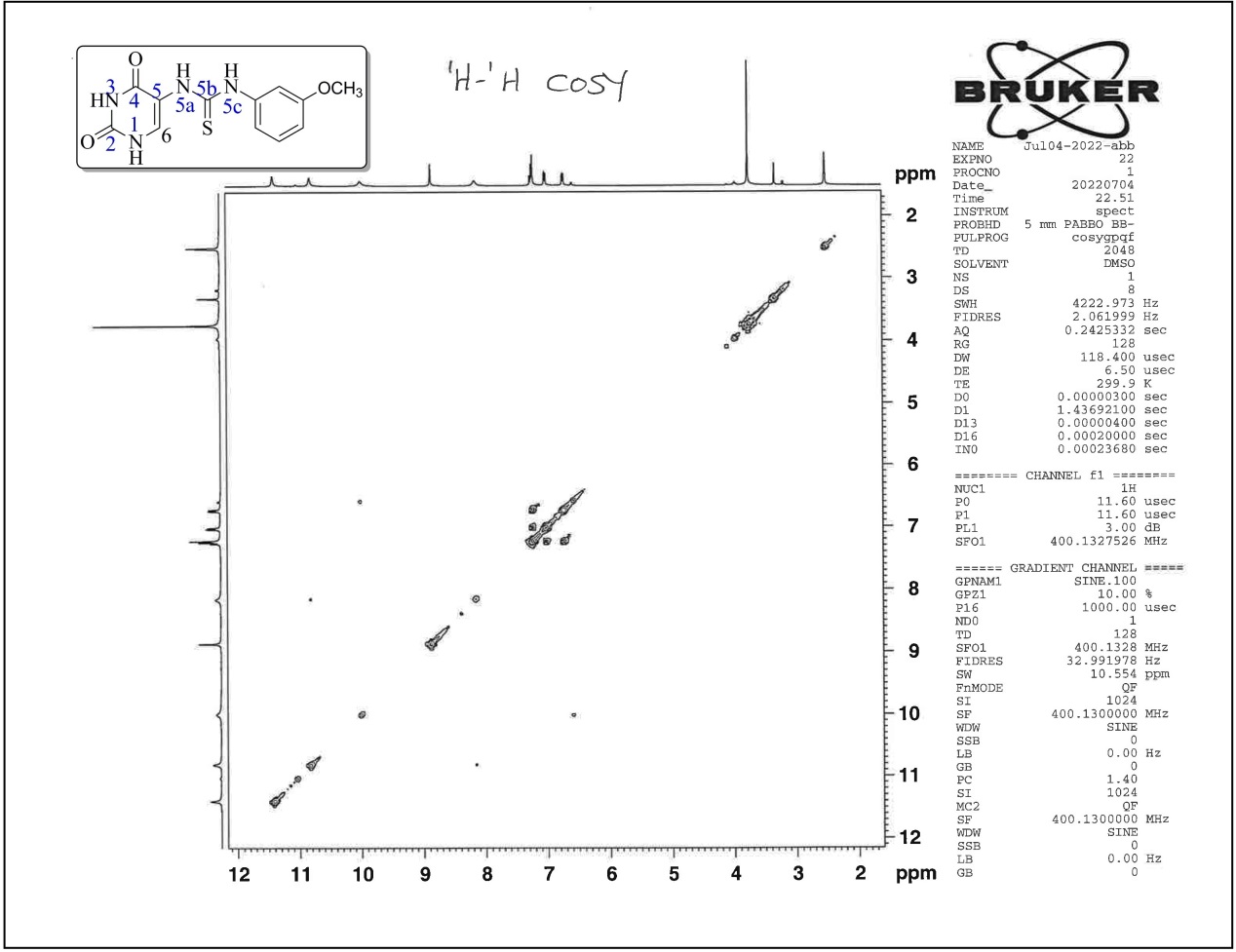


SI figure 35: ^1^H-^1^H COSY spectrum of **3c**


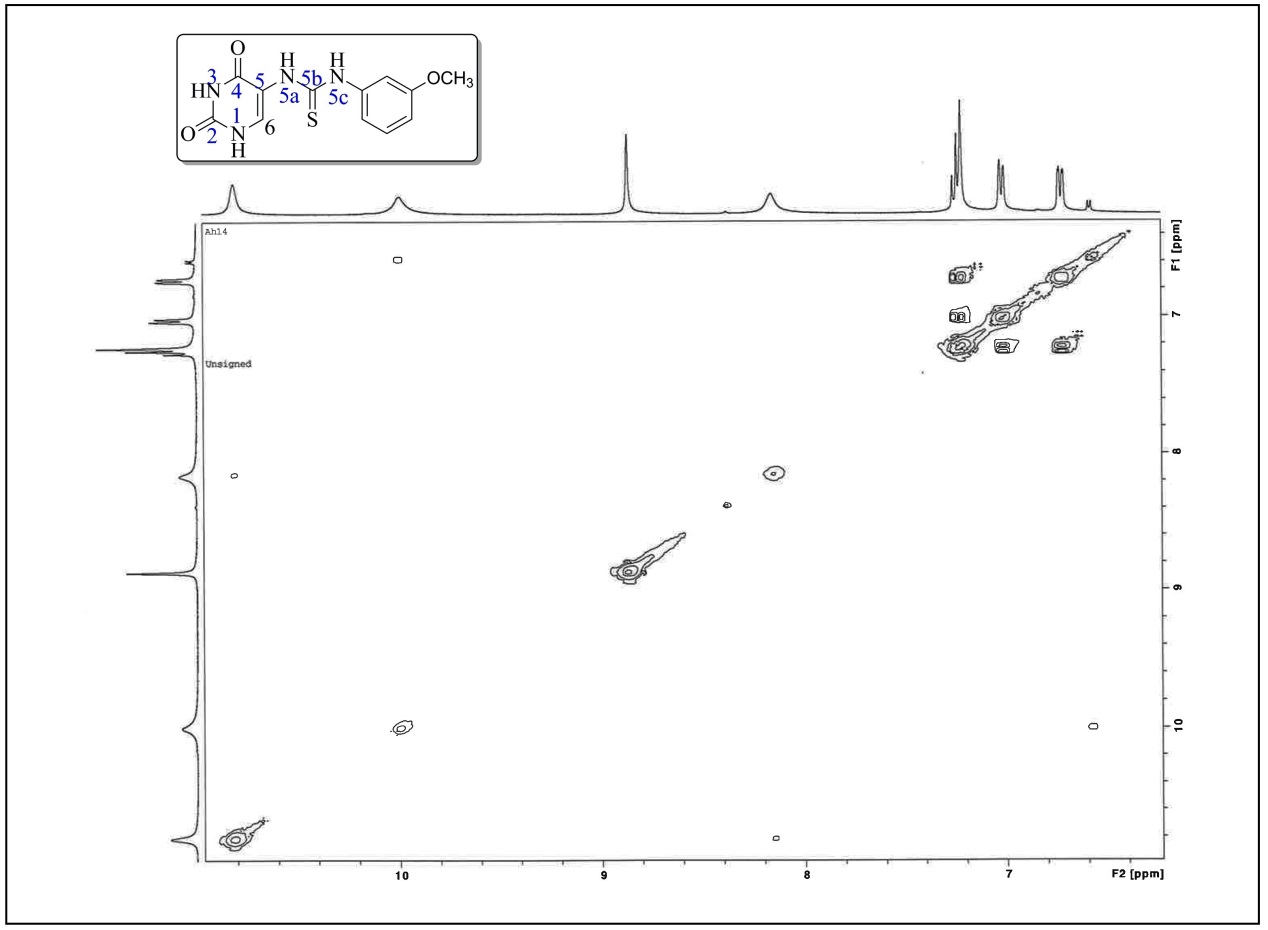


SI figure 36: ^1^H-^1^H COSY spectrum of **3c**


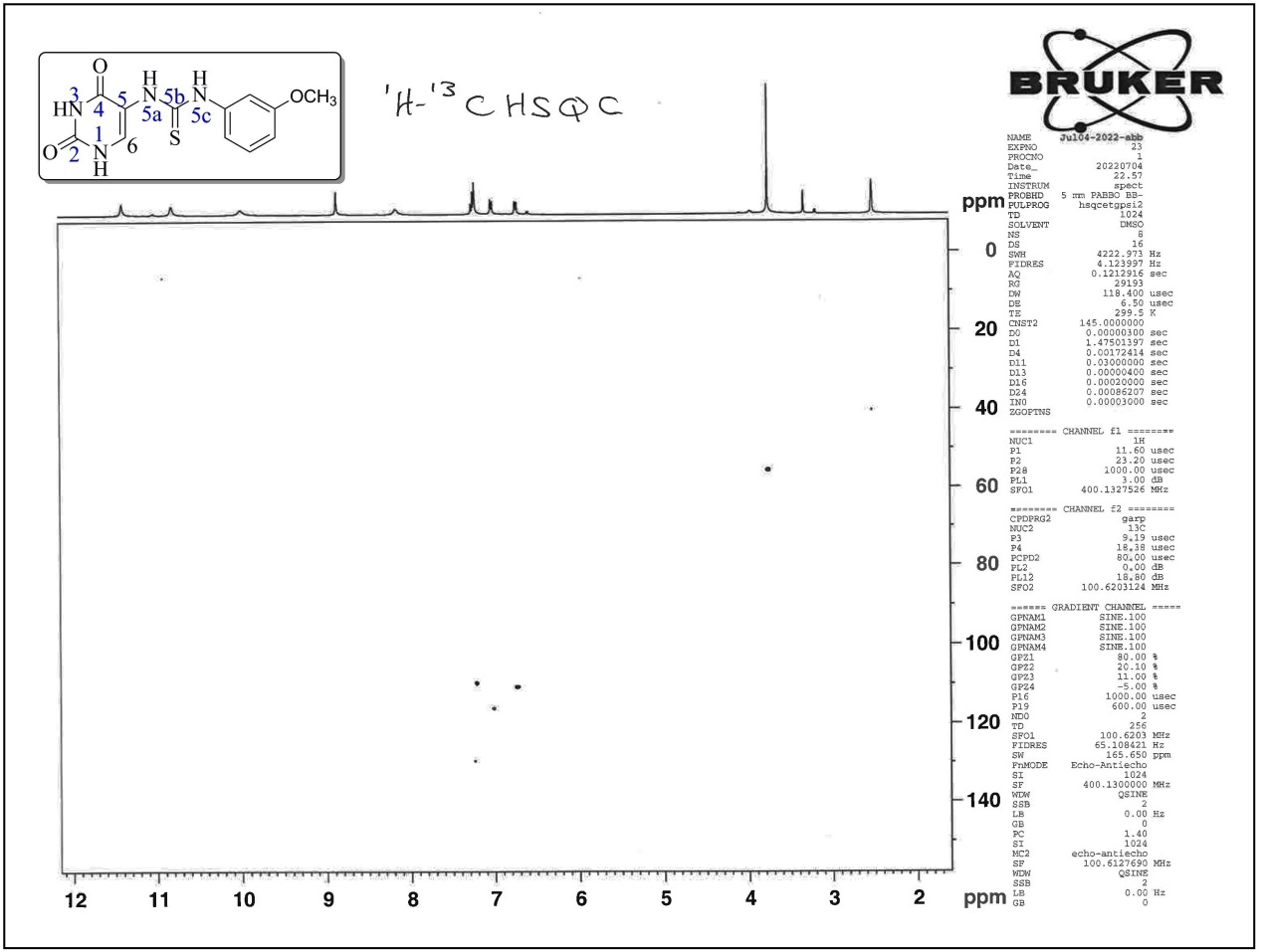


SI figure 37: ^1^H-^13^C HSQC spectrum of **3c**


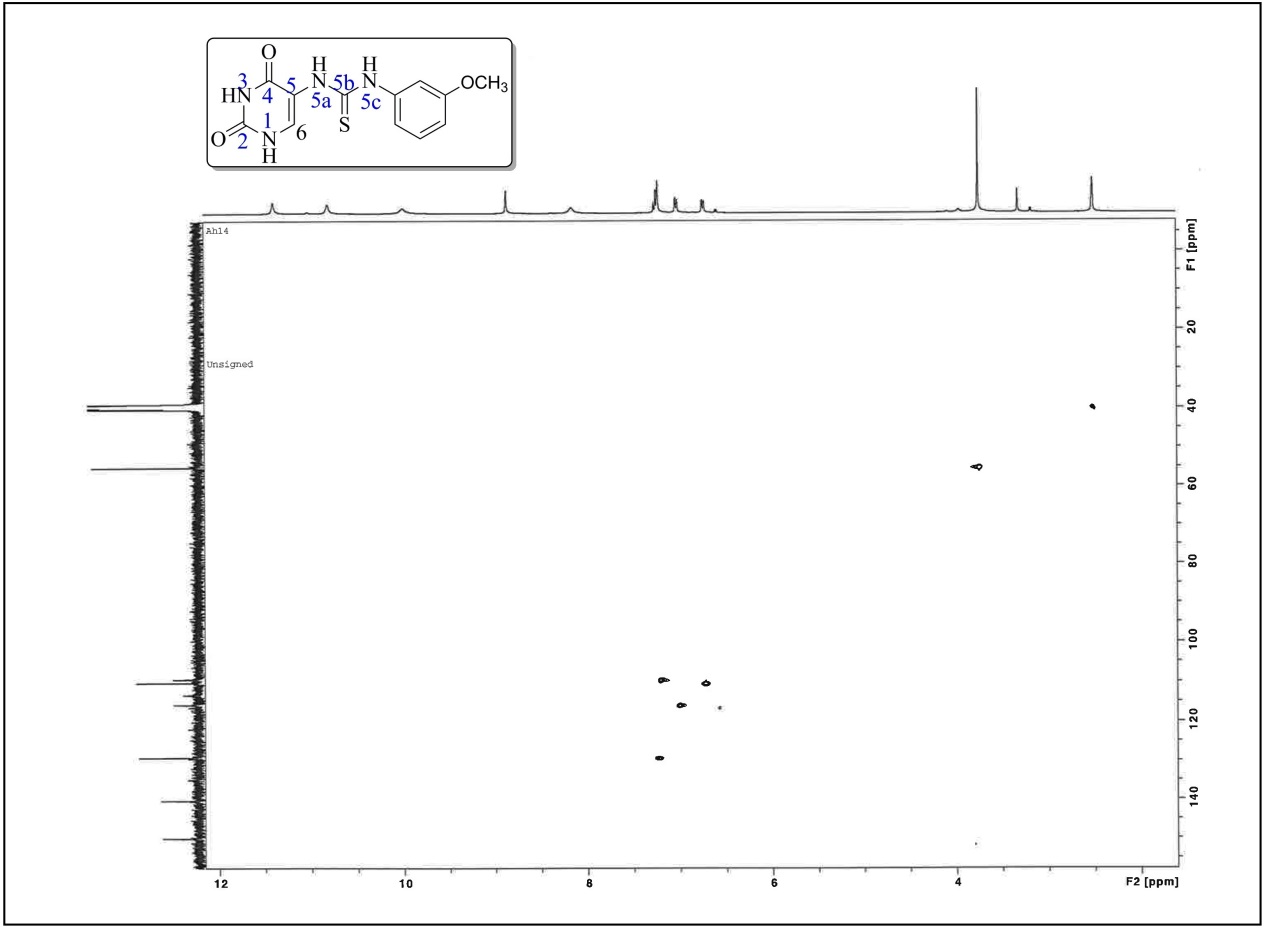


SI figure 38: ^1^H-^13^C HSQC spectrum of **3c**


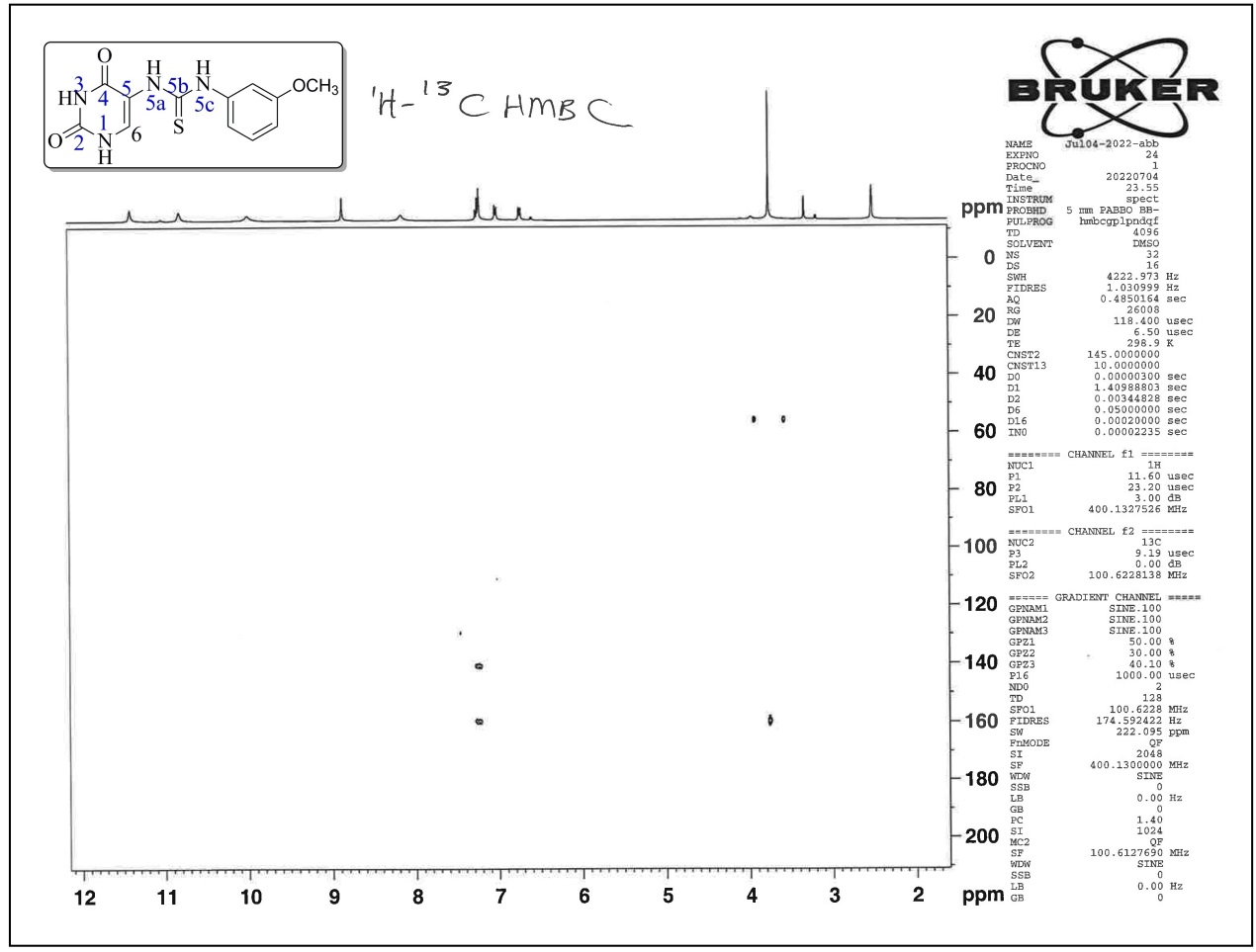


SI figure 39: ^1^H-^13^C-HMBC spectrum of **3c**


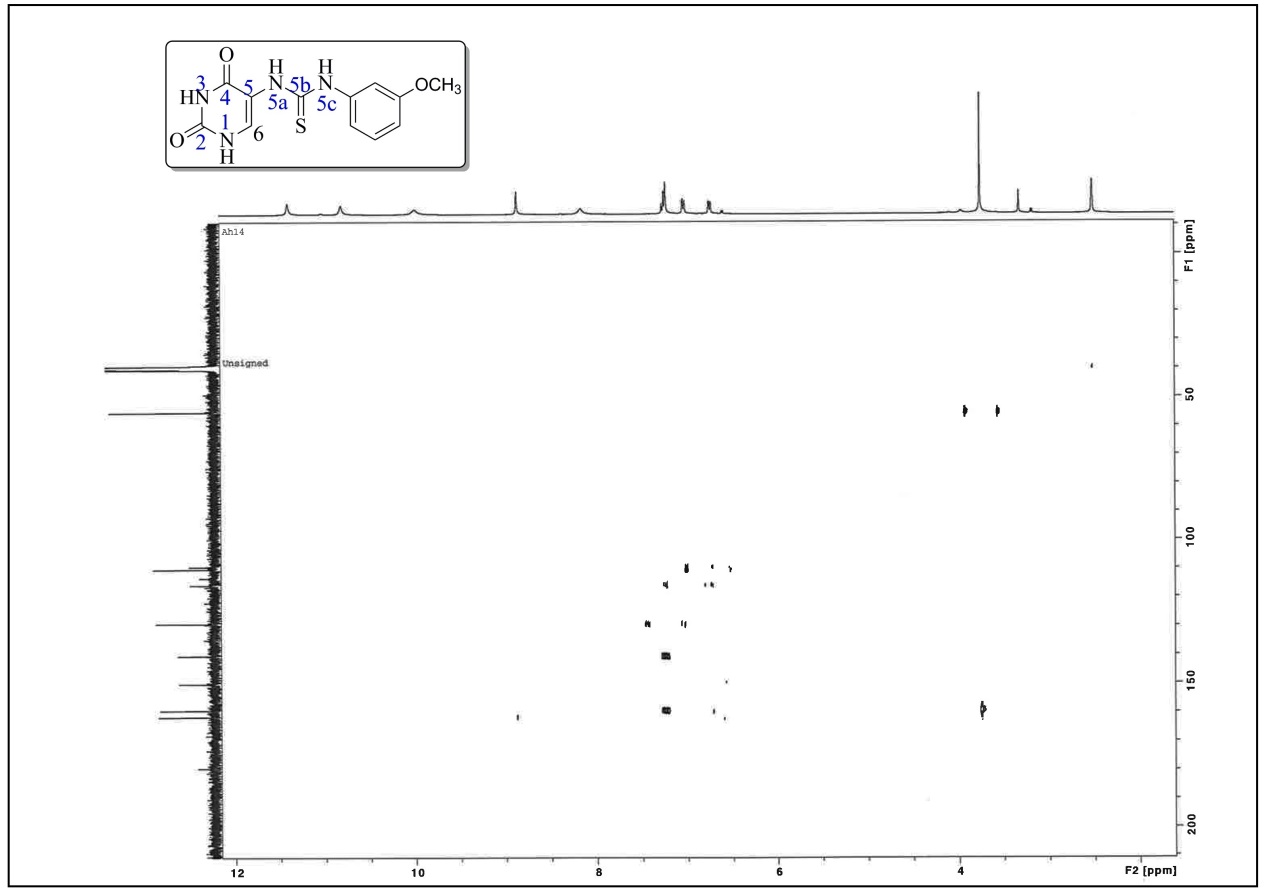


SI figure 40: ^1^H-^13^C-HMBC spectrum of **3c**


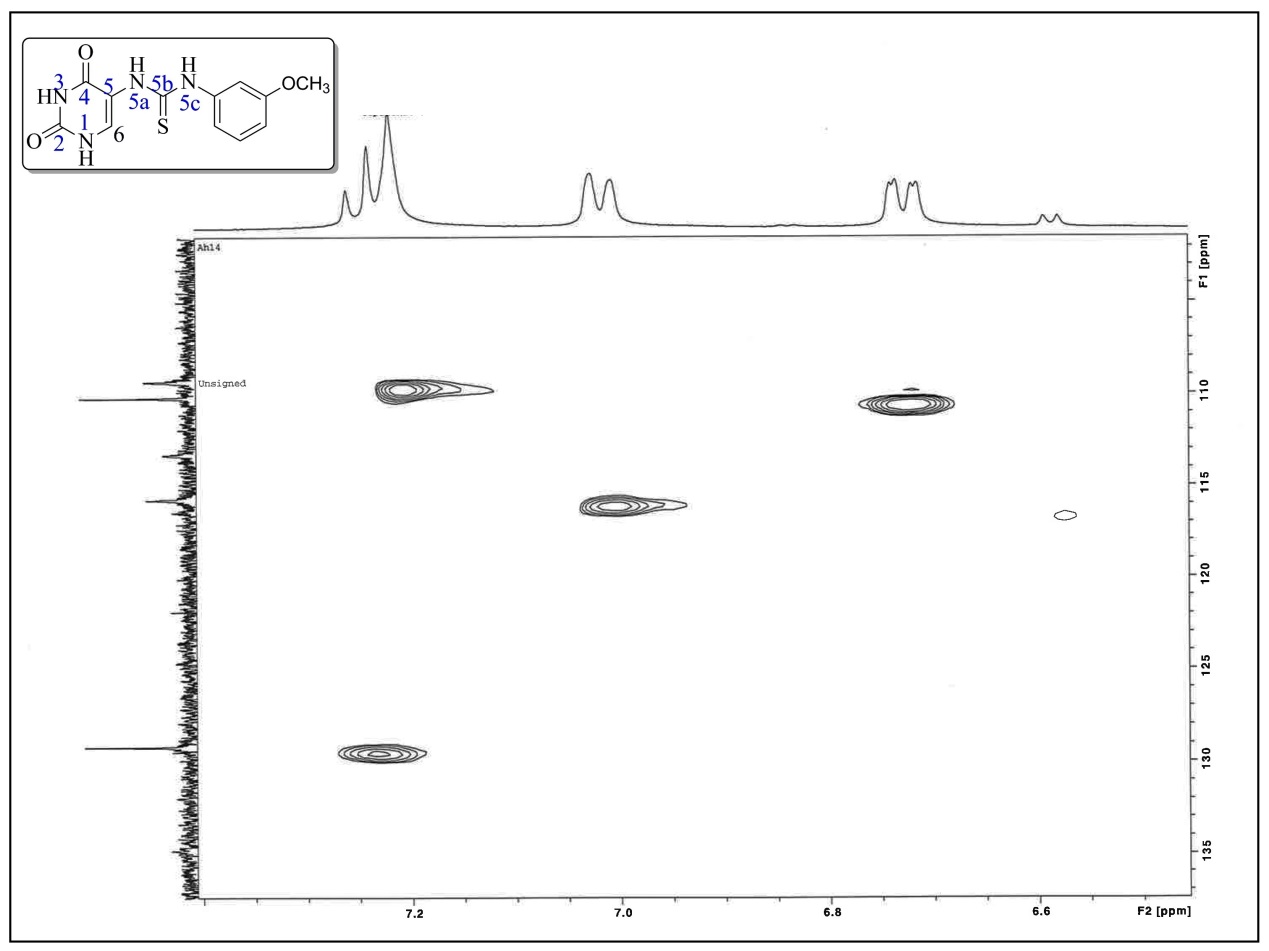


SI figure 41: ^1^H-^13^C-HMBC spectrum of **3c**


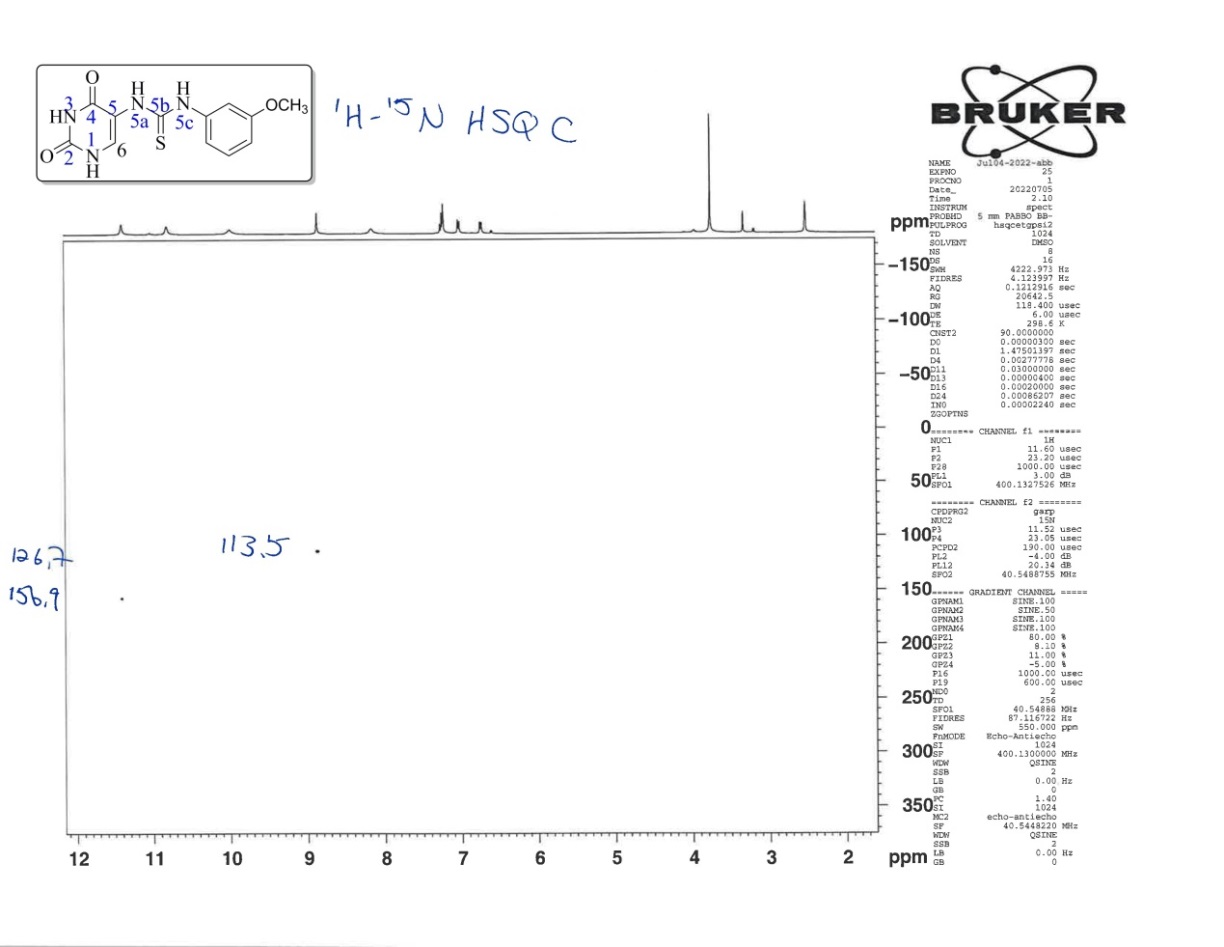


SI figure 42: ^1^H-^15^N-HSQC spectrum of **3c**


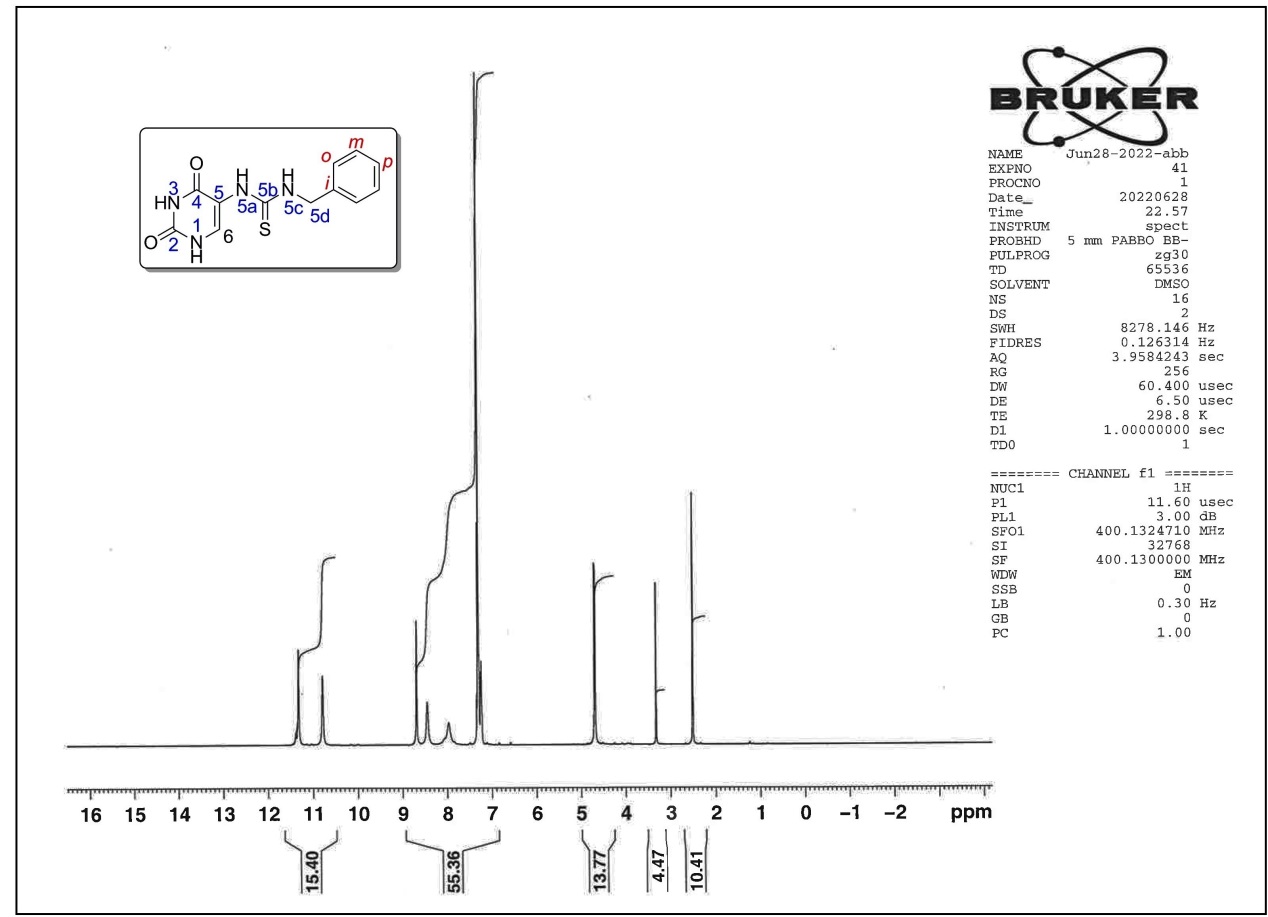


SI figure 43: ^1^H-^1^H-NMR spectrum of **3d**


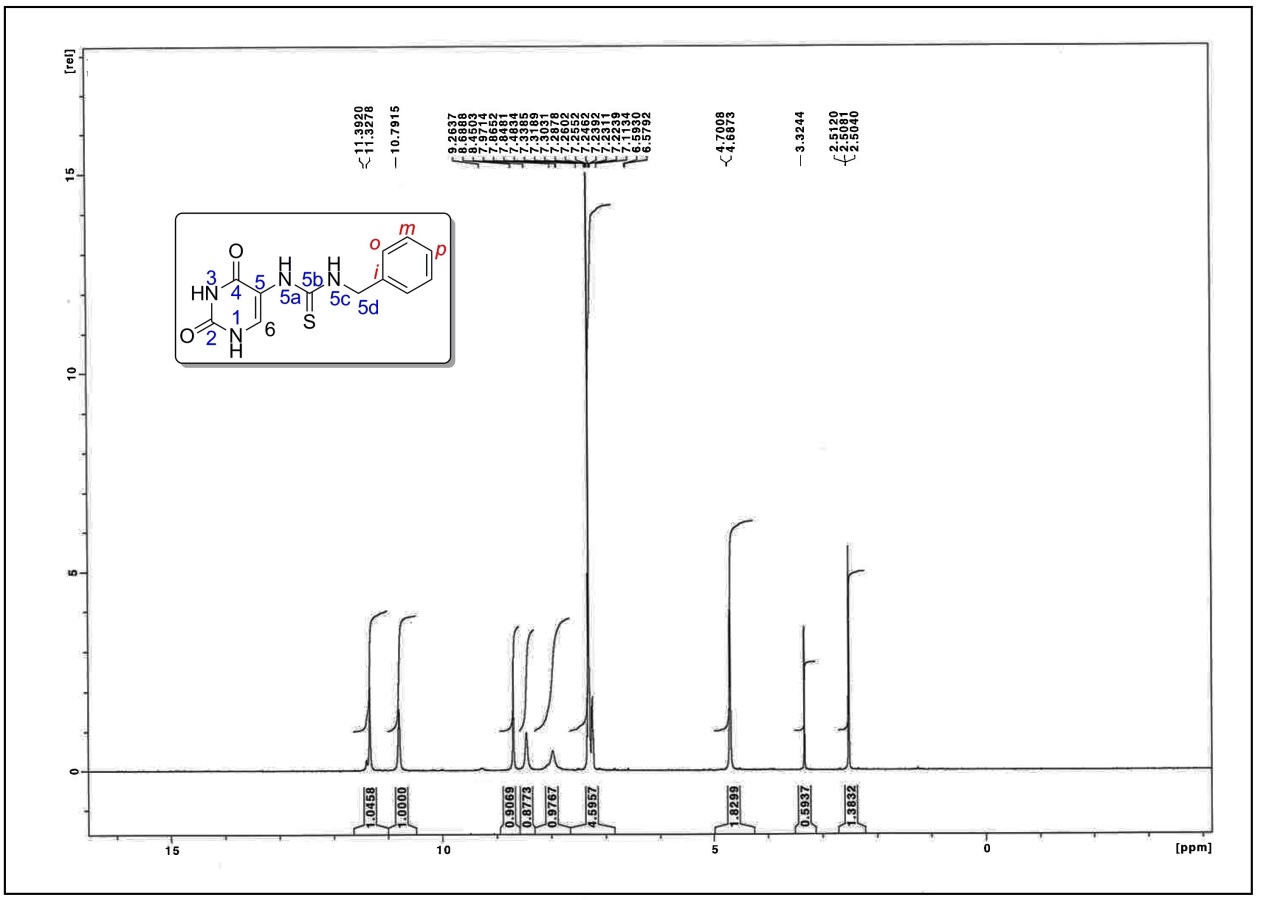


SI figure 44: ^1^H-^1^H-NMR spectrum of **3d**


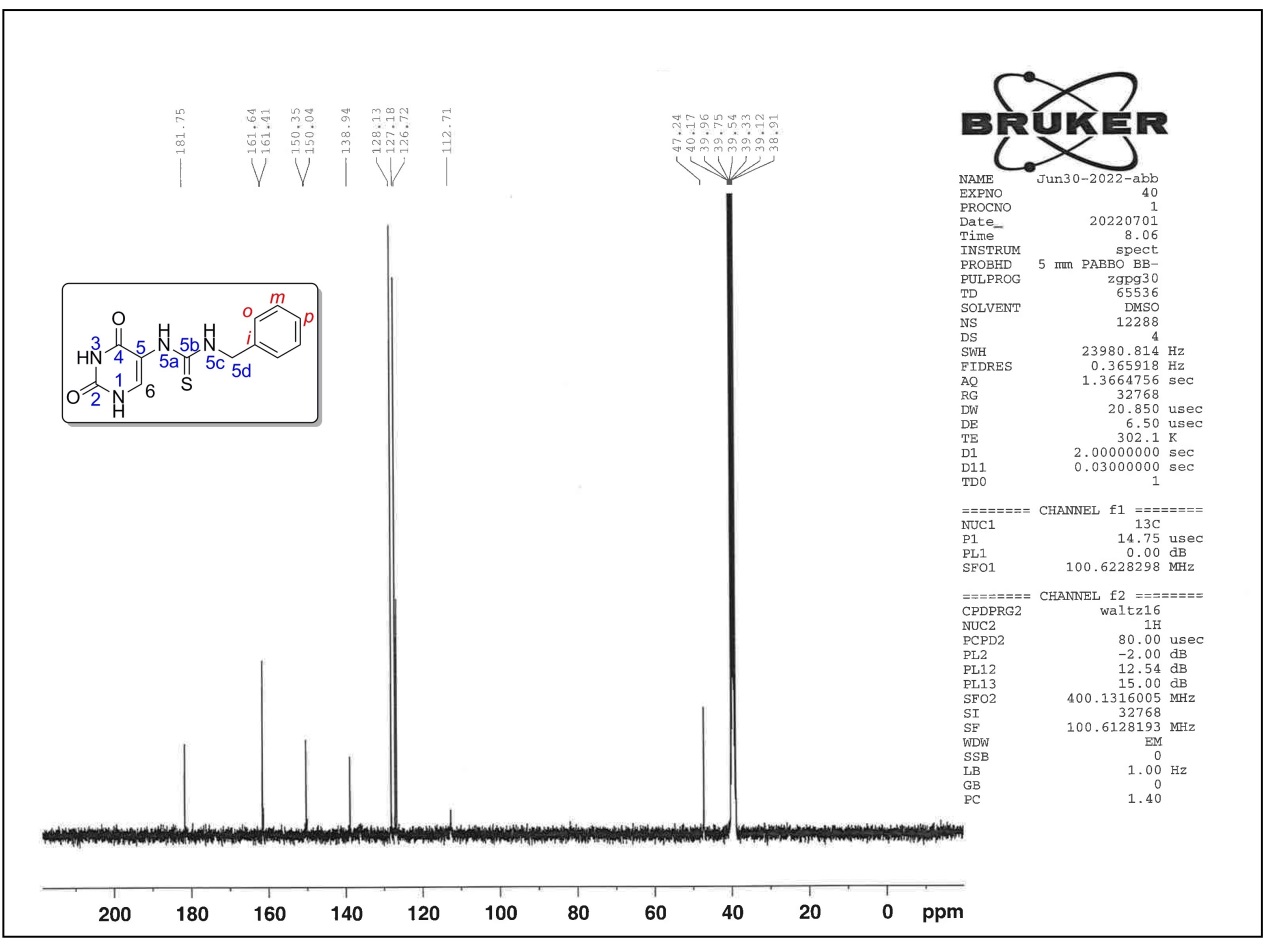


SI figure 45: ^13^C-NMR Spectrum of **3d**


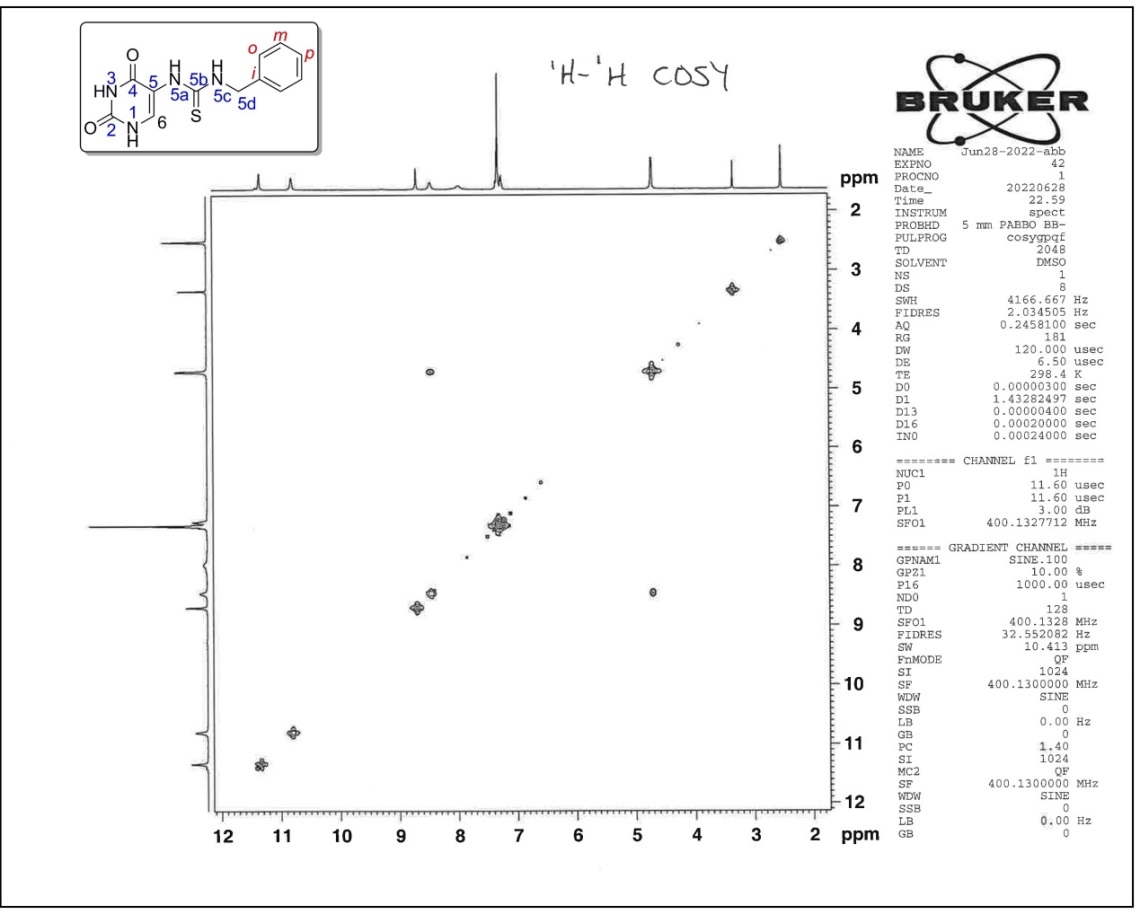


SI figure 46: ^1^H-^1^H COSY spectrum of **3d**


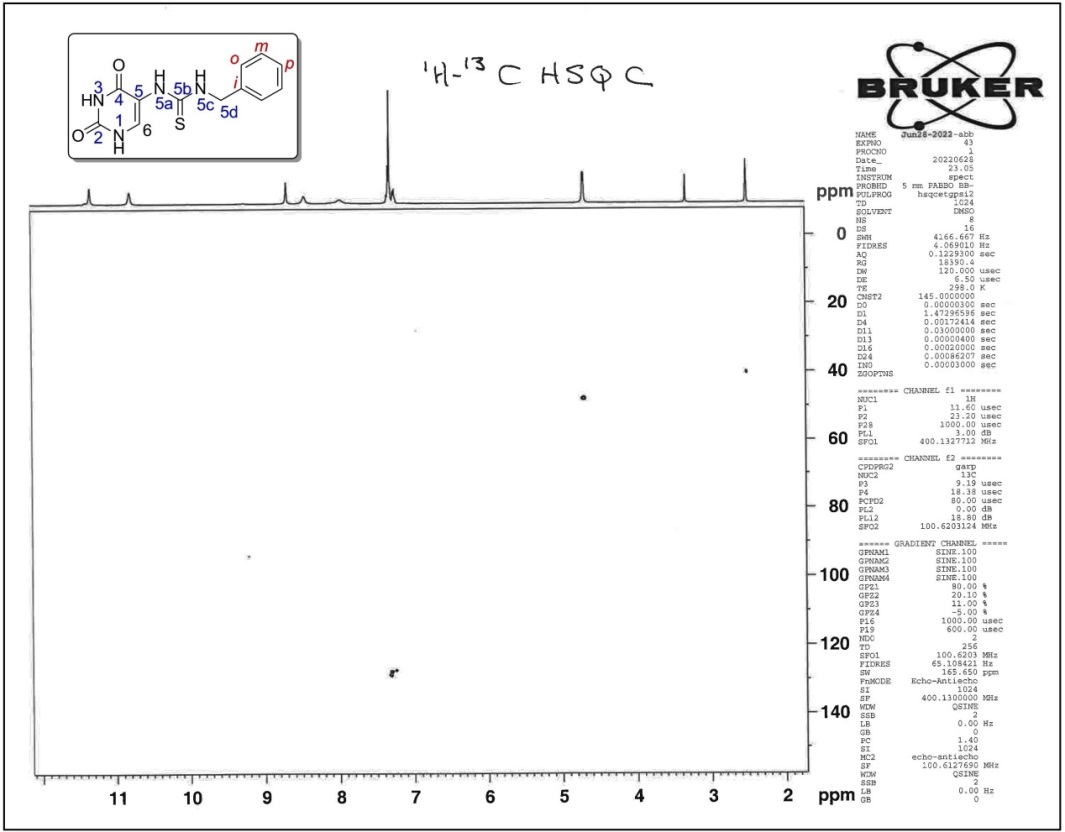


SI figure 47: ^1^H-^13^C HSQC spectrum of **3d**


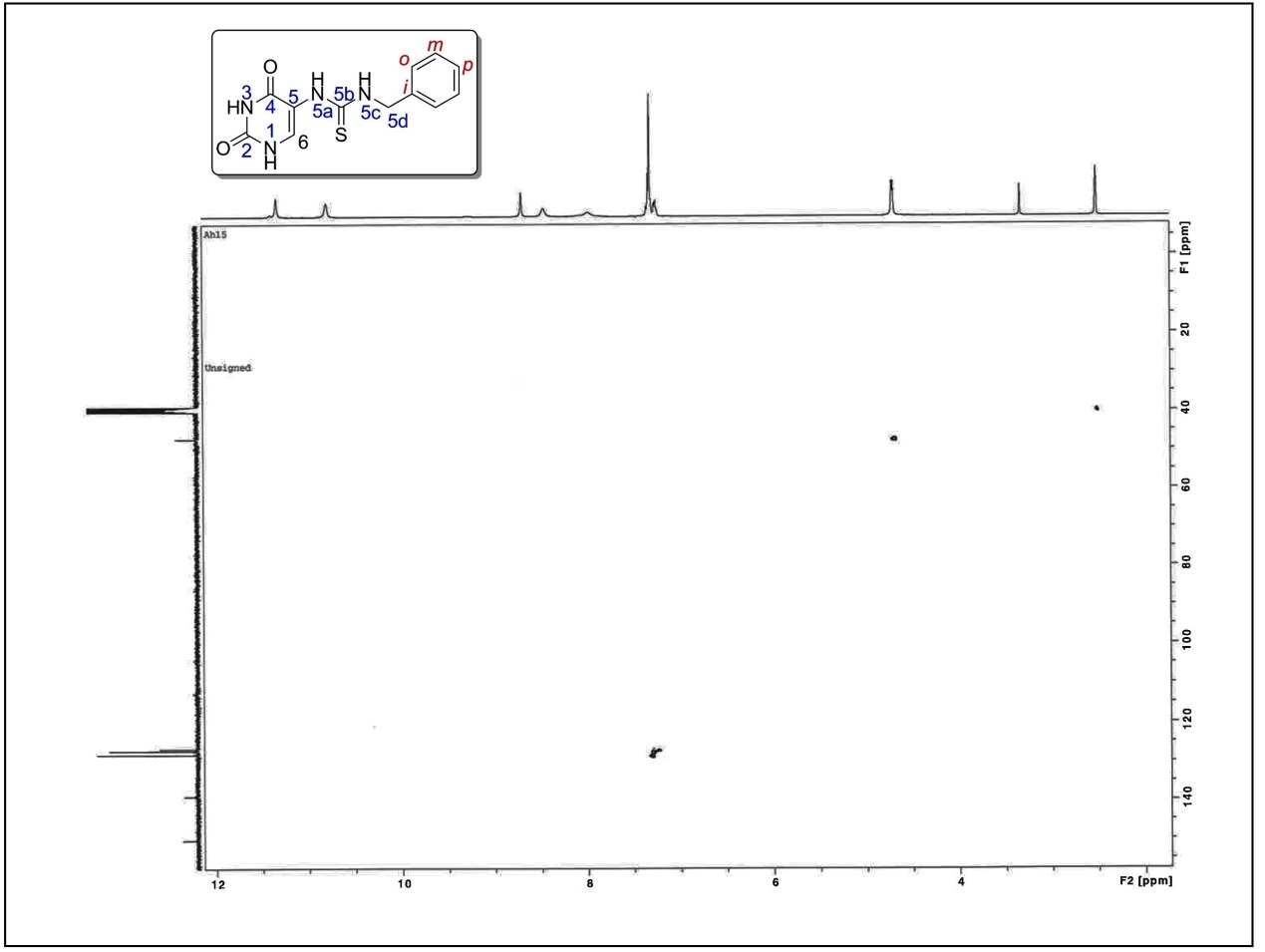


SI figure 48: ^1^H-^13^C HSQC spectrum of **3d**


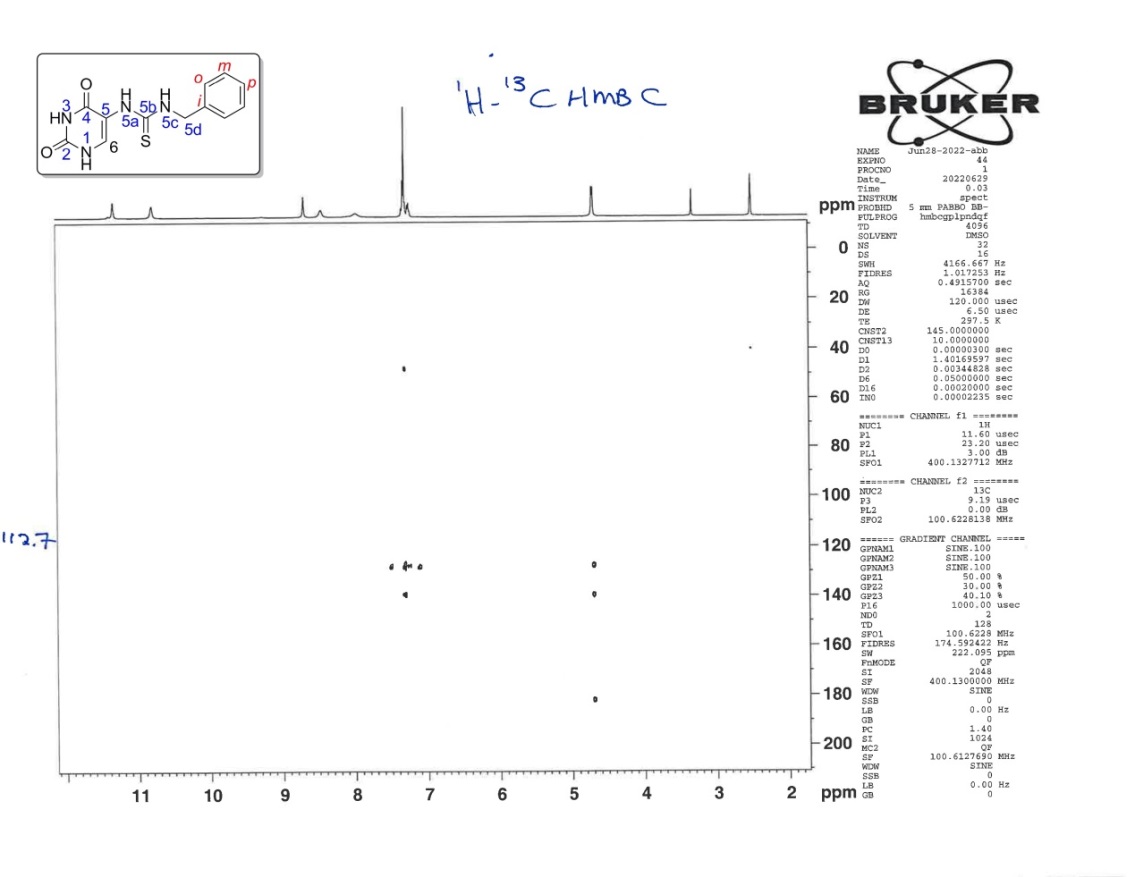


SI figure 49: ^1^H-^13^C HMBC spectrum of **3d**


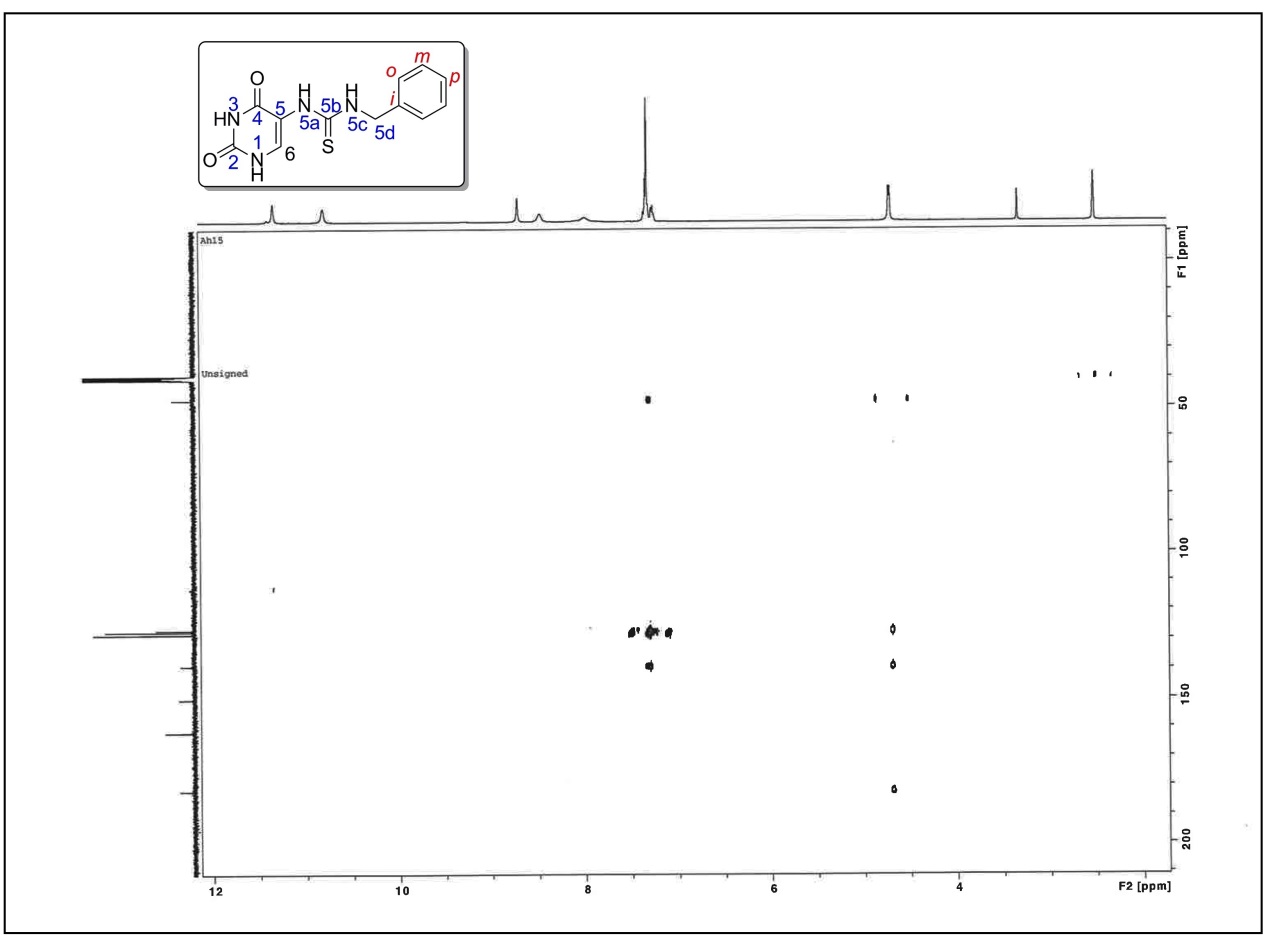


SI figure 50: ^1^H-^13^C HMBC spectrum of **3d**


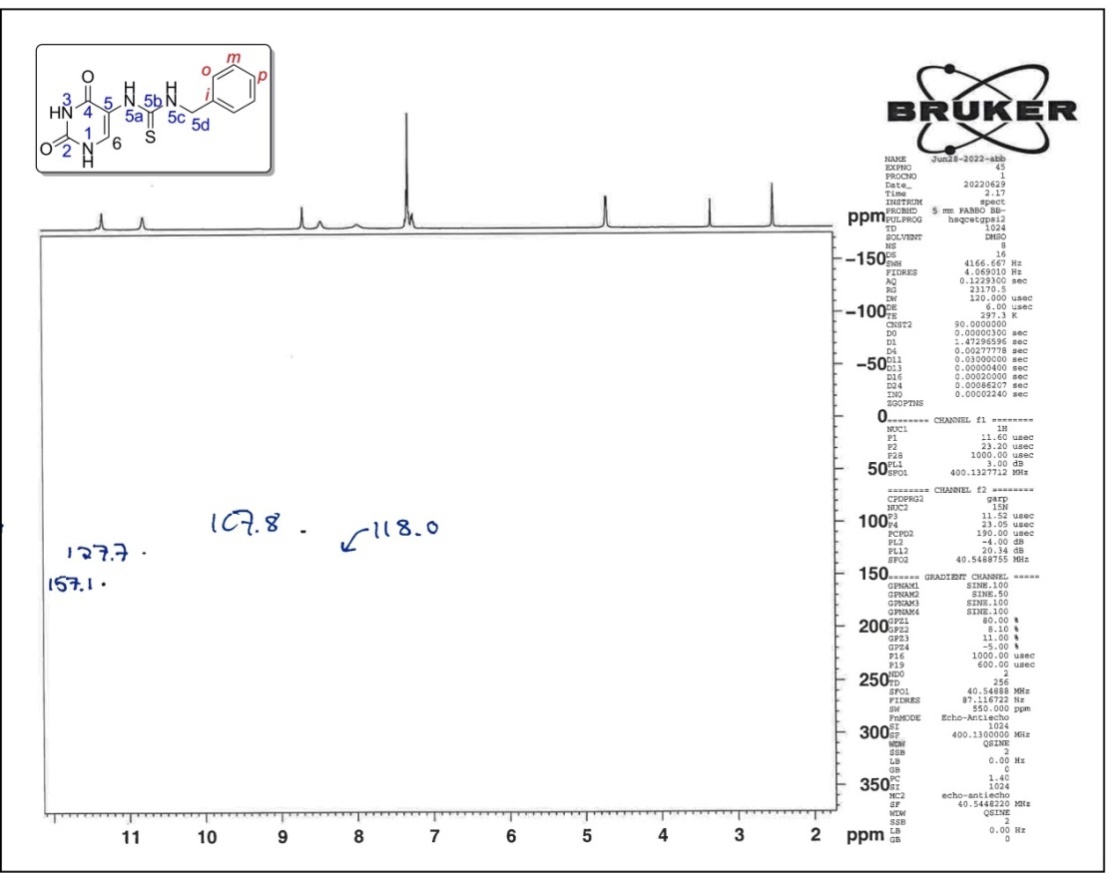


SI figure 51: ^1^H-^13^C HMBC spectrum of **3d**


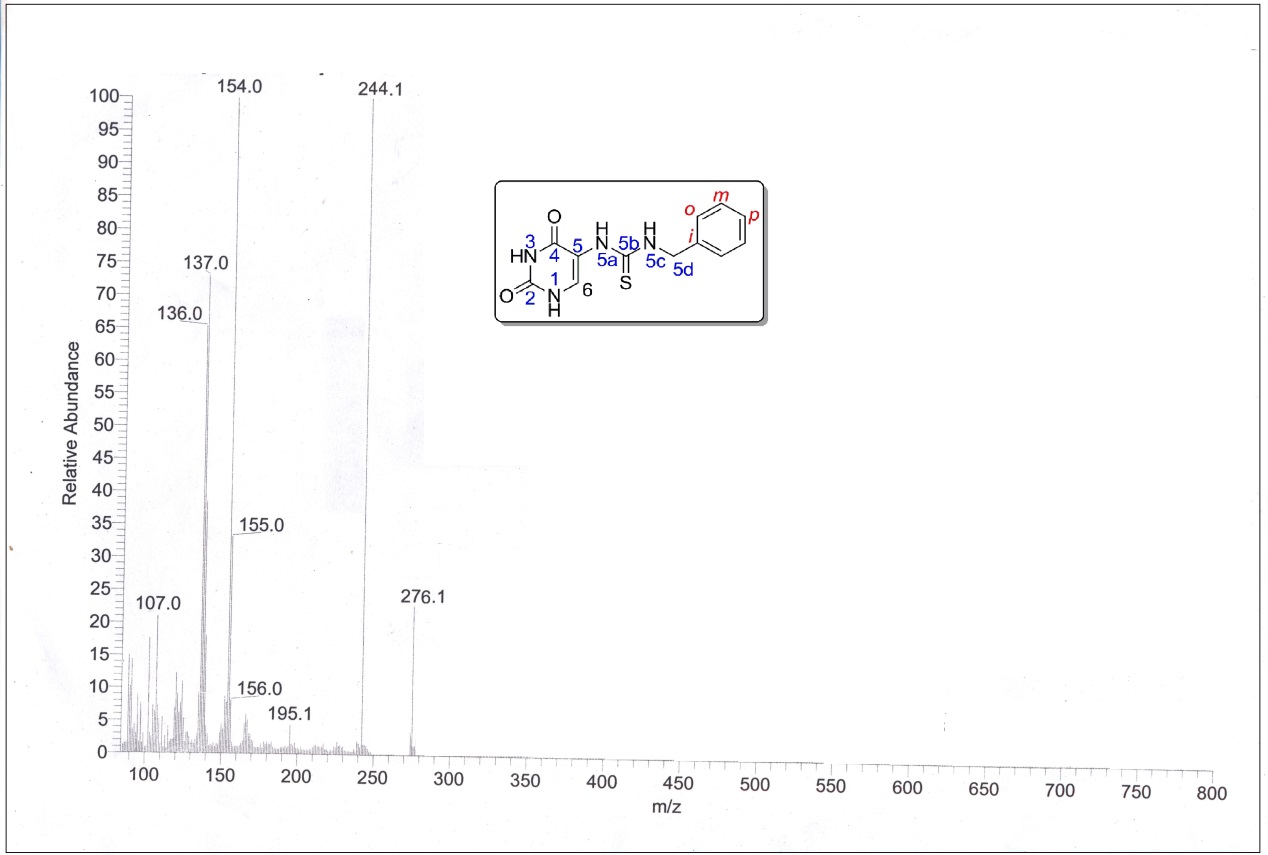


SI figure 52: Mass Spectroscopy spectrum of **3d**


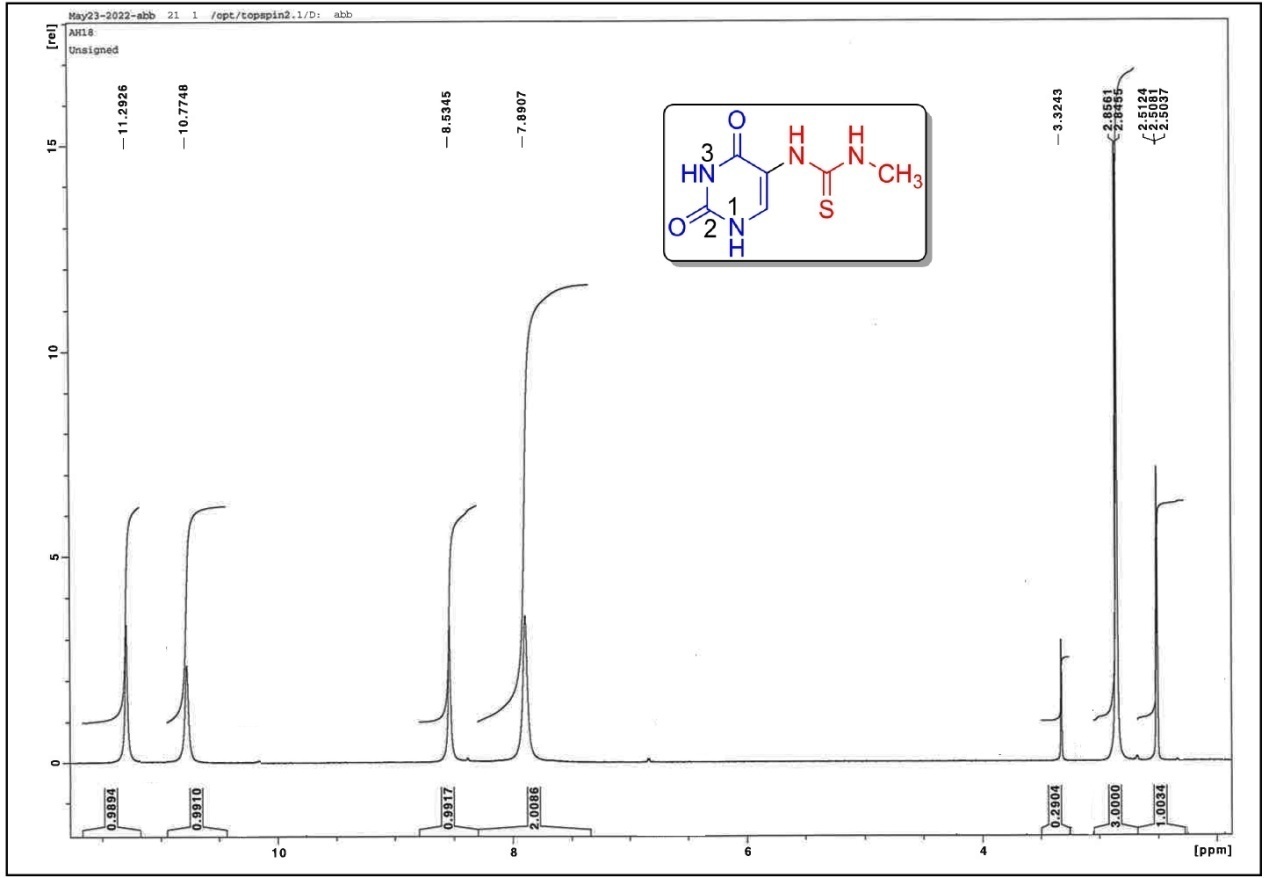


SI figure 53: ^1^H-NMR spectrum of **3e**


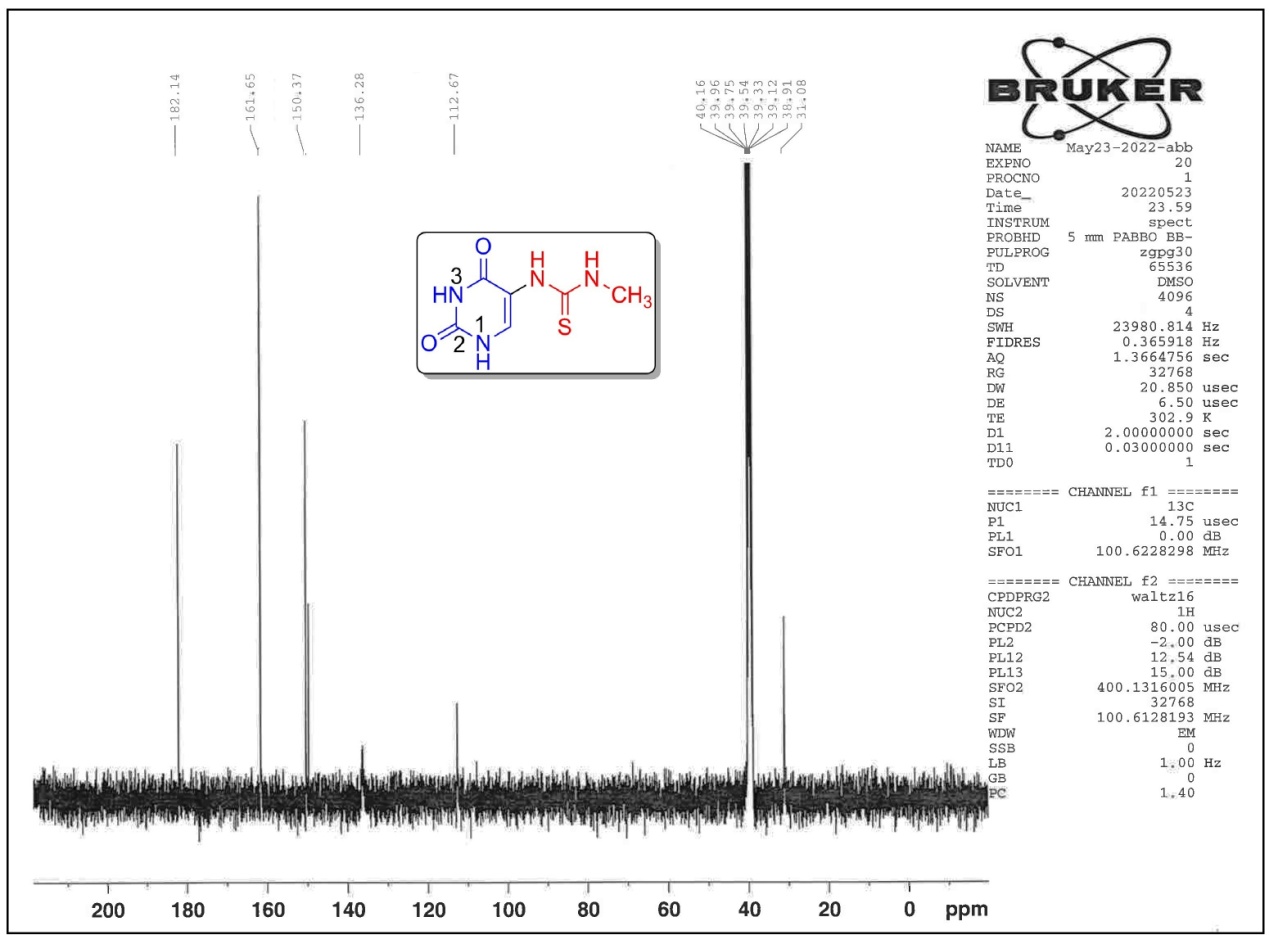


SI figure 54: ^13^C-NMR spectrum of **3e**


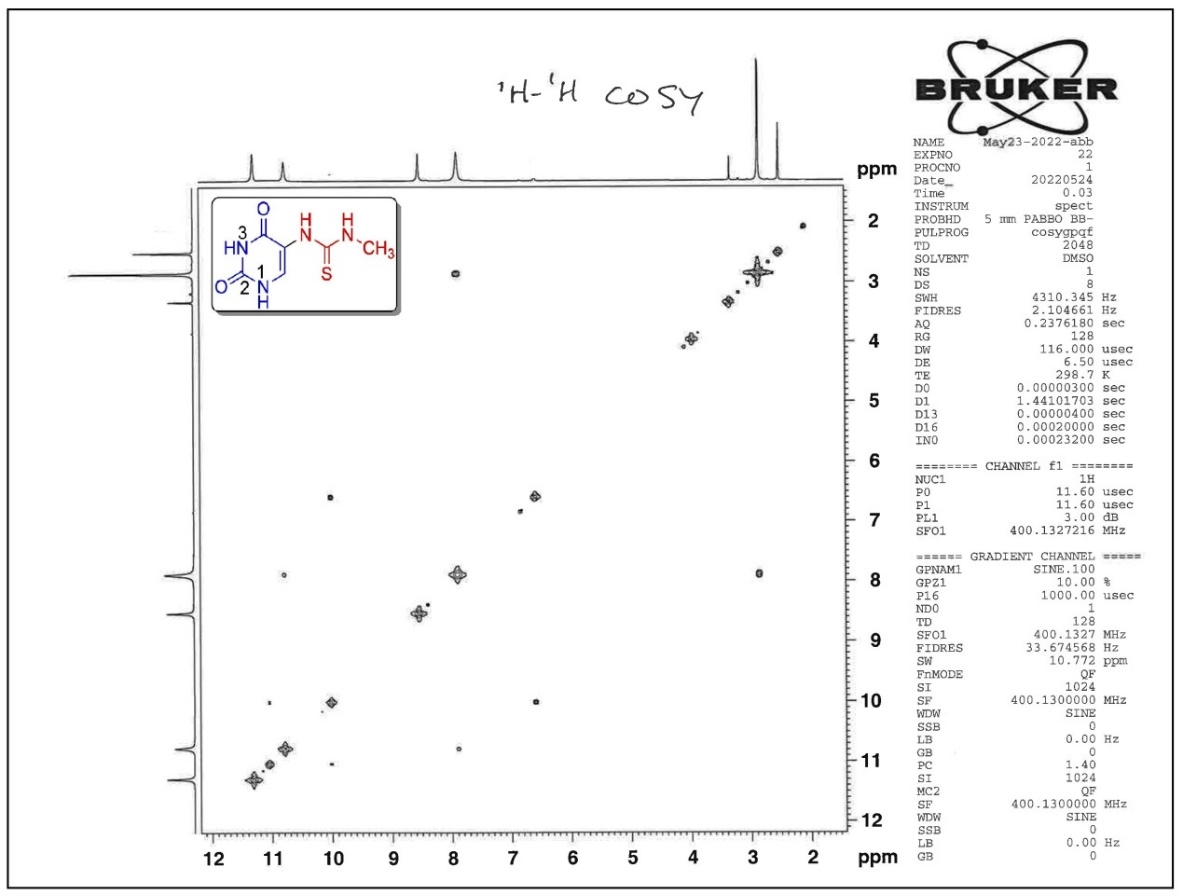


SI figure 55: ^1^H-^1^H COSY spectrum of **3e**


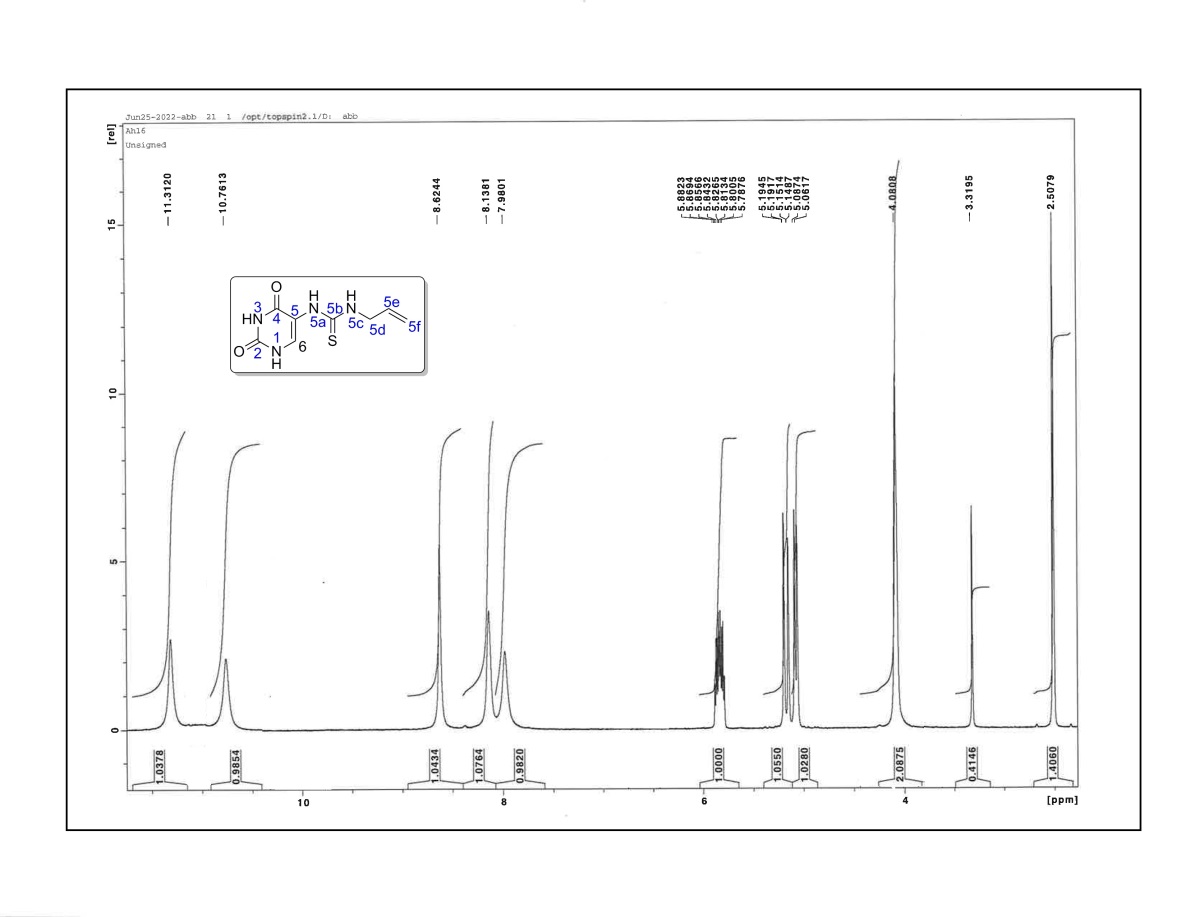


SI figure 56: ^1^H-NMR spectrum of **3f**


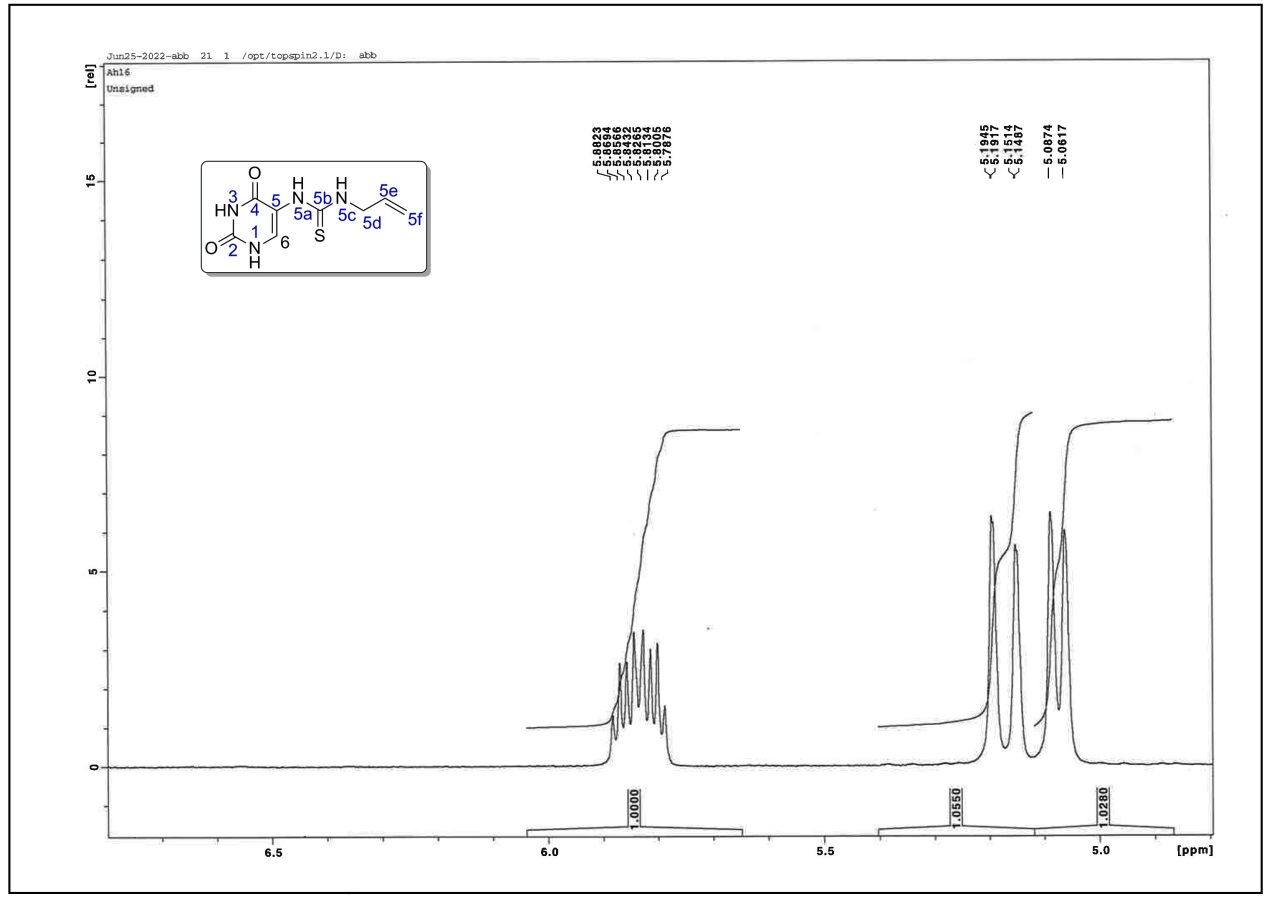


SI figure 57: ^1^H-NMR spectrum of **3f**


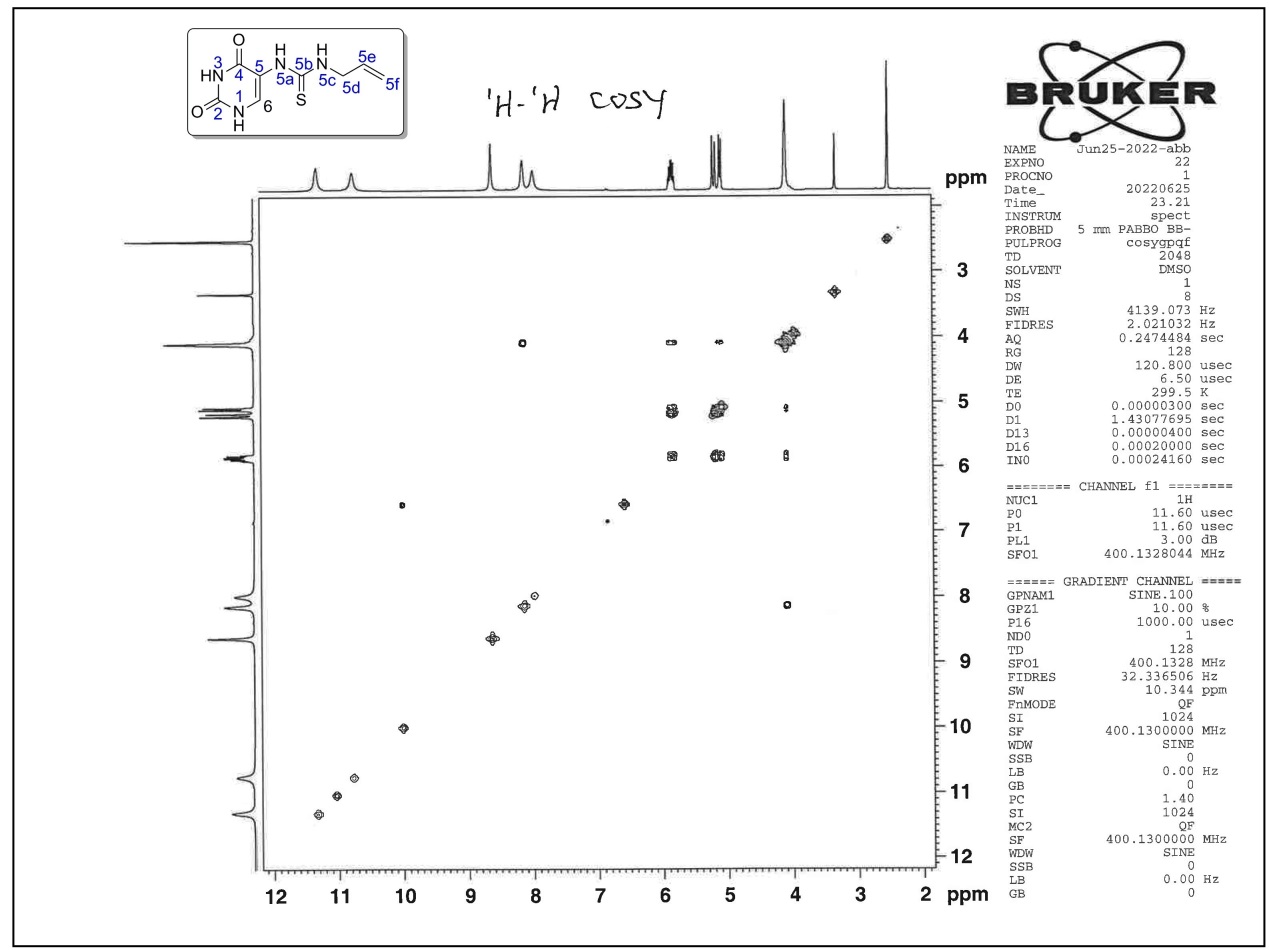


SI figure 58: ^1^H-^1^H COSY spectrum of **3f**


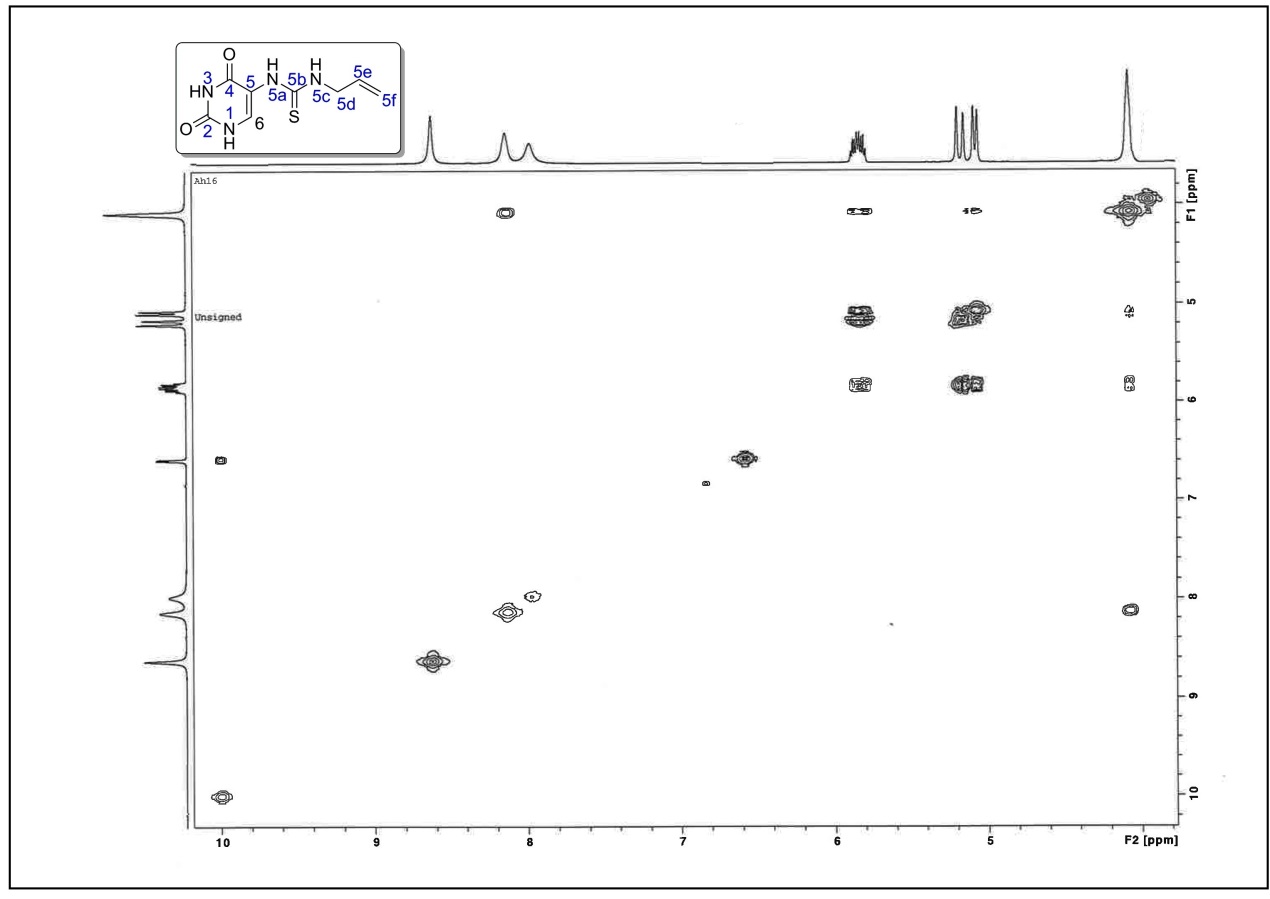


SI figure 58: ^1^H-^1^H COSY spectrum of **3f**


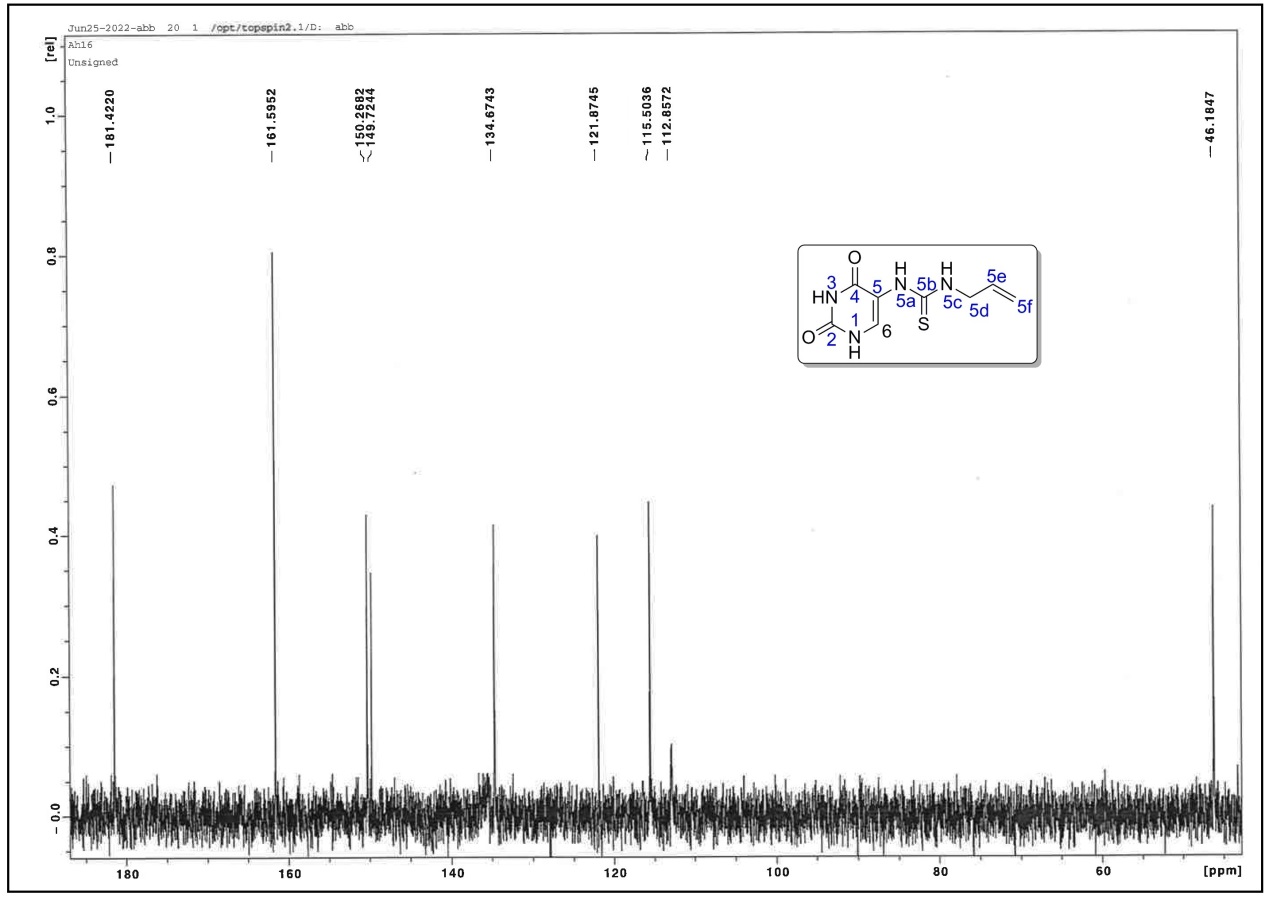


SI figure 59: ^13^C-NMR spectrum of **3f**


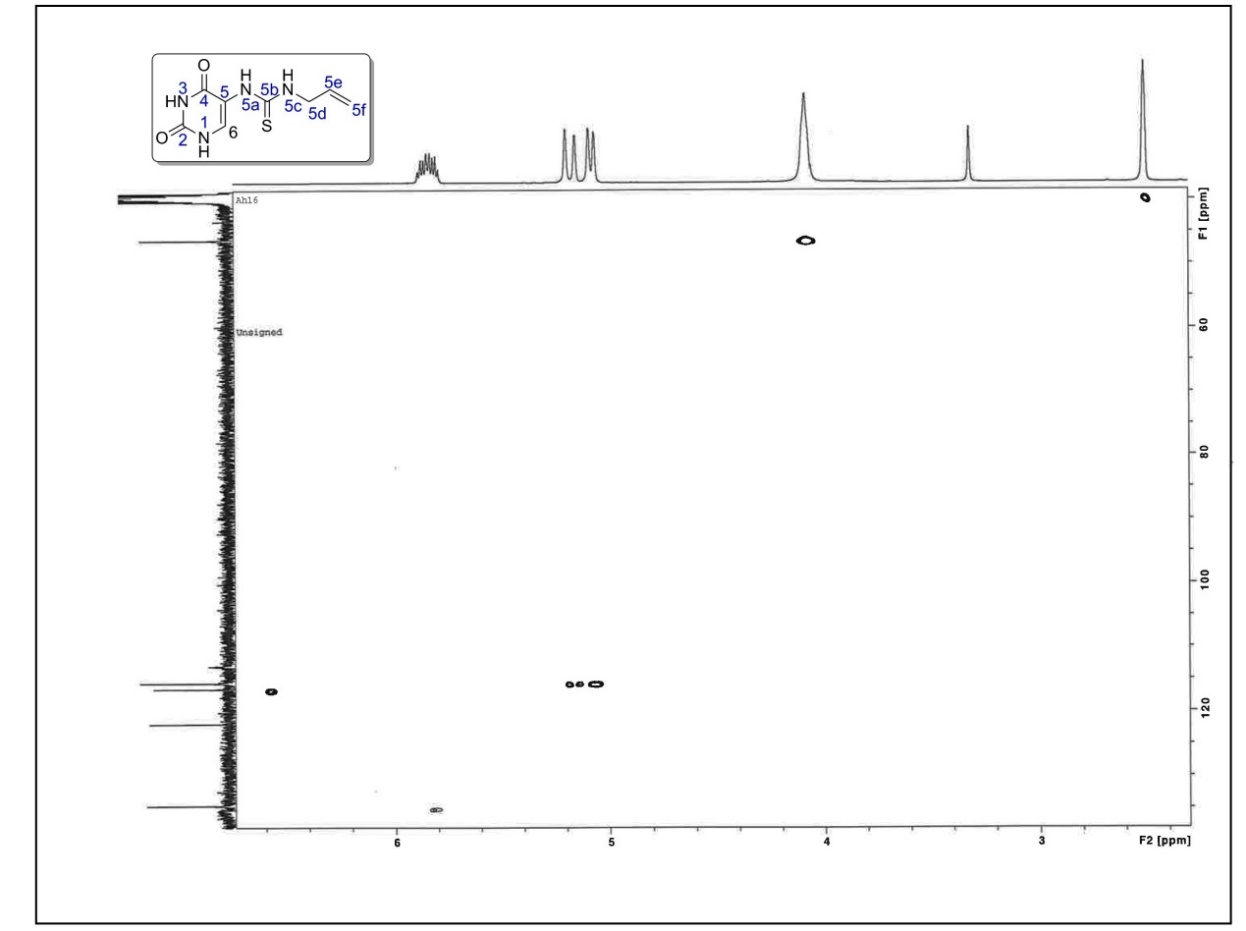


SI figure 60: ^1^H-^13^C HSQC spectrum of **3f**


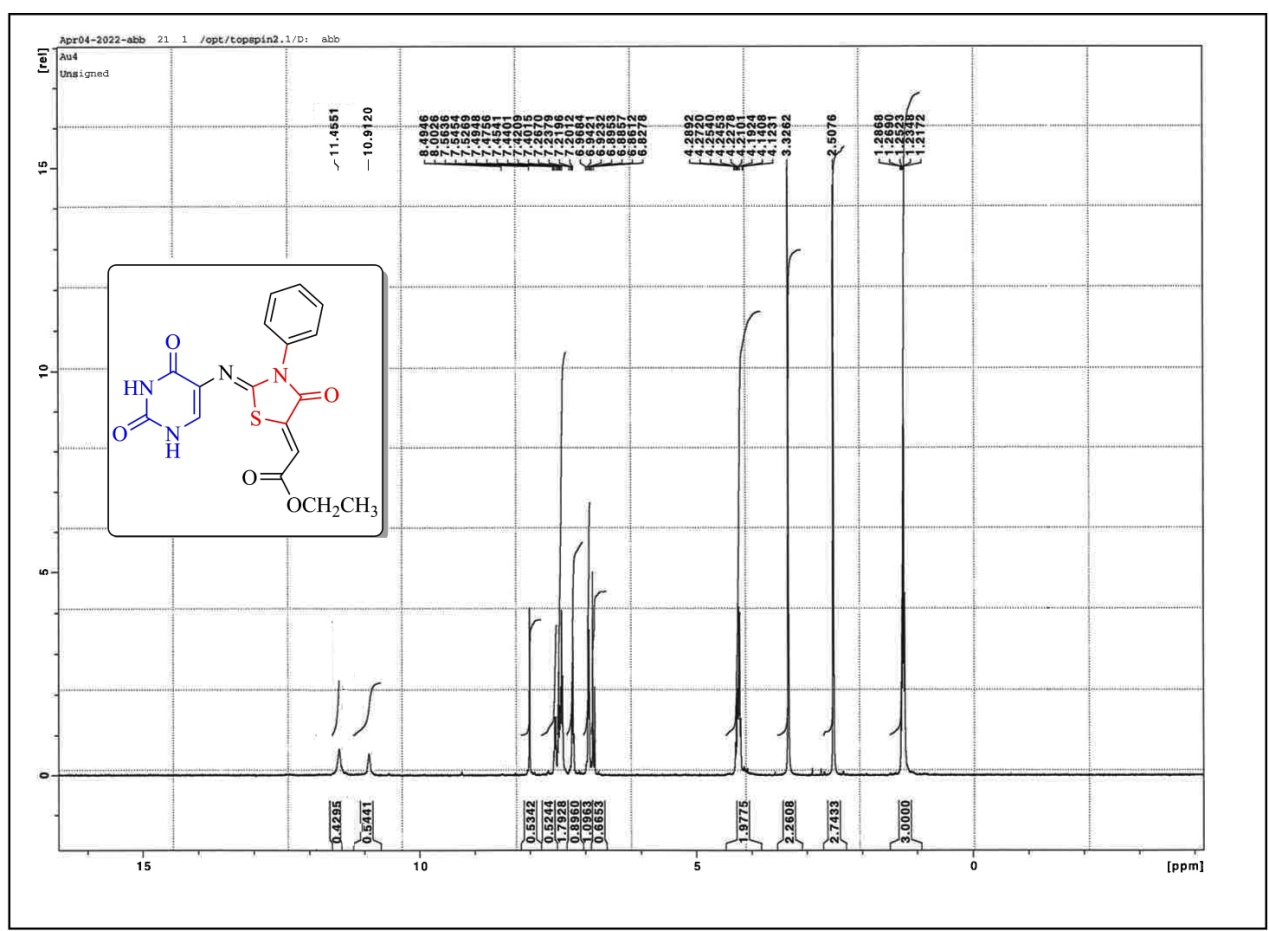


SI figure 61: ^1^H-NMR spectrum of **5a**


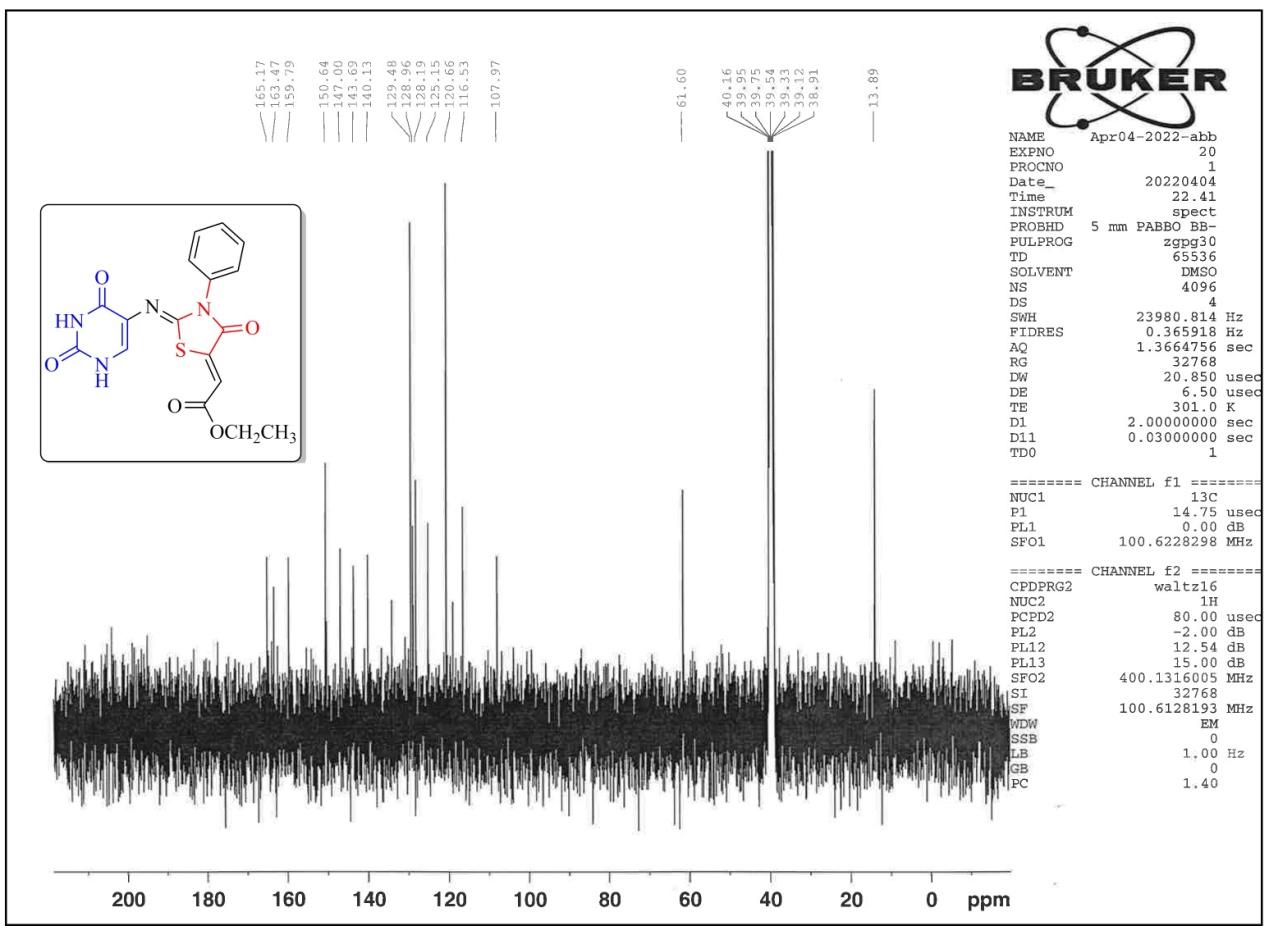


SI figure 62: ^13^C-NMR spectrum of **5a**


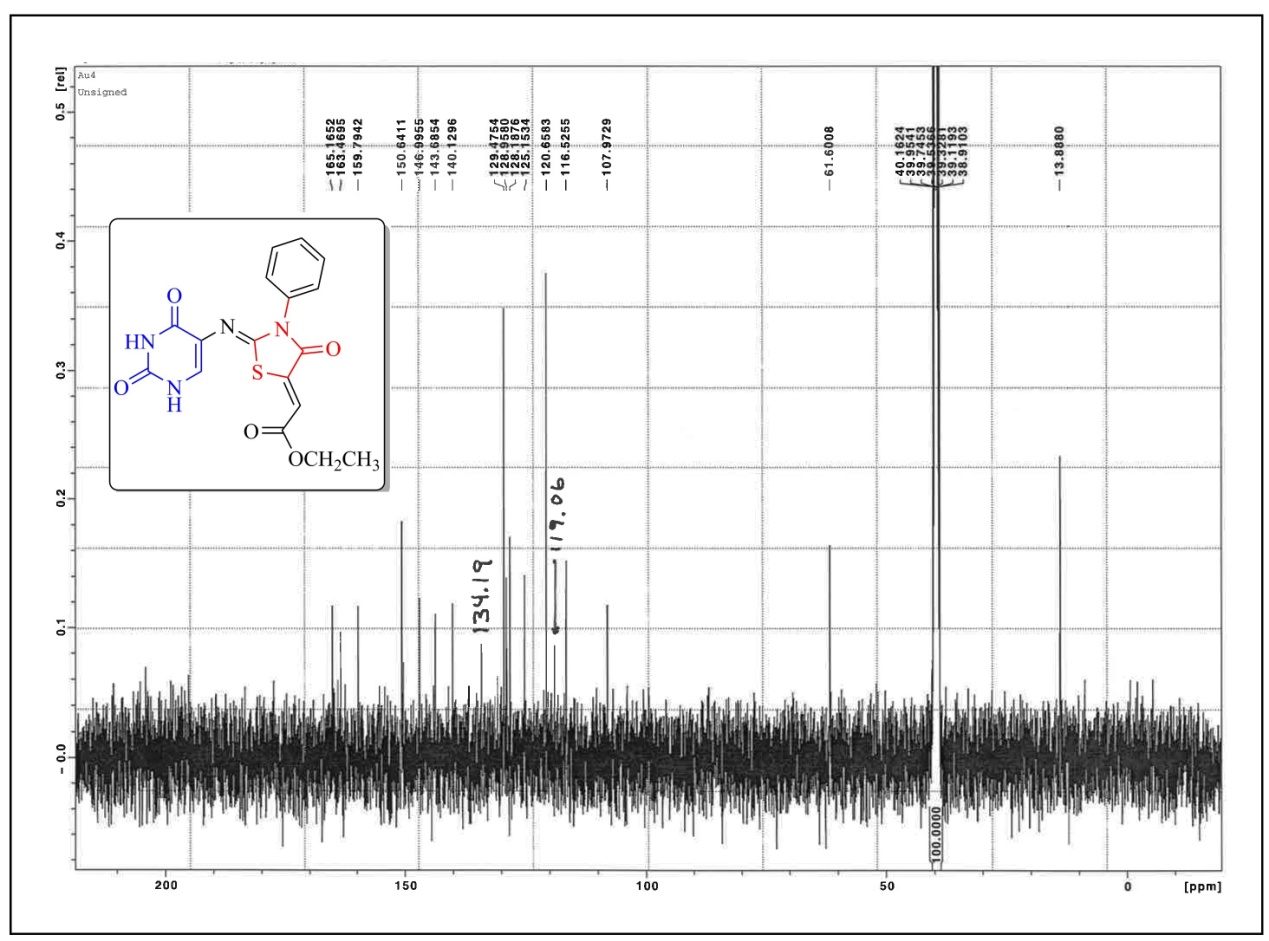


SI figure 63: ^13^C-NMR spectrum of **5a**


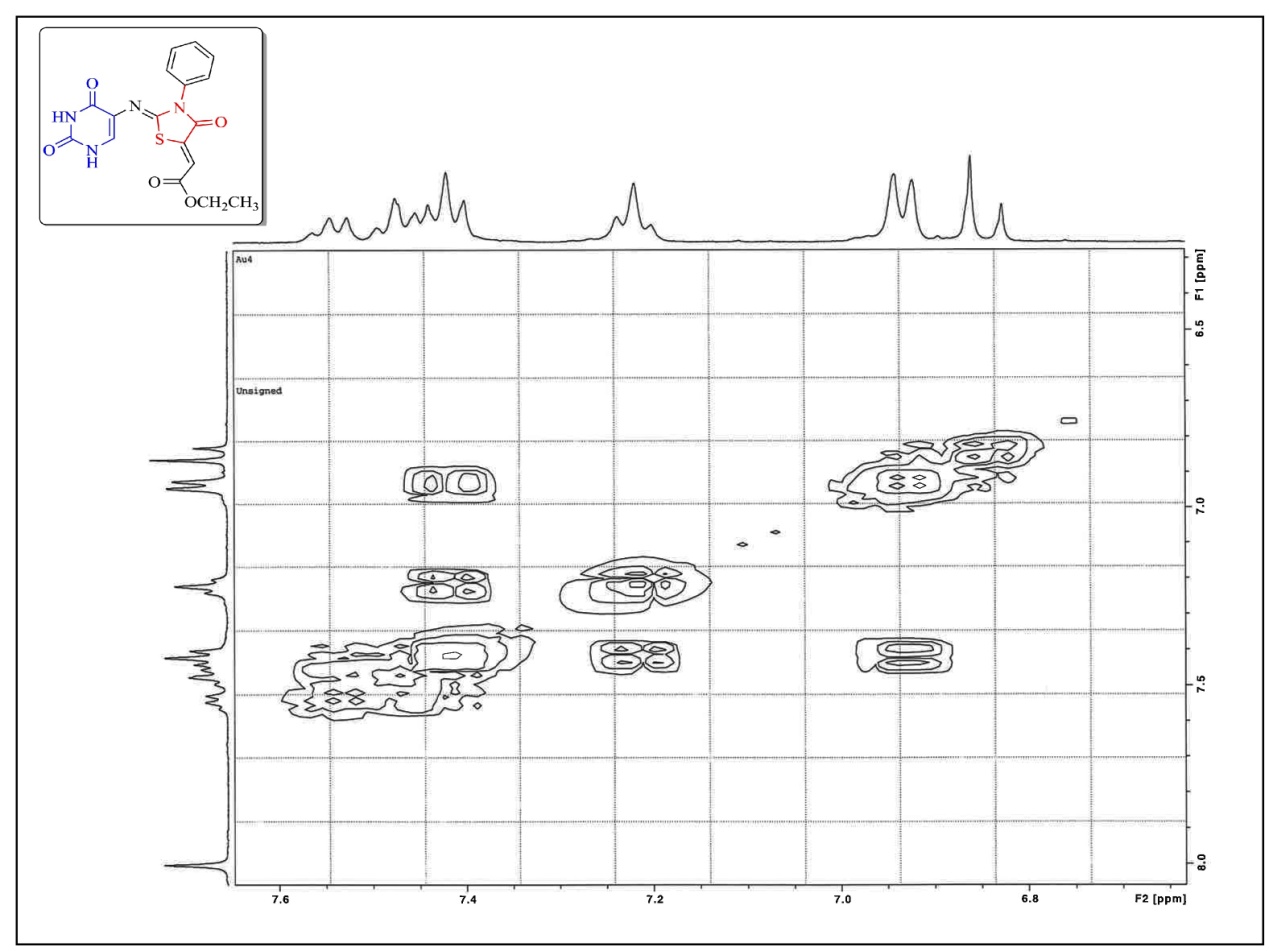


SI figure 64: ^1^H-^1^H COSY spectrum of **5a**


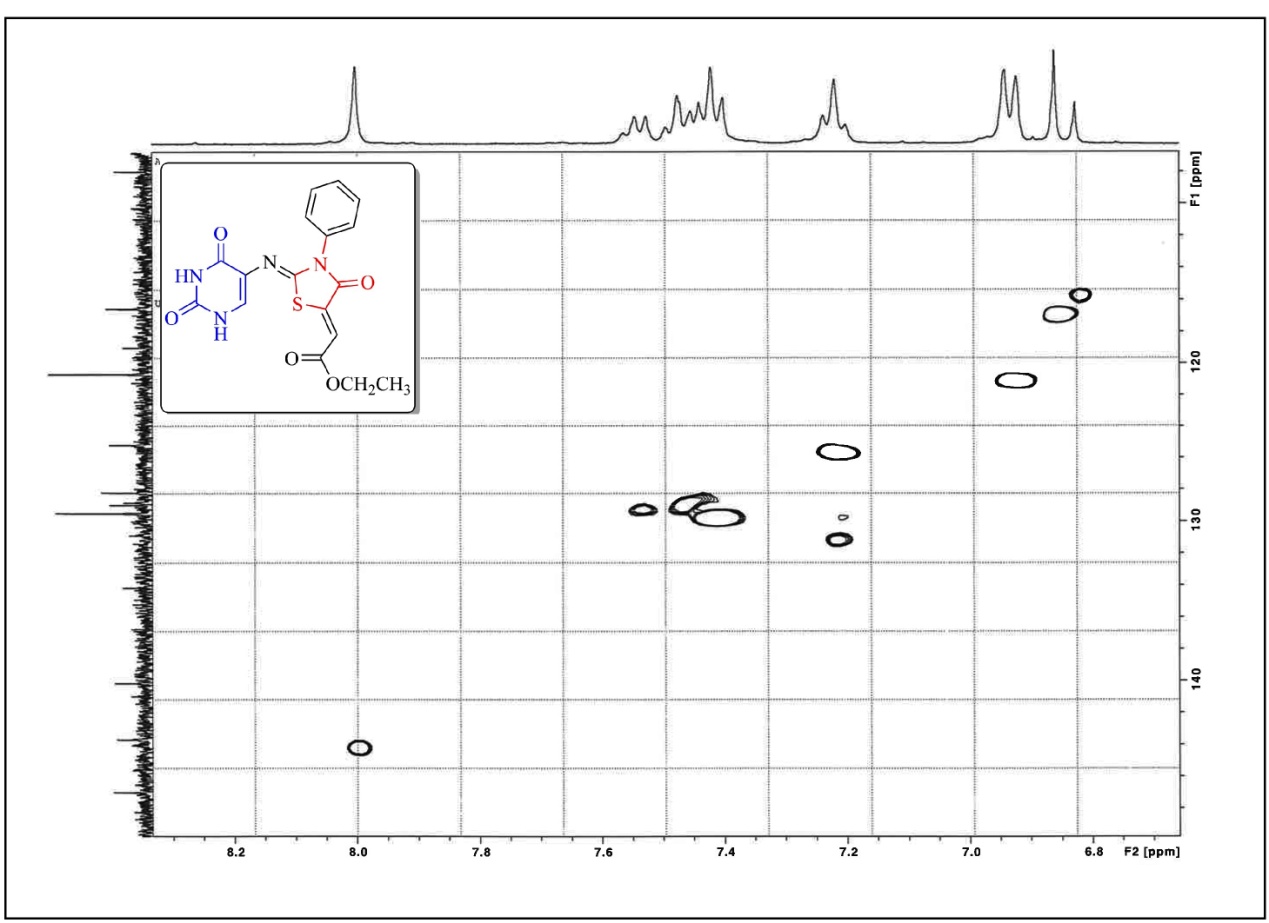


SI figure 65: ^1^H-^13^C-HSQC spectrum of **5a**


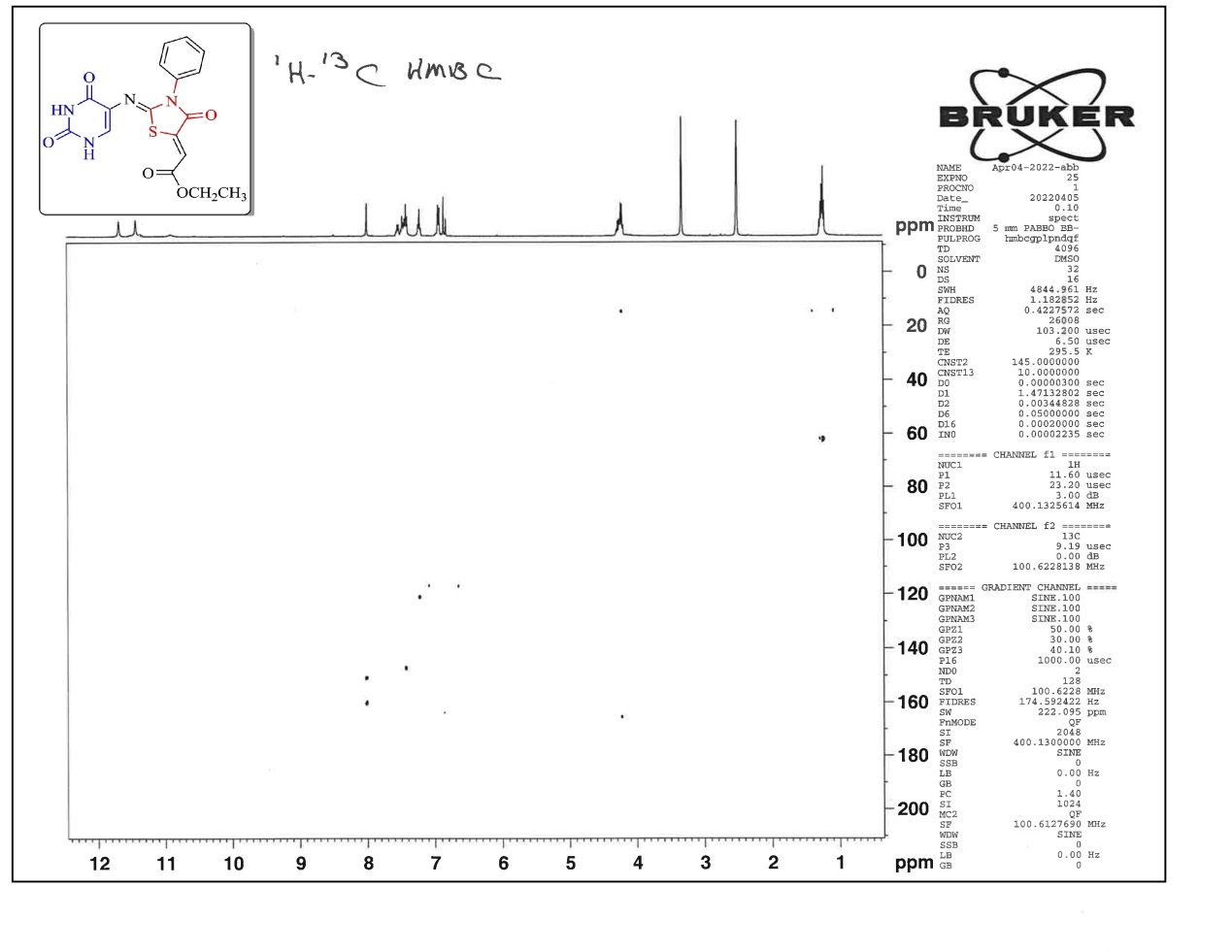


SI figure 66: ^1^H-^13^C-HMBC spectrum of **5a**


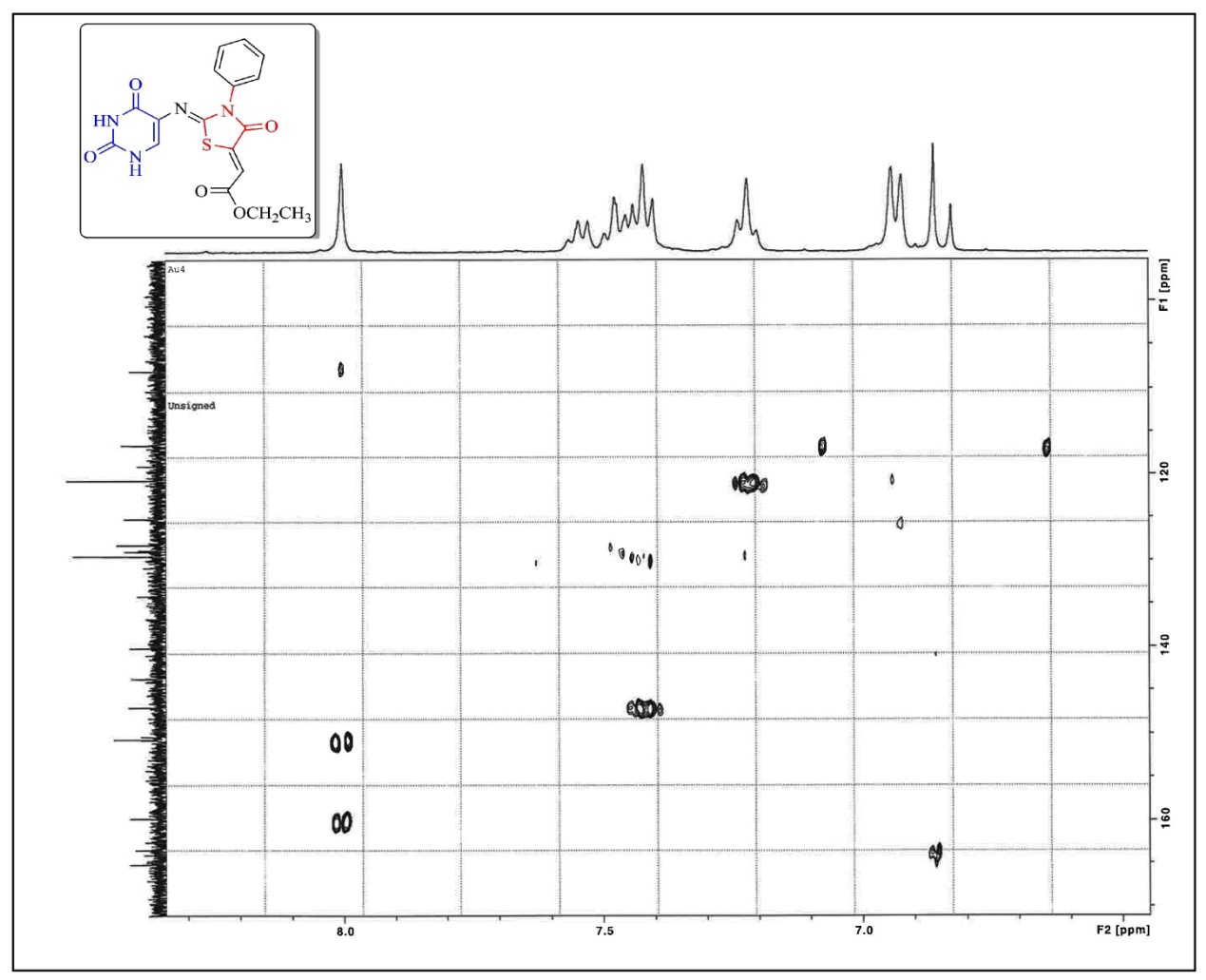


SI figure 67: ^1^H-^13^C-HMBC spectrum of **5a**


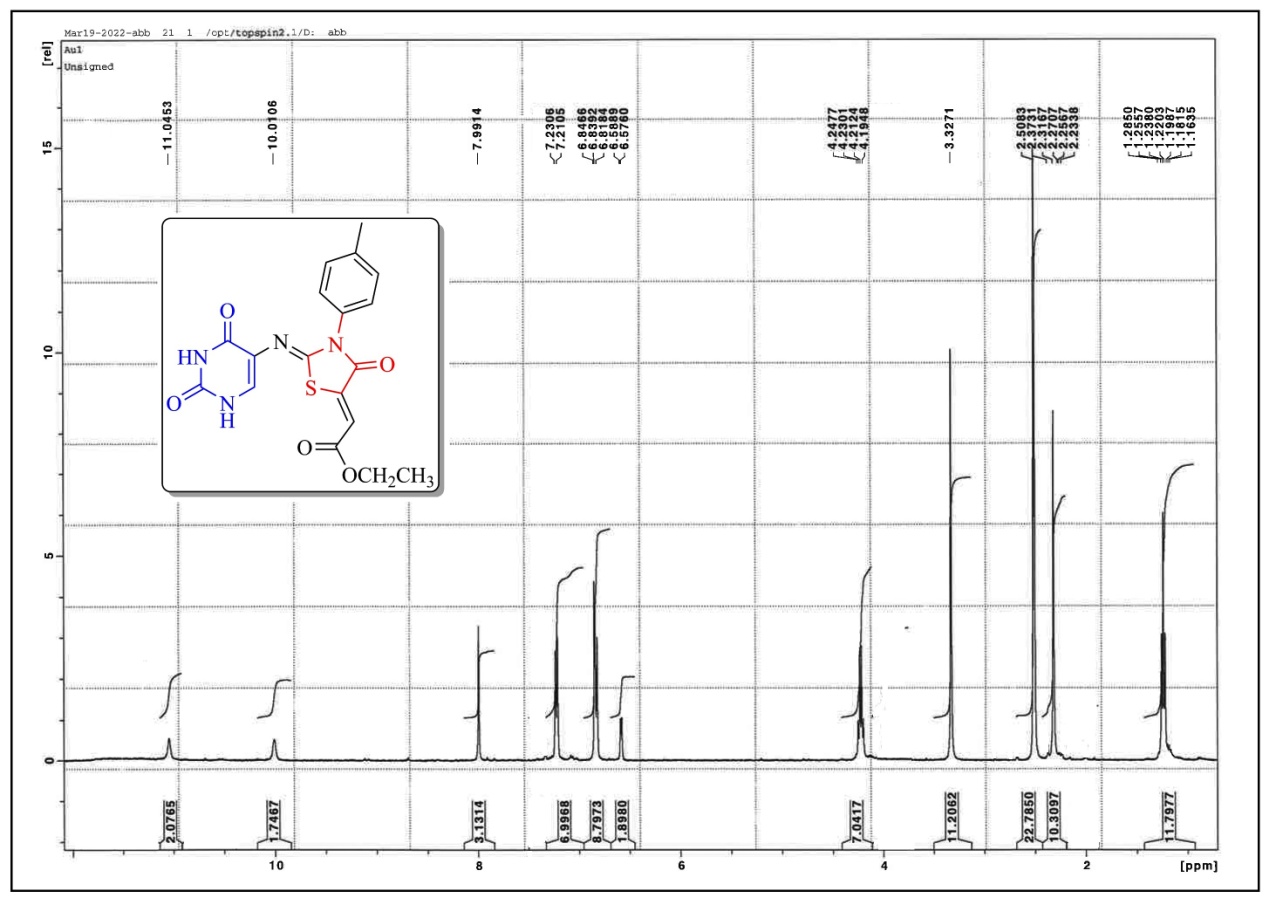


SI figure 68: ^1^H-NMR spectrum of **5b**


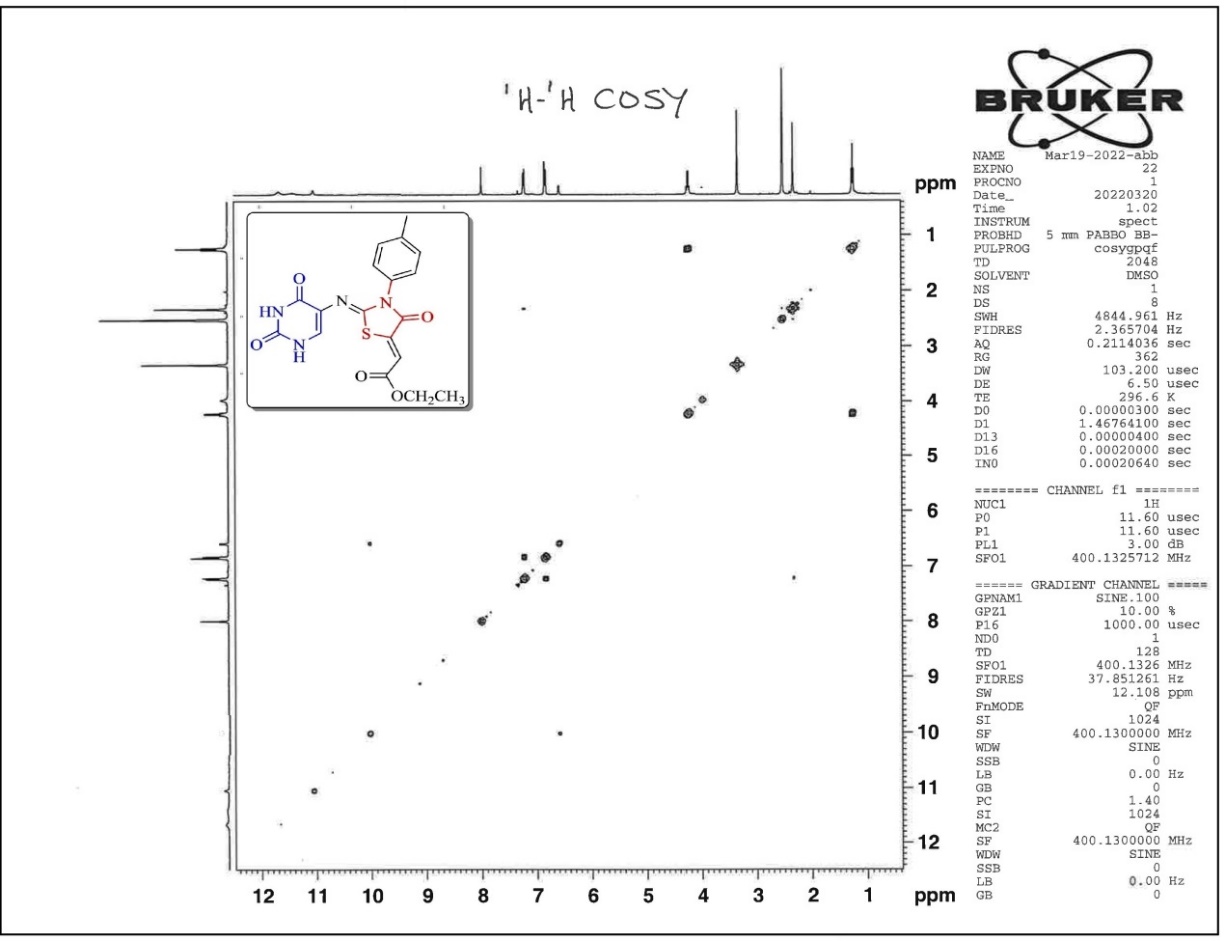


SI figure 69: ^1^H-^1^H COSY spectrum of **5b**


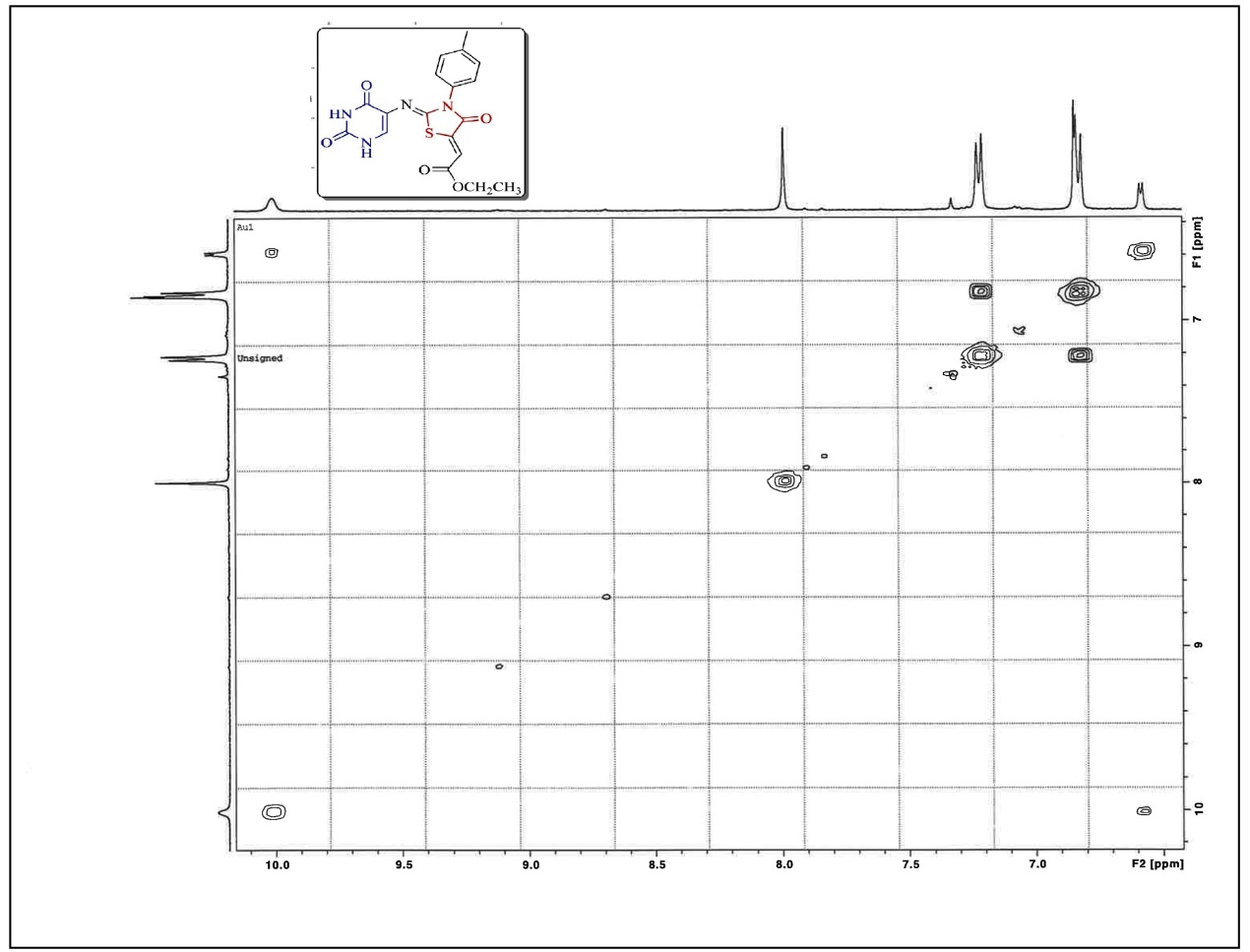


SI figure 70: ^1^H-^1^H COSY spectrum of **5b**


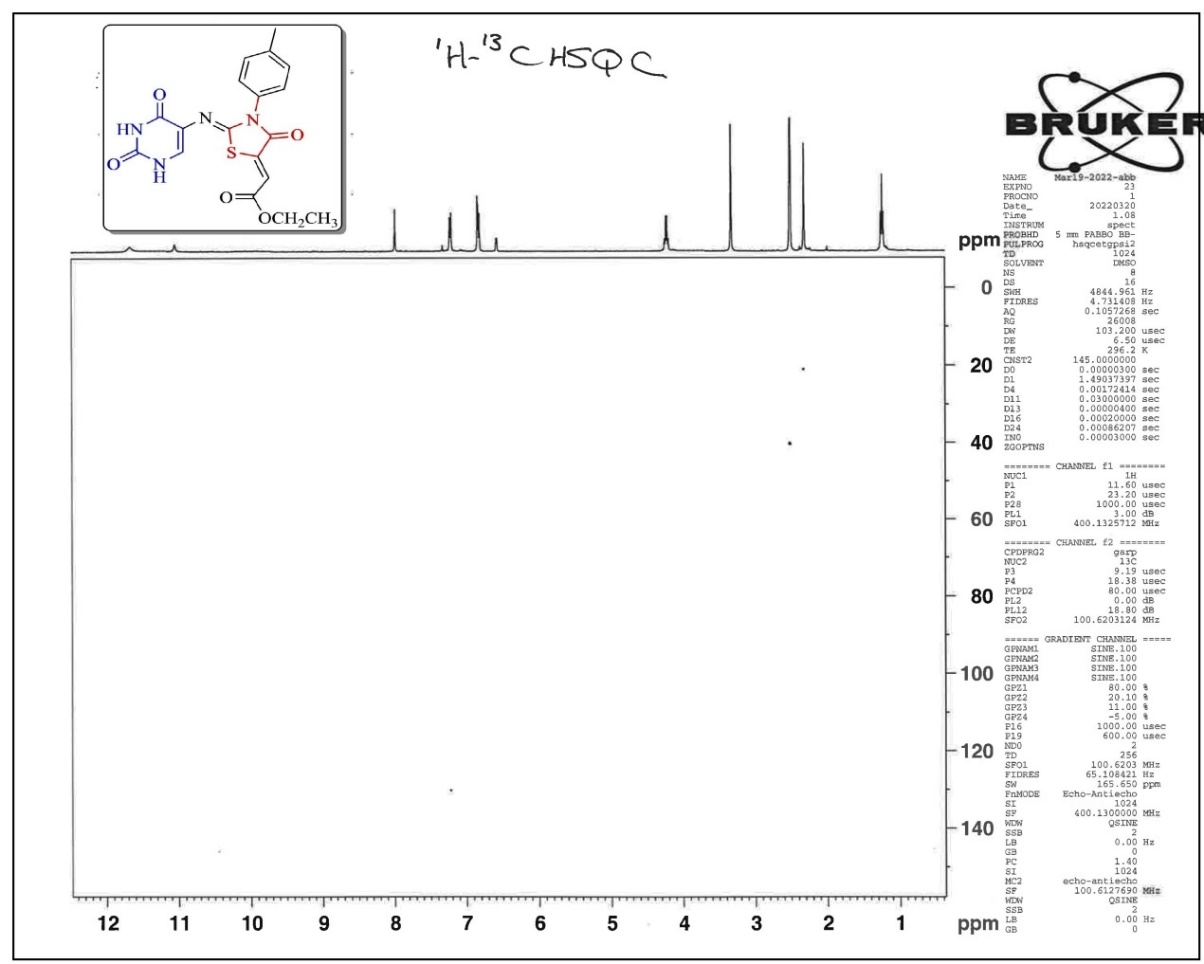


SI figure 71: ^1^H-^13^C- HSQC spectrum of **5b**


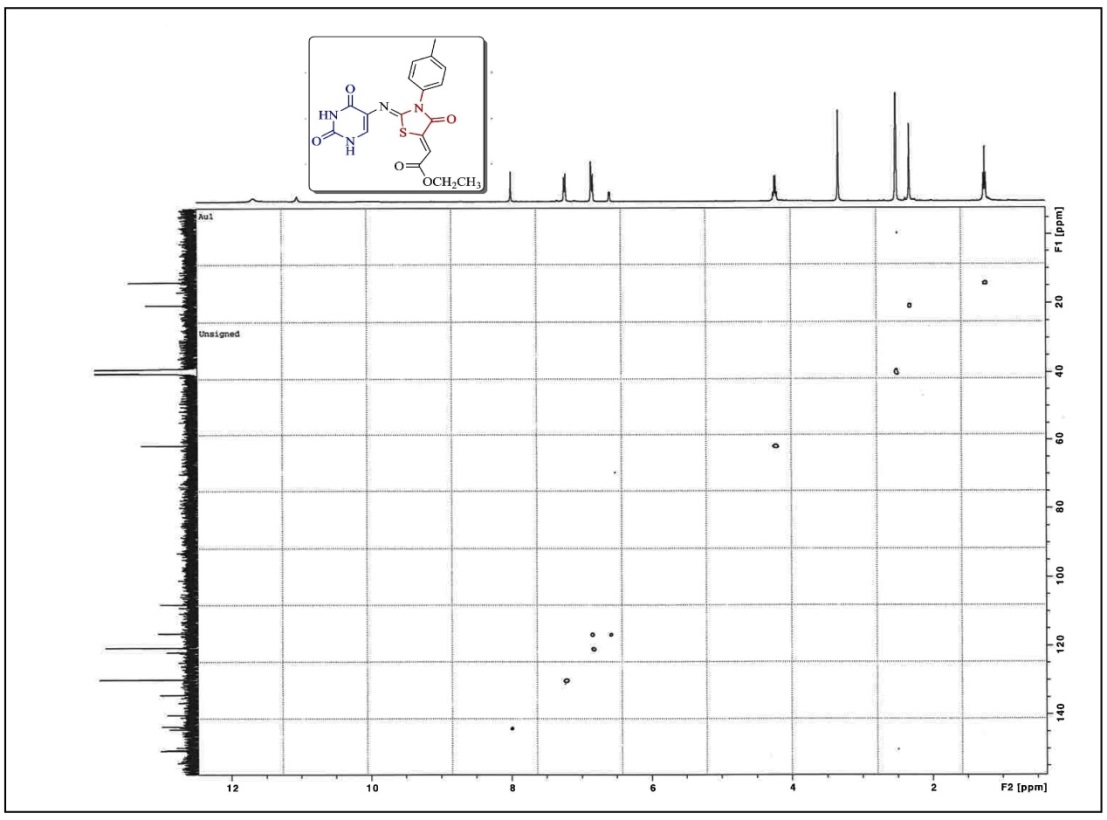


SI figure 72: ^1^H-^13^C- HSQC spectrum of **5b**


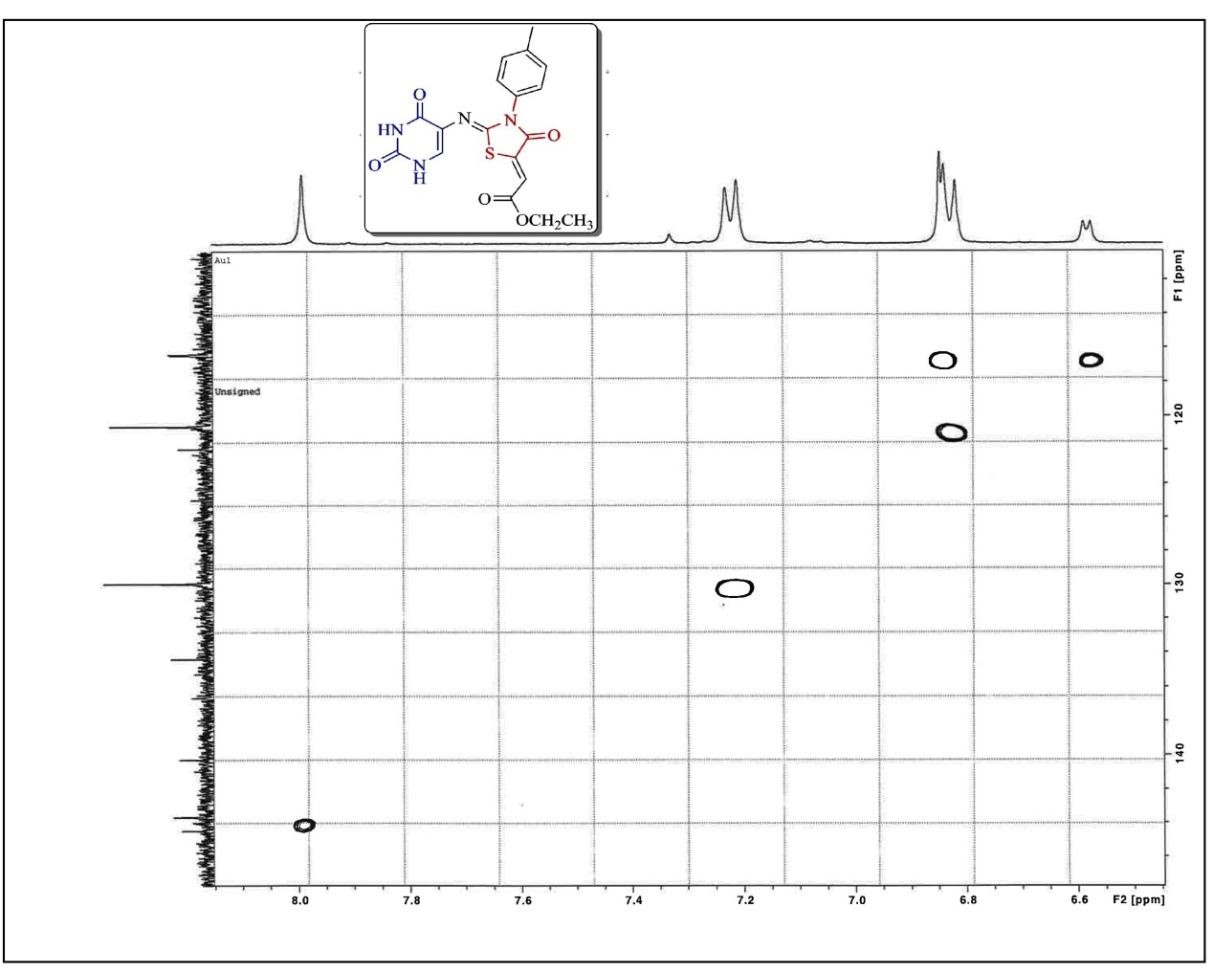


SI figure 73: ^1^H-^13^C- HSQC spectrum of **5b**


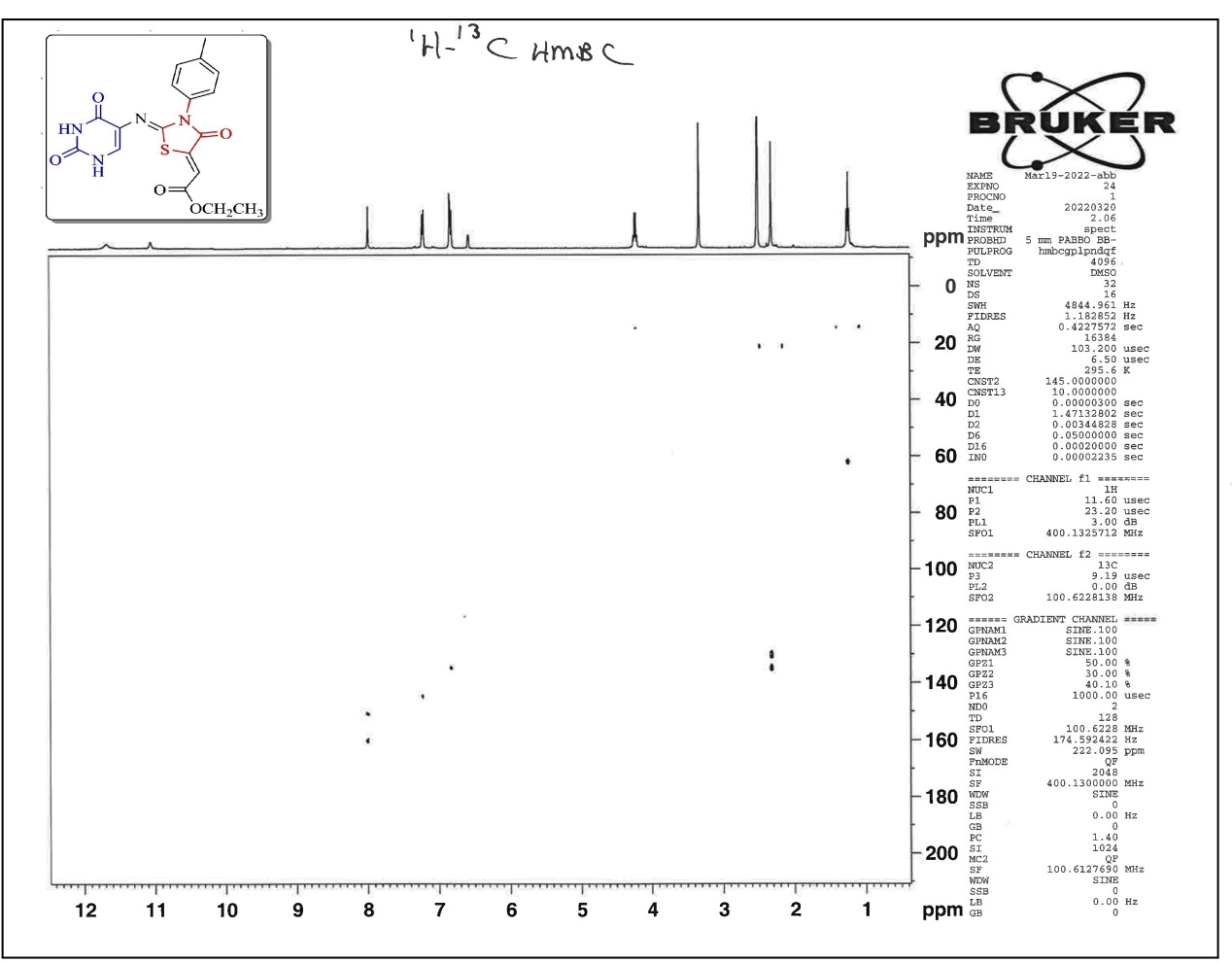


SI figure 74: ^1^H-^13^C- HMBC spectrum of **5b**


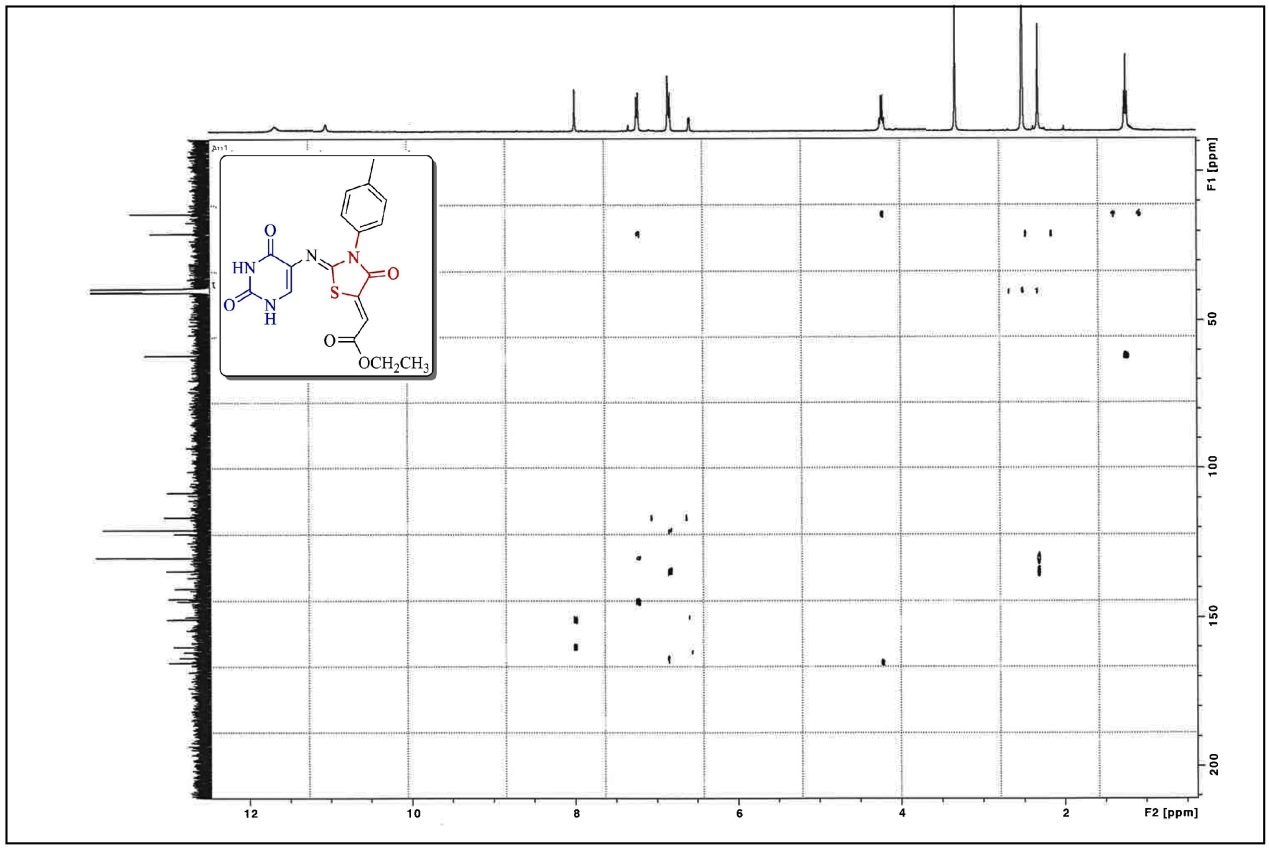


SI figure 75: ^1^H-^13^C- HMBC spectrum of **5b**


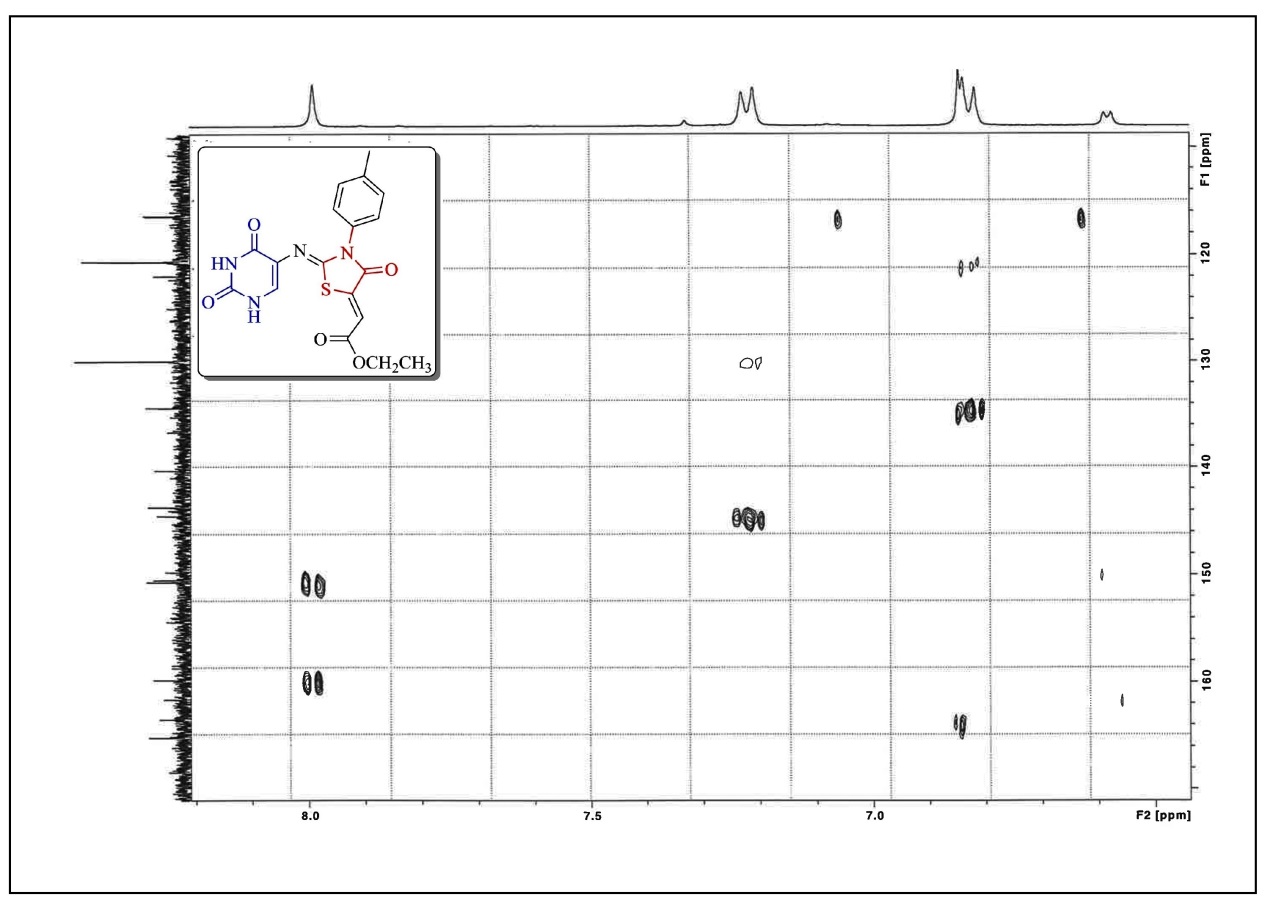


SI figure 76: ^1^H-^13^C- HMBC spectrum of **5b**


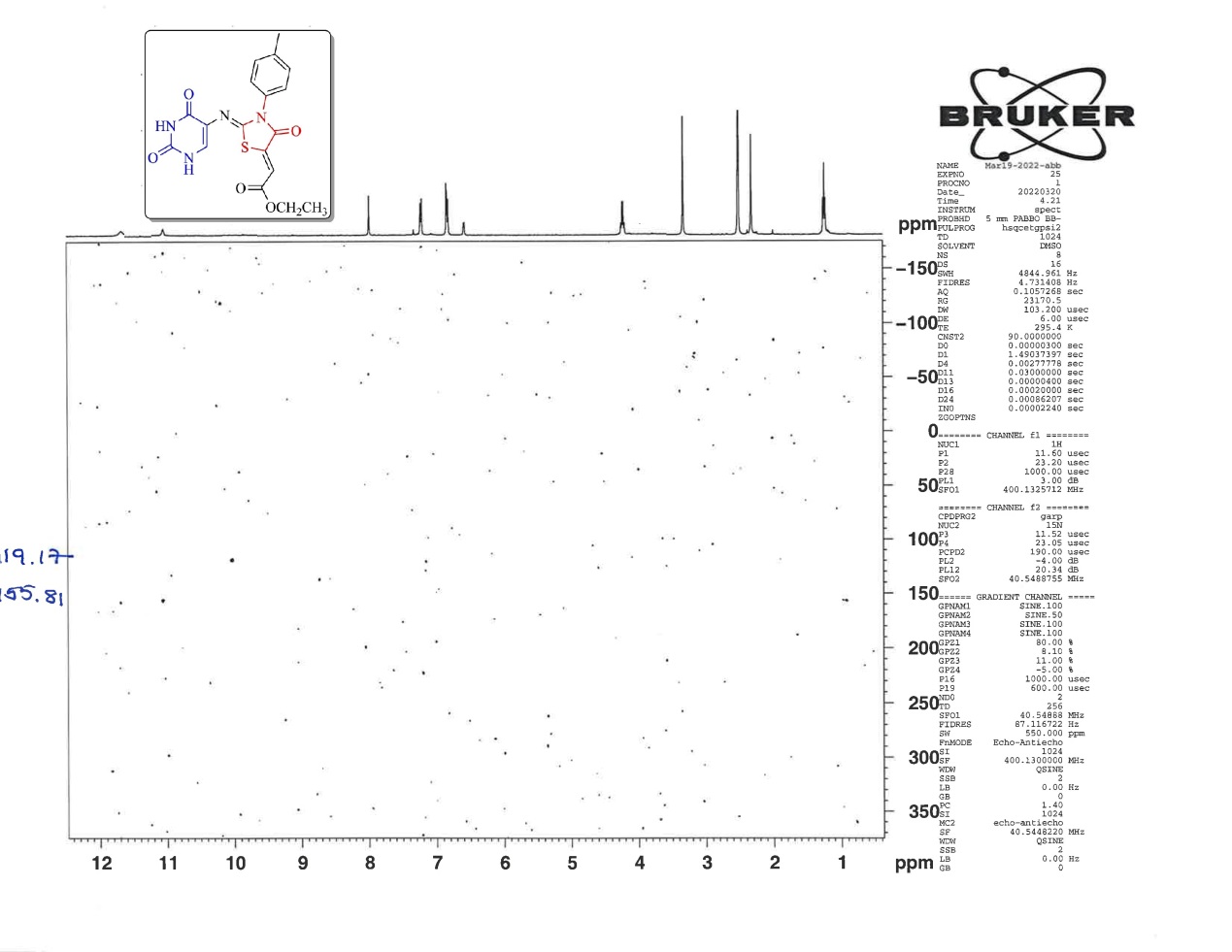


SI figure 76: ^1^H-^15^C- HSQC spectrum of **5b**


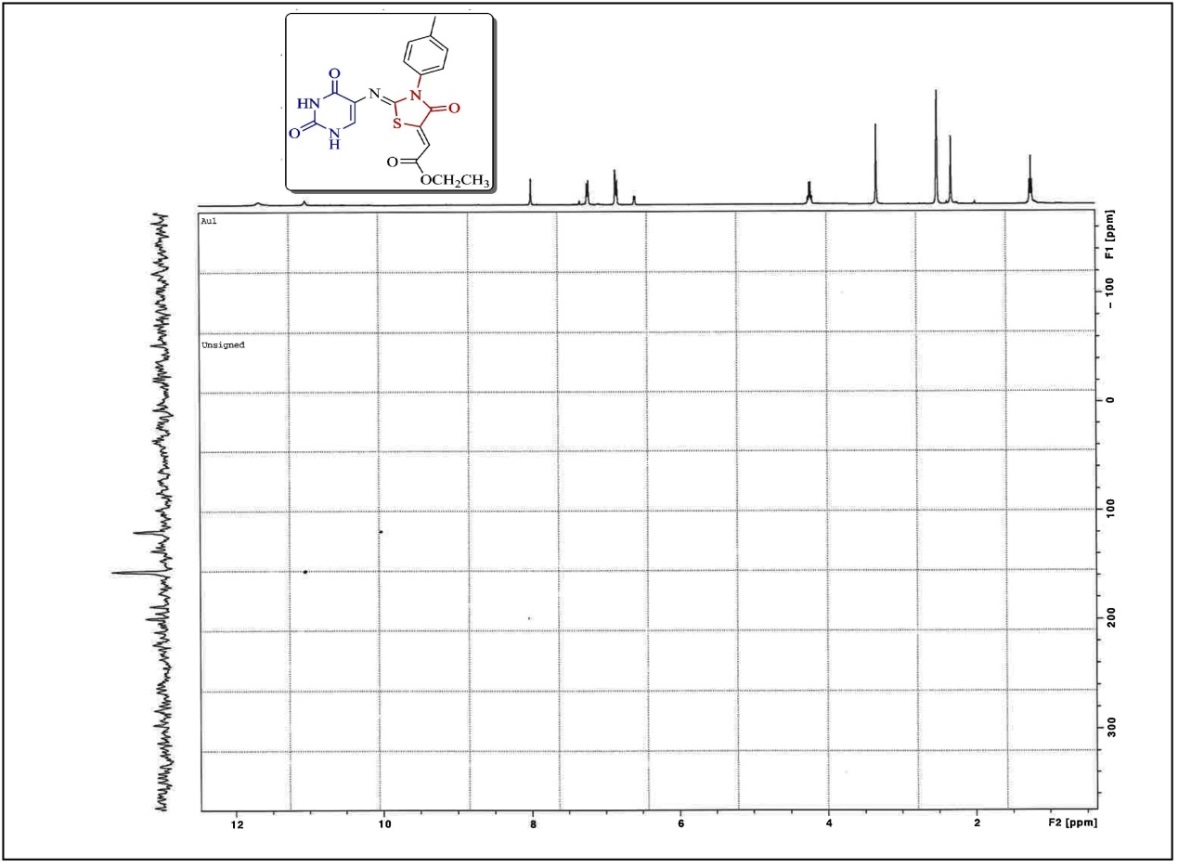


SI figure 77: ^1^H-^15^C- HSQC spectrum of **5b**


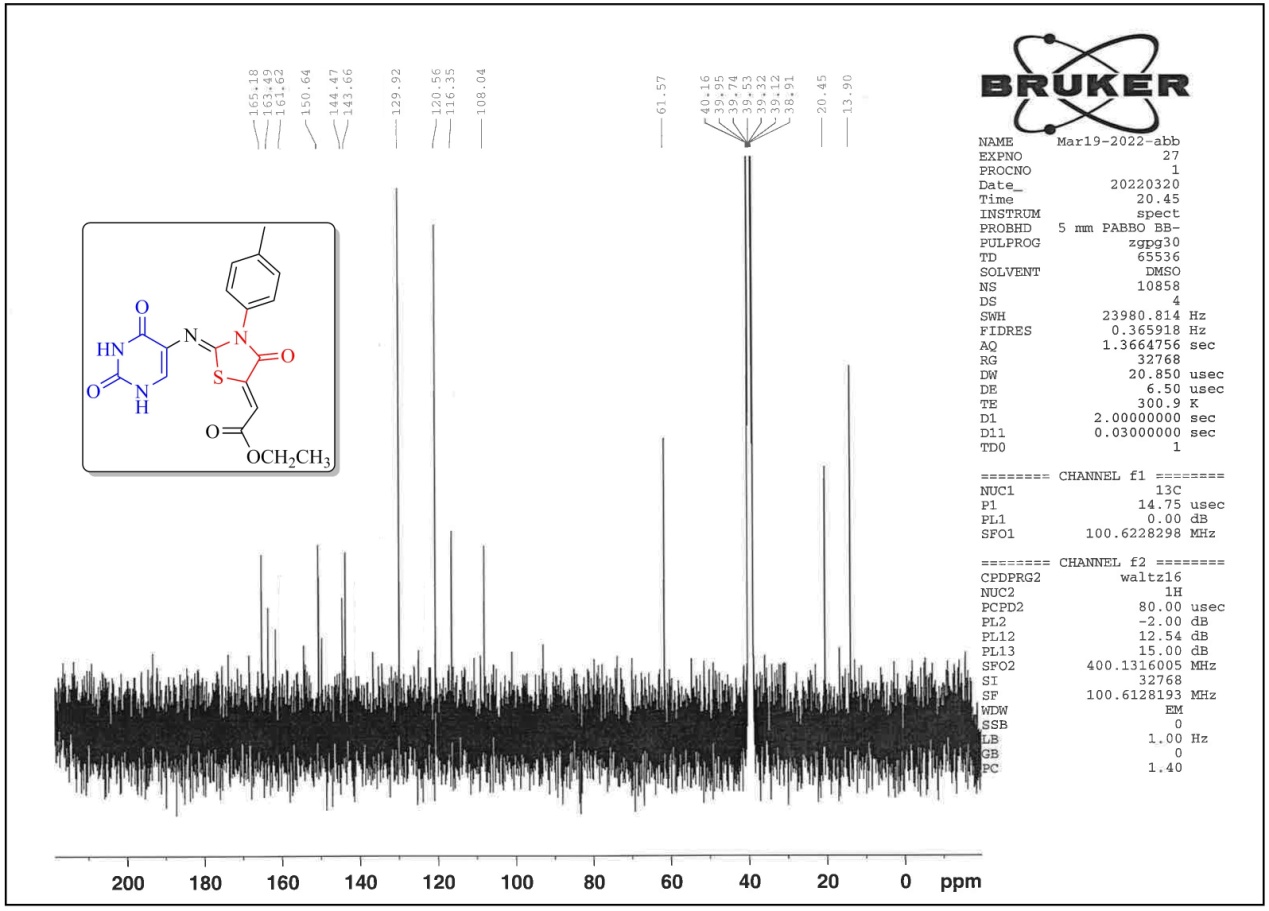


SI figure 78: ^13^C- NMR spectrum of **5b**


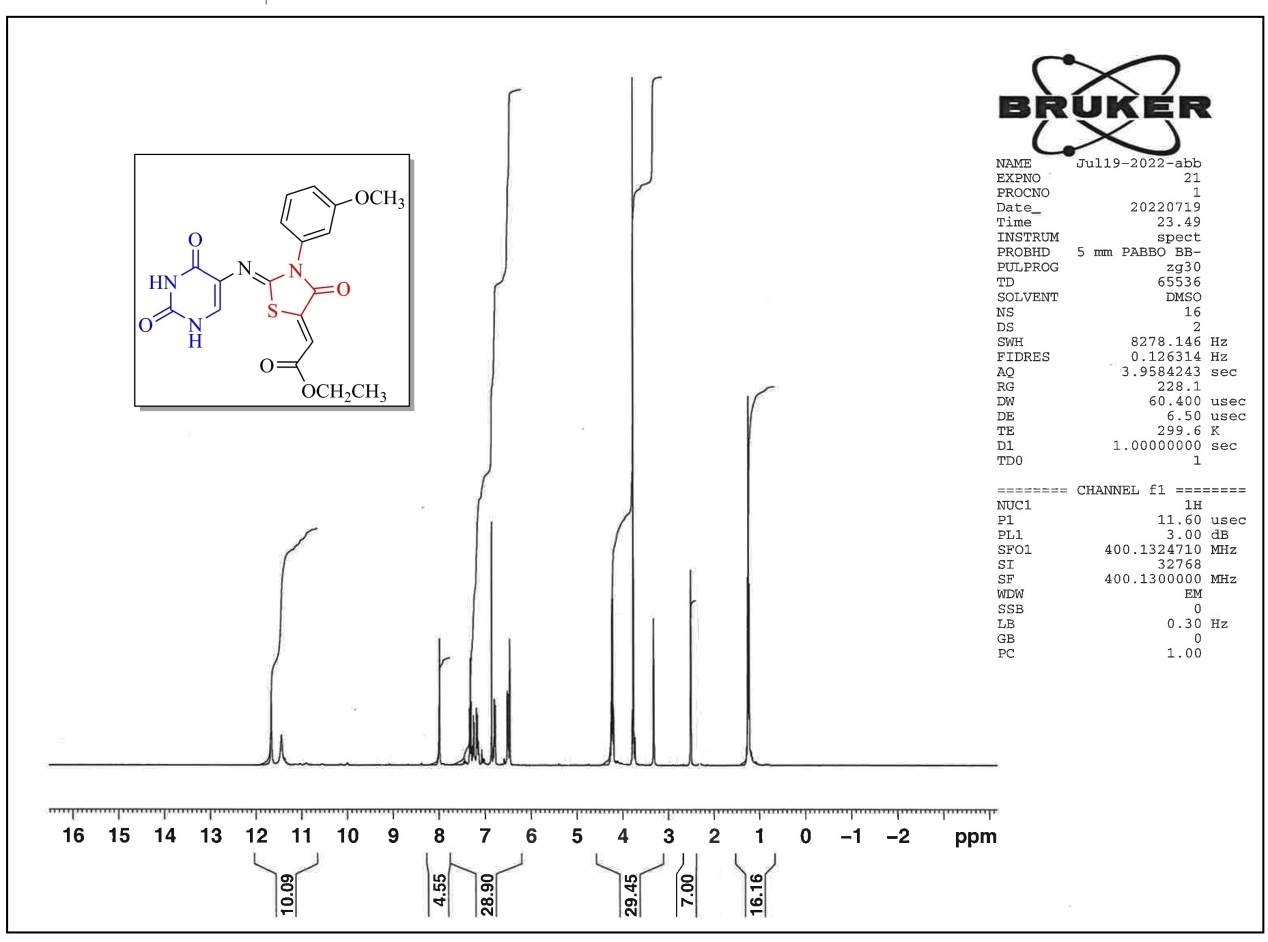


SI figure 79: ^1^H- NMR spectrum of **5c**


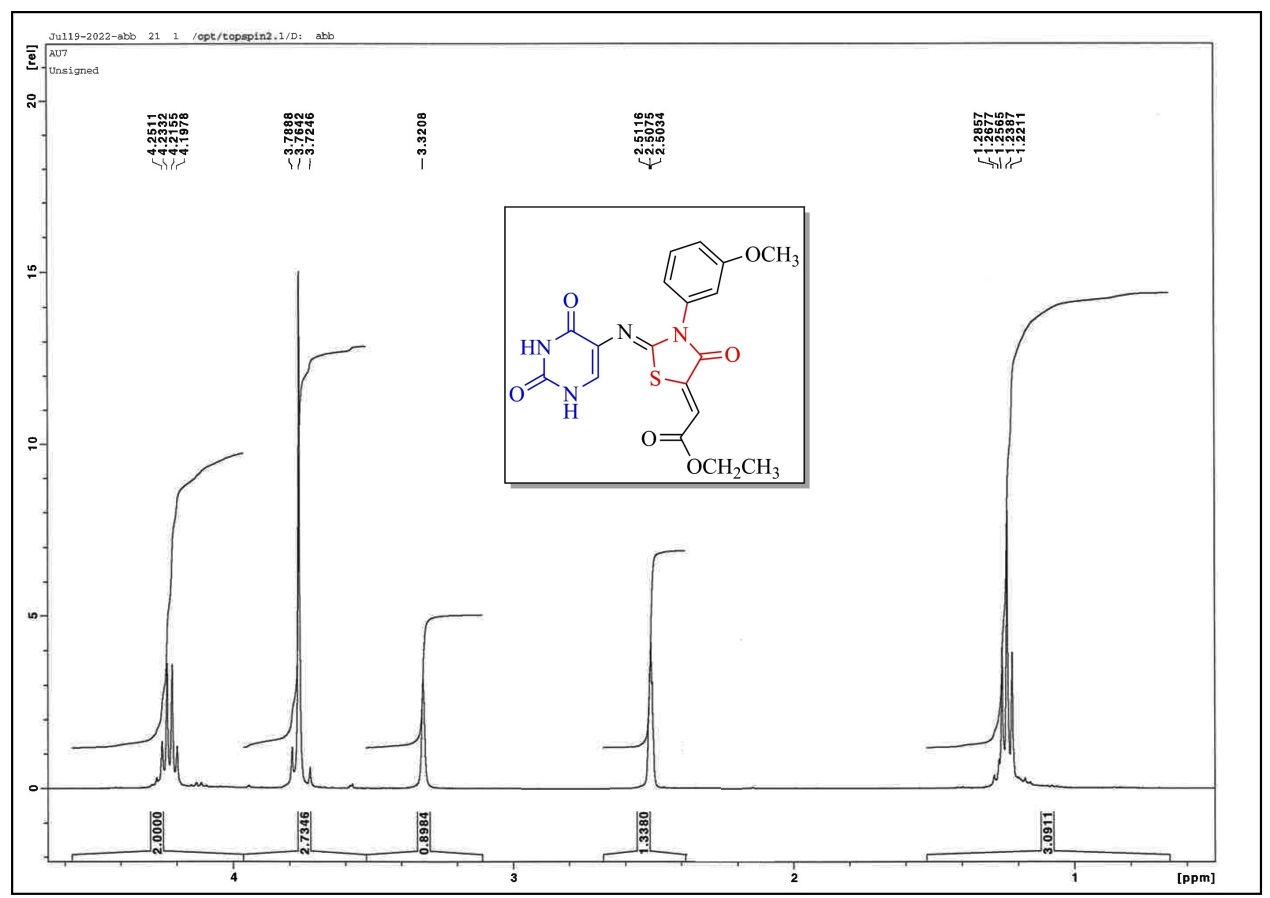


SI figure 80: ^1^H- NMR spectrum of **5c**


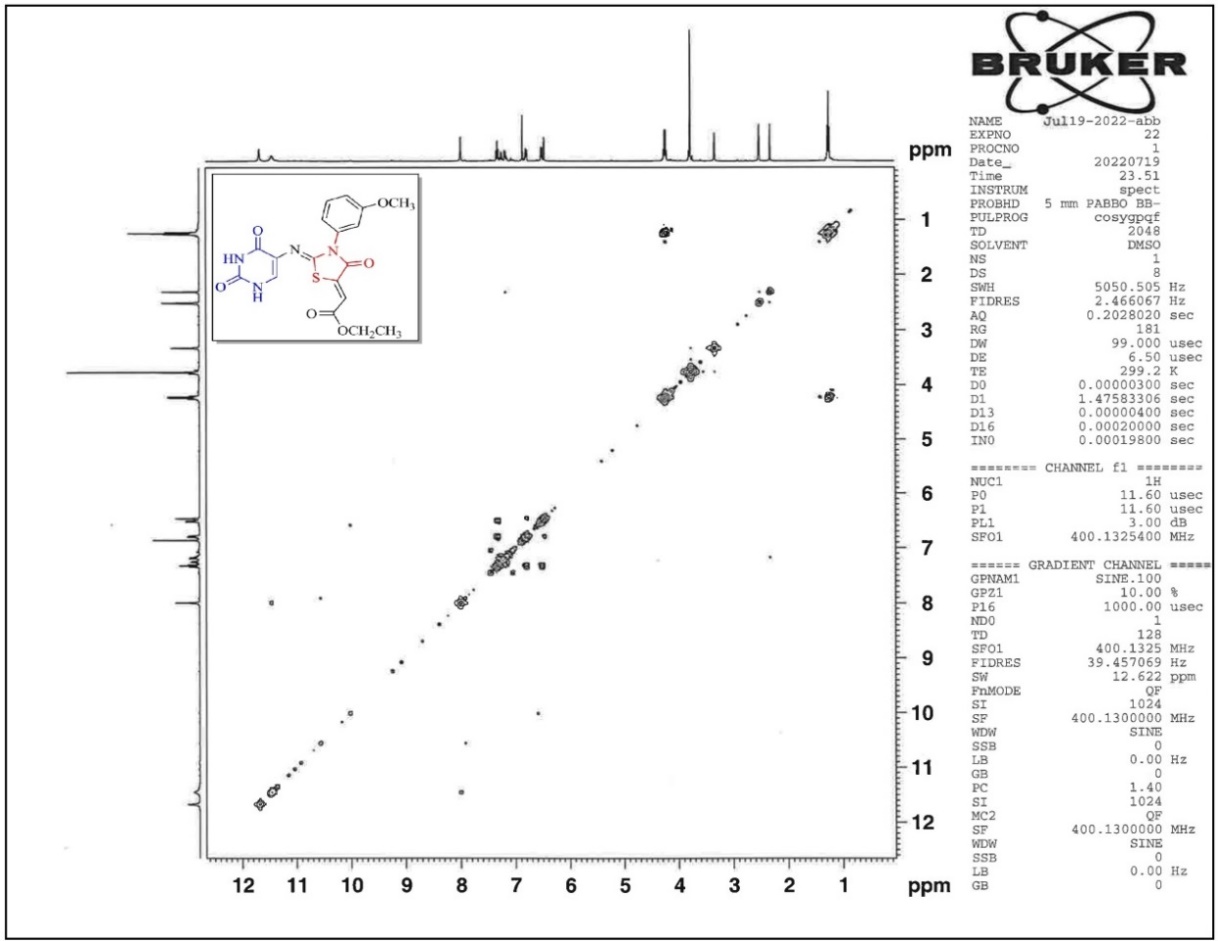


SI figure 81: ^1^H- ^1^H COSY spectrum of **5c**


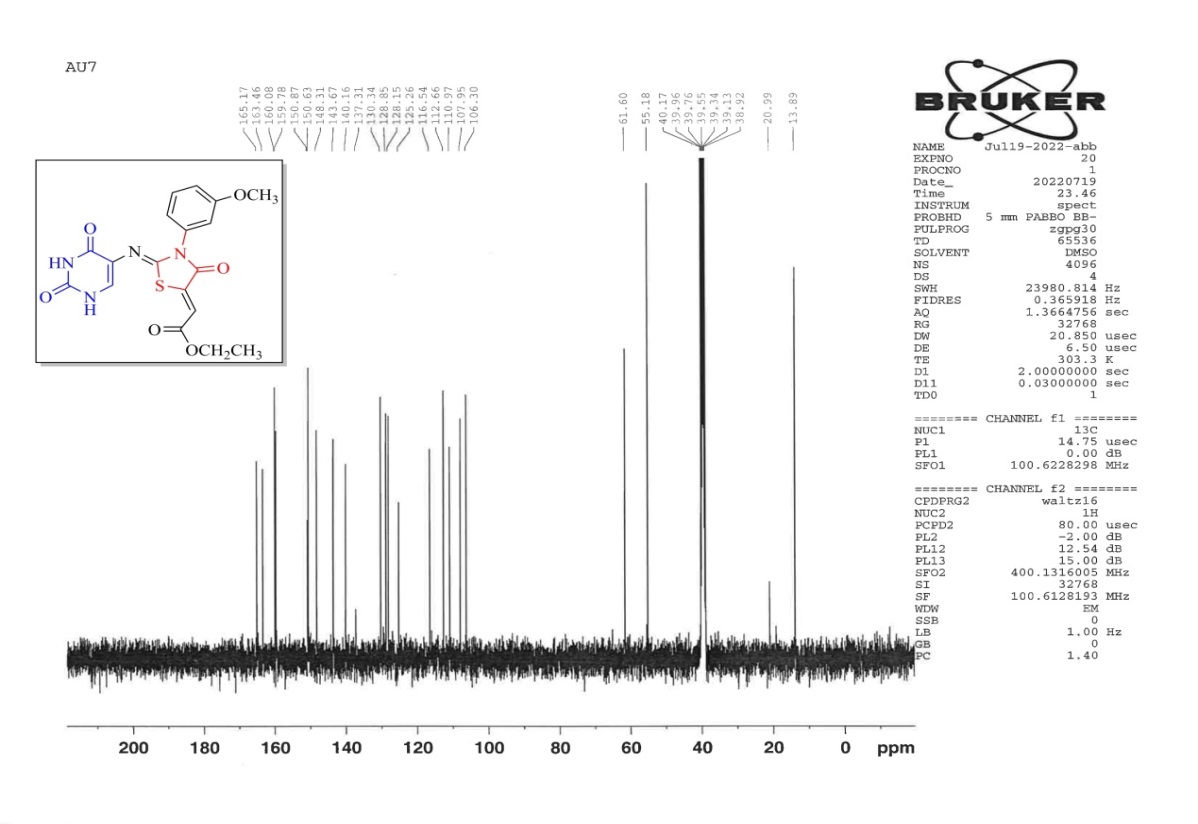


SI figure 82: ^13^C- NMR spectrum of **5c**


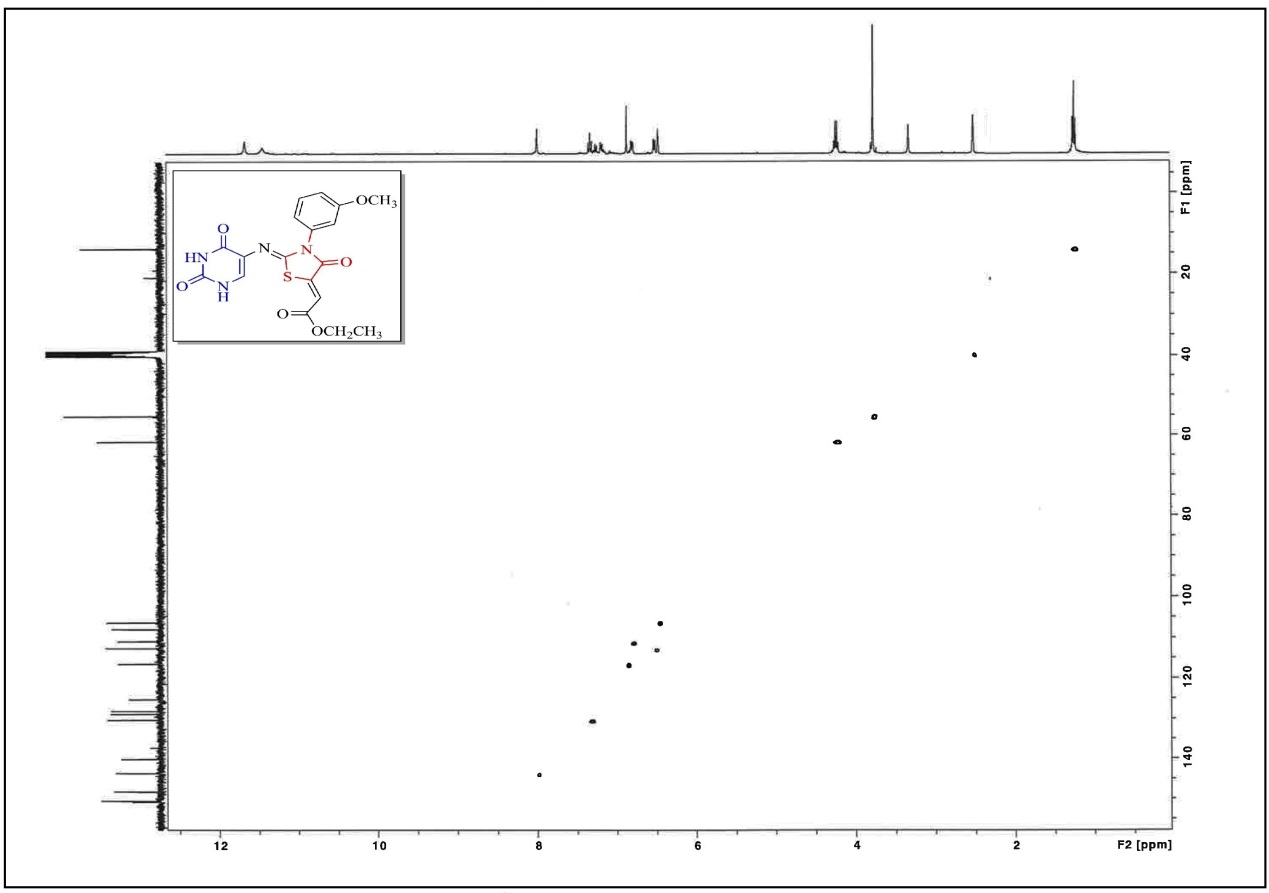


SI figure 83:^1^H- ^13^C- HSQC spectrum of **5c**


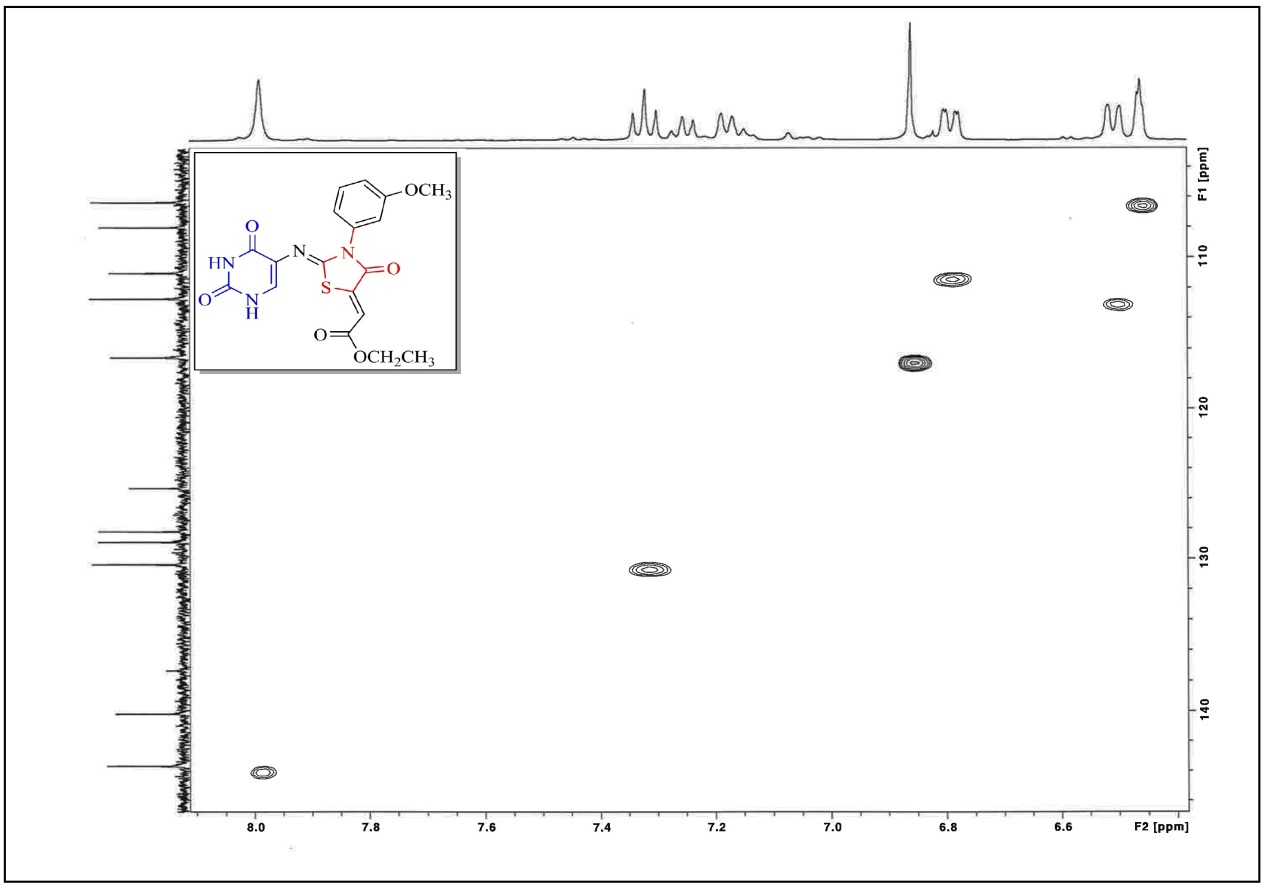


SI figure 84:^1^H- ^13^C- HSQC spectrum of **5c**


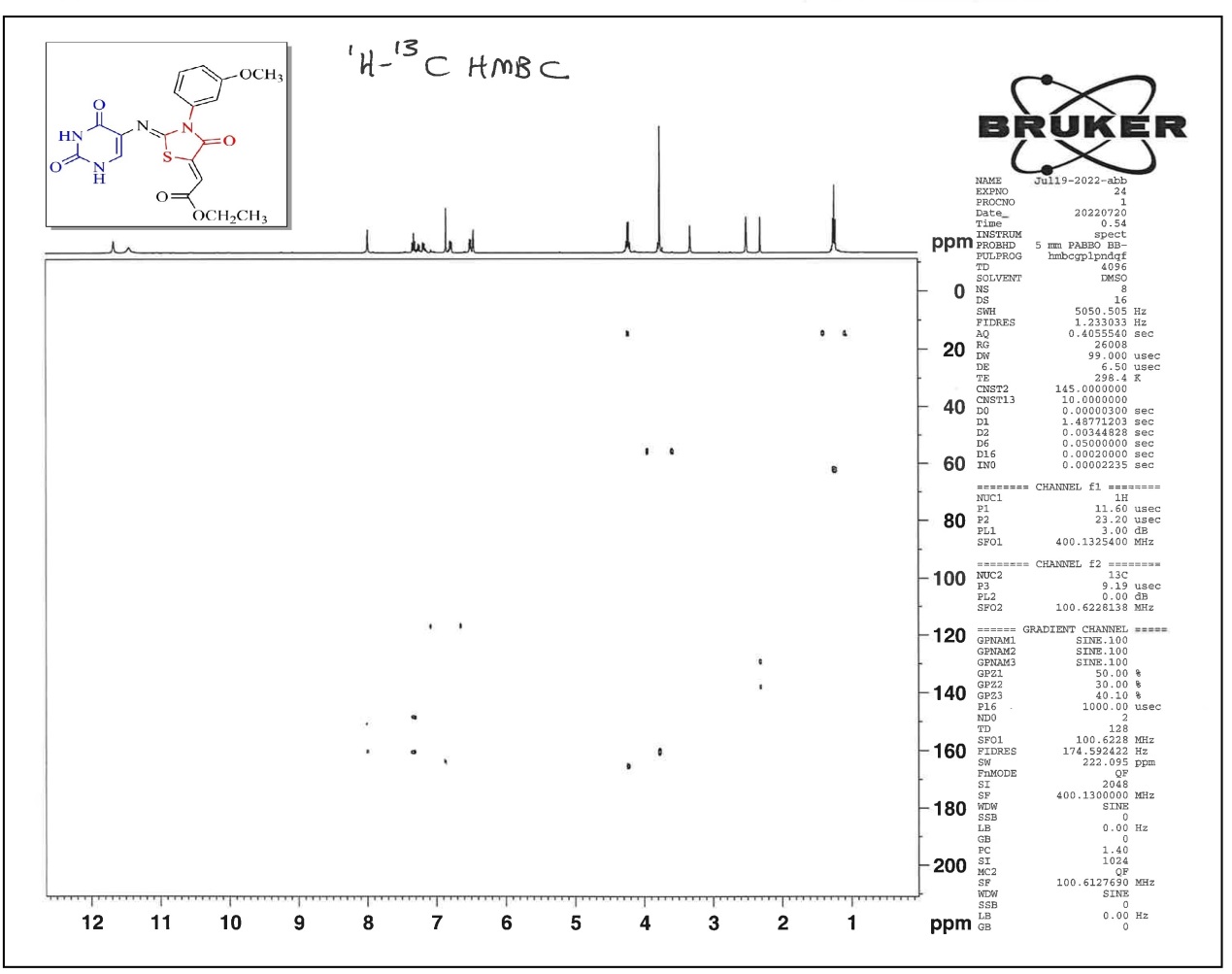


SI figure 83:^1^H- ^13^C- HMBC spectrum of **5c**


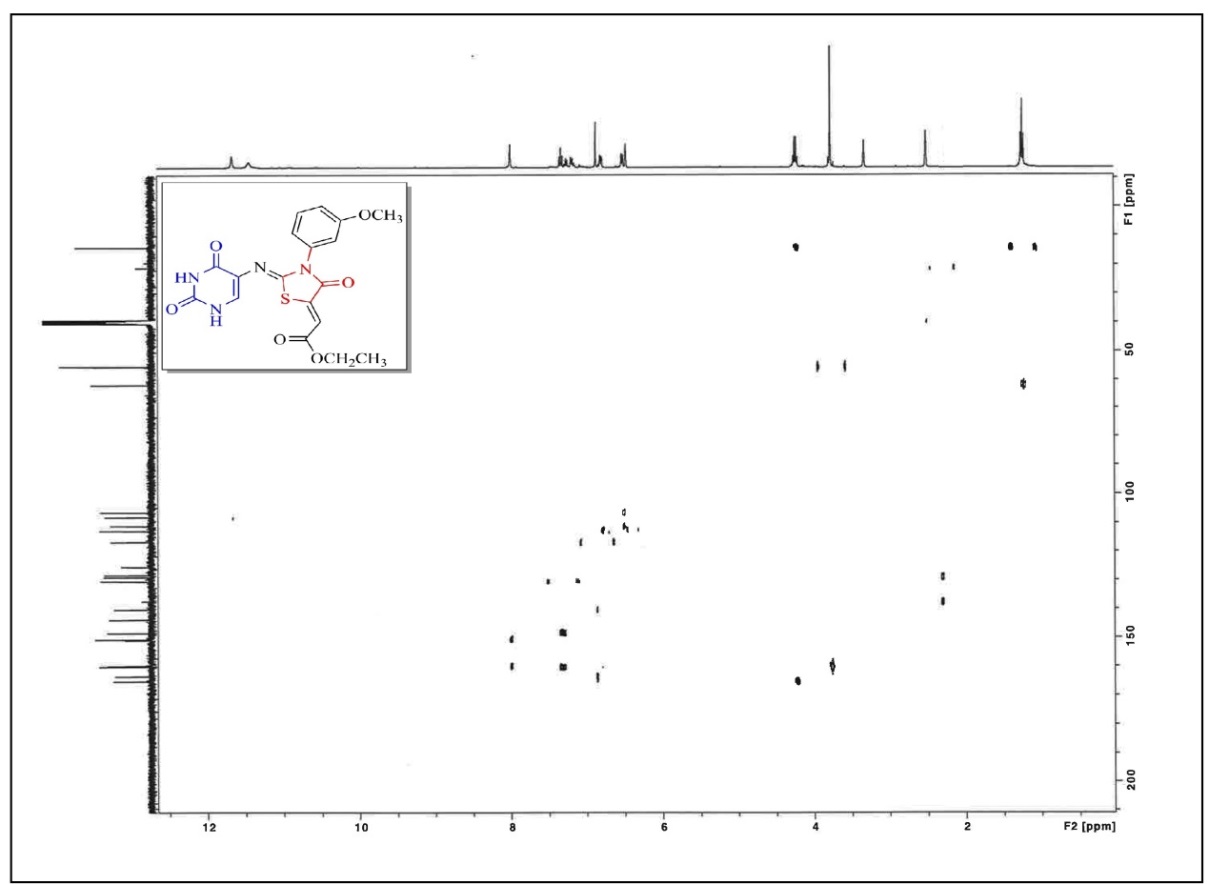


SI figure 84:^1^H- ^13^C- HMBC spectrum of **5c**


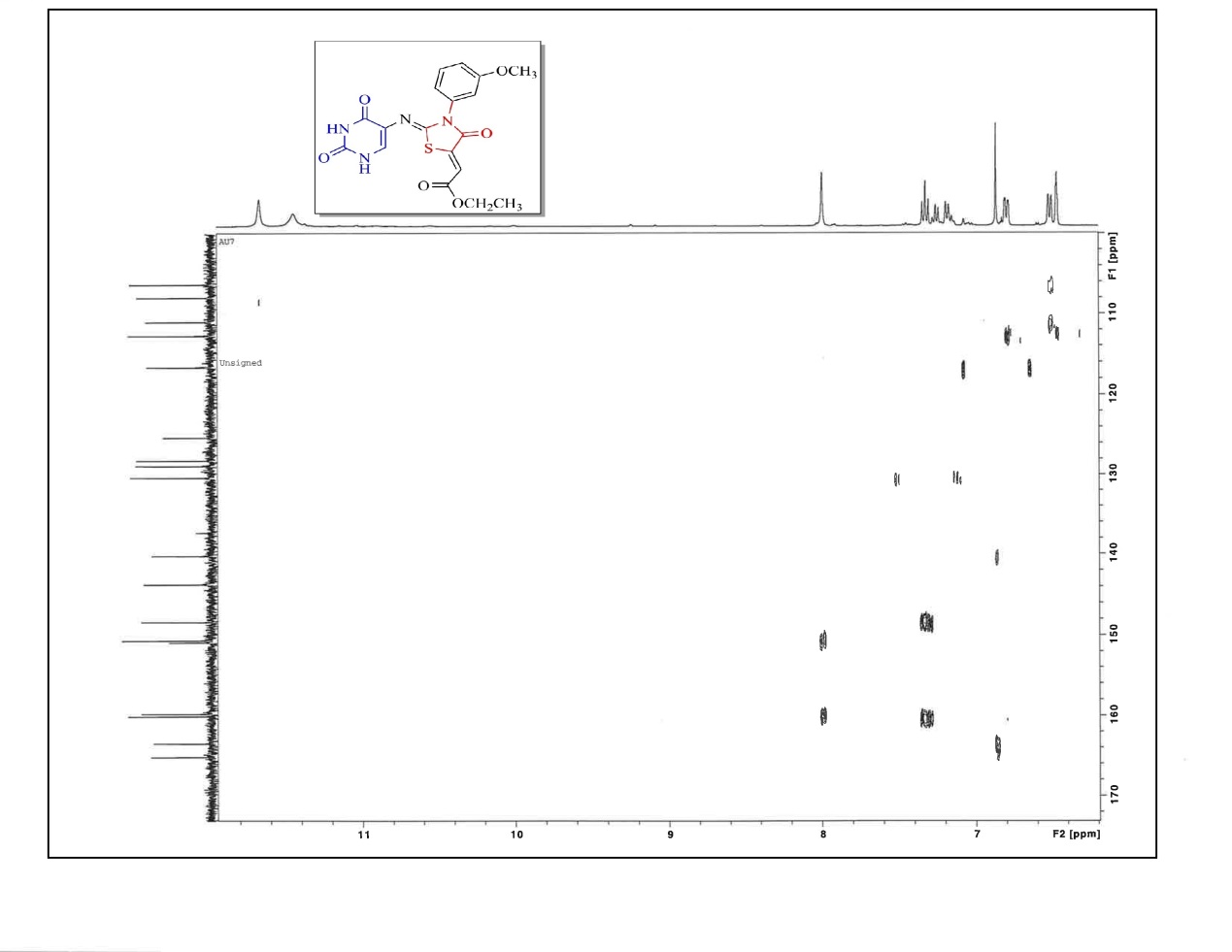


SI figure 85:^1^H- ^13^C- HMBC spectrum of **5c**


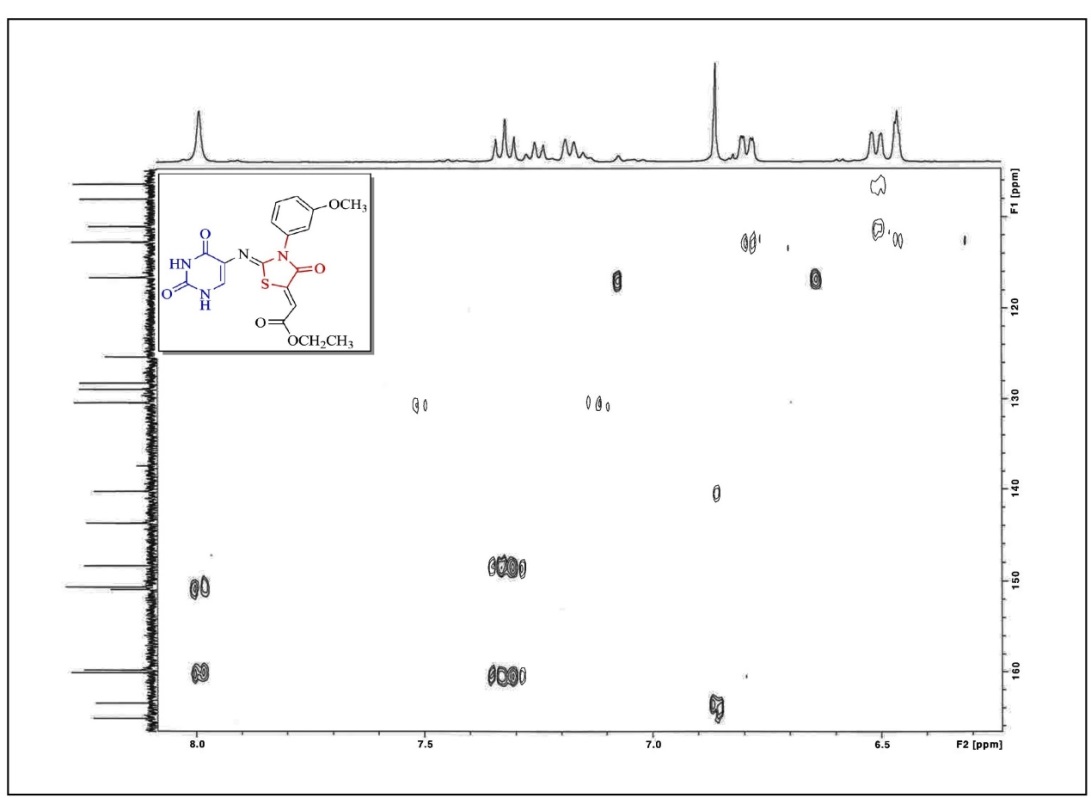


SI figure 86:^1^H- ^13^C- HMBC spectrum of **5c**


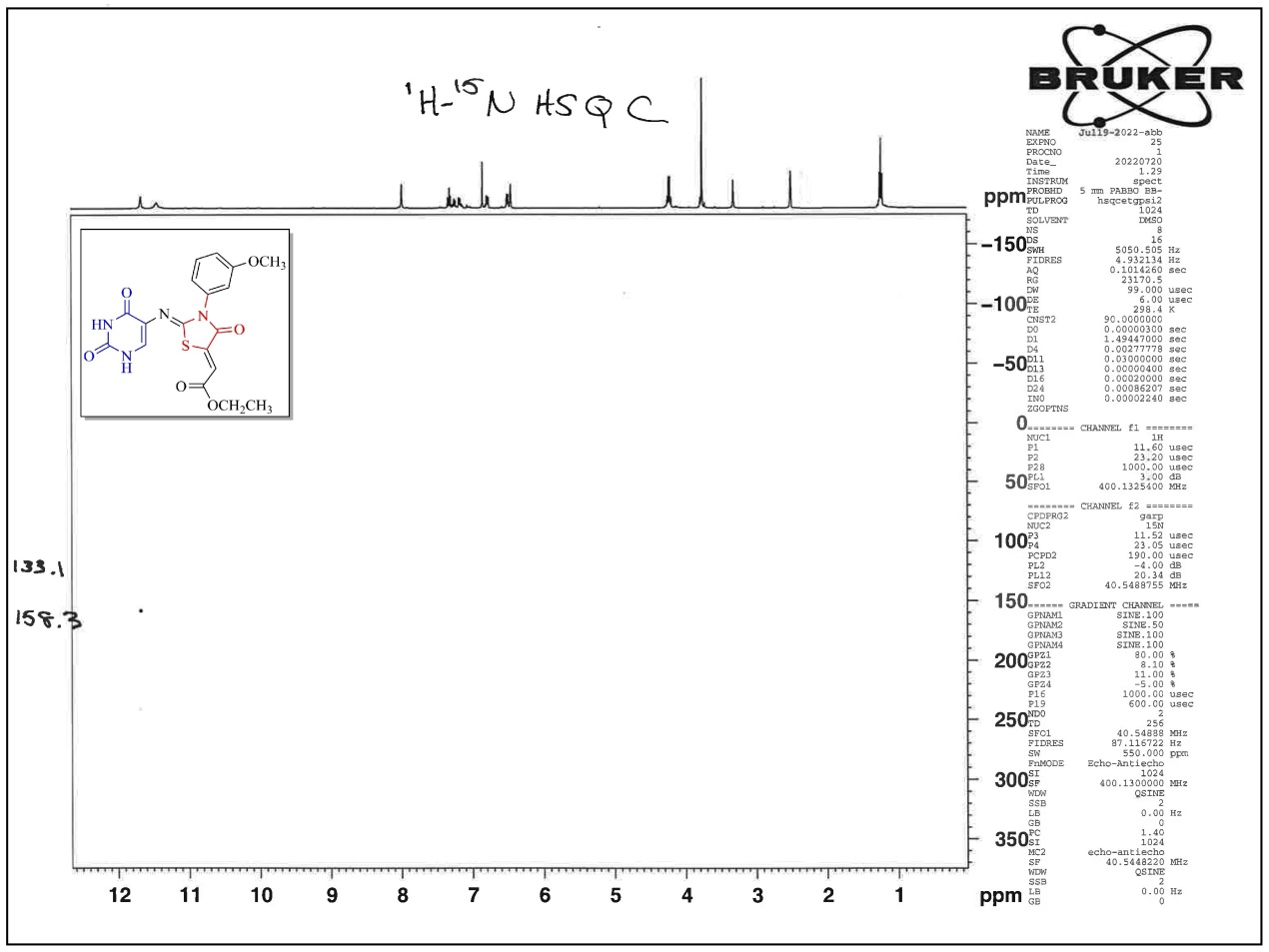


SI figure 87:^1^H- ^15^N- HSQC spectrum of **5c**


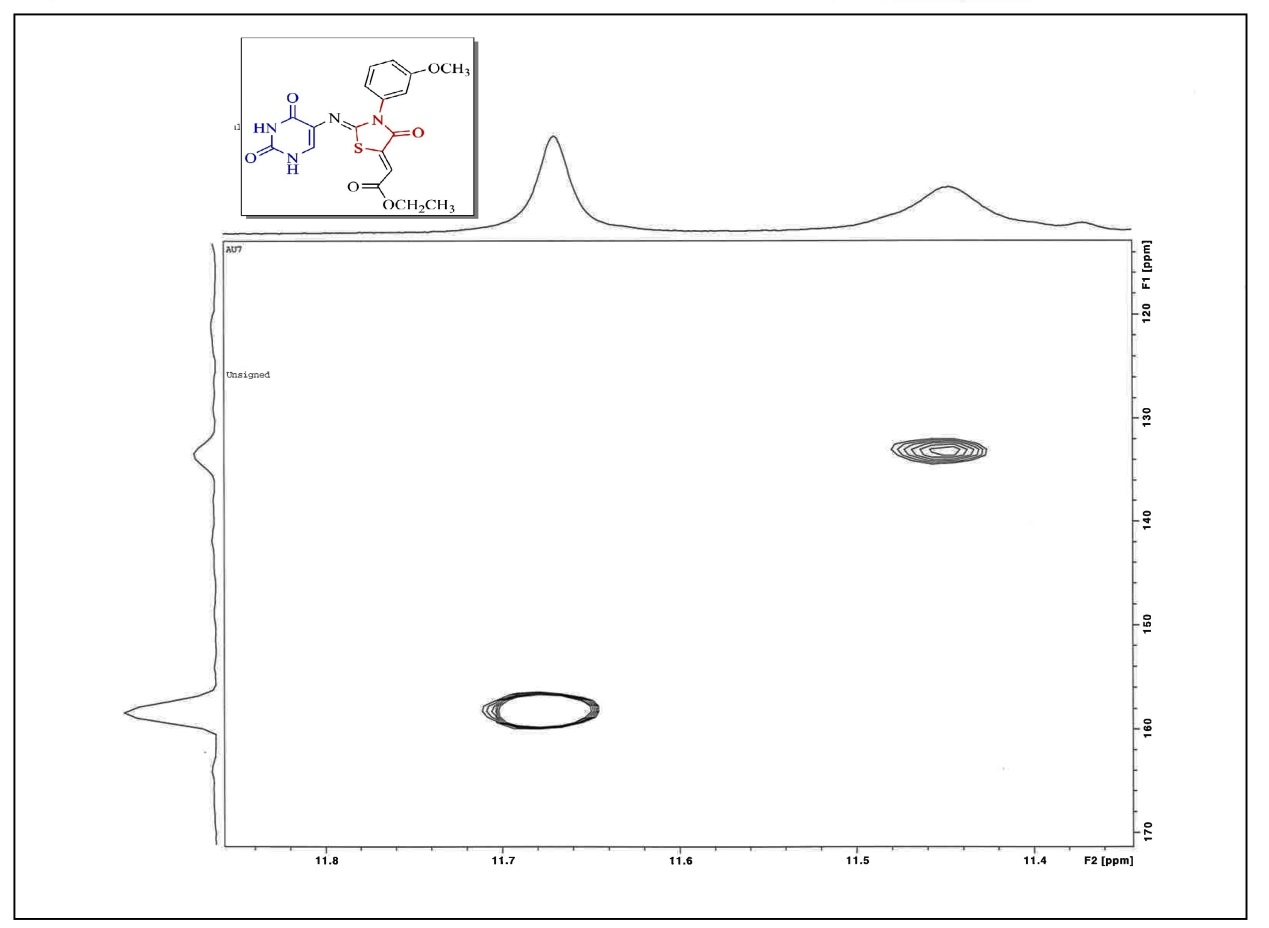


SI figure 88:^1^H- ^15^N- HSQC spectrum of **5c**


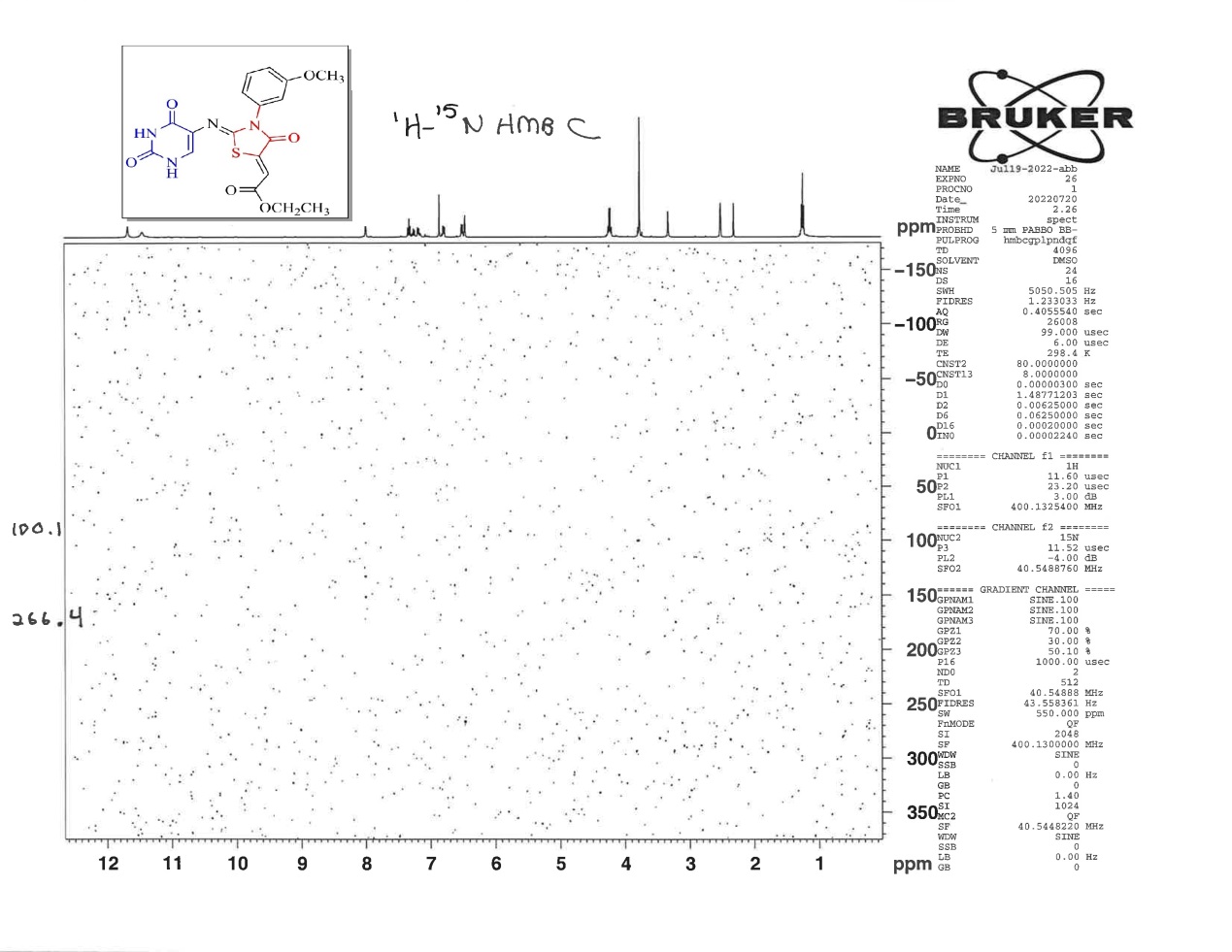


SI figure 89:^1^H- ^15^N- HMBC spectrum of **5c**


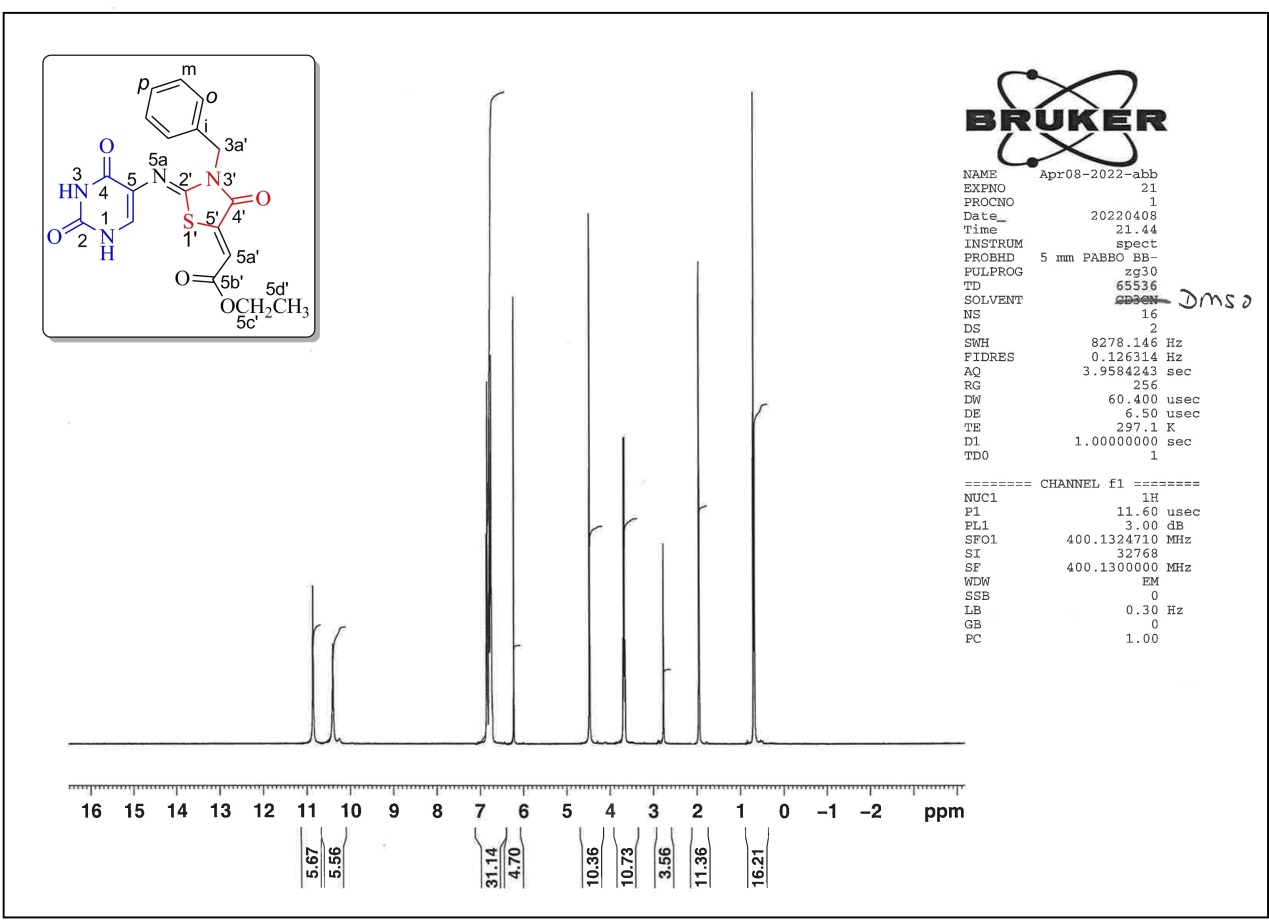


SI figure 90: ^1^H-NMR spectrum of **5d**


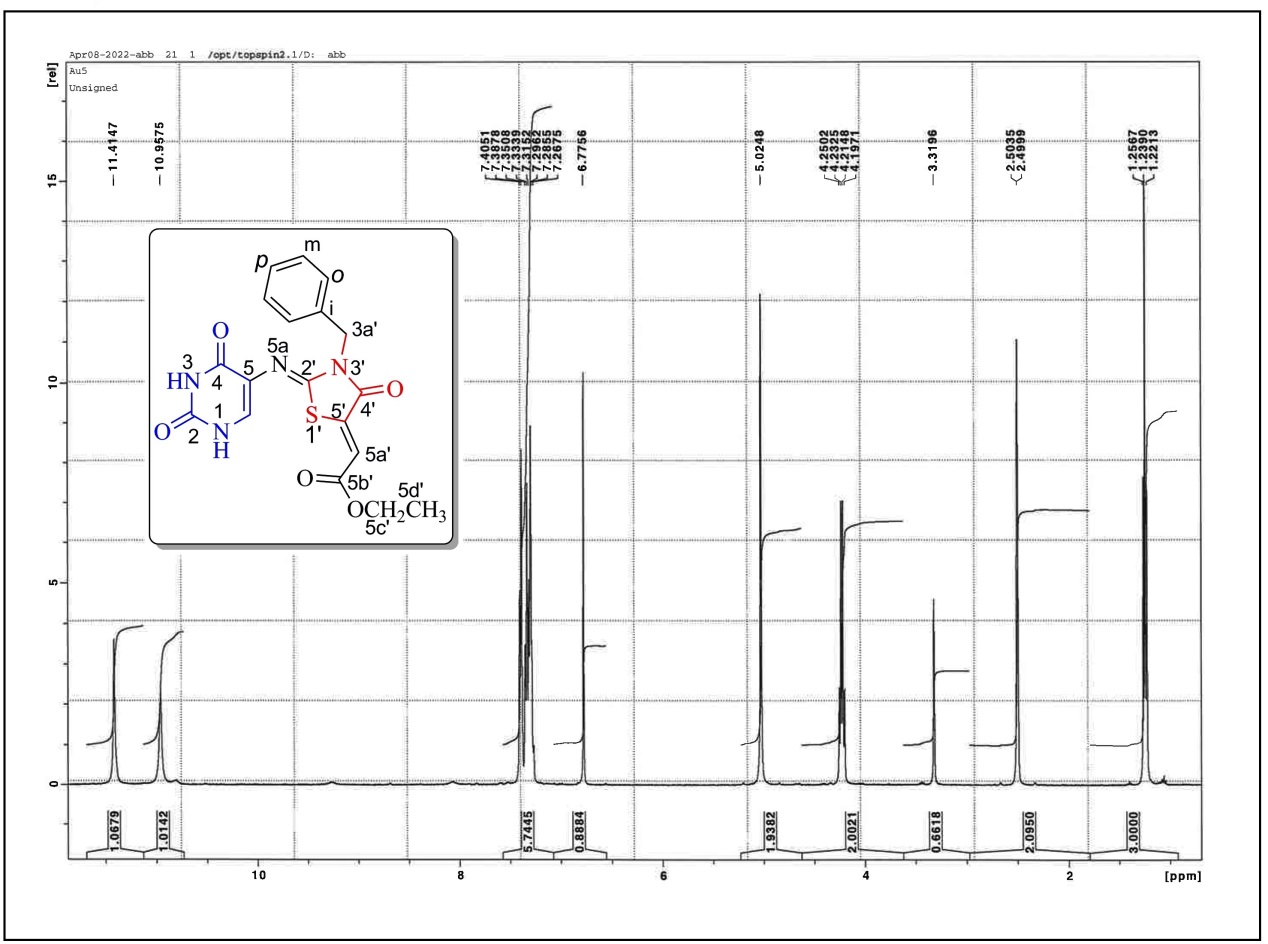


SI figure 91: ^1^H-NMR spectrum of **5d**


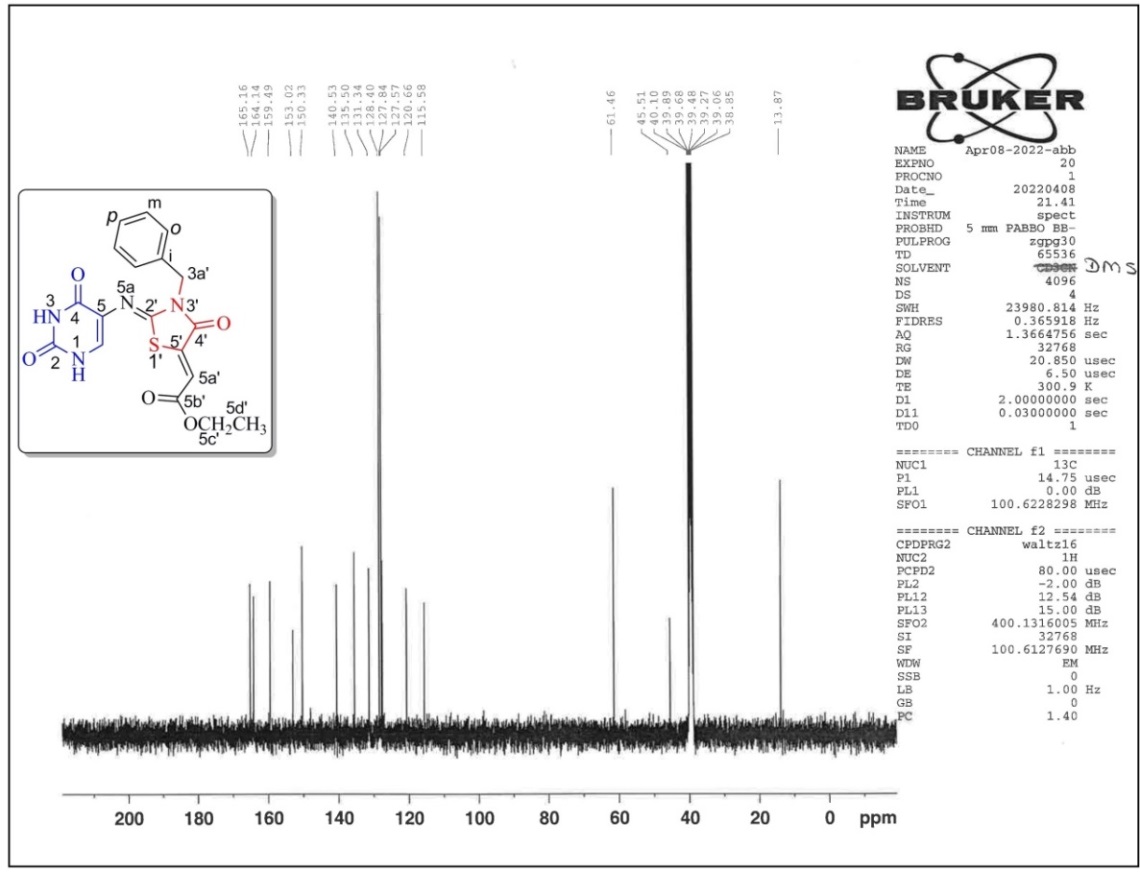


SI figure 92: ^13^C-NMR spectrum of **5d**


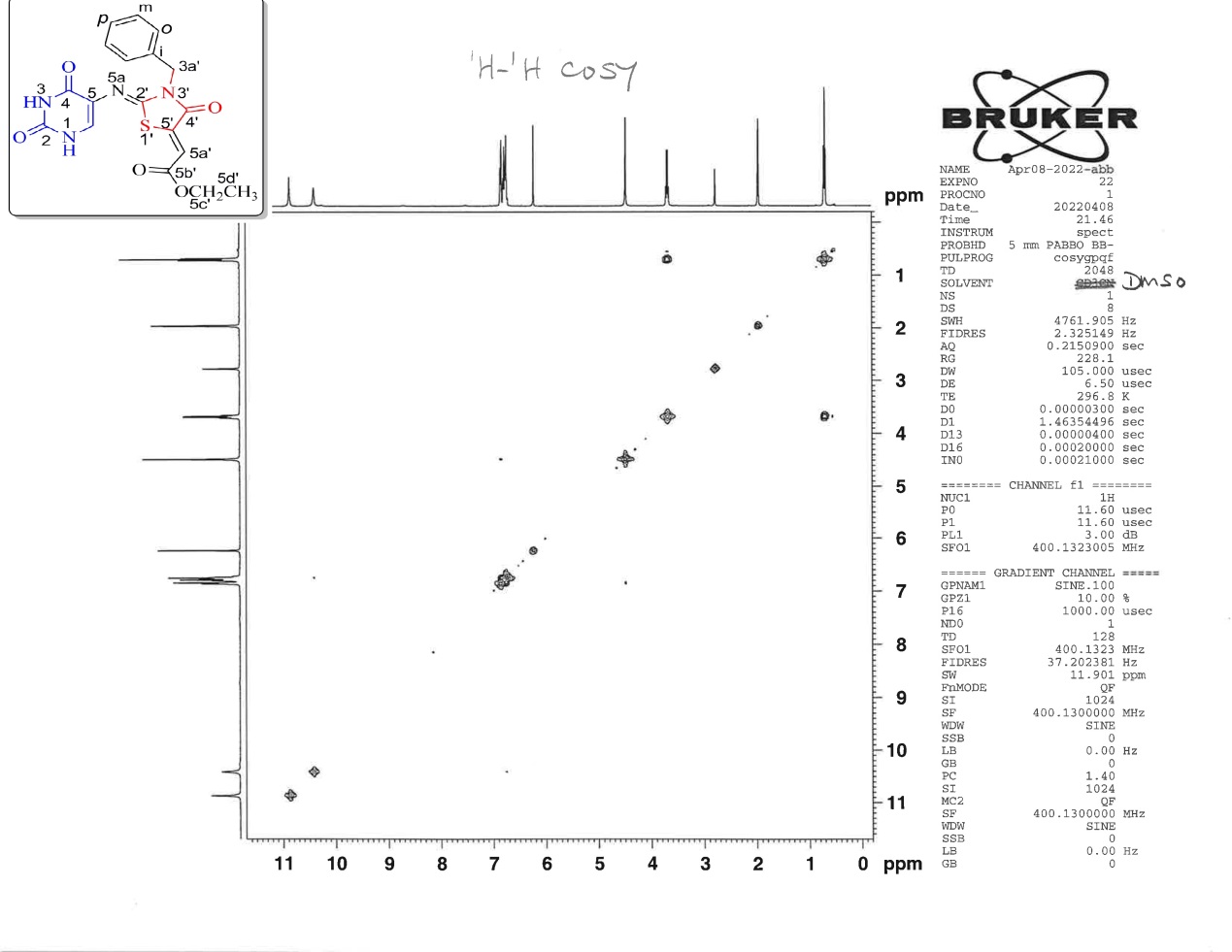


SI figure 93: ^1^H-H COSY spectrum of **5d**


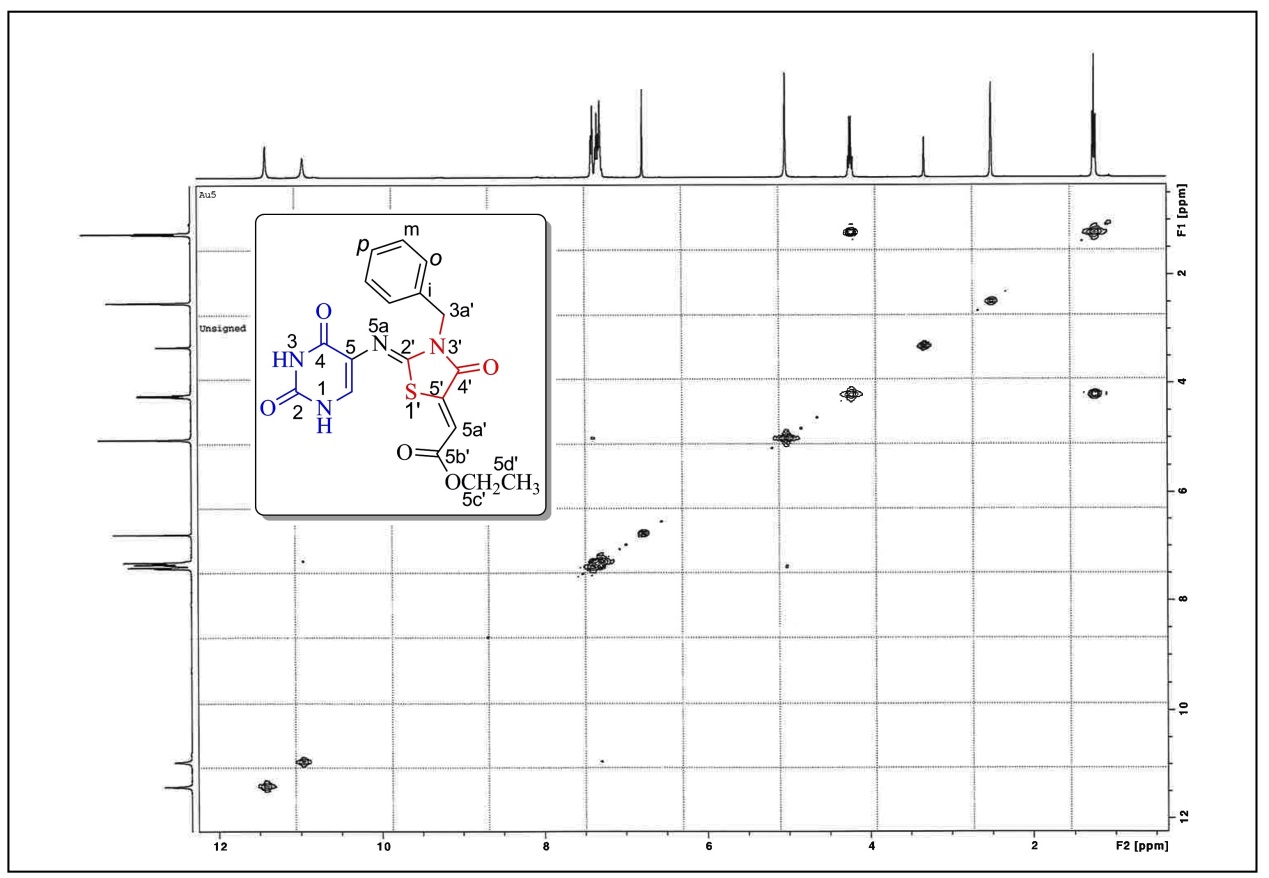


SI figure 94: ^1^H-H COSY spectrum of **5d**


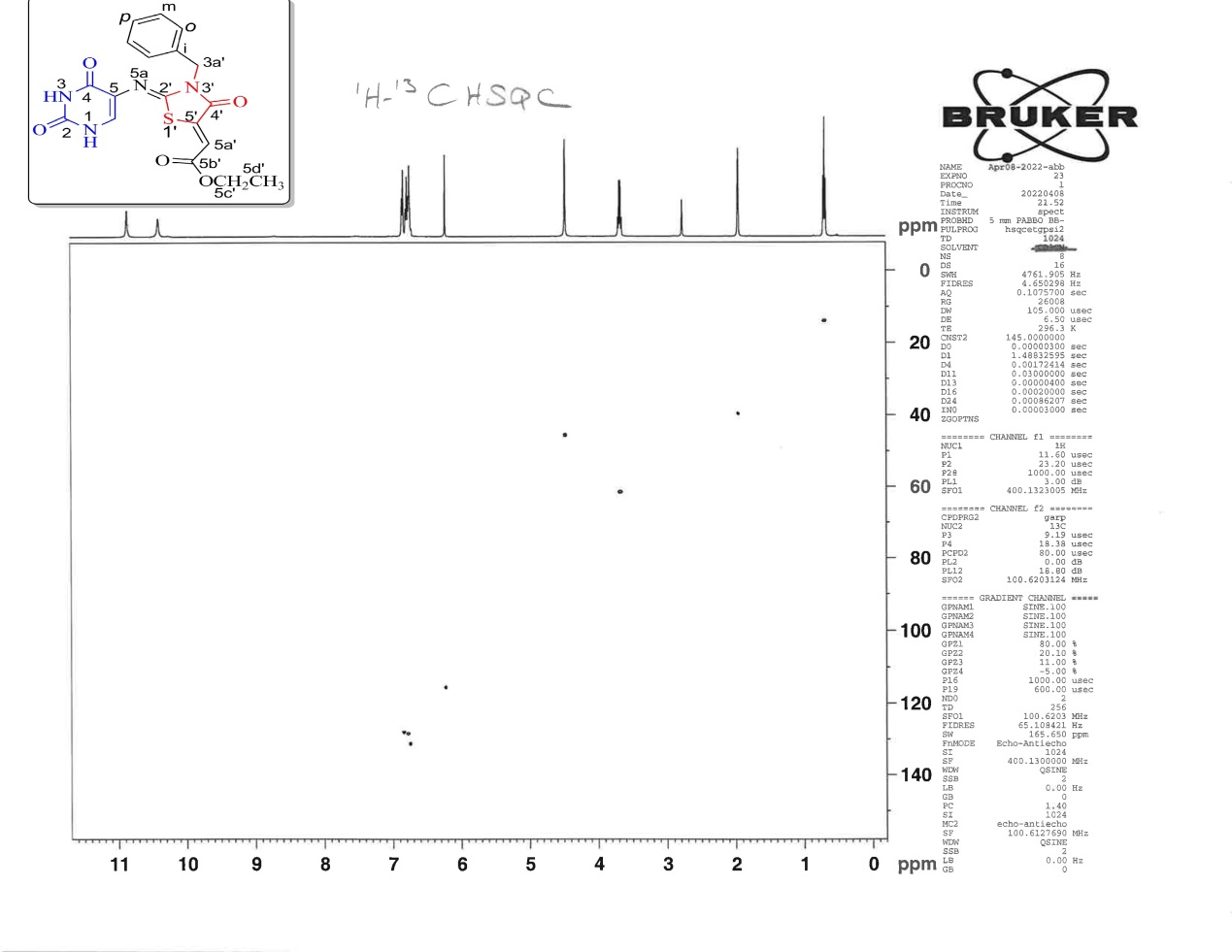


SI figure 95: ^1^H-^13^C HSQC spectrum of **5d**

SI figure 96: ^1^H-^13^C HSQC spectrum of **5d**

SI figure 97: ^1^H-^13^C HMBC spectrum of **5d**

SI figure 98: ^1^H-^13^C HMBC spectrum of **5d**

SI figure 99: ^1^H-^15^N HMBC spectrum of **5d**

SI figure 100: Mass Spectroscopy of **5d**

SI figure 101: ^1^H-NMR spectrum of **5e**

SI figure 102: ^1^H-NMR spectrum of **5e**

SI figure 103: ^13^C-NMR spectrum of **5e**

SI figure 104: ^1^H-^1^H COSY spectrum of **5e**

SI figure 105: ^1^H-^13^CHSQC spectrum of **5e**

SI figure 106: ^1^H-^13^CHSQC spectrum of **5e**

SI figure 107: ^1^H-^13^CHMBC spectrum of **5e**

SI figure 108: ^1^H-^13^CHMBC spectrum of **5e**

SI figure 109: ^1^H-NMR spectrum of **5e**

SI figure 110: ^1^H-NMR spectrum of **5e**

SI figure 111: ^13^C-NMR spectrum of **5e**

SI figure 112: ^1^H-^1^H COSY spectrum of **5e**

SI figure 113: ^1^H-^13^CHSQC spectrum of **5e**

SI figure 114: ^1^H-^13^CHSQC spectrum of **5e**

SI figure 115: ^1^H-^13^CHMBC spectrum of **5e**

SI figure 116: ^1^H-^13^CHMBC spectrum of **5e**

SI figure 117: ^1^H-^13^CHMBC spectrum of **5e**

SI figure 118: Mass Spectroscopy of **5e**

SI figure 119: ^1^H- NMR spectrum of **5g**

SI figure 120:^1^H- NMR spectrum of **5g**

SI figure 121:^1^H- NMR spectrum of **5g**

SI figure 122: ^1^H- ^1^H COSY spectrum of **5g**

SI figure 123:^1^H- ^1^H COSY spectrum of **5g**

SI figure 124:^13^C- NMR spectrum of **5g**

SI figure 125: ^1^H- ^13^C HSQC spectrum of **5g**

SI figure 126: ^1^H- ^13^C HSQC spectrum of **5g**

SI figure 127: ^1^H- ^13^C HSQC spectrum of **5g**

SI figure 128: ^1^H- ^13^C HMBC spectrum of **5g**

SI figure 129: ^1^H- ^13^C HMBC spectrum of **5g**

SI figure 130: ^1^H- ^13^C HMBC spectrum of **5g**

SI figure 131: ^1^H- ^15^N HSQC spectrum of **5g**

SI figure 132: ^1^H- ^15^N HMBC spectrum of **5g**

SI figure 133:^1^H- ^15^N HMBC spectrum of **5g**

SI figure 134:^1^H- ^15^N HMBC spectrum of **5g**

SI figure 135: ^1^H NMR Spectrum of **5j**

SI figure 136: ^1^H NMR Spectrum of **5j**

SI figure 137: ^13^C-NMR spectrum of **5j**

SI figure 138: ^1^H-^1^HCOSY spectrum of **5j**

SI figure 139: ^1^H-^13^CHSQC spectrum of **5j**

SI figure 140: ^1^H-^13^CHSQC spectrum of  **5j**

SI figure 141: ^1^H-^13^C-HMBC spectrum of  **5j**

SI figure 142: ^1^H-^13^C-HMBC spectrum of  **5j**

SI figure 143: ^1^H-^15^N-HSQC spectrum of  **5j**

SI figure 144: ^1^H-^15^N-HSQC spectrum of  **5j**

SI figure 145: ^1^H-^15^N-HSQC spectrum of  **5j**

SI figure 146: ^1^H-NMR spectrum of **5k**

SI figure 147: ^1^H-NMR spectrum of **5k**

SI figure 148: ^13^C-NMR spectrum of  **5k**

SI figure 149: ^1^H-^1^HCOSY spectrum of  **5k**

SI figure 150: ^1^H-^13^CHSQC spectrum of  **5k**

SI figure 51: ^1^H-^13^CHSQC spectrum of  **5k**

SI figure 152: ^1^H-^13^CHMBC spectrum of  **5k**

SI figure 53: ^1^H-^13^CHMBC spectrum of  **5k**

SI figure 154: ^1^H-^15^N-HSQC spectrum of  **5k**

SI figure 155: Mass Spectroscopy of  **5k**

SI figure 156: ^1^H-NMR spectrum of **5i**

SI figure 157: ^1^H-NMR spectrum of **5i**

SI figure 158: ^1^H-^1^H-COSY spectrum of **5i**

SI figure 159: ^1^H-^1^H-COSY spectrum of **5i**

SI figure 160: ^13^C-NMR spectrum of  **5i**

SI figure 161: ^1^H-^13^C-HSQC spectrum of  **5i**

SI figure 162: ^1^H-^13^C-HSQC spectrum of  **5i**

SI figure 163: ^1^H-^13^C-HMBC spectrum of  **5i**

SI figure 164: ^1^H-^13^C-HMBC spectrum of  **5i**

SI figure 165: Mass Spectroscopy of  **5i**

**Table S1.** Computed docking scores (in kcal/mol) and binding features for the synthesized compounds and controls against EGFR and BRAF^V600E^.

| **Compound Name** |  | **EGFR** | |  | BRAF^V600E^ | |
| --- | --- | --- | --- | --- | --- | --- |
|  |  | **Docking Score (kcal/mol)** | **Binding features (hydrogen bond length in Å) ^a^** |  | **Docking Score (kcal/mol)** | **Binding features (hydrogen bond length in Å)^a^** |
| **3c** |  | −8.0 | MET769 (1.84 Å),  CYS773 (2.84 Å),  ARG817 (2.13 Å),  ASP831 (2.01 Å) |  | −9.1 | ALA481 (3.22 Å),  ILE527 (3.49 Å),  GLN530 (1.97 Å),  CYS532 (1.82 Å) |
| **5b** |  | −8.3 | LYS721 (2.14 Å),  MET769 (2.09, 2.43 Å),  GLY772 (2.83 Å) |  | −11.8 | THR529 (2.04, 3.23 Å),  CYS532 (2.00 Å),  ASN580 (2.61 Å),  ASN581 (2.04 Å) |
| **5c** |  | −8.1 | MET769 (1.96 Å),  THR830 (2.70 Å) |  | −9.4 | LYS483 (2.18, 2.30 Å),  THR529 (1.72 Å),  CYS532 (2.19 Å),  ASN581 (2.49 Å),  ASP594 (1.78 Å) |
| **5h** |  | −8.0 | LYS721 (2.00, 3.38 Å),  MET769 (2.10, 2.46 Å),  GLY772 (2.79 Å) |  | −9.2 | THR529 (2.09, 3.24 Å),  CYS532 (2.92 Å),  ASN580 (2.66 Å),  ASN581 (2.05 Å) |
| **5i** |  | −8.0 | MET769 (1.98 Å),  THR830 (2.68 Å) |  | −9.2 | LYS483 (2.28 Å),  THR529 (1.68 Å),  CYS532 (2.12 Å),  ASN581 (2.51 Å),  ASP594 (2.81 Å) |
| **5j** |  | −8.2 | LYS721 (1.63, 2.96 Å),  THR766 (2.44, 2.84 Å),  GLN767 (2.29 Å),  MET769 (1.82 Å) |  | −10.5 | LYS483 (1.65 Å),  THR529 (2.51 Å),  ASN581 (1.80 Å),  ASP594 (2.08, 2.20 Å),  PHE595 (3.04 Å),  GLY596 (2.39 Å) |
| **Erlotinib** |  | −7.9 | MET769 (1.68 Å),  CYS773 (1.93 Å) |  | −7.8 | THR529 (2.14 Å), CYS532 (2.06 Å) |

^a^ Conventional hydrogen bond only (in Å) is registered.

**Appendix A**

**3. EXPERIMENTAL**

**Instrumentation**

All materials were obtained from commercial suppliers and used without further purification. Reactions were monitored by TLC (Kieselgel 60 PF_254_ precoated plates, E. Merck, Germany), and the spots were detected by exposure to a UV lamp at 254 nm. Melting points were determined on an electro-thermal melting point apparatus (Stuart Scientific Co.) and were uncorrected. NMR spectra were measured on a Bruker AV-400 spectrometer (Bruker Bio Spin Corp., Billerica, MA, USA) (400 MHz for 1H, 101 MHz for ^13^C) at the Florida Institute of Technology, USA. The 1H and 13C chemical shifts are given relative to internal standard TMS = 0., and external liquid ammonia = 0 for 15N. Coupling constants are stated in Hz. Correlations were established using ^1^H-^1^H COSY, and ^1^H-^13^C and ^1^H-^15^N HSQC and HMBC experiments. Mass spectrometry was recorded on a Varian MAT 312 instrument in EI mode (70 eV) at the Karlsruhe Institut für Technologie (KIT), Institute of Organic Chemistry, Karlsruhe, Germany.

**4.2. Biological evaluation**

**4.2.1. Cytotoxic activity using MTT Assay and evaluation of IC_50_**

**4.2.1.1. MTT assay**

MTT assay was carried out to study the effect of compounds on mammary epithelial cells (MCF-10A) (Youssif, B. G. M. et al., 2019; Mahmoud, M. A., et al., 2022). The medium in which cells were propagated contained Dulbecco's modified Eagle's medium (DMEM)/ Ham's F-12 medium (1:1) supplemented with epidermal growth factor (20 ng/mL), hydrocortisone (500 ng/mL), insulin (10 μg/mL), 2 mM glutamine and 10% foetal calf serum. After every 2-3 days, the cells were passaged using trypsin ethylenediamine tetra acetic acid (EDTA). The cells were seeded at a density of 10^4^ cells mL^-1^ in flat-bottomed culture plates containing 96 wells each. After 24 h, medium was removed from the plates and the compounds in (in 0.1% DMSO) were added (in 200 μL medium to yield a final concentration of 0.1% v/v) to the wells of plates. A single compound was designated with four wells followed by incubation of plates for 96h at 37°C. After incubation, medium was removed completely from the plates followed by addition of MTT (0.4 mg/mL in medium) to each well and subsequent incubation of plates for 3h. MTT (along with the medium) was removed and DMSO (150μL) was added to each well of the culture plates, followed by vortexing and subsequent measurement of absorbance (at 540 nm) using microplate reader. The data are shown as percentage inhibition of proliferation in comparison with controls containing 0.1% DMSO.

**4.2.1.2. Assay for antiproliferative effect**

To explore the antiproliferative potential of compounds MTT assay was performed according to previously reported procedure (El-Sherief, H.A.M., et al., 2018; Abdelrahman, M. H., et al., 2017) using different cell lines to explore the antiproliferative potential of compounds propidium iodide fluorescence assay was performed using different cell lines. To calculate the total nuclear DNA, a fluorescent dye (propidium iodide, PI) is used which can attach to the DNA, thus offering a quick and precise technique. PI cannot pass through the cell membrane and its signal intensity can be considered as directly proportional to quantity of cellular DNA. Cells whose cell membranes are damaged or have changed permeability are counted as dead ones. The assay was performed by seeding the cells of different cell lines at a density of 3000-7500 cells/well (in 200µl medium) in culture plates followed by incubation for 24h at 37 °C in humidified 5% CO_2_/95% air atmospheric conditions. The medium was removed; the compounds were added to the plates at 10 µM concentrations (in 0.1% DMSO) in triplicates, followed by incubation for 48 h. DMSO (0.1%) was used as control. After incubation, medium was removed followed by the addition of PI (25 µl, 50µg/mL in water/medium) to each well of the plates. At -80 °C, the plates were allowed to freeze for 24 h, followed by thawing at 25^o^C. A fluorometer (Polar-Star BMG Tech) was used to record the readings at excitation and emission wavelengths of 530 and 620 nm for each well. The percentage cytotoxicity of compounds was calculated using the following formula:

Where A*_TC_*= Absorbance of treated cells and A_C_= Absorbance of control. Erlotinib was used as positive control in the assay.

**4.2.1.3. EGFR inhibitory assay**

EGFR-TK assay was performed to evaluate the inhibitory potency of the tested compounds against EGFR (Mohamed, F. A. M., et al., 2021). Baculoviral expression vectors including pBlueBacHis2B and pFASTBacHTc were used separately to clone 1.6 kb cDNA coding for EGFR cytoplasmic domain (EGFR-CD, amino acids 645–1186). 5ʹ upstream to the EGFR sequence comprised a sequence that encoded (His)_6_. Sf-9 cells were infected for 72h for protein expression. The pellets of Sf-9 cells were solubilized in a buffer containing sodium vanadate (100 µM), aprotinin (10 µg/mL), triton (1%), HEPES buffer (50mM), ammonium molybdate (10 µM), benzamidine HCl (16 µg/mL), NaCl (10 mM), leupeptin (10 µg/mL) and pepstatin (10 µg/mL) at 0°C for 20 min at pH 7.4, followed by centrifugation for 20 min. To eliminate the non-specifically bound material, a Ni-NTA super flow packed column was used to pass through and wash the crude extract supernatant first with 10 mM and then with 100 mM imidazole. Histidine-linked proteins were first eluted with 250 and then with 500 mM imidazole after dialysis against NaCl (50 mM), HEPES (20 mM), glycerol (10%) and 1 µg/mL each of aprotinin, leupeptin and pepstatin for 120 min. The purification was performed either at 4 °C or on ice. To record autophosphorylation level, EGFR kinase assay was carried out based on DELFIA/Time-Resolved Fluorometry. The compounds were first dissolved in DMSO absolute, after dilution to appropriate concentration using HEPES (25 mM) at pH 7.4. Each compound (10 µL) was incubated with recombinant enzyme (10 µL, 5 ng for EGFR, 1:80 dilution in 100 mM HEPES) for 10 min at 25^o^C, after the addition of 5X buffer (10 µL, containing 2 mM MnCl_2_, 100 µM Na_3_VO_4_, 20 mM HEPES and 1 mM DTT) and ATP-MgCl_2_ (20 µL, containing 0.1 mM ATP and 50 mM MgCl_2_) and incubation for 1h. The negative and positive controls were included in each plate by the incubation of enzyme either with or without ATP-MgCl_2_. The liquid was removed after incubation and the plates were washed thrice using wash buffer. Europium-tagged antiphosphotyrosine antibody (75 µL, 400 ng) was added to each well followed by incubation of 1h and then washing of the plates using buffer. The enhancement solution was added to each well and the signal was recorded at excitation and emission wavelengths of 340 at 615 nm. The autophosphorylation percentage inhibition by compounds was calculated using the following equation:

Using the curves of percentage inhibition of eight concentrations of each compound, IC_50_ was calculated. Majority of signals detected by antiphosphotyrosine antibody were from EGFR because the enzyme preparation contained low impurities.

**4.2.1.4. BRAF kinase assay**

V^600E^ mutant BRAF kinase assay was performed to investigate the activity of tested compounds against BRAF (Mohassab, A. M., et al., 2021). Mouse full-length GST-tagged BRAF^V600E^ (7.5 ng, Invitrogen, PV3849) was pre-incubated with drug (1 µL) and assay dilution buffer (4 µL) for 60 min at 25^o^C. In assay dilution buffer, a solution (5 µL) containing MgCl_2_ (30 mM), ATP (200 µM), recombinant human full length (200 ng) and *N*-terminal His-tagged MEK1 (Invitrogen) was added to start the assay, subsequent to incubation for 25 min at 25^o^C. The assay was stopped using 5X protein denaturing buffer (LDS) solution (5 µL). To further denature the protein, heat (70° C) was applied for 5 min. 4-12% precast NuPage gel plates (Invitrogen) were used to carry out electrophoresis (at 200 V). 10 µL of each reaction was loaded into the precast plates and electrophoresis was allowed to proceed. After completion of electrophoresis, the front part of the precast gel plate (holding hot ATP) was cut and afterwards cast-off. The dried gel was developed using a phosphor screen. A reaction without active enzyme was used as negative control while that containing no inhibitor served as positive control. To study the effect of compounds on cell-based pERK1/2 activity in cancer cells, commercially available ELISA kits (Invitrogen) were used according to manufacturer’s instructions.

**4.3. Statistical analysis**

Computerized Prism 5 program was used to statistically analyzed data using one-way ANOVA test followed by Tukey’s as post ANOVA for multiple comparison at P ≤.05. Data were presented as mean ± SEM.
